# Which Reaction Conditions Work on Drug-Like Molecules? Lessons from 66,000 High-Throughput Experiments

**DOI:** 10.1021/acscentsci.5c02031

**Published:** 2026-02-05

**Authors:** Jesse Ahlbrecht, Marius D. R. Lutz, Vera Jost, Michael Färber, Stefan Bräse, Georg Wuitschik

**Affiliations:** † Institute of Biological and Chemical Systems, 150232 Karlsruhe Institute of Technology (KIT), 76131 Karlsruhe, Germany; ‡ Roche Pharma Research and Early Development, Roche Innovation Center Basel, 1529 F. Hoffmann-La Roche Ltd., 4070 Basel, Switzerland; § AI Center ScaDS.AI Dresden/Leipzig, TUD Dresden University of Technology, 01069 Dresden, Germany

## Abstract

High-throughput experimentation (HTE) accelerates chemical discovery by shortening the lead times for molecule synthesis. The choice of initial reaction conditions directly influences the outcome and length of any reaction optimization. But human involvement in plate design and data analysis remains a significant cost factor and is accompanied by biases. Therefore, making the most out of past reaction outcomes is crucial. While advances in machine learning allow us to generate promising reaction conditions, this approach is often not suitable because not enough relevant reaction data are available or it is of insufficient quality. Herein we introduce a robust statistical method using *z*-scores to analyze 66,000 internal HTE reactions on complex molecules. Additionally, we publish the underlying data as well as a tool to analyze and draw actionable conclusions from this data set. We exemplify the power of this method for the widely employed Buchwald–Hartwig and Suzuki–Miyaura cross-coupling reactions. The results reveal optimal conditions that differ significantly from literature-based guidelines. These data-driven insights provide high-quality starting points for optimization campaigns, improving their overall efficiency.

## Graphical abstract

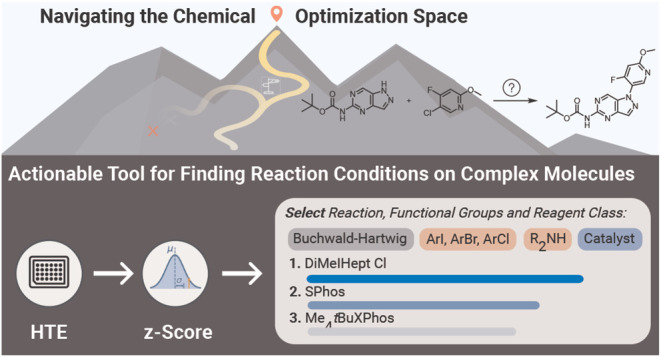

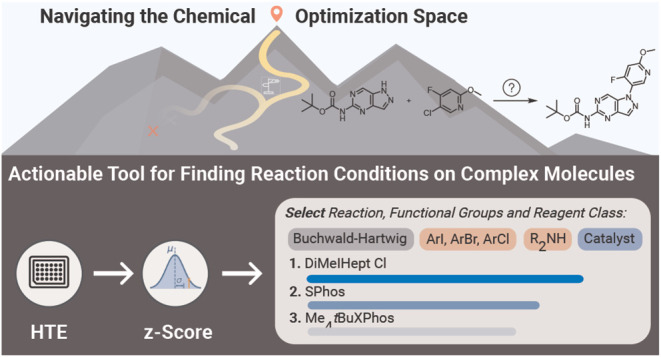

## Introduction

In recent years, the pharmaceutical industry has deployed high throughput experimentation (HTE) in their drug discovery pipeline to optimize small molecule chemistry and efficiently explore reaction condition spaces (Figure 1A).^1,2^ Dedicated HTE laboratories accelerate the pace of discovery by iteratively designing, executing, and analyzing experiments. In the process, HTE produces more data than traditional laboratories by running parallel reactions on miniature scale. This data can be used in different ways to make predictions for new reactions (Figure 1B). Recent work focuses on using HTE data to train machine learning prediction models.^3–7^ Despite their performance in train-test-split experiments, in productive use, humans continue to design the overwhelming majority of HTE plates.^8–10^ This indicates that there is room for a simpler, faster and more understandable way to use HTE data for arriving at optimization starting points, a need that was also recognized by other groups.^11^

**Figure 1:**
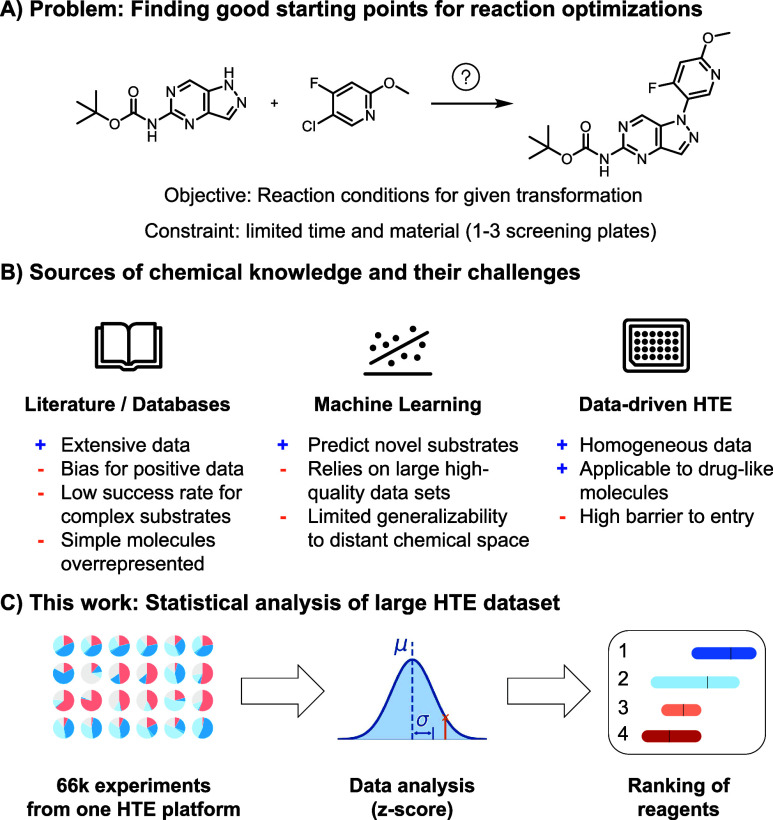
A) Reaction optimization for molecular synthesis is a complex problem and in the pharmaceutical industry material and time resources are limited. B) Starting points for an experimental screening campaign can arise from literature mining, machine learning algorithms or mining of experimental high-throughput experimentation data (HTE). C) In this work we showcase how statistical analysis of a large HTE-derived data set enables ranking of promiscuous reaction conditions.

In our HTE lab, we handle more than 200 chemical transformations per year, mainly from drug discovery and early process research. We use the HTE platform described by us,^12^ leading to about 25,000 new reaction outcomes every year. Our workflow involves several automated steps such as robotic solid dosing, analytics and data processing. Some steps like experimental design and data interpretation are still conducted manually.

Most transformations we receive failed to yield meaningful amounts of product before being submitted for screening. Thus, our data set is skewed toward difficult reaction types and substrate combinations, where small changes to the reaction conditions or substrate structure lead to big changes in outcome. Descriptors of the products of the reactions screened in this data set are shown in Figure S2 in the Supporting Information. We often receive advanced intermediates as starting materials and our customers expect us to use them resourcefully for a maximum number of experiments. Conversely, reducing the reaction scale too much can compromise result quality, specifically for heterogeneous reactions or those conducted close to the reaction medium’s boiling point. Therefore, we try to reduce the number of experiments by increasing the hit-rate and quality.

A good starting point for optimization is important in HTE, as campaigns are often constrained to a few (*n* = 1–3) plates with a large number of experiments each.^13^ We rarely receive enough starting material or time to run more than two screening plates. Thus, it is critical to have a simple, yet effective way of using past reaction data to produce viable hits on the first plate iteration.

While we follow the literature on new methodologies and conduct database searches for designing initial plates, we found that relying on our internal data translates to better reaction outcomes. This trend became more prevalent as the scale of our internal reaction data set increased. Large reaction databases sourced from patents and scientific papers, while diverse, are biased toward substrates with limited complexity. Moreover, many publications report only a few sets of positive results per substrate combination and reaction execution differs from lab to lab. Consequently, training machine learning models on this data becomes challenging.^8,14^ Thus, we see a need for a robust and explainable method utilizing real-world HTE data in human-centric and autonomous experiment design.

A successful method needs to tolerate outliers in the results and handle substrate diversity. In search of a data-driven analysis method that does not rely on heuristics or complex machine learning models, we arrived at *z*-score analysis, a simple statistical method (Figure 1C).^15–17^ It has recently been used in the context of evaluating other chemical transformations, but to the best of our knowledge, has not found application in large reaction optimization data sets.^18,19^ Z-scores measure how many standard deviations a given data point is away from the mean of the distribution. As a result, reaction components with exceptional performance are assigned a high *z*-score, compared to those on plates where many conditions are similarly effective. It also excels for reaction types with sparse reaction data.

## Results and Discussion

Our workflow integrates HTE data with statistical analysis. It enables the extraction of actionable reagent recommendations from a real-world data set. We publish a comprehensive data set without substrate structures, encompassing 66,000 reactions across 42 distinct reaction types. Of these, 13 reaction types each contain over 1,000 reactions. Alongside the data set, we publish the source code for an analysis tool that provides reagent recommendations by reaction type, reacting functional group and reagent category. We also host this tool as a web app at https://go.roche.com/zScoreApp. Herein we detail two case studies to demonstrate what users can expect when using the app: Buchwald–Hartwig (20,000 reactions) and Suzuki–Miyaura (11,000 reactions) cross-couplings, as they are the most prevalent reaction types in our collection and of particular relevance.

### Buchwald–Hartwig Cross-Couplings

Aromatic amines feature commonly as structural motifs in many fields of chemistry, including drug discovery. As many aryl halides are commercially available or easy to prepare, a methodology that couples them robustly with nitrogen nucleophiles under mild conditions has attracted enormous interest.^20^ While copper- or nickel-catalyzed as well as uncatalyzed versions of this transformation exist, the palladium-catalyzed Buchwald–Hartwig reaction still has the broadest substrate scope.^21,22^ We also see in our screenings that the majority of hits are palladium-based, despite the recent advances in ligands for Ullmann-type couplings.^23–26^

Figure 2 illustrates the coverage of the Buchwald–Hartwig reaction space in our data set, showing the frequency across both nucleophile and electrophile types. Aryl bromides and aryl chlorides are the two main electrophile types. While nucleophile types are more evenly distributed, primary aliphatic amines and primary anilines are the two most frequently observed. The distributions of both electrophiles and nucleophiles resembles those in the literature.^27^

**Figure 2:**
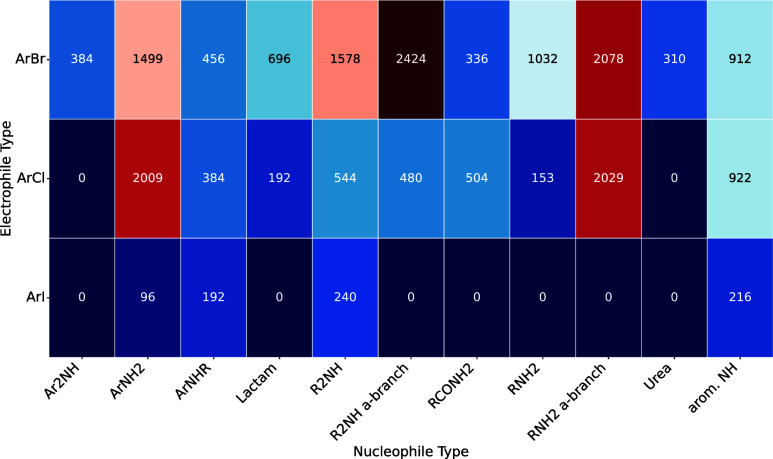
Electrophile–nucleophile combination matrix for Buchwald–Hartwig cross-couplings. Each element shows the number of reactions for that combination of electrophile/nucleophile type. Phenols and aryl sulfonates are excluded from the visualization due to very low counts in the data set. R refers to alkyl groups in this context, a-branch indicates substituents alpha to the carbon connected to the nitrogen.

Our tool allows the user to visualize the data by selecting functional groups, reaction and reagent types. The *z*-score distribution for the best reagents is then presented as a series of box-plots.

When comparing ligands for Buchwald–Hartwig cross-couplings involving aryl halides reacting with unhindered secondary aliphatic amines, several differences to what has been observed in literature analyses emerge (Figure 3). The structures of all ligands used in palladium-catalyzed couplings can be found in Figure S1. The NHC ligand DiMeIHept Cl^28^ leads in performance but is used relatively infrequently because of its higher cost compared to other chlorocarbene ligands such as IPent Cl.^29^ The dialkylbiaryl phosphine ligand SPhos^30^ ranks second, but its distribution with two distinct maxima suggests a substrate dependency not captured by our coarse categorization. Looking at the underlying substrate structures, it appears as if aryl halides containing five-membered heterocycles are responsible for the cluster of underperformance whereas SPhos performs well with more activated six-membered ring heteroaryl halides.

**Figure 3:**
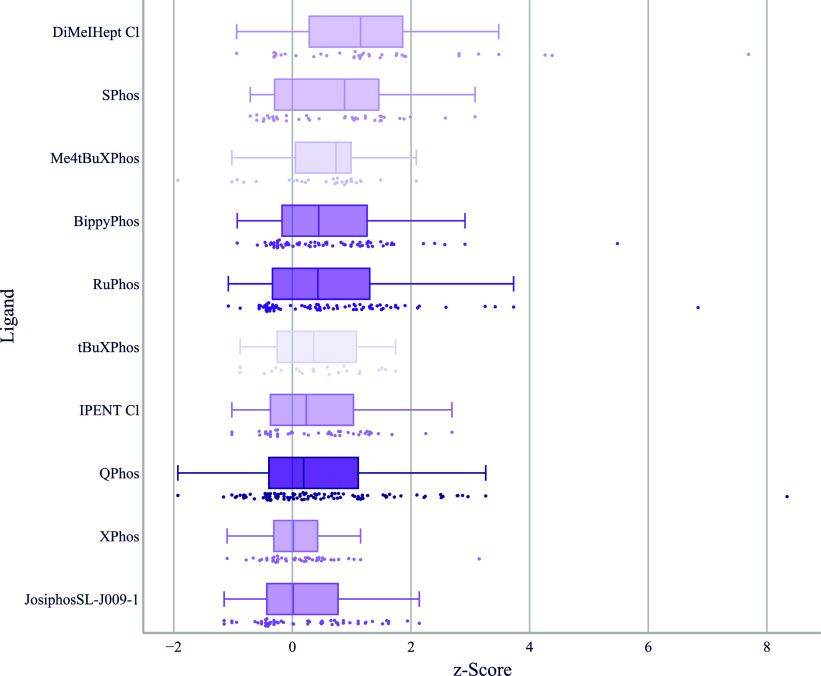
Boxplot of *z*-score by ligand for aryl halides reacting with unhindered secondary aliphatic amines. The top 10 ligands that are shown were used in at least five different chemical transformations and the top five hits per transformation were considered for each ligand. Below each boxplot we show all underlying data points. We use a color gradient to indicate the number of reactions, thereby highlighting ligands that are supported by a greater number of data points.

While SPhos was the preferred ligand for this use case in a study by Ingoglia et al.,^31^ it ranked below other ligands in our own previous work.^27^ For our substrates, SPhos performs remarkably well, only surpassed by DiMeIHept Cl. Me_4_^*t*^BuXPhos,^32^ despite being used less often for this use case by us and others, shows a consistent overperformance compared to the more frequently used BippyPhos and RuPhos ligands. It is also notable that BrettPhos (i.e., with cyclohexyl substituents) significantly outperforms ^*t*^BuBrettPhos for this substrate category. Other nitrogen nucleophiles are coupled more efficiently using ^*t*^BuBrettPhos or AdBrettPhos. Surprisingly, QPhos, despite being the most frequently used ligand in this subset and being the top ligand in our previous analysis^27^ of literature Buchwald–Hartwigs of this type, ranks only eighth for secondary amines.

Our lab solely relies on Pd-precatalysts and for this analysis, all precatalysts of a given ligand are treated as being equivalent, although the tool offers the option to consider them individually. While the ligand has an outsized effect on reaction outcomes in many cases, analysis at the level of individual catalysts reveals differences (Figure 4). It is striking that SPhos as a ligand ranks so much higher when the precatalyst type is not considered. The reason for that is that we use SPhos in the form of SPhos Pd­(allyl)­OTf in two Buchwald–Hartwig couplings of secondary amines. In these it performed so well that it would have topped the list with a median *z*-score of 1.36, if it had occurred in the data set five times or more. This is the default setting for the minimum number of transformations a reagent has to occur in for it to be shown. SPhos Pd­(crotyl)Cl only achieves a median *z*-score of 0.55. Pooling the two results in the observed overall median *z*-score of 0.95 for SPhos as a ligand. This performance variance aligns with reports that steric bulk on the allyl ligand prevents Pd­(I)-dimer formation^33^ as well as the switch from chloride as a counterion to triflate.^34^ We believe, however, that data from two transformations are not enough to make a general statement, especially since in none of the transformations the two SPhos precatalysts were compared head-to-head. We observe that Pd-PEPPSI-IPent Cl *o*-picoline is ranked third place, while Pd-PEPPSI-IPent Cl 3-chloropyridine ranks far below. A similar split can also be observed for ^*t*^BuXPhos, where the G3 precatalyst we use performs worse than the corresponding allyl triflate.^35^ Similar differences have been observed before.^36^ We have to caution, however, that these differences could also result from batch quality differences or biases present in catalyst selection and reaction substrates. In our laboratory, we minimize unexplained batch-to-batch variability by implementing a batch-tracking system, but use too few different batches of each catalyst for being able to track batch-to-batch influence.

**Figure 4:**
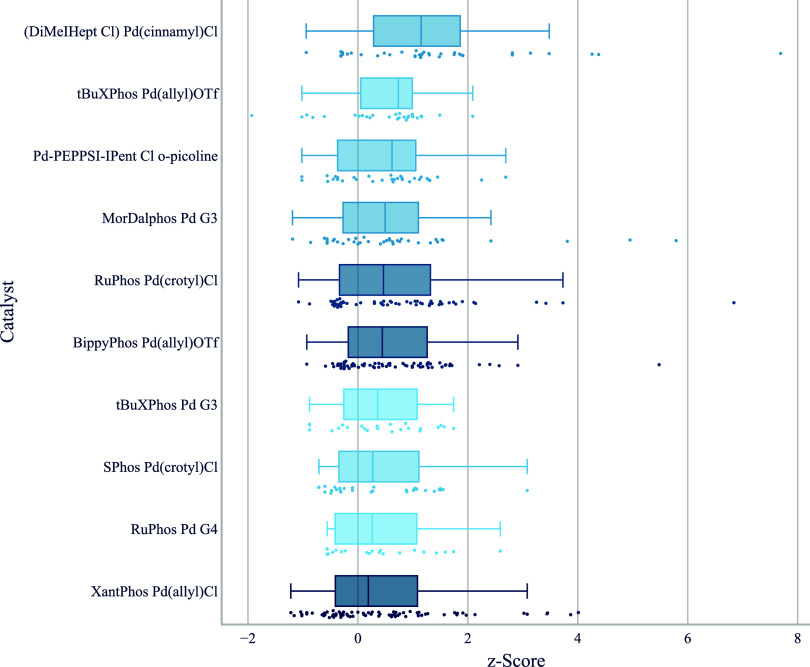
Boxplot of *z*-score by catalysts for aryl halides reacting with unhindered secondary aliphatic amines. The top 10 catalysts that are shown were used in at least five different chemical transformations, and the top five hits per transformation were considered for each catalyst. Below each boxplot we show all underlying data points. We use a color gradient to indicate the number of reactions, thereby highlighting catalysts that are supported by a greater number of data points.

### Comparing our Buchwald–Hartwig Recommendations with the Literature

When chemists set up and optimize new reactions, they typically consult scientific literature and reaction databases. To evaluate the difference between using literature review and our real-world HTE data-driven tool, we compared the best ligands by *z*-score to the evaluation of Buchwald–Hartwig literature published in our previous work.^27^ For this we used the decision tree in the latter and compared those top three recommendations for various amine classes to our experimentally best performing ligands. The literature recommendations are based on the median yield of the ligand, ours on the ligand’s median *z*-score. As shown in Figure 5, there is minimal overlap between the two sets. For several of the previous recommendations, we lack sufficient examples to assess their performance. In the cases where we can draw comparisons, the recommendations underperform in our data set. There is only one shared condition among the top three recommendations across all reaction types. While other classical ligands like Xantphos or BINAP still rank high in several categories, we observe that commonly utilized ligands, including triphenylphosphine, dppf or XPhos, rank low in our data set compared to their more modern counterparts.

**Figure 5:**
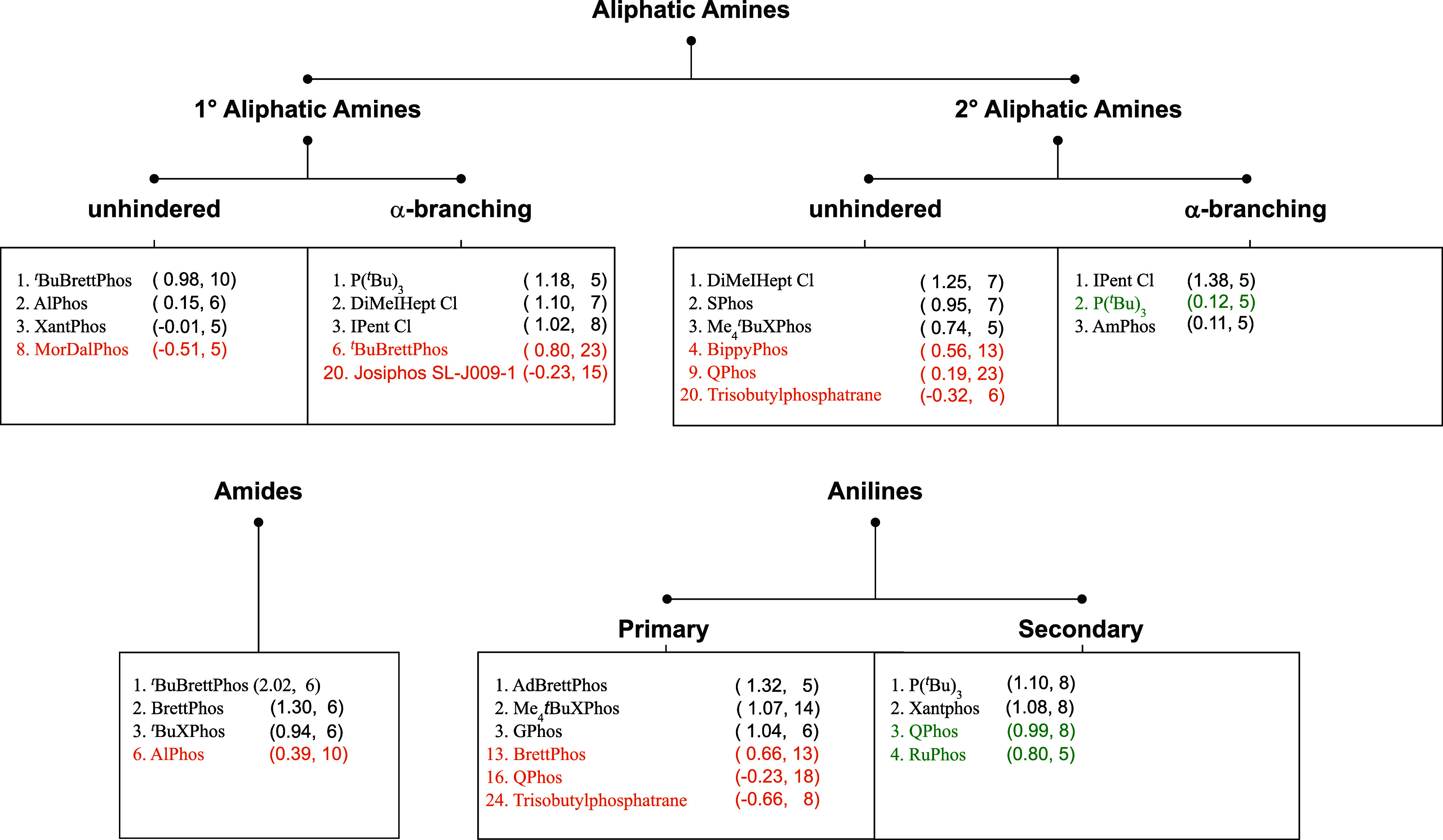
Hierarchical tree diagram illustrating the recommended Pd ligands for the Buchwald–Hartwig cross-coupling of aryl halides with three primary nucleophile classes: aliphatic amines, amides, and anilines. Ligand recommendations are based on *z*-score across a minimum of five chemical transformations for each substrate category (*z*-score and number of experiments in brackets). Recommendations previously published by us when analyzing literature data are indicated in orange if they do not overlap, and green if they are shared in both sets.^27^

The perceived increased substrate complexity compared to typical literature substrates may be the reason why we observe differing solvent and base preferences for the coupling of unhindered secondary aliphatic amines. We see Cs_2_CO_3_ outperforming more commonly used strong bases like NaO^*t*^Bu or KHMDS (see the Supporting Information, Figure S42). We also observe that more polar solvents like ^*t*^AmOH or propionitrile show excellent performance and compare favorably to toluene or dioxane which are more commonly used in the literature (see the Supporting Information, Figure S45). These observations may serve as another indication that the transformations in our data set differ significantly from the ones in the literature.

### Suzuki–Miyaura Cross-Couplings

In Suzuki–Miyaura cross-couplings involving an aryl halide reacting with an arylboronic acid, arylboronic ester, or aryltrifluoroborate, we observe fewer deviations from literature-known optima with respect to catalysts (Figure 6). We observe that monodentate electron-rich phosphines like cataCXium A,^37^ AmPhos^38^ and P­(^*t*^Bu)_3_ perform best, followed by IPent Cl and a range of commonly used bidentate ligands like dtbpf or Xantphos. SPhos, another commonly used ligand for Suzuki–Miyaura couplings, ranks lower. We again observe an influence of the precatalyst activation system for example in the different performance of precatalysts containing CataCXium A. In our setup, an aqueous base as well as the boronate are almost always present from the beginning, providing a path to Pd(0) via nucleophilic attack, reductive elimination or reduction.^39^ But we nevertheless observe that CataCXiumA Pd G6 Br dimer is clearly superior to CataCXiumA Pd­(allyl)Cl and the G3 variant. In contrast to Buchwald–Hartwig reactions of secondary amines, PdCl_2_ precursors of Xantphos and dppf are superior to their allyl and G3 counterparts. It has been reported that these PdCl_2_ precursors react to the frequently catalytically more active monophosphine oxide Pd(0) species.^40,41^ This may be an explanation for the lower performance of other precursors that would result in the bisphosphine Pd(0) species.

**Figure 6:**
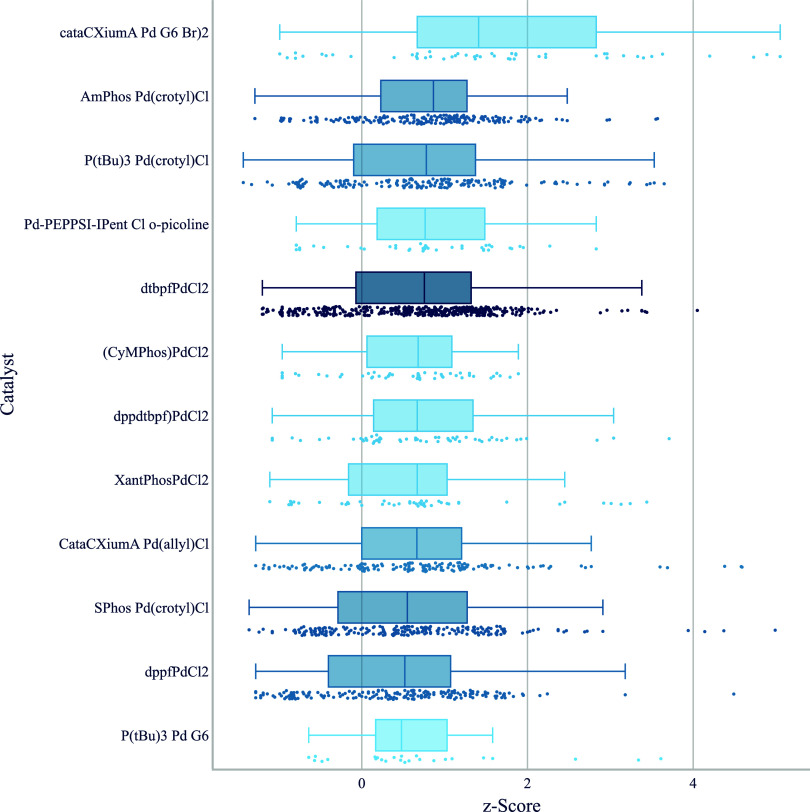
Boxplot of *z*-score by catalyst for aryl halides reacting with different types of aryl boronates. The top 12 catalysts that are shown were used in at least five different chemical transformations, and the top five hits per transformation were considered for each catalyst. Below each boxplot we show all underlying data points. We use a color gradient to indicate the number of reactions, thereby highlighting catalysts that are supported by a greater number of data points.

For Suzuki–Miyaura couplings the right combination of solvent and base can be as important as the right catalyst (Figure 7). One reason may be the importance of bridging the polarity differences between the aryl boronate, aryl halide and the catalyst.^42^ To facilitate transmetalation of the boron species, water is routinely added. In our data set, inorganic bases are used as aqueous solution with the exception of Cs_2_CO_3_ where we commonly either use no water or add only a few equivalents. We use these conditions in Suzuki–Miyaura couplings to prevent hydrolysis of sensitive functional groups like esters. Another way to achieve this is to use a weaker base like aqueous NaHCO_3_ or a less polar organic solvent like toluene or isopropyl acetate. The prominent representation of Cs_2_CO_3_, NaHCO_3_ and water-immiscible solvents in Figure 7 is likely attributed to the prevalence of sensitive substrates we receive for screening.

**Figure 7:**
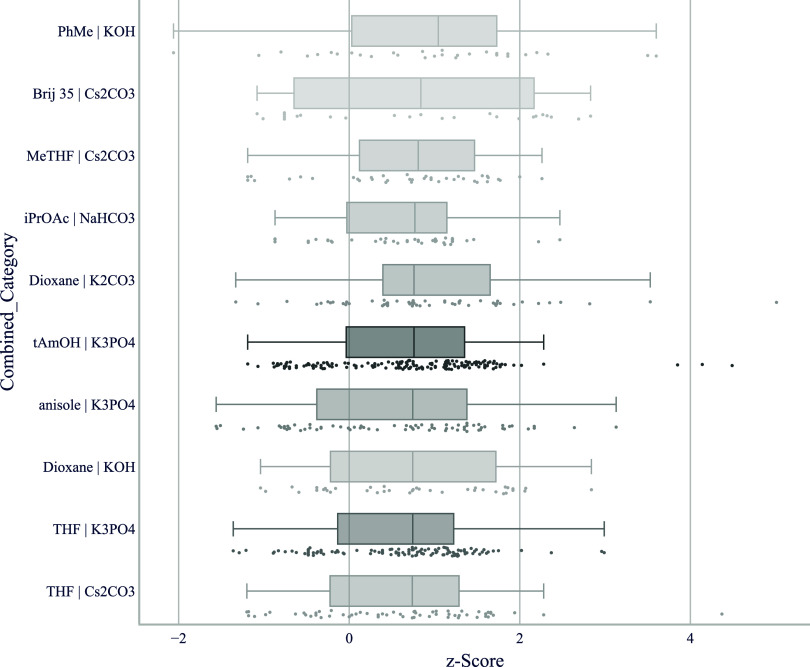
*z*-score boxplot of solvent/base-combinations for aryl halides reacting with different types of aryl boronates in Suzuki–Miyaura couplings. The top 10 base–solvent combinations that are shown were used in at least five different chemical transformations, and the top five hits per transformation were considered for each catalyst. Below each boxplot we show all underlying data points. We use a color gradient to indicate the number of reactions, thereby highlighting bases that are supported by a greater number of data points.

In order to exemplify the usefulness of this tool compared to existing approaches, we selected two pairs of available sterically hindered amines and bromopyridines, expecting the resulting Buchwald–Hartwig couplings to be difficult in order to obtain a large effect size. For both coupling reactions, we then designed one plate each using the *z*-score app, an AI-model for Buchwald–Hartwig reactions published by the Denmark group^4^ and literature precedent, making sure that the literature precedent plates were designed by a different co-worker than the others.

In the reaction of 3,3-dimethylmorpholine with 3-bromo-2-methylpyridine, partial conversion to product was only observed on the *z*-score plate using chloro carbene-based catalysts in the presence of NaO^*t*^Bu in either toluene or MeTHF. For the reaction of 2,5-dimethylpyrrole with 3-bromoisonicotinaldehyde, we found no hits for any Pd- or Cu-catalyzed reaction condition. The detailed results can be found in the Supporting Information.

### Caveats

While extensive, our data set is not a random sampling of the chemical space and is subject to several inherent biases that influence its composition. First, a survivorship bias exists, as the reactions included are primarily “difficult reactions” where initial attempts by the submitting chemist failed. Transformations solvable through routine literature searches or standard recommended reaction conditions therefore tend to be underrepresented. Thus, we would expect successful hits from these reaction conditions to be less frequent in the data set and these would thereby have lower *z*-scores. We therefore expect that the recommended conditions tend to favor modern catalysts and milder reaction conditions (i.e., more expensive catalysts, more polar solvents and bases with lower p*K*_A_) compared to literature-sourced ones explored in our previous work.^27^

Second, we choose reagents for a plate design not solely because of their expected performance. Other factors such as cost, availability on scale or workup considerations can prevent an otherwise superior reagent from being included. As these reagents may be used for discovery chemistry customers or after other options have failed, their *z*-score may be reduced. Similarly, newly added reagents are initially underrepresented and have to be consciously included in plate designs until enough data points are available for them to be included in our tool.

Third, we rarely receive product reference material from our customers. Thus, we use reduced peak area percent of product (vide infra) to measure reaction success at the level of chemical transformations. While inferior to actual yields, *z*-scores computed from these product peak area percentages are still comparable across chemical transformations and therefore fulfill their function of ranking reagents.

Finally, pharmaceutical projects often span multiple years and involve numerous molecules grouped around a common scaffold. This can result in the overweighing of similar optimal reaction conditions found for similar substrate pairs. As a result, recommendations for reaction types that contain more of these similarity islands may be flawed. With these caveats in mind, this data set nevertheless offers unique insights into practical chemical discovery.

A necessary caveat when interpreting data sets derived from HTE campaigns is the challenge of translatability to conventional bench-scale synthesis. This challenge arises from differences in heat and mass transfer, mixing efficiency, degree of solid grinding and surface area-to-volume ratios between micro- and gram scales. However, it cannot be overstated that HTE primarily serves to identify good starting points for process optimization; robust and scalable conditions require dedicated follow-up optimization at the bench. In our hands the most successful HTE conditions qualitatively reproduce the optimal outcome (e.g., product/selectivity) on larger scale. Moreover, we could show that calibrated peak area percentages agree with isolated yields of scale-ups in a quantitative case study.^43^

## Conclusion

In this study, we have developed and applied a *z*-score-based statistical methodology to effectively analyze a real-world HTE data set from a pharmaceutical drug discovery environment. Our analysis provides data-driven recommendations that challenge and refine conventional wisdom, particularly for challenging transformations like the Buchwald–Hartwig amination, where we identified a performance hierarchy of ligands substantially different from literature-based guides. This approach offers a simple and explainable tool for chemists to make more informed decisions, ultimately increasing hit rates and conserving precious starting materials. By bridging the gap where complex machine learning models may fail due to data sparsity, our methodology and the accompanying tool represent a significant practical step toward more intelligent laboratory workflows. This work provides a crucial feedback component for iterative experimental design and will provide better starting points for reaction optimization of complex transformations.

## Methodology

Herein, we publish a comprehensive data set without substrate structures, encompassing 66,000 reactions across 42 distinct reaction types. Of these, 13 reaction types each contain over 1,000 reactions. Box plots for the other reaction types can be found in Figures S25–S65 in the Supporting Information. On average, for each transformation 113 experiments were conducted and each experiment was sampled 2–3 times. A distinguishing feature of this data set is its almost exclusive focus on transformations of highly functionalized molecules within a drug discovery context, setting it apart from many existing literature data sets.

Our data processing workflow is as follows: First, we calculate the *z*-scores of all experiments in a given chemical transformation. Then we calculate the median of the top *n* *z*-scores in which a given reagent was present. When *n* is large, reagents are preferred that show good performance under a variety of conditions. When *n* is small, individual examples of large significant outperformance are weighted higher. By default, *n* is set to five which in our experience strikes a good balance between selecting for robust performance (large *n*) of a reagent and rewarding reagents for extraordinary performance under specific conditions (small *n*).

### Yield Calculation and Evaluation

Accurate evaluation of reaction outcomes is essential for conducting robust data analysis in chemistry. The gold standard is isolated yield with full product characterization in terms of identity and purity. We loosen these standards in HTE in order to increase throughput. To achieve this, we use area percent measurements from liquid chromatography–mass spectrometry (LC–MS) analyses as a proxy (Figure 8).

**Figure 8:**
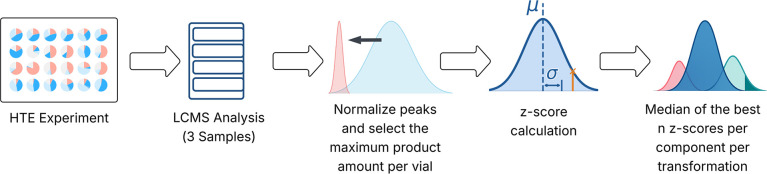
A schematic representation of the data flow of the tool. LC–MS peak area percentages are normalized and *z*-scores calculated using the maximum product amount measured in each vial. The median is then calculated based on the top *n* *z*-scores of all vials in a transformation that contain a given reagent.

We include an internal standard in each reaction to calculate a relative yield for all reaction components, which is helpful to compare hits beyond raw peak area percentages. The caveat of this method is an increased measurement error which can result in outliers. These errors may stem from the sampling of heterogeneous reaction mixtures, solid dosing or peak integration. Also, yields relative to an internal standard would require normalization to allow for comparisons across different chemical transformations. Finally, we rarely receive product samples for calibrations that would allow us to calculate absolute solution yields.

To arrive at a robust measure of reaction outcome, we instead opt to transform peak area percentages. We renormalize the measured peak area percent to exclude injection peaks and all peaks resulting from reagents, solvents or internal standards. This is accomplished by recalculating peak area percentages from the raw peak areas while omitting all peaks that do not result from starting materials or products. Typically, we sample each reaction at three different time/temperature points. The maximum observed value is then included in the final data set. The exact time–temperature profile is displayed in the web app when hovering over a data point. We only include plates for which at least one set of conditions resulted in a peak with more than ten area percent normalized product.

We demonstrate the predictive power of reduced peak area percentages in a recent publication on the Pd-catalyzed coupling of barbituric and Meldrum’s acid with aryl halides.^43^ In it, we showed for 14 examples that high, medium and low peak area percentages in HTE translate well, upon scale-up in the lab, into peak area percentages and finally isolated yields. We observed that chemical instability during workup or high hydrophilicity preventing extraction can reduce isolated yields but did not break the link between HTE results and outcomes in the laboratory. This translatability is further supported by benchmark studies from other HTE groups, which demonstrated that the quantitative conversions are maintained when scaling from miniaturized 20 μmol HTE vials to preparative 8 mL vials.^44^

One of the drawbacks of this method is that errors are introduced if not all side products are integrated and if the composition of side products differ significantly among the conditions tested. Furthermore, differences in UV-absorption coefficients between starting materials and products will influence peak areas and complicate comparison within and across chemical transformations. Nevertheless, we found these normalized peak area percentages to be more robust than relative yields. We calculate the latter by multiplying the ratio of product peak area and internal standard with the ratio of actual weights dosed of internal standard and limiting starting material. This relative yield is useful for comparing hits within a transformation but also prone to produce large outliers introduced through weighing or integration errors. Thus, we prefer to use normalized peak area percentages to evaluate and compare reaction outcomes within a chemical transformation.

### *z*-Score Methodology for Performance Assessment

Defining a metric that is both comparable across transformations and mapping the complexity of a “good” hit requires accounting for the context of other results within the same transformation. For instance, 30% product peak can be superior to 80%, if the former was achieved in a transformation with no other hits and the latter in a transformation where several 90% hits were found.

We base our metric on the *z*-score:

1 $$z=\frac{x-\mu }{\sigma }$$

where

- *z* is the standard score
- *x* is the observed area %
- μ is the mean area % for the given substrate pair
- σ is the corresponding standard deviation

*z*-scores take these differences into account by comparing an individual result with the mean of all outcomes of a chemical transformation, normalized with the outcome distribution’s standard deviation (eq 1). Screening hits that are among a large number of failed reactions will receive a high *z*-score compared to a hit that is one of many in a given transformation.

Using the *z*-score we calculate the rank of the reagent in the following way:

$$Rank\left(r\right)=median\left(Z_{r,topn}\right)$$

where

- *R* is the set of all reagents and reagent combinations (for clarity only called reagent in the following)
- *T* is the set of all chemical transformations
- *Z*(*r*,*t*) is the *z*-score of a reaction involving reagent *r* ∈ *R* and transformation *t* ∈ *T*
- *Z*_*r*_ = {*Z*(*r*,*t*)|*t*∈*T*}­is the set of all *z*-scores for reactions involving reagent *r* ∈ *R*
- *Z*_*r*,*topn*_ is the set of the *n* highest *z*-scores in *Z*_*r*_
- *n* is the user-defined number of top *z*-scores to consider
- *Rank*(*r*) is the final rank of reagent *r*

We allow the user to control the number of top *z*-scores to be included, because the user’s preference may vary. Using a large number of *z*-scores will favor reagents that work robustly under a variety of conditions whereas picking only the top one or two *z*-scores will favor reagents that work outstandingly well, but only under certain conditions. This equips the user with more granular control, especially in combination with setting a minimum number of transformations in which a reagent has to have been tested. While we only include the top *z*-scores of a reagent, we do not require them to be positive. This means that positive as well as negative performance of a reagent in different transformations is taken into account. We aim to limit the influence of outliers on the reagent ranking by using the median of the *z*-scores instead of the average.

Empirically, we set the minimum number of transformations as well as the number of *z*-scores to be included to five. Importantly we do not include all *z*-scores per category and transformation. This is critical as we search for the optima of the reaction space and therefore disregard the minima. Users can influence the displayed ranking by filtering out reagents that were not used in a minimum number of transformations. The minimum number of transformations in which a reagent has to be present in order to be displayed prevents outliers from being displayed that showed good results only in a small number of transformations. In some cases, users may be interested in these outliers and thus reduce that number. This can be because even rarely used reagents should be tried or because the specific combination of transformation, reacting functional groups and component roles contains few data points.

We generally advise the user to avoid excessive data slicing when using our tool. Instead, it is preferable to focus on a single category selection and the specific reacting functional groups that one is interested in. Other reaction types can be explored in the web app or by exploring the raw data provided in the Supporting Information.

One important caveat for interpreting the results is that the underlying distributions are non-normal. They exhibit substantial positive skew (median skewness = 1.36) and none of the reaction types pass the Shapiro–Wilk normality test (α = 0.05) (further information is available in the Supporting Information sections: Data set Information per Reaction Type and Underlying Distribution Histograms). This skewness is inherent to challenging reaction data sets. Although *z*-scores can be computed for any distribution, they do not permit probabilistic inference when normality is violated. In this work, *z*-scores are therefore used solely for normalization, not for interpreting probabilities.

## Supplementary Material









## Data Availability

The HTE data set containing 66,000 reactions is available as part of the github repository. An implementation of our method, as well as a web app visualizing our published data set using python and plotly dash are available at: https://github.com/georg-wuitschik-Roche/zScore-App. A hosted version of the web app can be found here: https://go.roche.com/zScoreApp.

## Notes

Plate design details for the Arylation of 2,5-dimethylpyrrole with 3-bromoisonicotinaldehyde (XLSX)
Plate design details for the arylation of 3,3-dimethylmorpholine with 3-bromo-2-methylpyridine (XLSX)
General information, data set information, results of comparison experiments, visualizations for other reaction types, limitations on statistical significance testing, underlying distribution histograms, many supporting figures of various data (PDF)
Transparent Peer Review report available (PDF)

## Notes

We respectfully dedicate this paper to Professor Steven V. Ley in honor of his 80th birthday.
