# Supplementary material for: Which Reaction Conditions Work on Drug-Like Molecules? Lessons from 66,000 High-Throughput Experiments
Source: ACS Cent Sci. 2026 Feb 5;12(2):222–32. doi: 10.1021/acscentsci.5c02031 (PMC12947288; doi:10.1021/acscentsci.5c02031)
Supplement: Supplementary file 3 [file oc5c02031_si_003.pdf]

Supporting Information

Which Reaction Conditions Work on Drug-Like Molecules? Lessons from 66,000 High-Throughput Experiments

Jesse Ahlbrecht[1, 2], Marius D.R. Lutz[2], Vera Jost[2], Michael Färber[3], Stefan Bräse[1,*], and Georg Wuitschik[2,*]

[1] Institute of Biological and Chemical Systems, Karlsruhe Institute of Technology (KIT), 76131 Karlsruhe, Germany
[2] Roche Pharma Research and Early Development, Roche Innovation Center Basel, F. Hoffmann-La Roche Ltd., 4070 Basel, Switzerland
[3] AI Center ScaDS.AI Dresden/Leipzig, University of Technology Dresden (TUD), 01069 Dresden, Germany

* Corresponding authors: stefan.braese@kit.edu; georg.wuitschik@roche.com

Table of Contents

General Information S2
Results of Comparison Experiments S3
Results for Other Reaction Types and Reagents S21
Limitations on Statistical Significance Testing S62
Dataset Information per Reaction Type S62
Underlying Distribution Histograms S71
References S75

General Information

Experiments were conducted in a glovebox under an inert atmosphere as described in our publication on HTE OS[1]:

Pd-Ligands in the Dataset

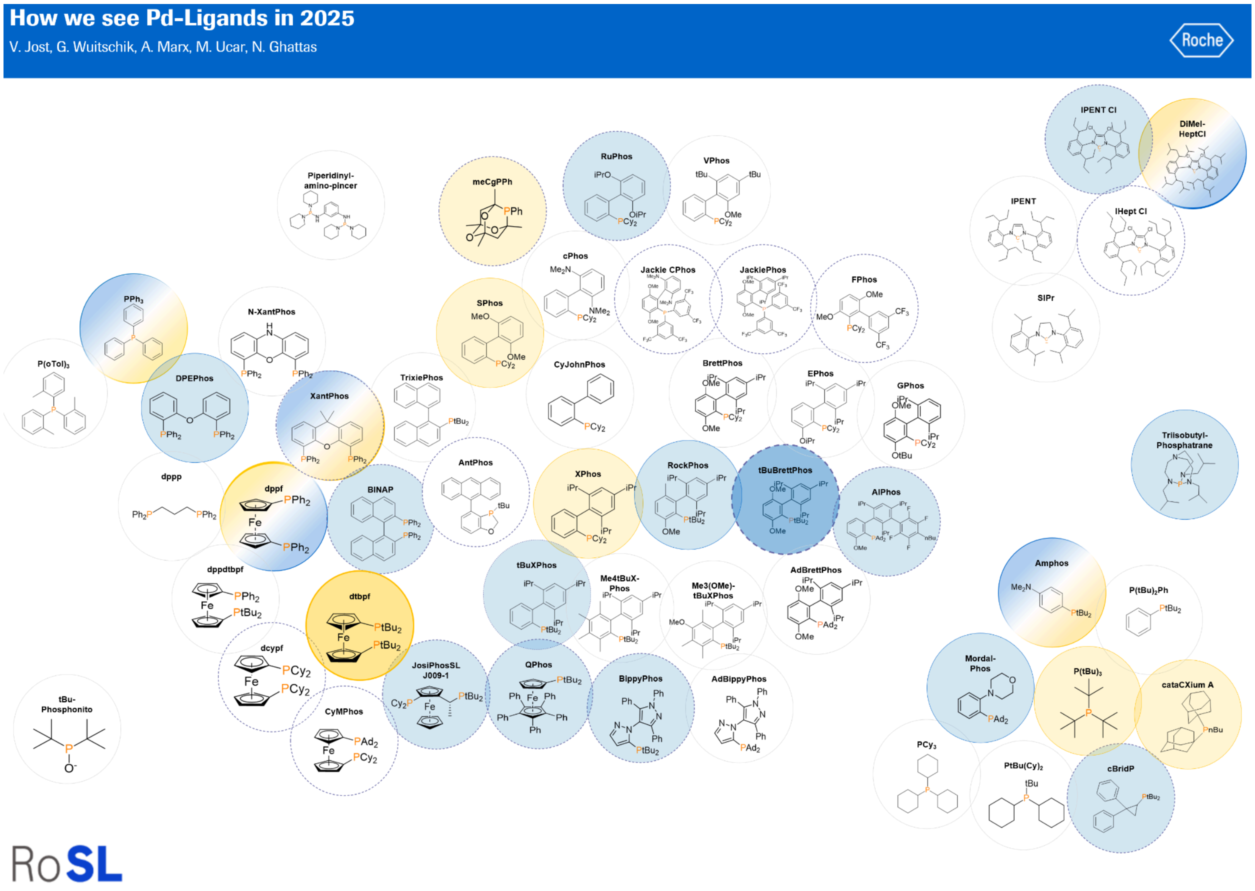

Figure S1: Pd-Ligands employed by us in the form of precatalysts. The arrangement in the 2D-plane is based on structural similarity and observation of similar behavior in coupling reactions. Blue shading indicates ligands we found to perform consistently well in Buchwald-Hartwig reactions. Yellow shading indicates ligands we found to perform consistently well in Suzuki-Miyaura couplings.

Descriptors of the underlying dataset

Due to necessary confidentiality concerns and intellectual property (IP) protection, we must maintain the anonymity of the synthesized structures. Instead, we describe the ensemble of synthesized products by 11 descriptors. These include measures of drug-likeness (clogP,

fraction C(sp3), total polar surface area (PTSA), number of hydrogen bonding acceptors/donors), as well as counts of rings, heteroatoms and heavy atoms.

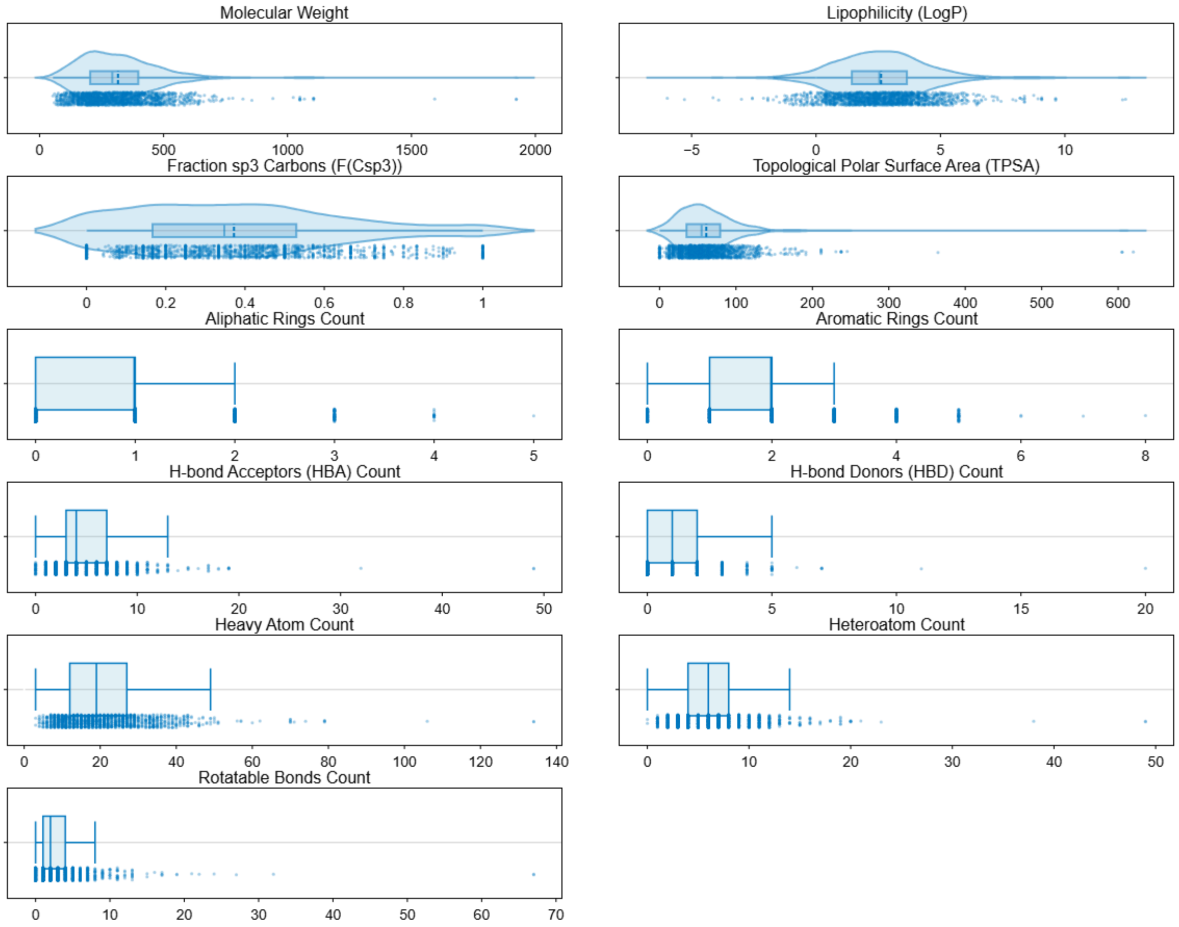

Figure S2: Boxplots of 11 descriptors of the synthesized products in this dataset. Descriptors were calculated with rdkit version 2024_03_3.[2]

Results of Comparison Experiments

To benchmark the insights of our dataset compared to literature data and machine learning models, we conducted two reactions using plate designs derived from three inspiration sources. Two Buchwald-Hartwig reactions were selected based on their anticipated high difficulty and availability of the starting materials in house the company stockroom and without prior knowledge on the reaction outcomes.

All results we obtained are presented and we did not run any experiments on other transformations. Design of the plates based on literature-precedent was performed by a different person and without seeing the designs created using the z-score app or the Buchwald-Hartwig AI model released by the Denmark group.[3] Following our standard procedure,[1] two plates were solid-dosed in a nitrogen-filled glovebox, sealed, transferred to another glovebox where liquids

were added, heating/stirring was performed as well as sampling. Information related to the reaction execution can be found in the cheatsheets for these reactions generated by HTE OS that are part of the supporting information.

For the arylation of 3,3-dimethylmorpholine with 3-bromo-2-methylpyridinehe the z-score app predicted the chlorocarbene catalysts that show at least some activity in this transformation. As these are not part of the scope of the AI prediction model and also not picked up during Scifinder search, neither of these plates showed product.

For the arylation of 2,5-dimethylpyrrole with 3-bromoisonicotinaldehyde, none of the Pd-catalyzed reaction conditions resulted in product. Product mass was observed in some of the Cu-catalyzed conditions on the plate designed using Scifinder, but the associated peaks in UV were too minute for us to be comfortable to assign them to be product.

Arylation of 3,3-dimethylmorpholine with 3-bromo-2-methylpyridine

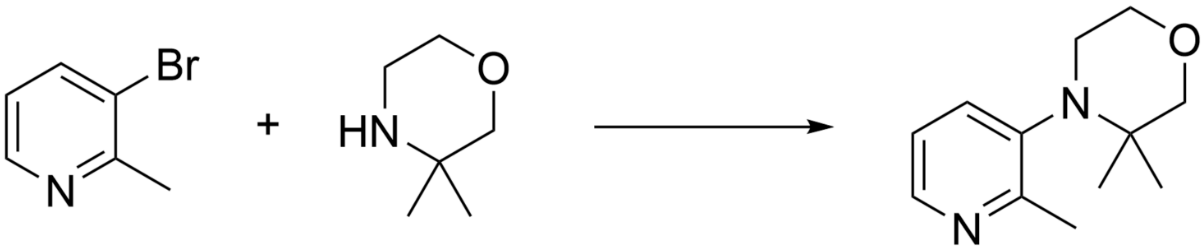

Equation S1: Arylation of 3,3-dimethylmorpholine with 3-bromo-2-methylpyridine

Design through the z-Score App

In order to obtain the design for this plate, catalysts, bases and solvents were retrieved separately for Buchwald-Hartwig reactions of α-branched secondary amines with aryl bromides and chlorides. Because there are only 9 transformations in the dataset for this combination of reaction type and reacting functional groups, the parameter minimum number of ELNs in the options section of the app was set to one. The top six catalysts were selected and amended by two catalysts further down the list that differ structurally significantly from the others. For solvents (minimum number of ELNs = 5) and bases (minimum number of ELNs = 4), the top four entries were chosen and 12 chosen from all permutations based on chemical compatibility and diversity. The resulting plate design as well as the reaction outcomes are shown in Figures S3 to S5.

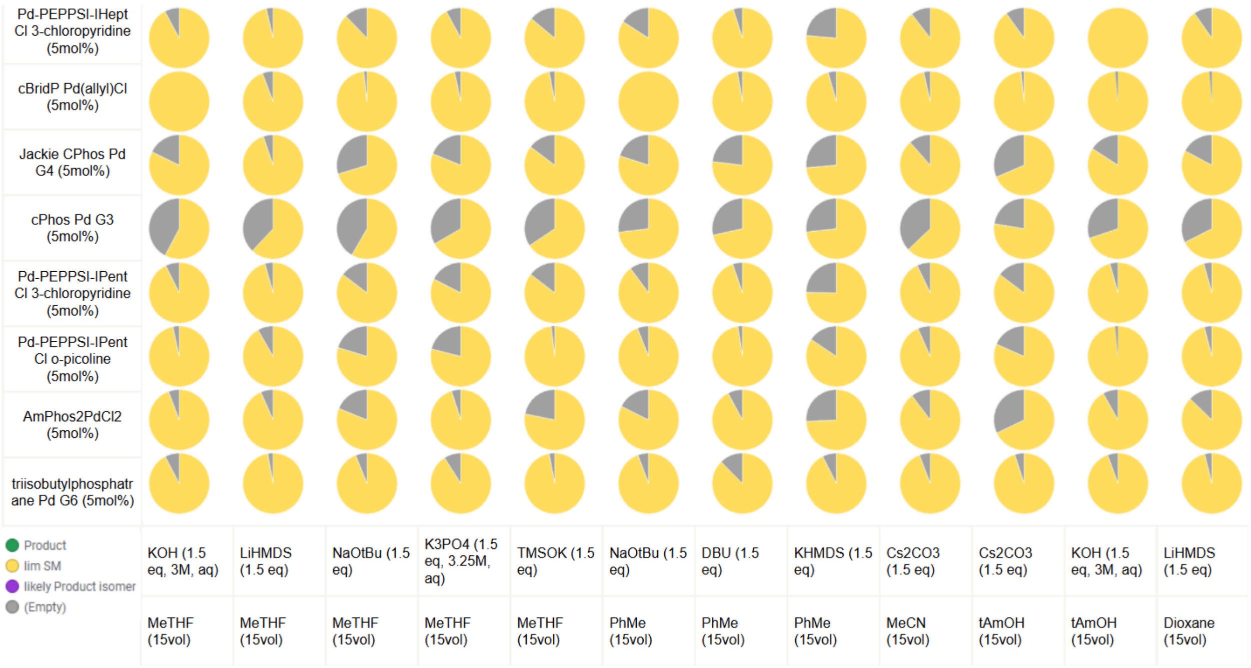

Figure S3: Results of z-score plate, Sample 1 taken after stirring at 2 h, 80 °C.

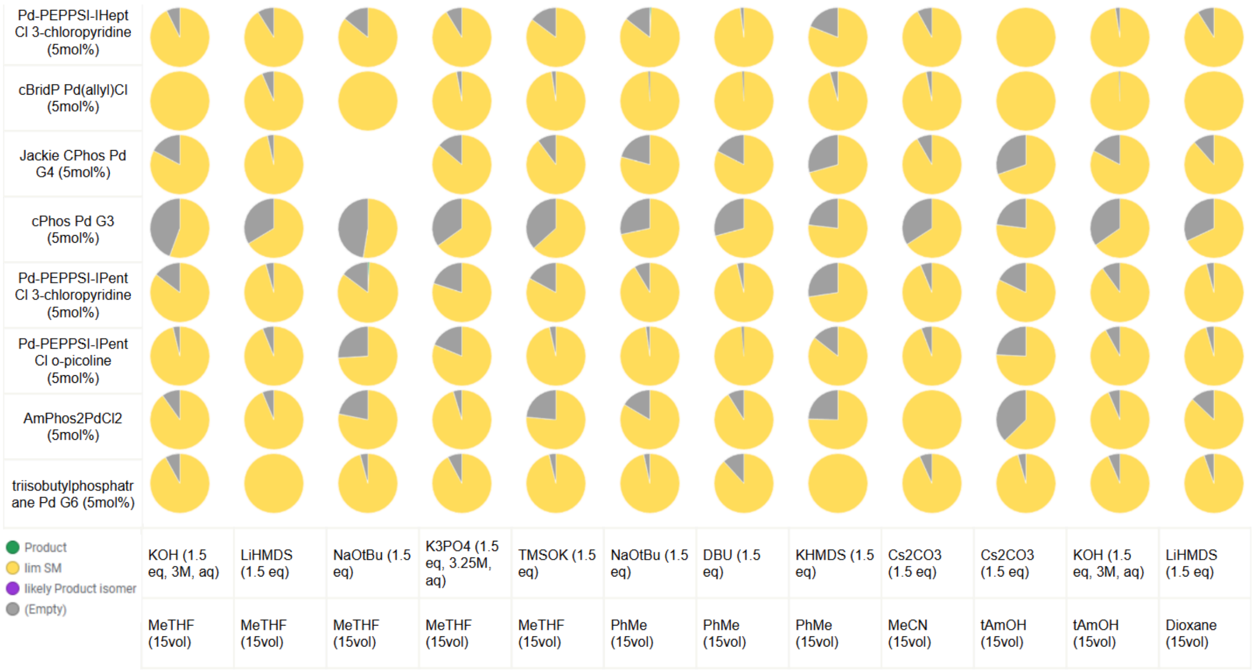

Figure S4: Results of z-score plate, Sample 2 taken after stirring at 4 h, 80 °C.

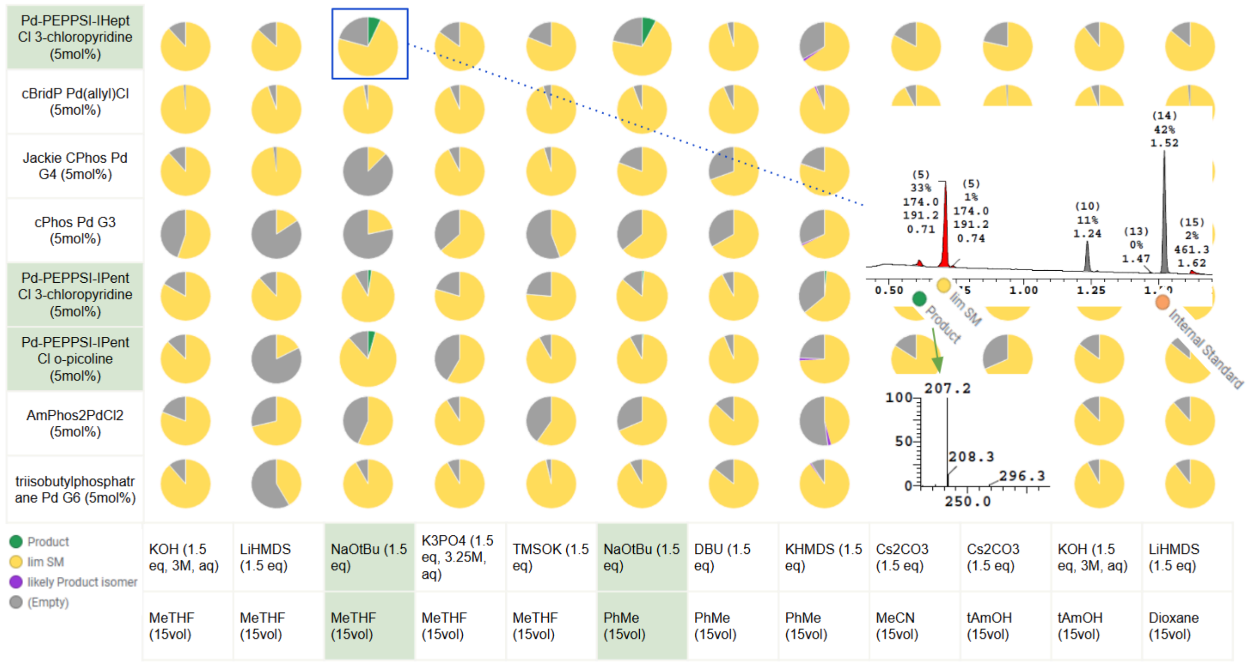

Figure S5: Results of z-score plate, Sample 3 taken after stirring at 21 h, 80 °C. We highlighted the LC-MS of vial A3 to give an impression of the size of the product peak and the cleanliness of the reaction. Also visible are the small amounts of what we believe to be product isomer formed in column 9 (KHMDS/PhMe).

We observe two distinct groups of peaks with product mass, one formed in the presence of chlorocarbene catalysts and NaO^{t}Bu and one group in the presence of KHMDS in toluene. The peaks of the latter are only barely visible in Figure S5 and are consistent with respect to retention time to the peaks observed on the AI plate under the same conditions. The former group of peaks was assigned to the product, as it has a retention time distinct from the benzyne product, it is associated with the expected mass and is formed under a set of related conditions. Also, we could not conceive other product isomers that could be formed under these reaction conditions. Although we have not isolated the product and characterized it, we believe the peak is real and the result a good starting point for further optimization.

Design through Scifinder research

The design of the literature-driven plate was based on Scifinder reaction query (Equation S2), searching for C-N disconnections of the indicated substructure, which afforded 11k hits. The results were manually screened and transition-metal free reactions (e.g. nucleophilic aromatic substitution) were excluded. A 96 well plate was designed based on the prevalent catalysts, bases and solvents.

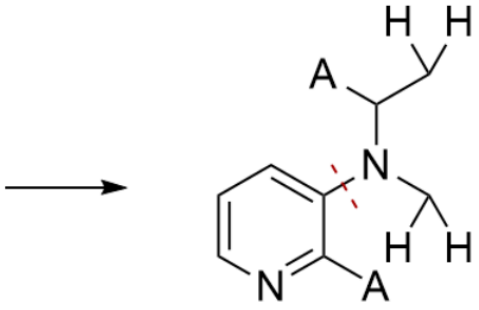

Equation S2: Scifinder query used to find reaction conditions.

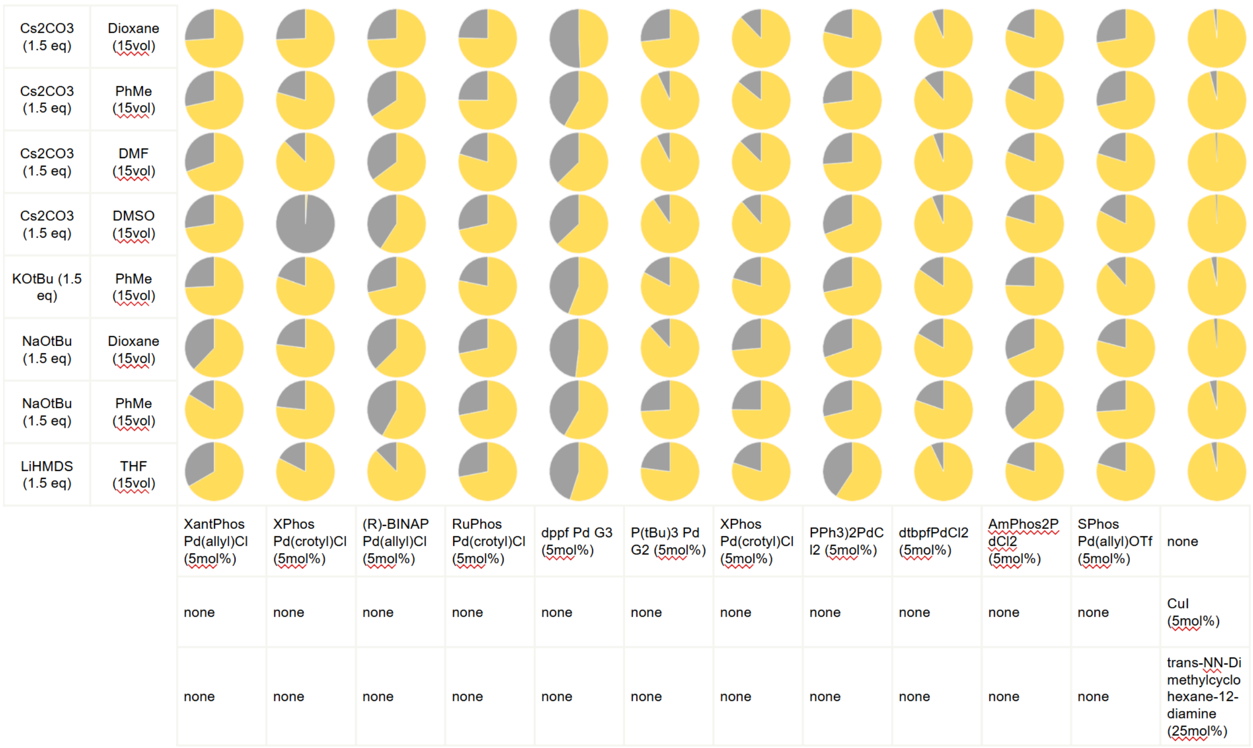

Figure S6: Results of Scifinder plate, Sample 1 taken after stirring at 2 h, 80 °C.

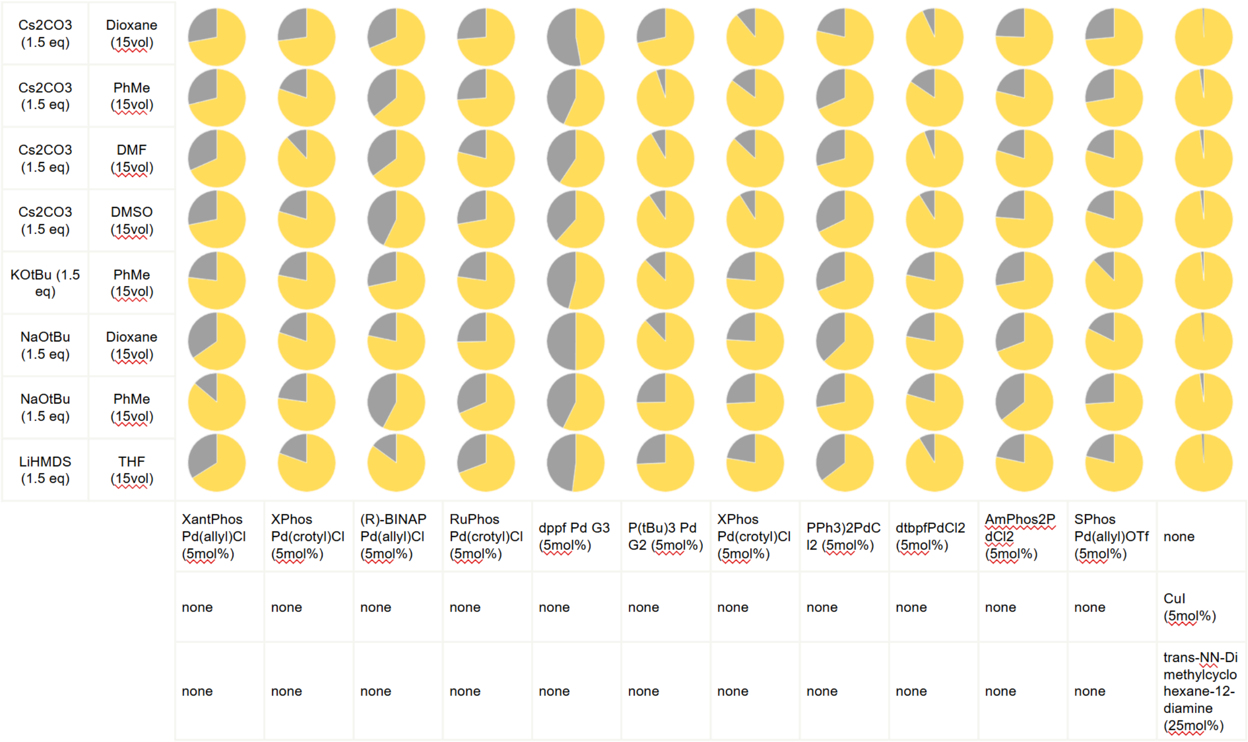

Figure S7: Results of Scifinder plate, Sample 2 taken after stirring at 4 h, 80 °C.

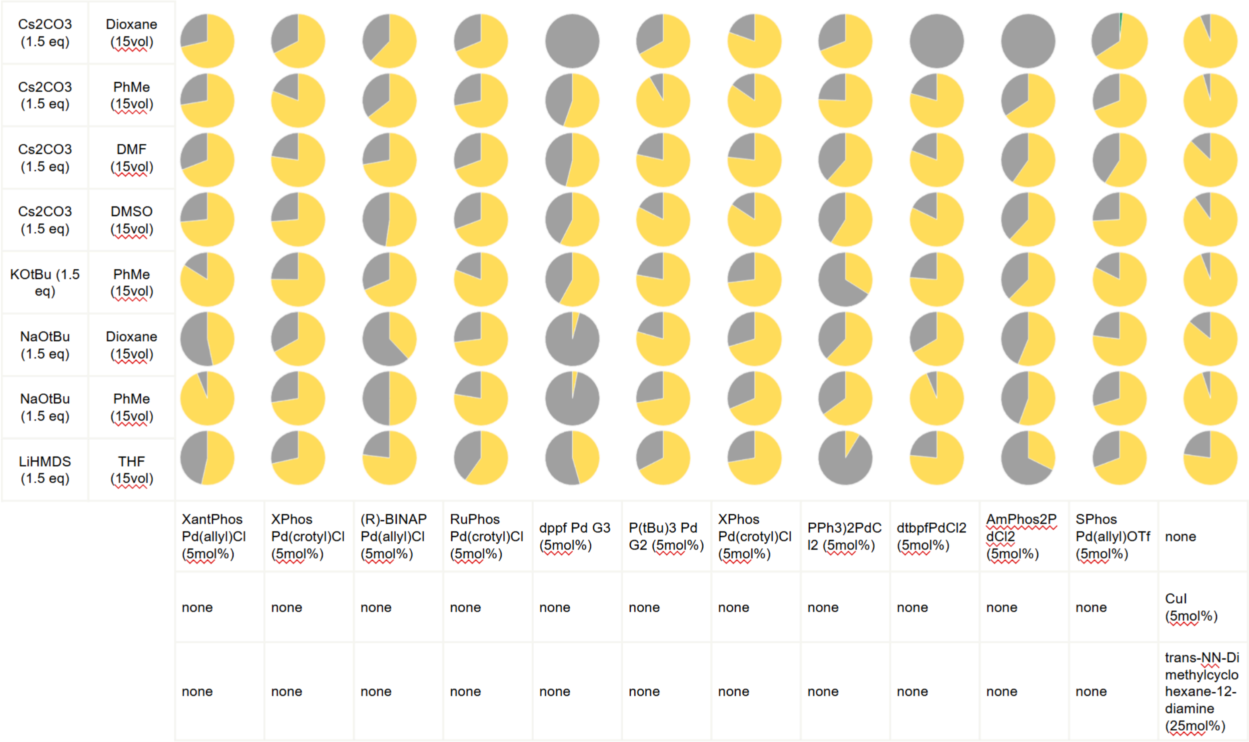

Figure S8: Results of Scifinder plate, Sample 3 taken after stirring at 19 h, 80 °C.

Design through AI Prediction

The prediction of the reaction outcomes was conducted using the published version of the model published by Denmark et al.[3] The results are shown in Figure S9.

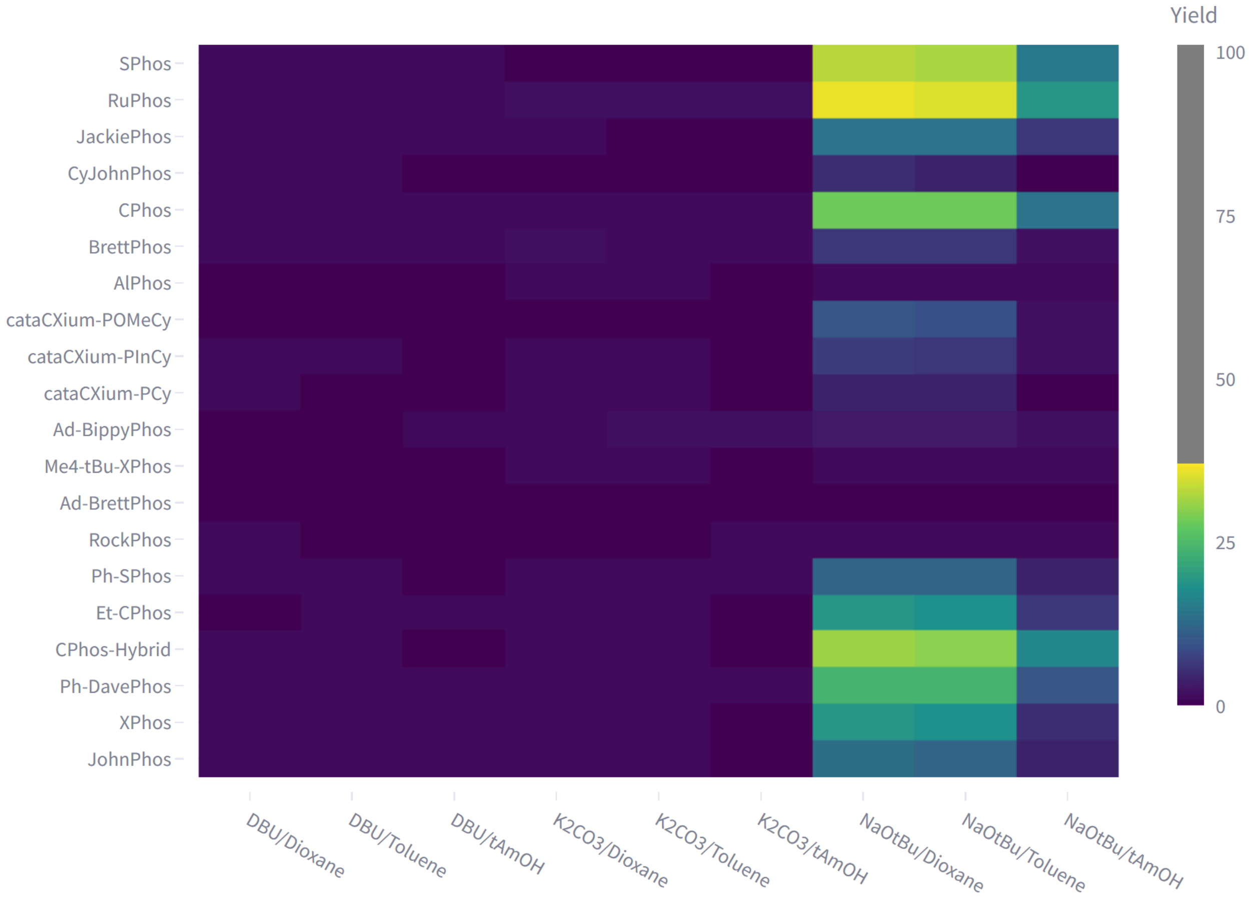

Figure S9: AI prediction for the arylation of 3,3-dimethylmorpholine with 3-bromo-2-methylpyridine.

Based on this prediction, we arrived at the following plate layout (Figure S10). As the number of reagent combinations predicted to result in product was smaller than 96, we filled the plate with similar solvents and bases as well as with catalysts predicted to have small or no activity. Et-CPhos was omitted, since we do not have a Pd-precatalyst of it available.

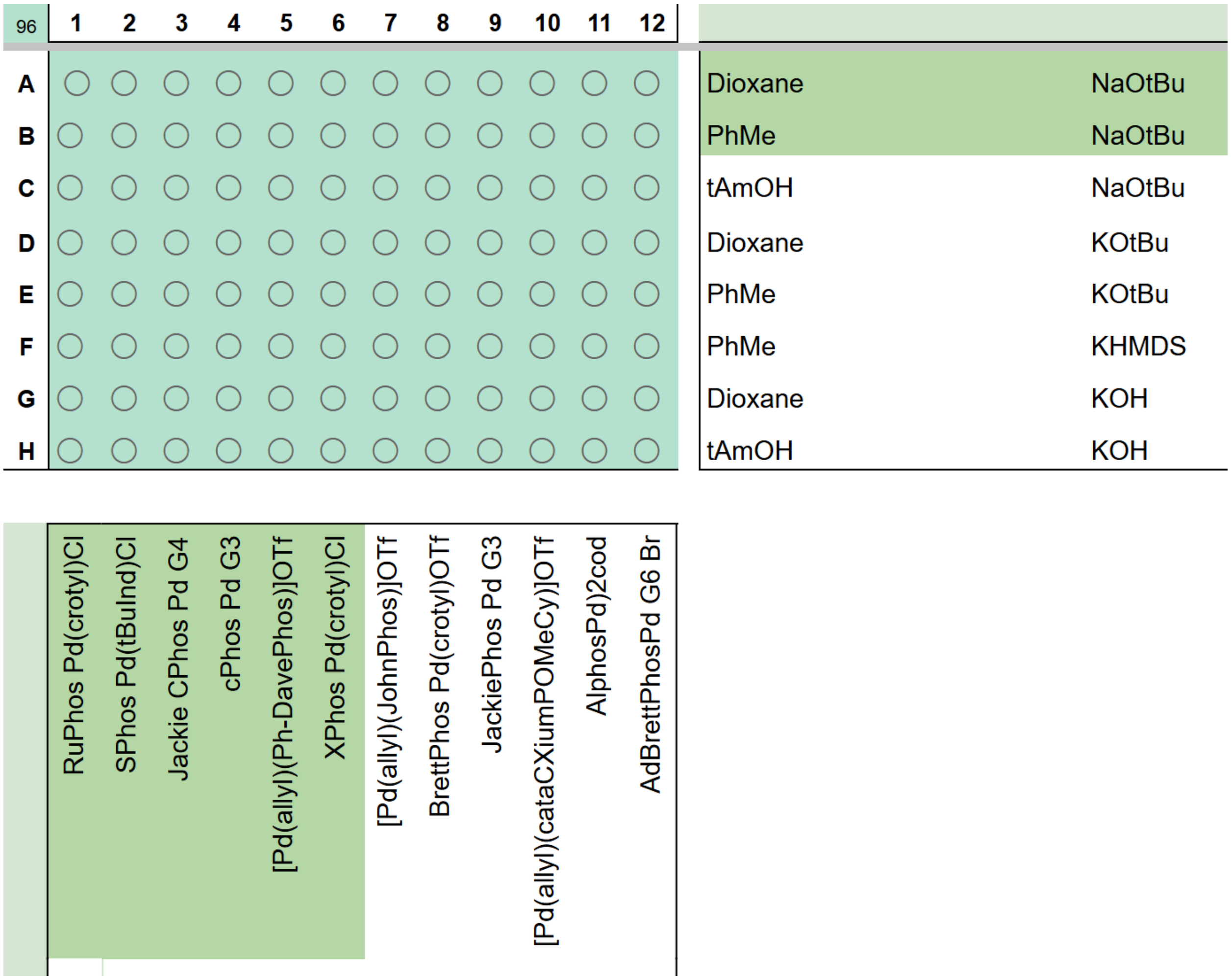

Figure S10: Plate designed for the arylation of 3,3-dimethylmorpholine with 3-bromo-2-methylpyridine based on AI prediction. Reagents predicted to lead to product conversion highlighted in green.

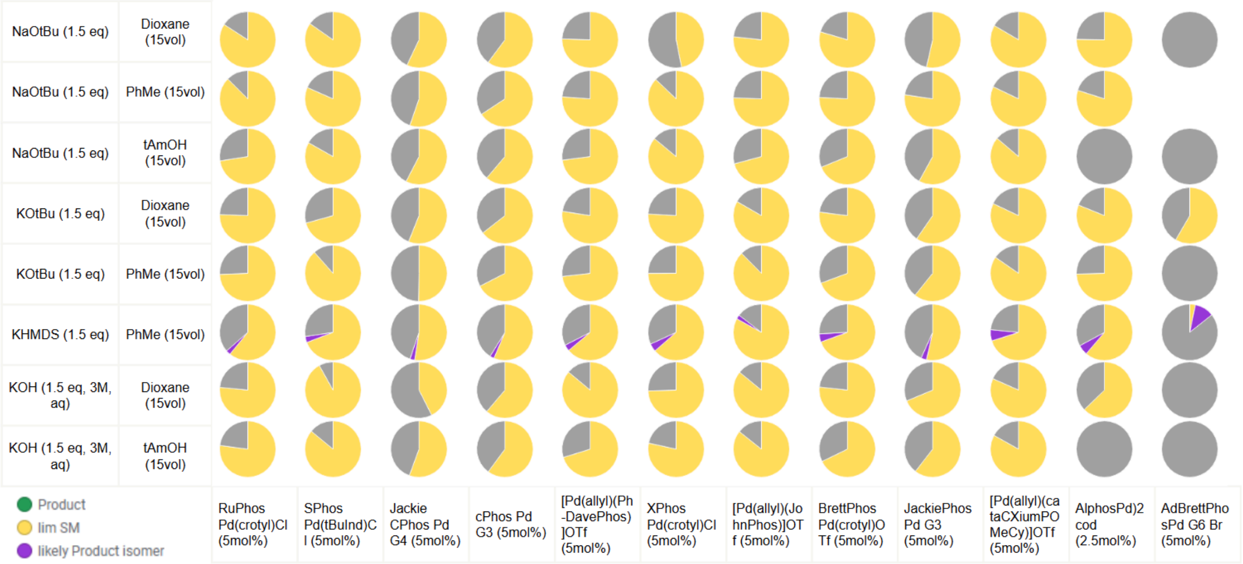

Figure S11: Results of the AI prediction plate, Sample 1 taken after stirring at 2 h, 80 °C. Missing pies observed in this plate indicate problems during LCMS measurement that lead to data loss.

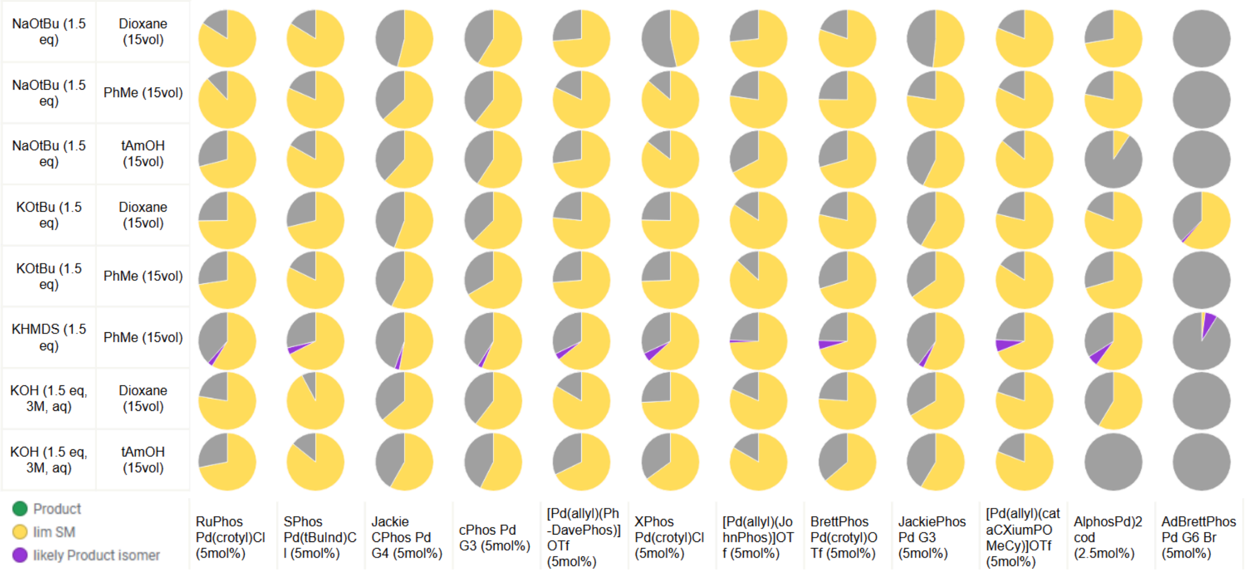

Figure S12: Results of the AI prediction plate, Sample 2 taken after stirring at 5 h, 80 °C.

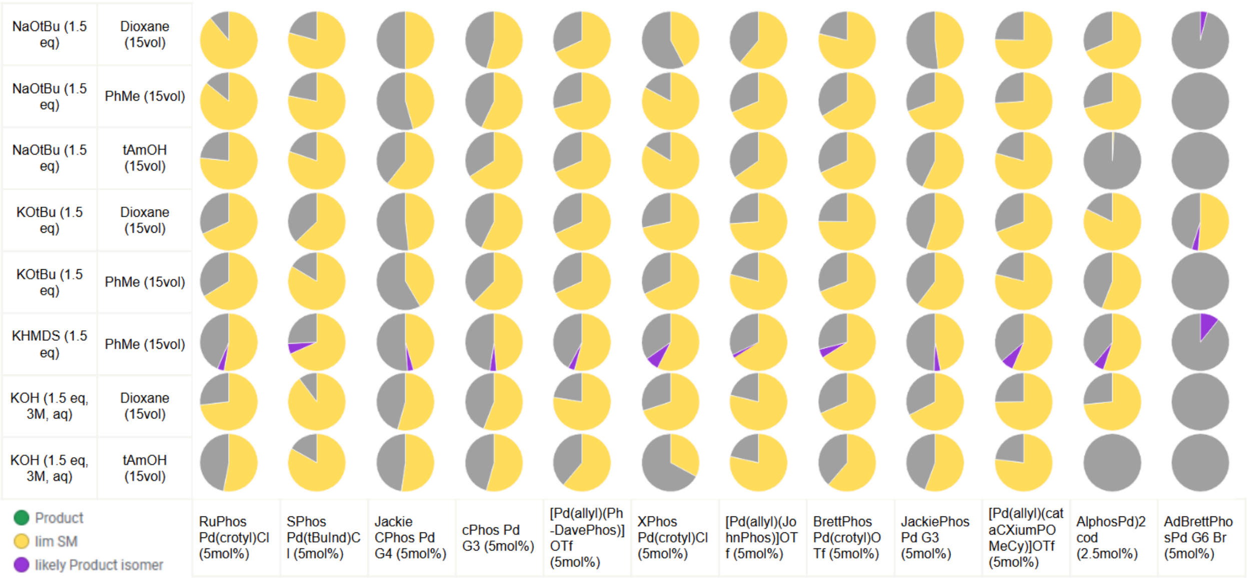

Figure S13: Results of the AI prediction plate, Sample 3 taken after stirring at 22 h, 80 °C.

The results shown in Figure S11 to S13 indicate that no product is formed under any of these conditions. We do observe what we believe to be the formation of the 4-pyridyl isomer in the presence of KHMDS/PhMe, a peak with a retention time distinct from what we believe to be the product that we observed in the z-score plate. As the formation of the 4-pyridyl isomer is proposed to proceed through a benzyne mechanism (Equation S3), its presence irrespective of catalyst would be explained.

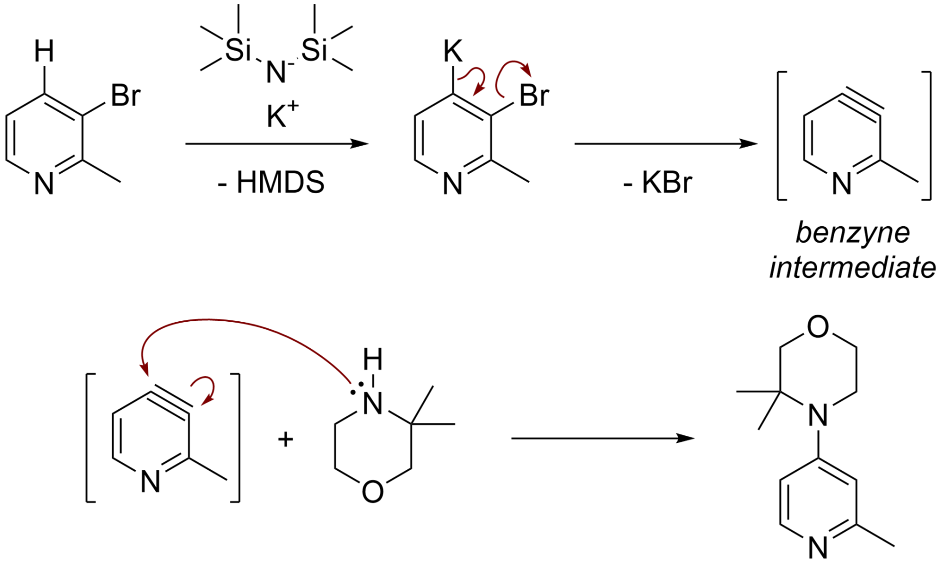

Equation S3: Proposed mechanism for the base-promoted formation of the 4-arylated isomer via a benzyne intermediate.

Arylation of 2,5-dimethylpyrrole with 3-bromoisonicotinaldehyde

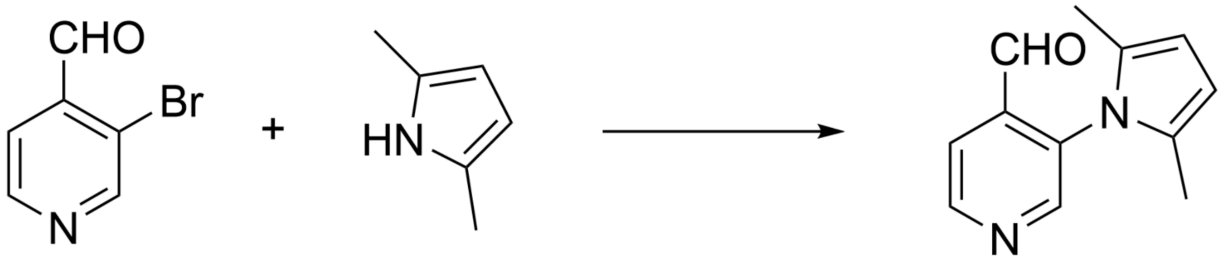

Equation S4: Arylation of 2,5-dimethylpyrrole with 3-bromoisonicotinaldehyde.

Design through the z-Score App

In order to obtain the design for this plate, catalysts, bases and solvents were retrieved separately for Buchwald-Hartwig reactions of aromatic NH-groups with aryl halides. We selected catalysts based on the top ligands as well as appropriate combinations of the top bases and solvents.

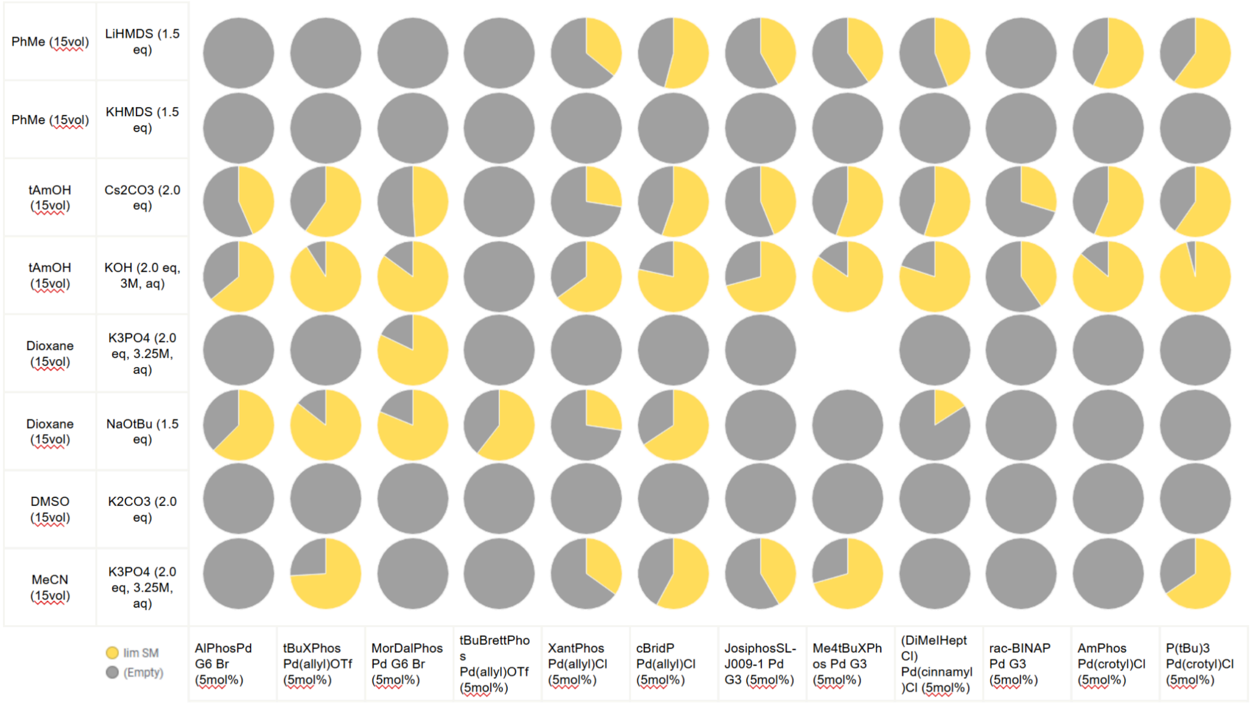

Figure S14: Results of z-score plate, Sample 1 taken after stirring at 3 h, 80 °C. Missing pies observed in this plate indicate problems during LCMS measurement that lead to data loss.

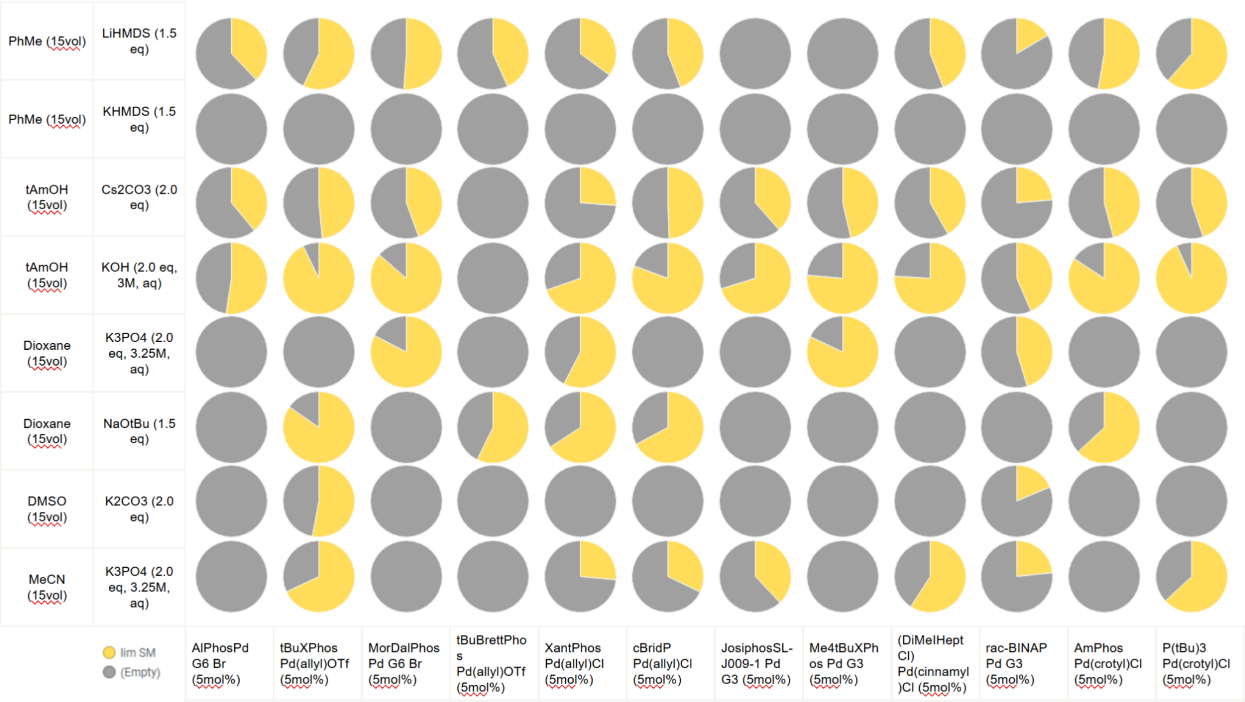

Figure S15: Results of z-score plate, Sample 2 taken after stirring at 5 h, 80 °C.

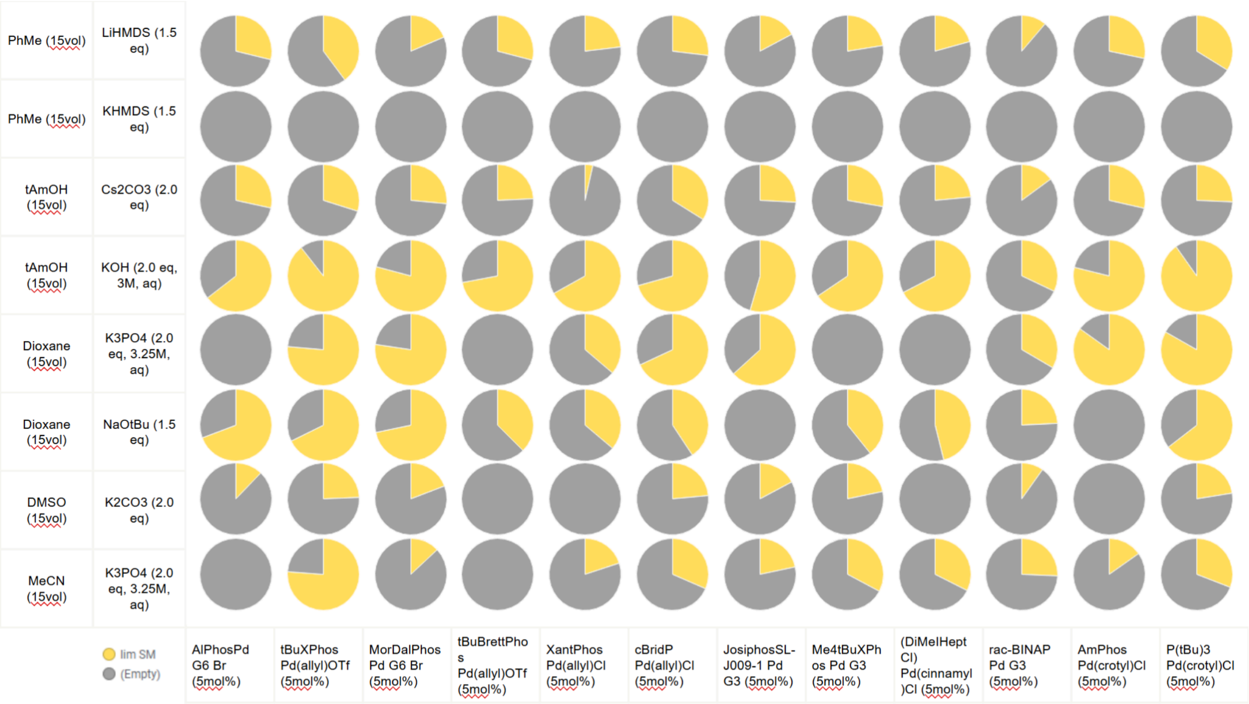

Figure S16: Results of z-score plate, Sample 3 taken after stirring at 19 h, 80 °C.

The results shown in Figure S14 to S16 indicate that no product is formed under any of these conditions.

Design through Scifinder research

The design of the literature-driven plate was based on Scifinder reaction query (Equation S5), searching for C-N disconnections of the indicated substructure, which afforded 28k hits. The results were manually screened and transition-metal free reactions (e.g. pyrrole ring formation) were excluded. A 96 well plate was designed based on the prevalent catalysts, bases and solvents.

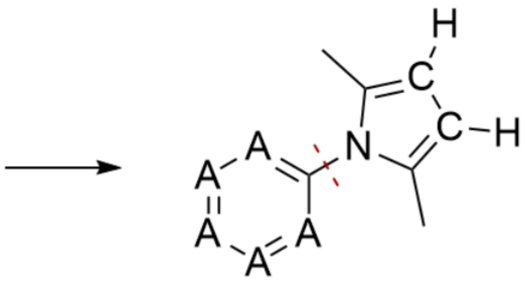

Equation S5: Scifinder query used to find reaction conditions.

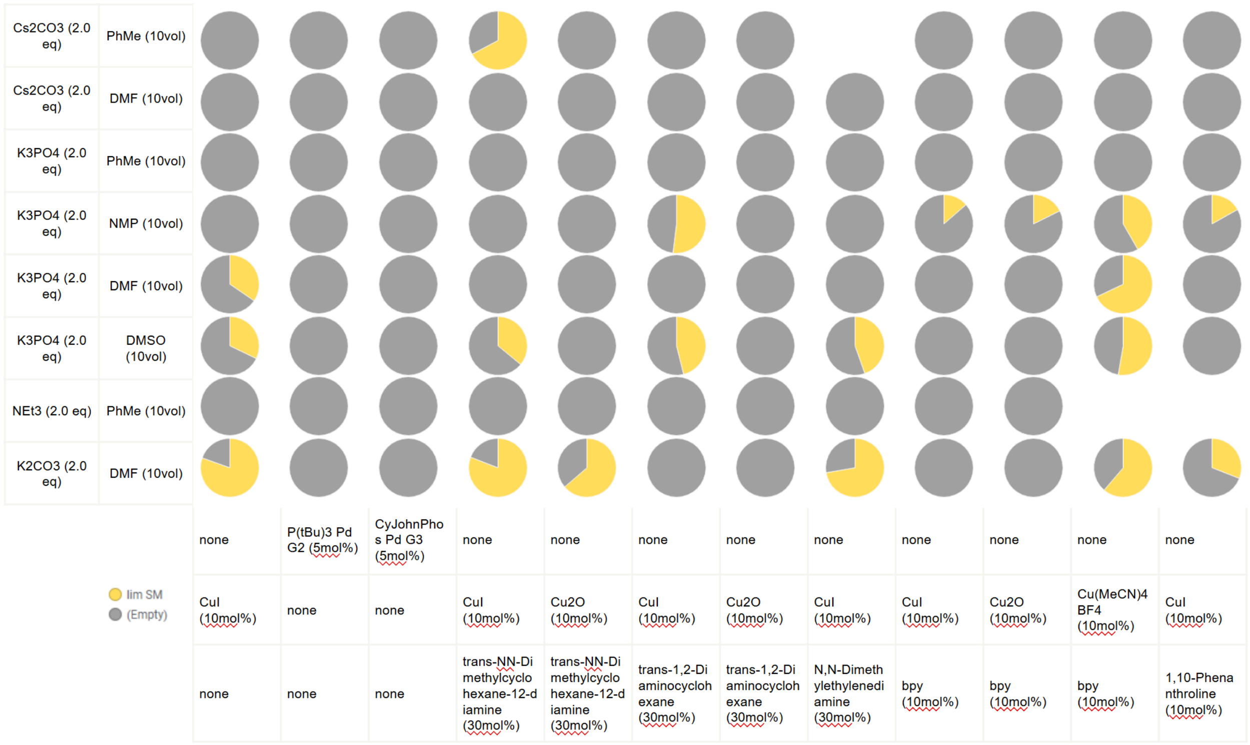

Figure S17: Results of Scifinder plate, Sample 1 taken after stirring at 3 h, 110 °C. Missing pies observed in this plate indicate problems during LCMS measurement that lead to data loss.

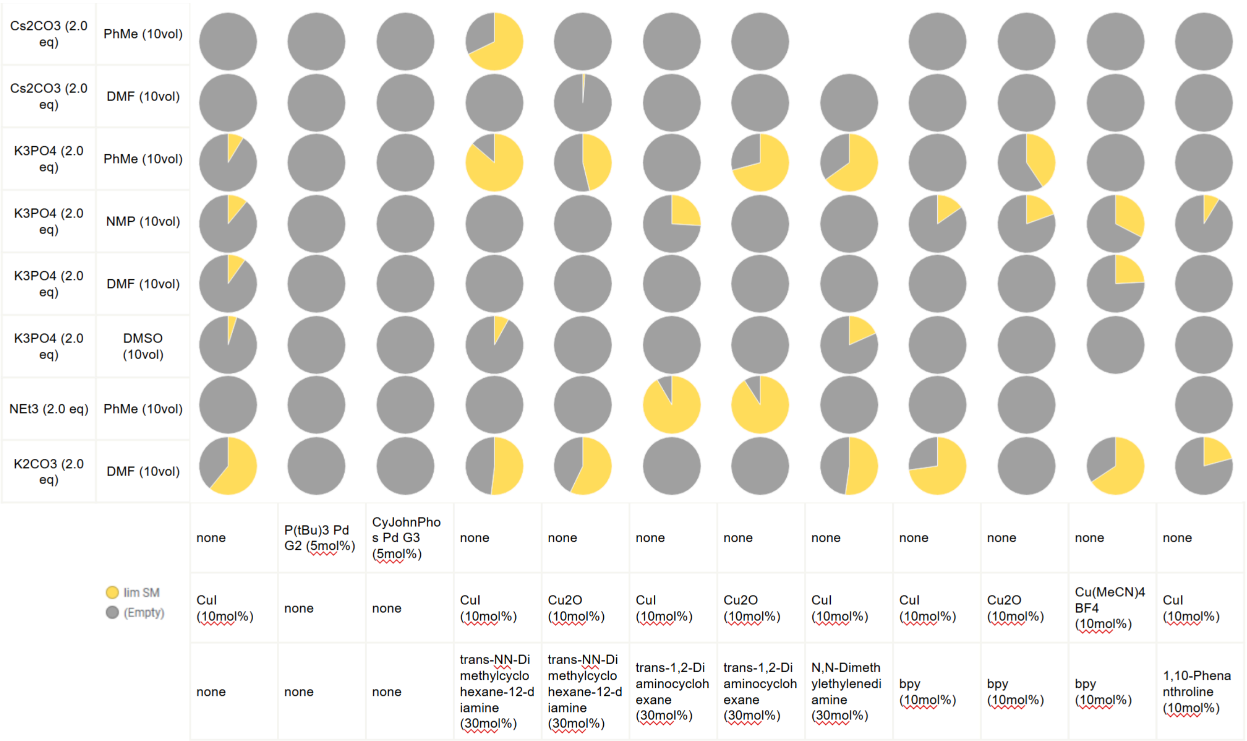

Figure S18: Results of Scifinder plate, Sample 2 taken after stirring at 7 h, 110 °C. Missing pies observed in this plate indicate problems during LCMS measurement that lead to data loss.

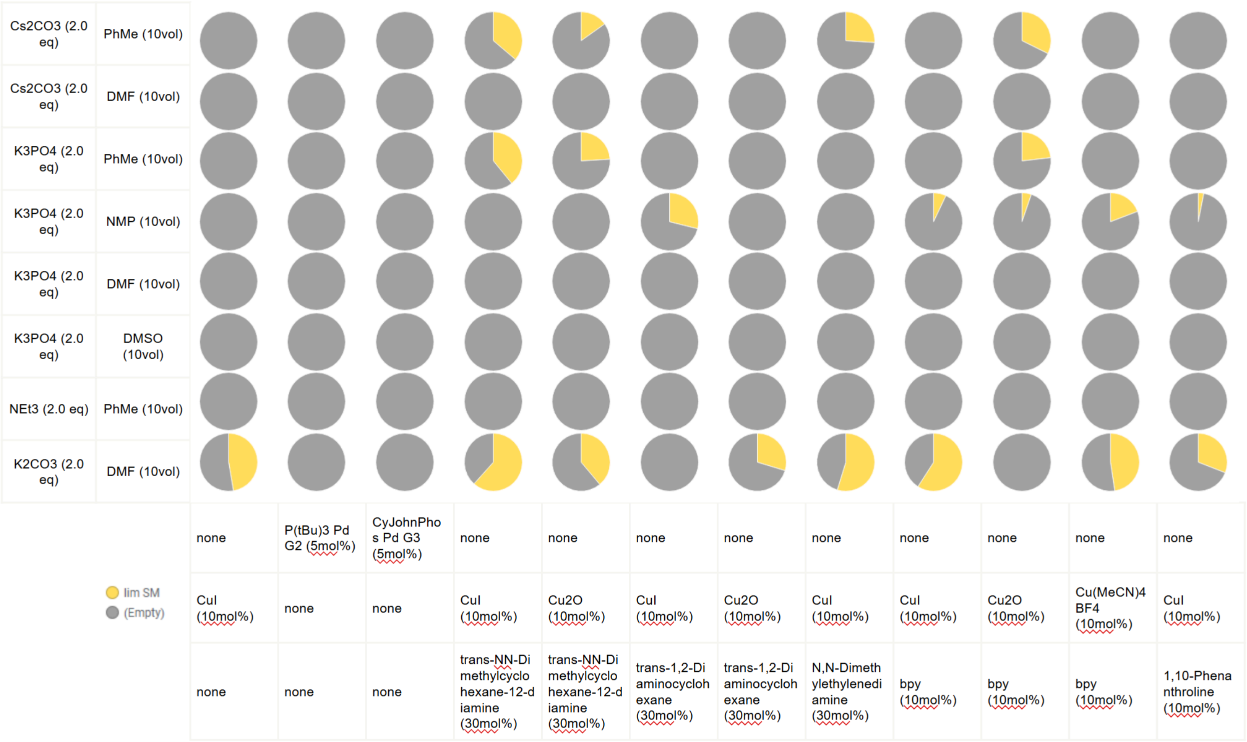

Figure S19: Results of Scifinder plate, Sample 3 taken after stirring at 23 h, 110 °C.

The results shown in Figure S17 to S19 indicate that no product is formed under any of these conditions. For the Cu-catalyzed conditions that were included on this plate because they were part of the search results, we observed what could be interpreted as traces of product, but the peaks were so small and inconsistent that we decided against tagging them as such.

Design through AI Prediction

The prediction of the reaction outcomes was conducted using the published version of the model published by Denmark et al.[3] The results are shown in Figure S20.

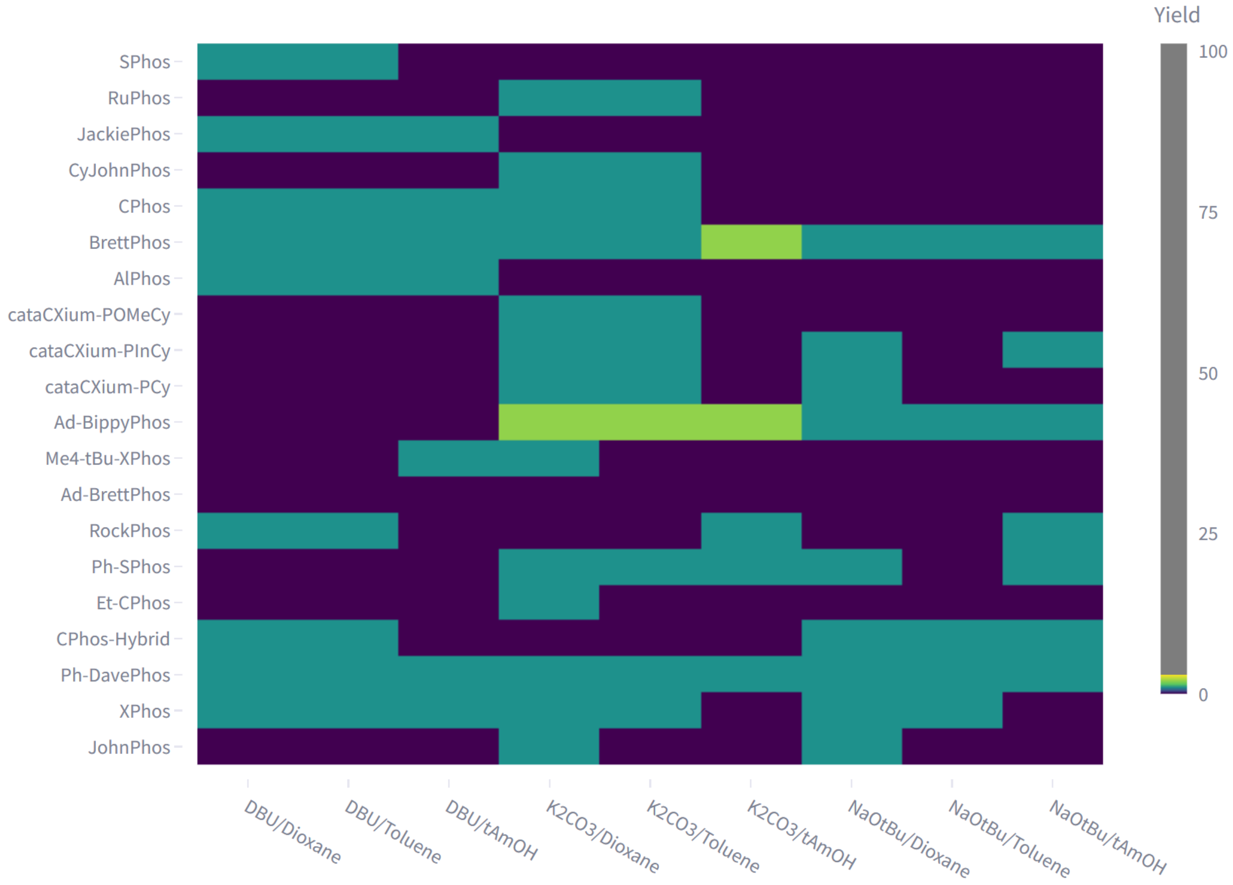

Figure S20: AI prediction for the arylation of 3,3-dimethylmorpholine with 3-bromo-2-methylpyridine.

Based on this prediction, we arrived at the following plate layout (Figure S21). As the model predicts little conversion for the whole condition space, we included conditions that were predicted to show at least some activity:

96	1	2	3	4	5	6	7	8	9	10	11	12		
A													K2CO3	tAmOH
B													NaOtBu	tAmOH
C													DBU	tAmOH
D													K2CO3	Dioxane
E													NaOtBu	Dioxane
F													DBU	Dioxane
G													NaOtBu	PhMe
H													DBU	PhMe
	BrettPhos Pd(crotyl)OTf	[Pd(allyl)(Ad-BippyPhos)]OTf	XPhos Pd(tBuInd)Cl	cPhos Pd G3	CyJohnPhos Pd G3	RuPhos Pd(crotyl)Cl	[Pd(allyl)(Ph-DavePhos)]OTf	[Pd(allyl)(Ph-SPhos)]OTf	RockPhos Pd G3	Jackie CPhos Pd G4	AlphosPd)2cod	JackiePhos Pd G3		

Figure S21: Plate designed for the arylation of 2,5-dimethylpyrrole with 3-bromoisonicotinaldehyde.

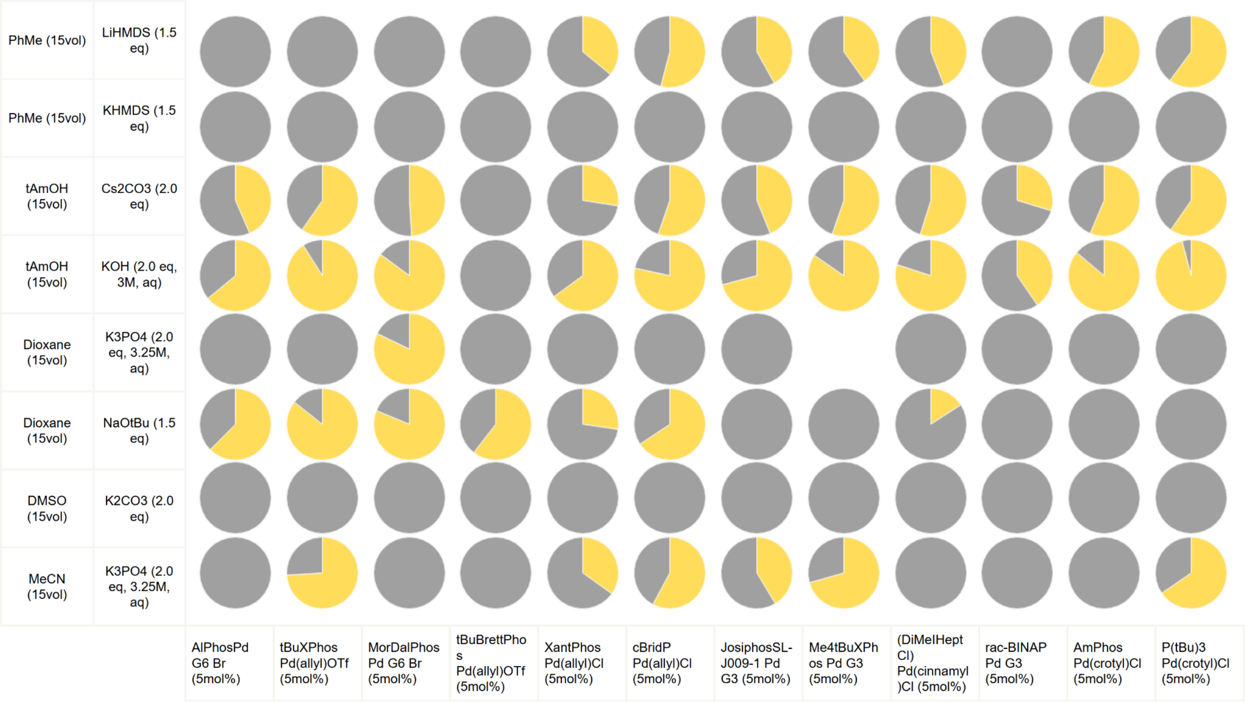

Figure S22: Results of the 2nd AI prediction plate, Sample 1 taken after stirring at 3 h, 80 °C. Missing pies observed in this plate indicate problems during LCMS measurement that lead to data loss.

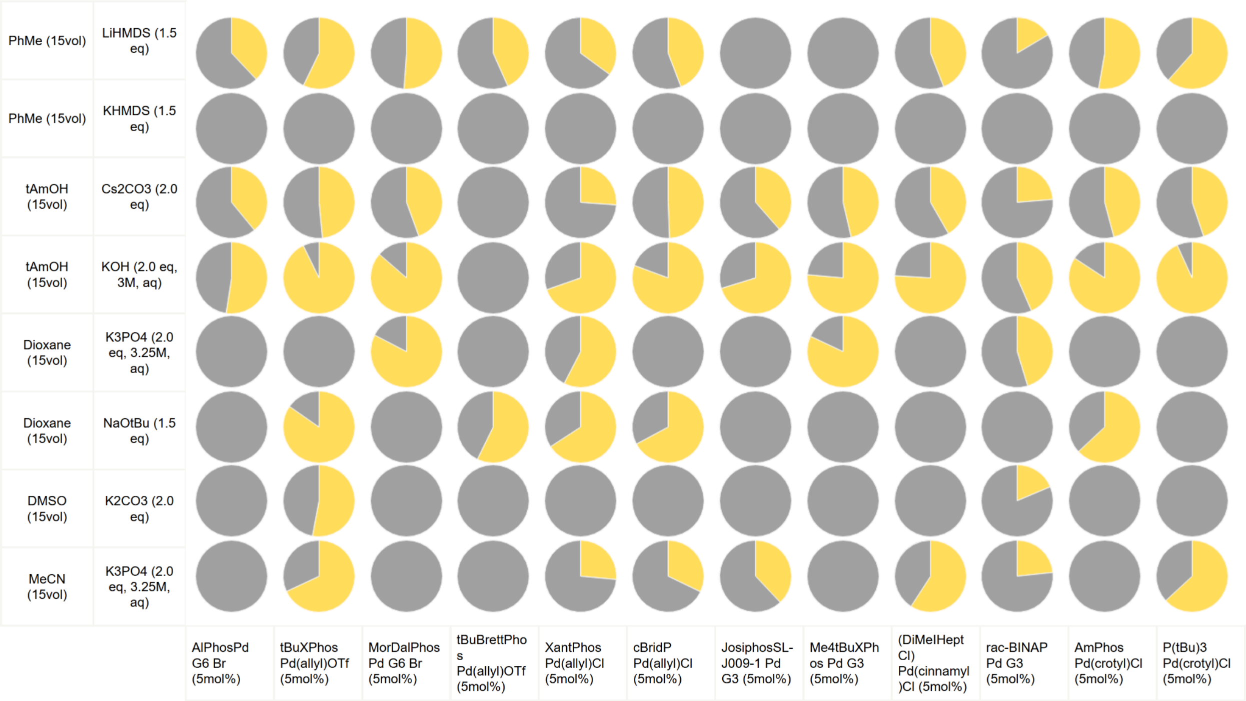

Figure S23: Results of the 2nd AI prediction plate, Sample 2 taken after stirring at 5 h, 80 °C.

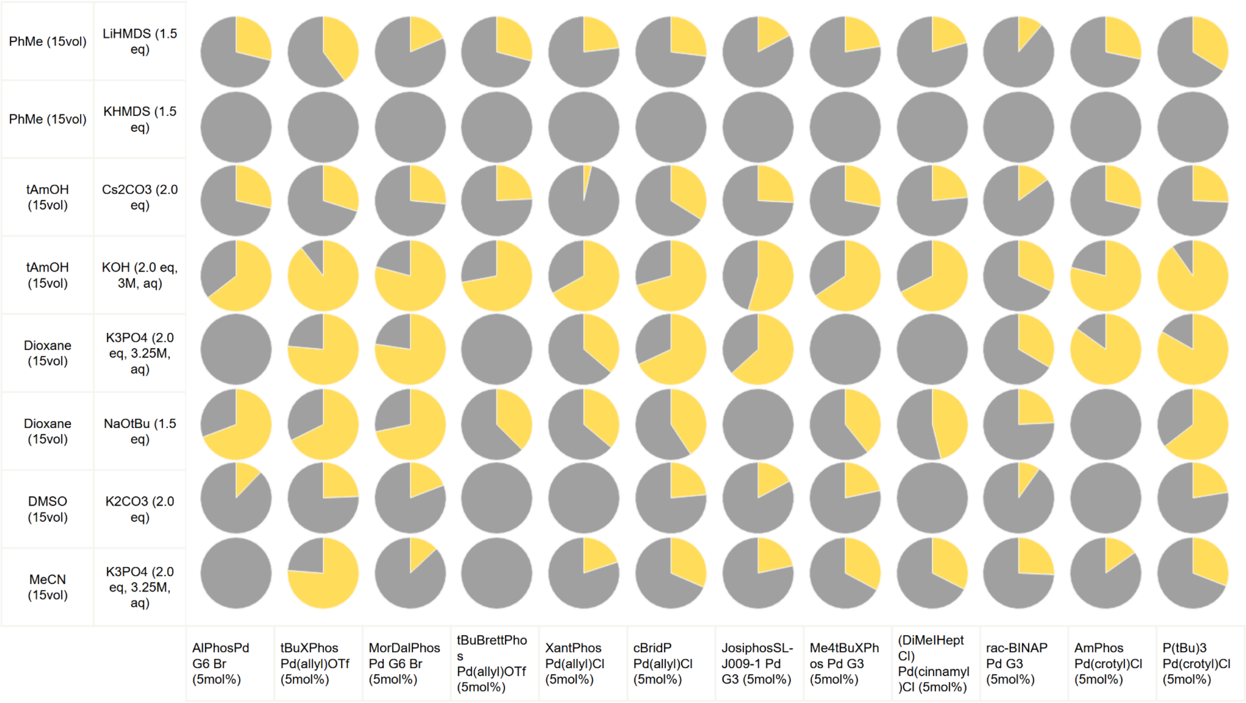

Figure S24: Results of the 2nd AI prediction plate, Sample 3 taken after stirring at 19 h, 80 °C.

The results shown in Figure S22 to S24 indicate that no product is formed under any of these conditions. This outcome is in good agreement with the predicted, low, yields.

Results for Other Reaction Types and Reagents

Below each boxplot we show all underlying datapoints. We use a color gradient to indicate the number of reactions, thereby highlighting ligands that are supported by a greater number of datapoints.

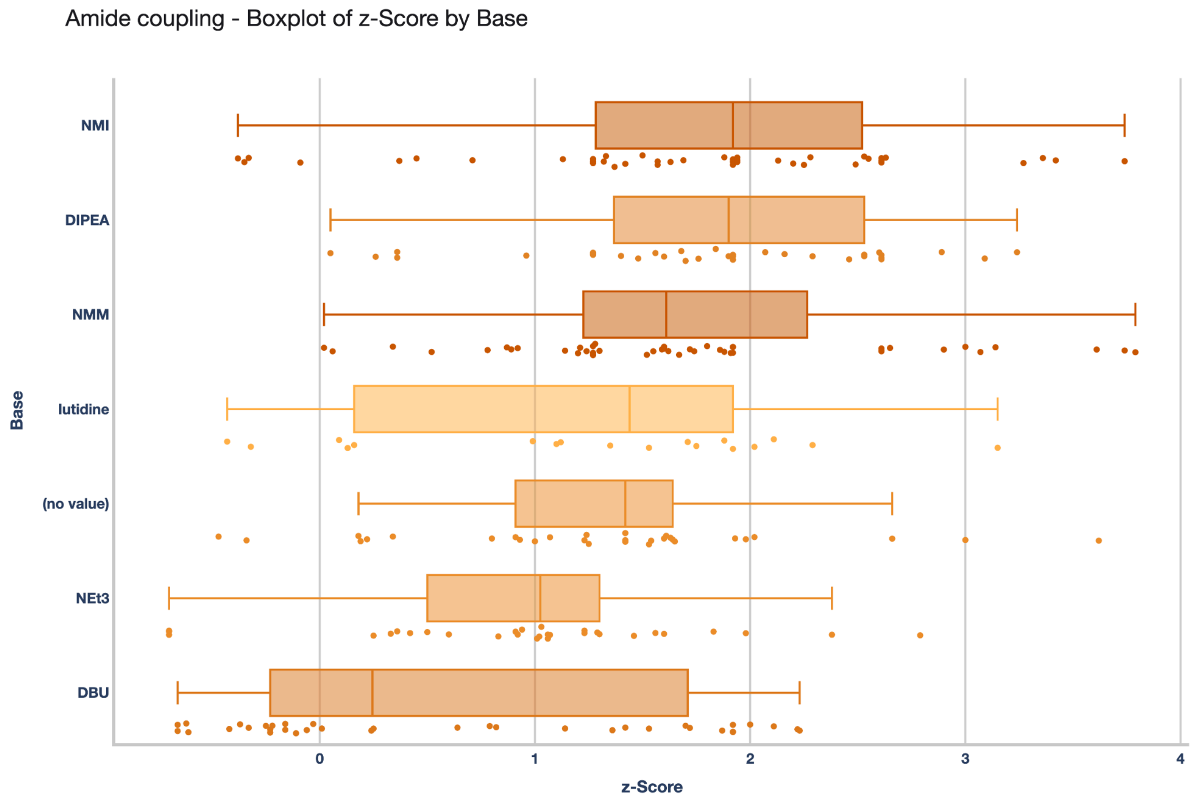

Figure S25: Base boxplot for Amide coupling reactions

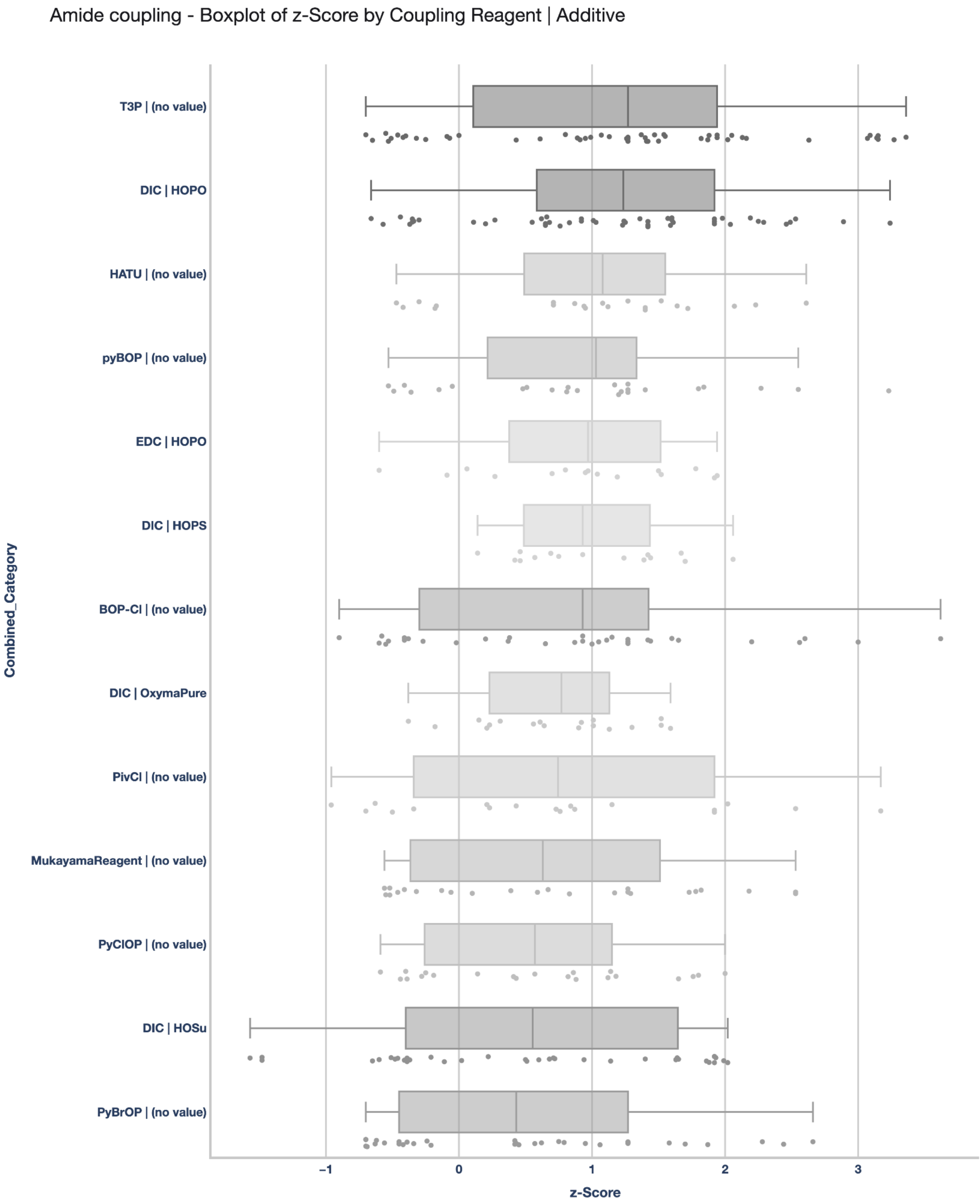

Figure S26: Coupling Reagent + Additive boxplot for Amide coupling reactions

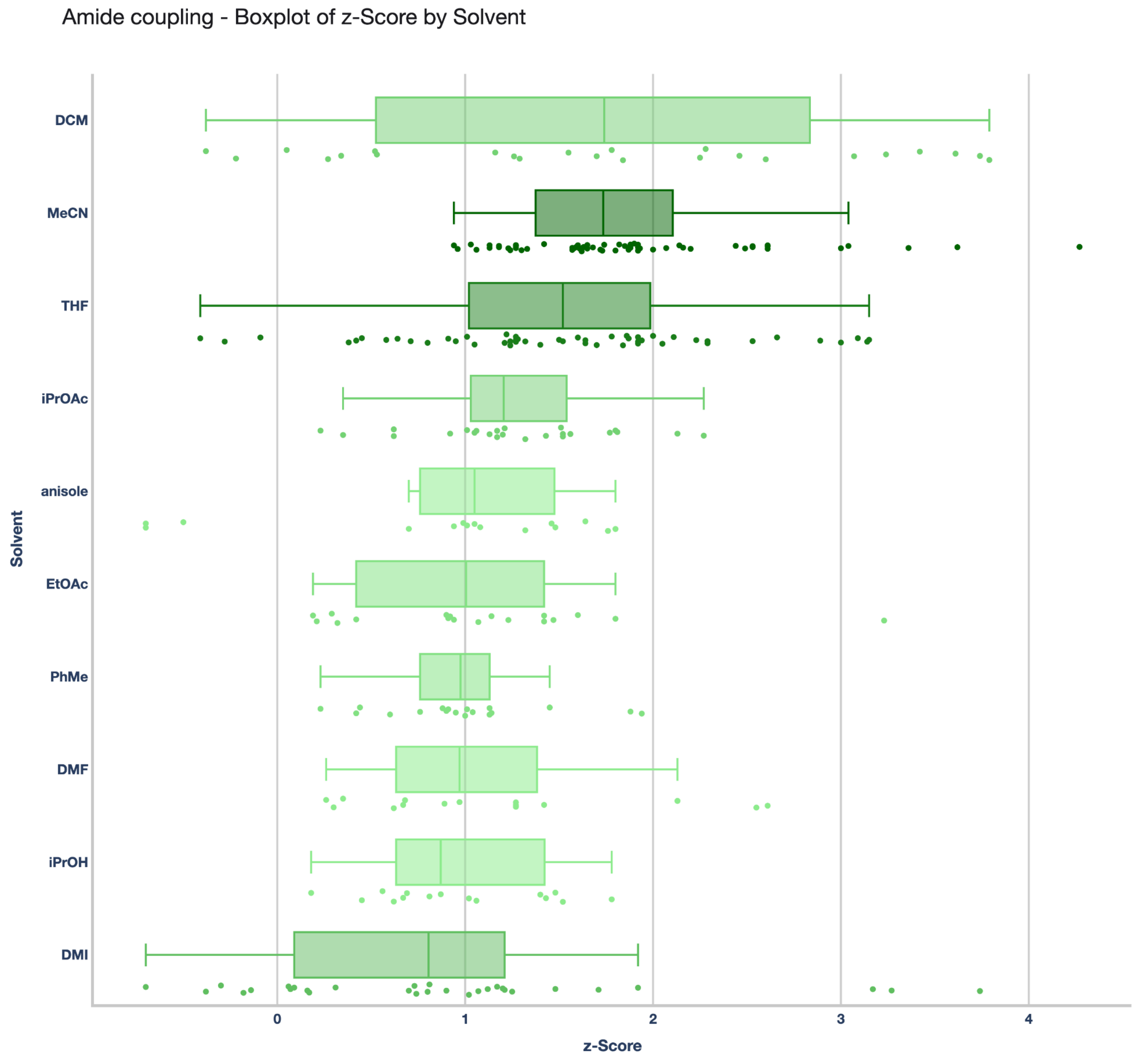

Figure S27: Solvent boxplot for Amide coupling reactions

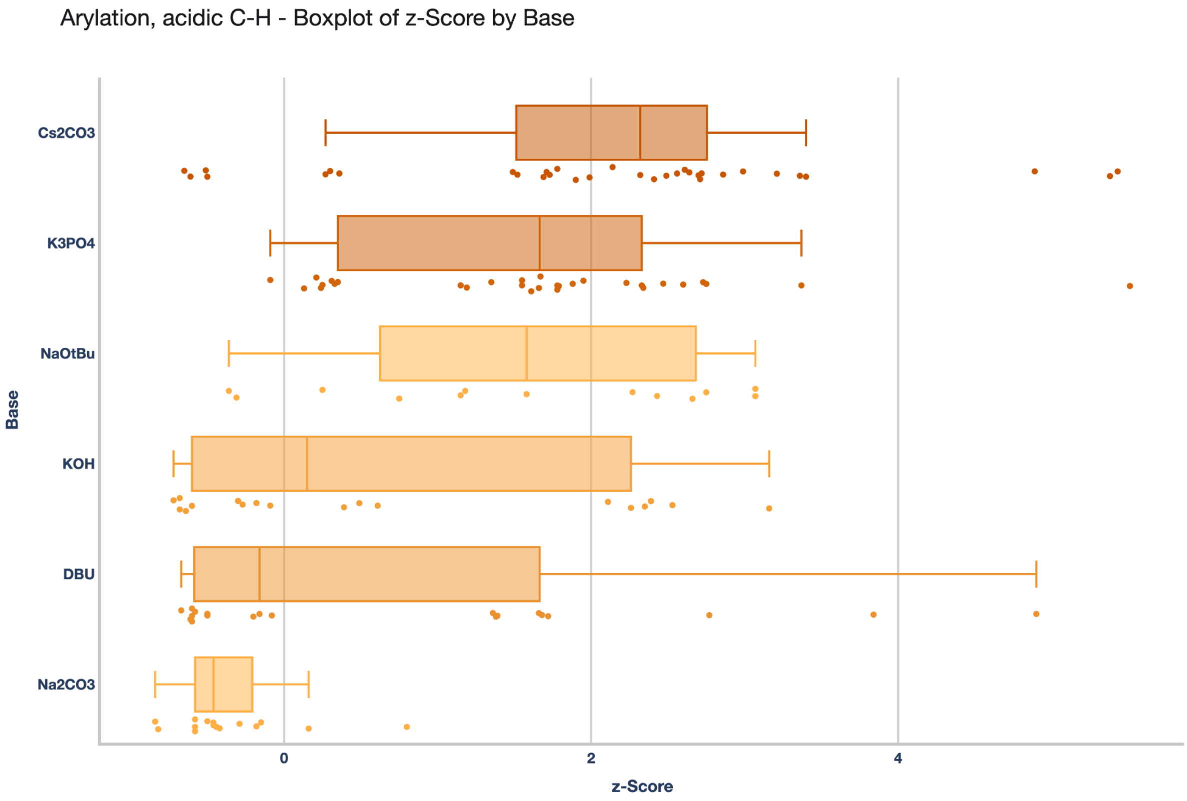

Figure S28: Base boxplot for Arylation, acidic C-H reactions

Arylation, acidic C-H - Boxplot of z-Score by Catalyst

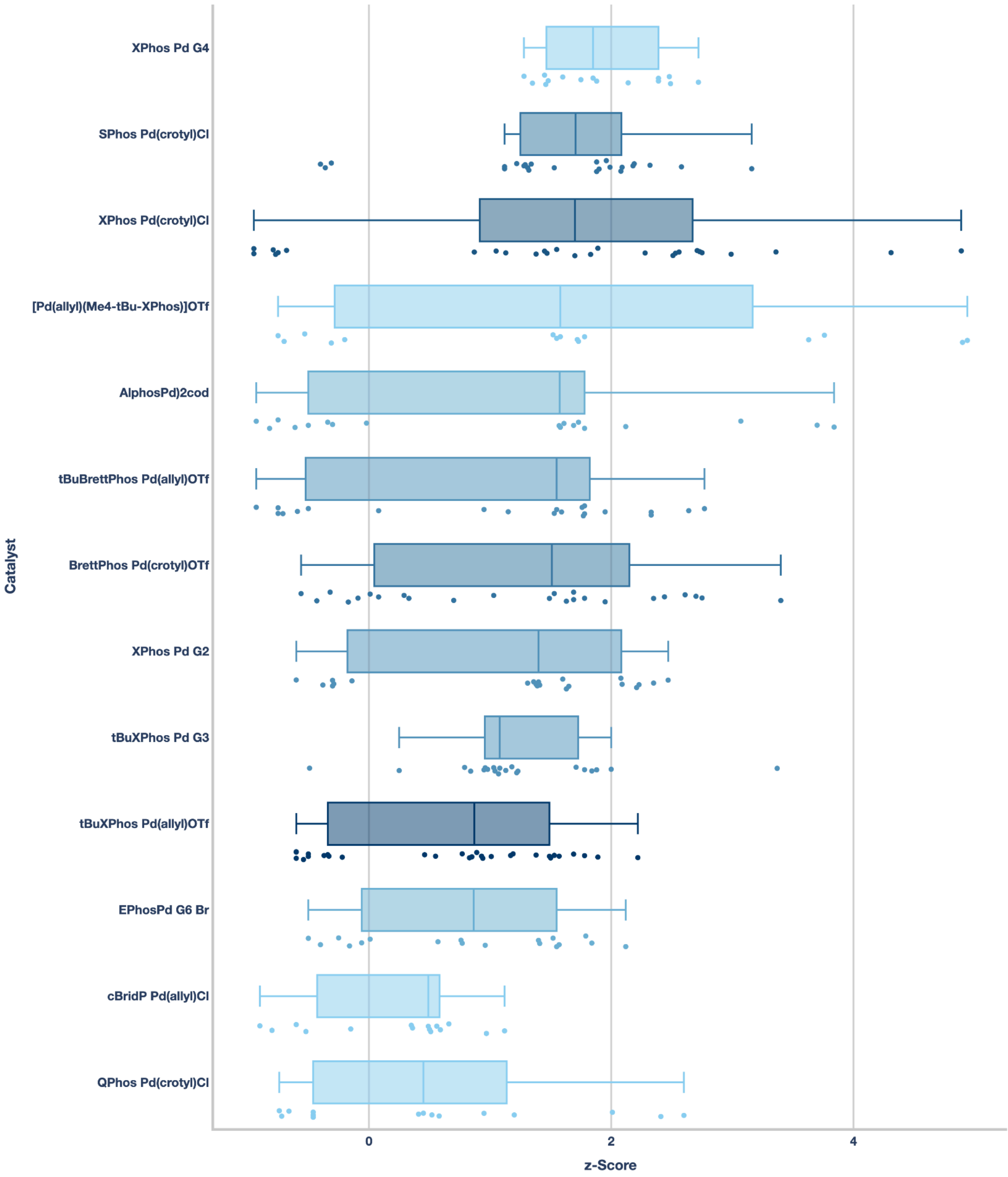

Figure S29: Catalyst boxplot for Arylation, acidic C-H reactions

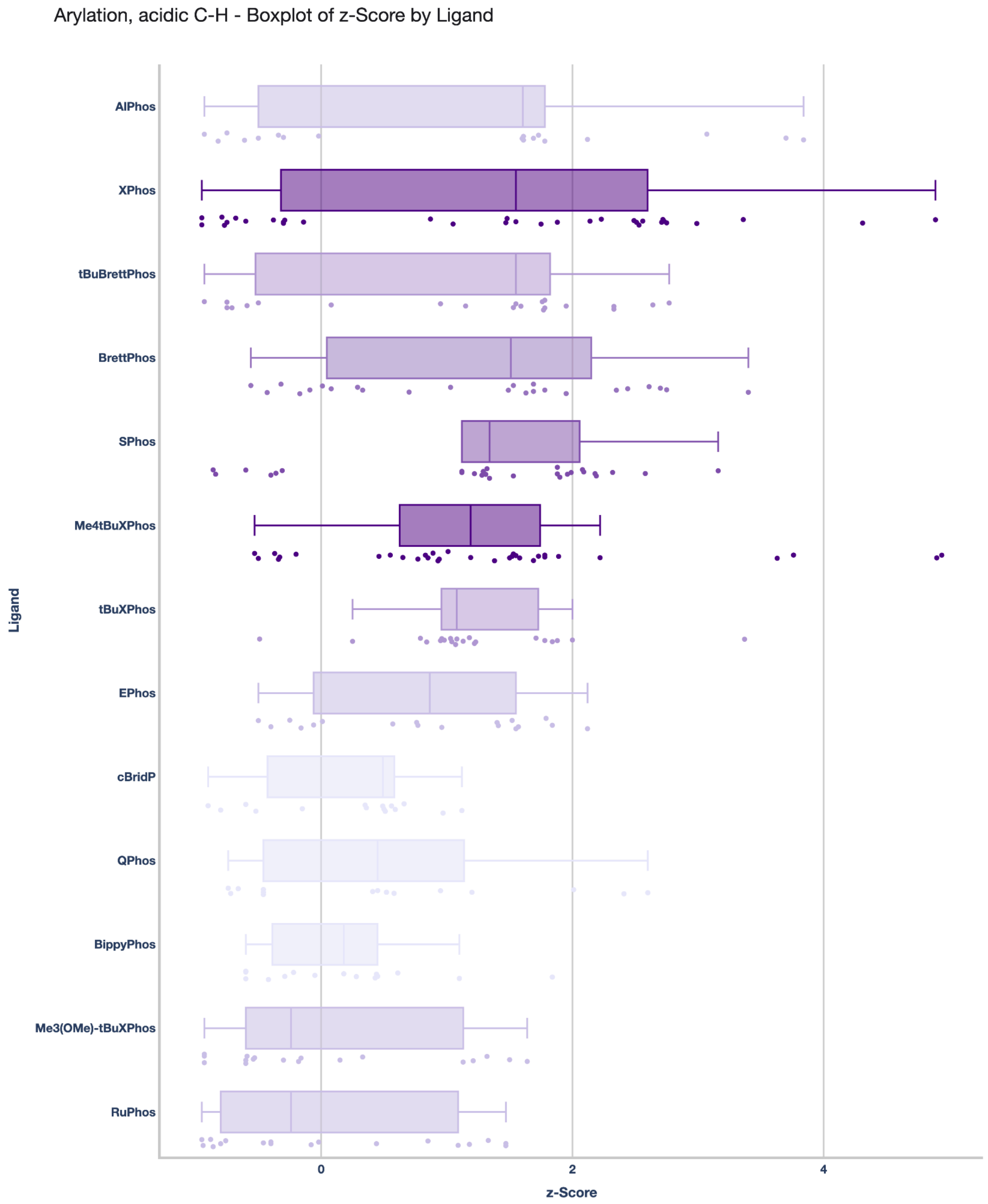

Figure S30: Ligand boxplot for Arylation, acidic C-H reactions

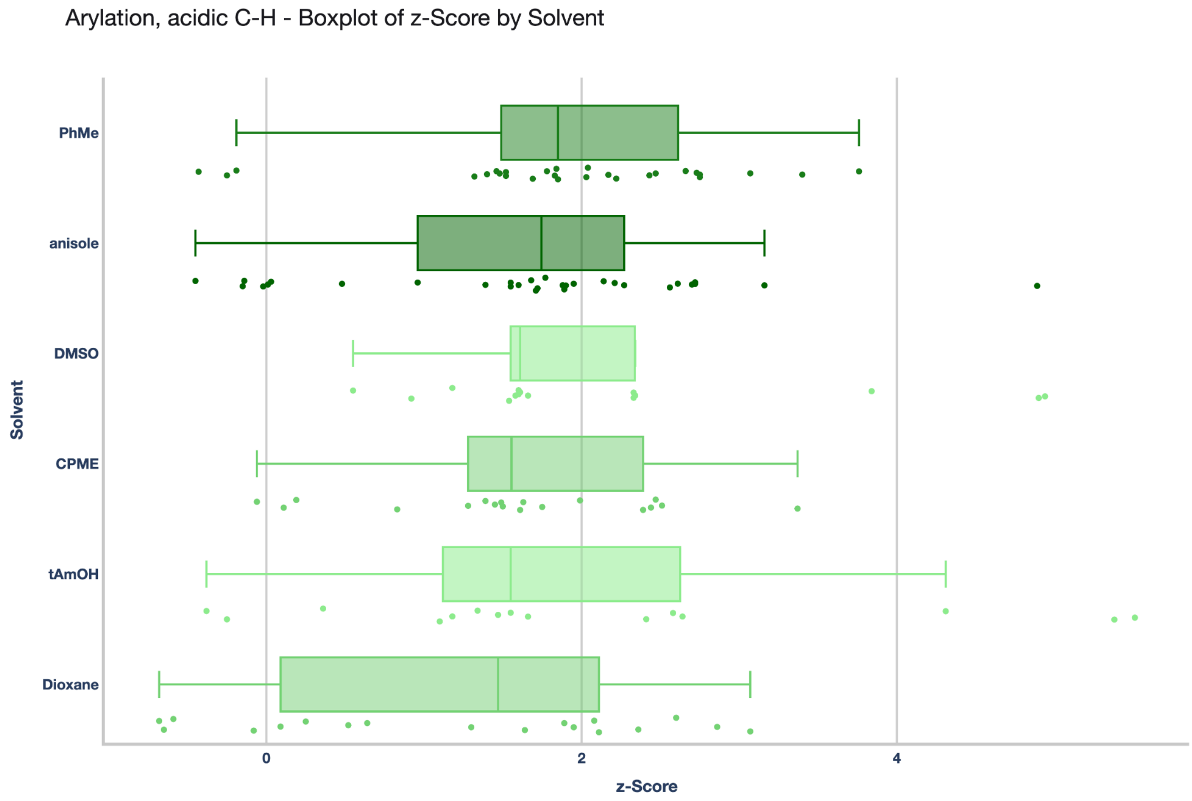

Figure S31: Solvent boxplot for Arylation, acidic C-H reactions

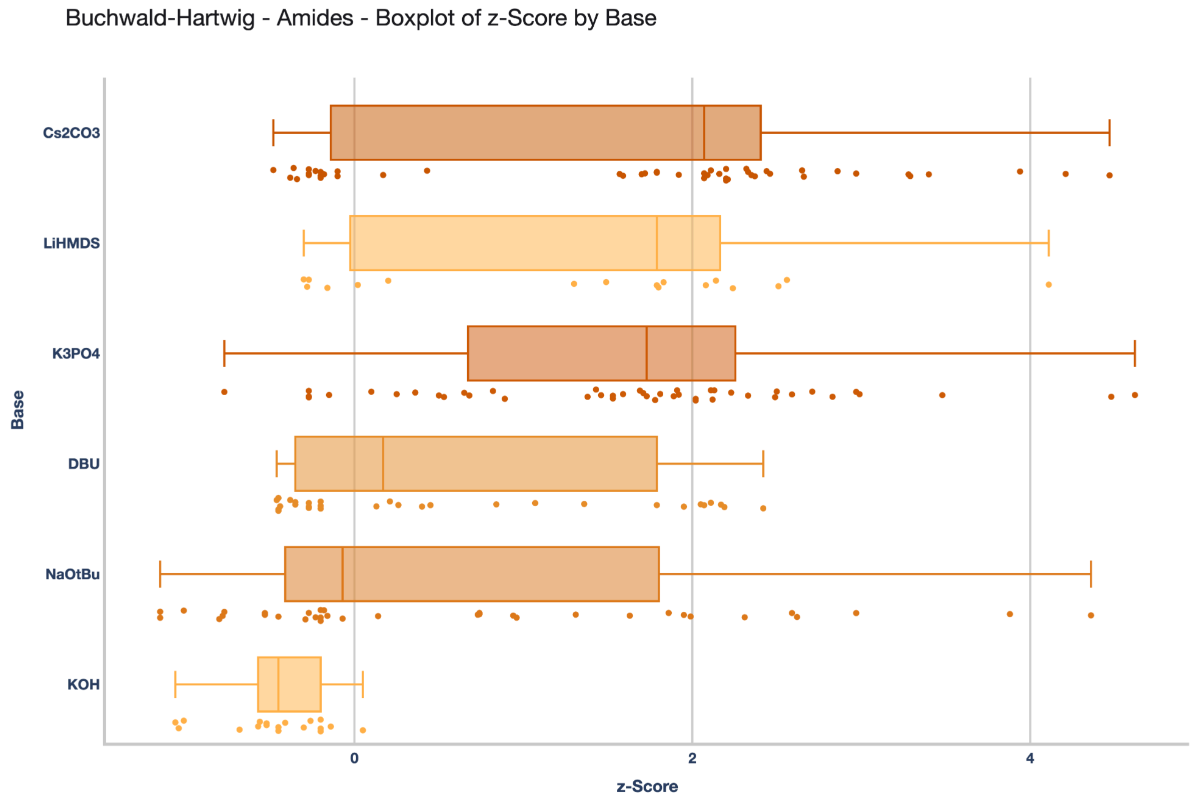

Figure S32: Base boxplot for Buchwald-Hartwig reactions for Amides reacting with aryl halides. Most weak inorganic bases with the exception of Cs_2CO_3 are generally being used as aqueous solutions. If solid K_2CO_3 is used, we employ a 325 mesh variant.

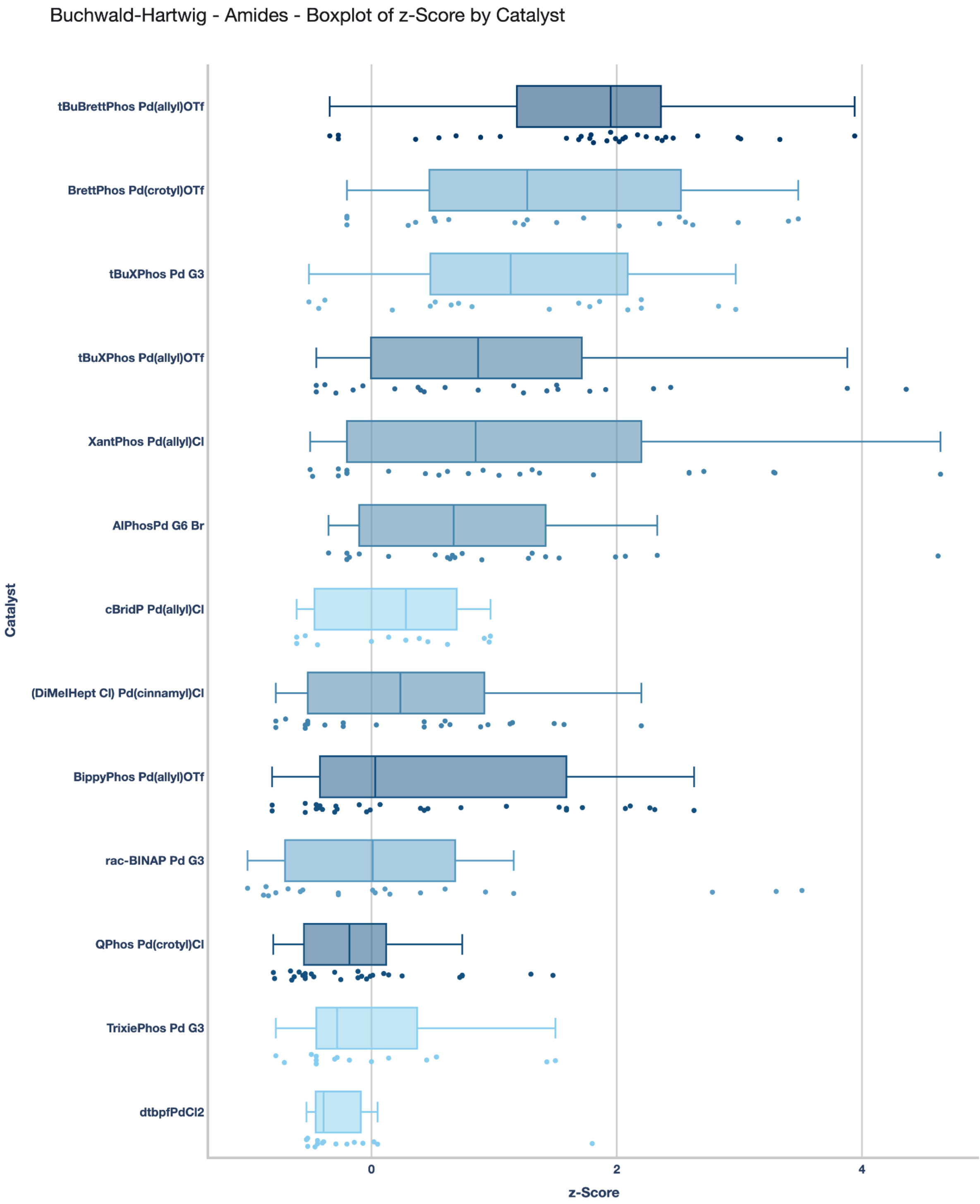

Figure S33: Catalyst boxplot for Buchwald-Hartwig reactions for Amides reacting with aryl halides

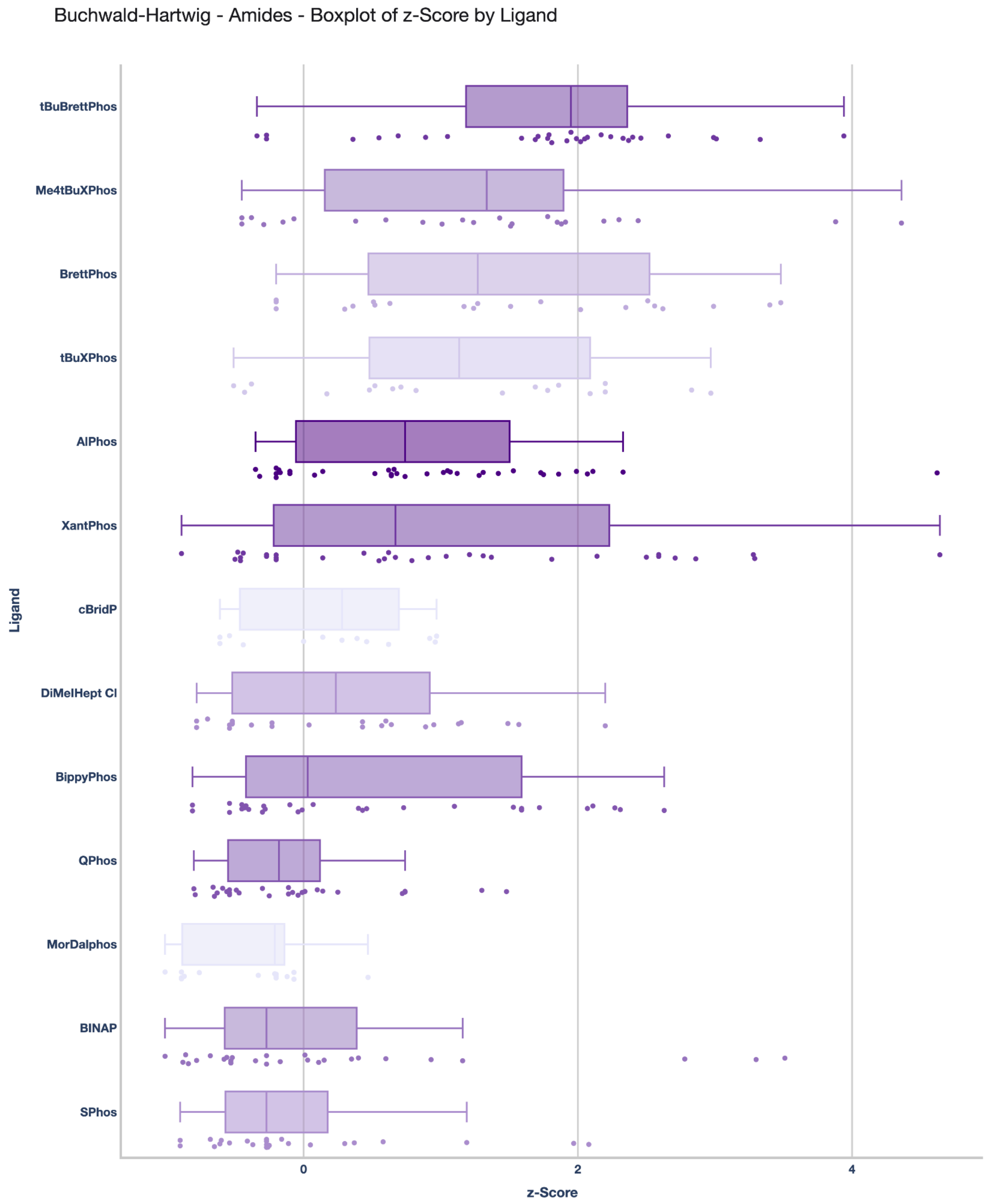

Figure S34: Ligand boxplot for Buchwald-Hartwig reactions for Amides reacting with aryl halides

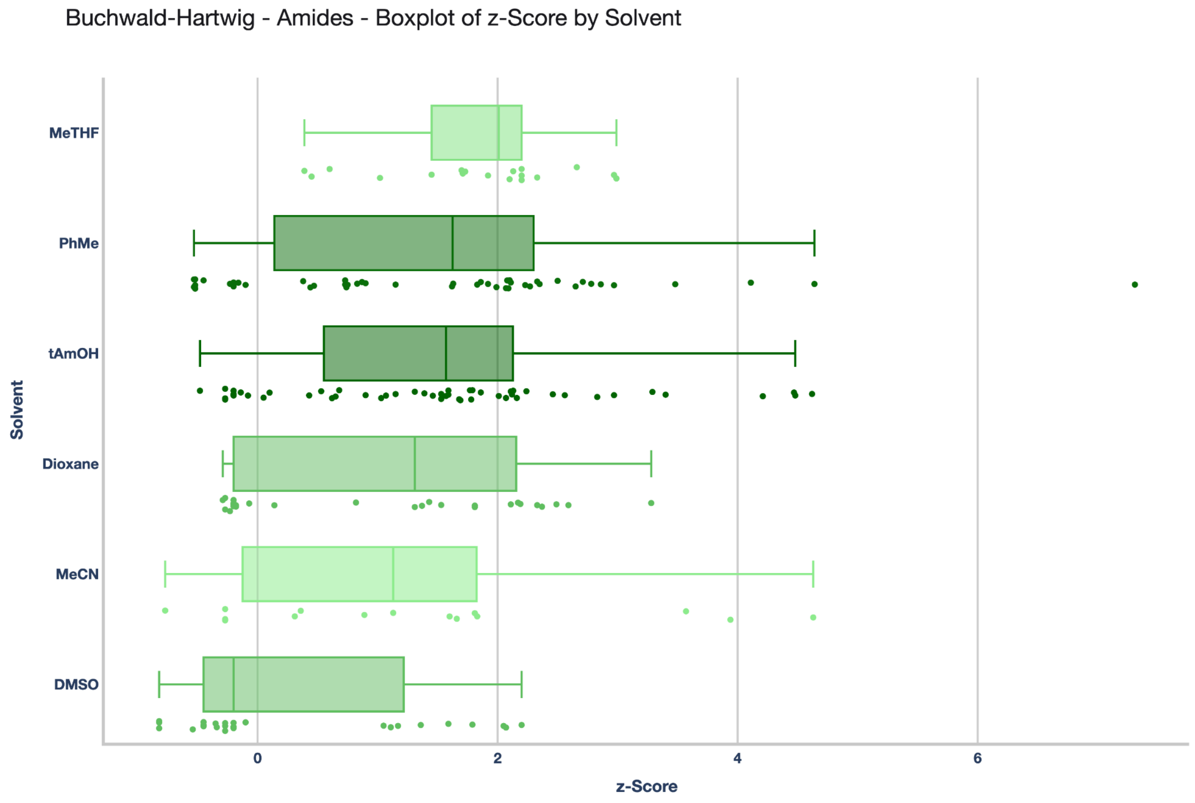

Figure S35: Solvent boxplot for Buchwald-Hartwig reactions for Amides reacting with aryl halides

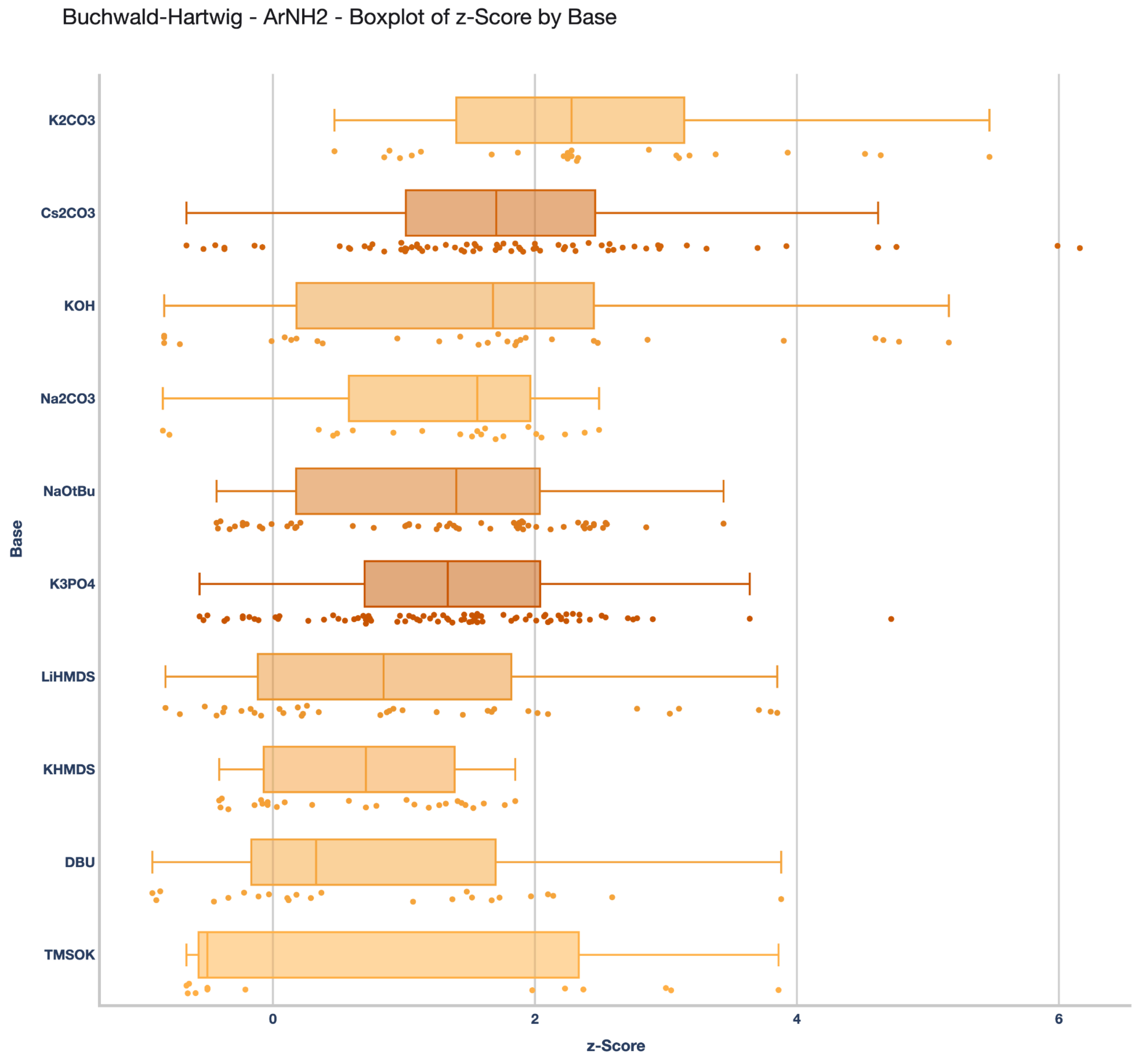

Figure S36: Base boxplot for Buchwald-Hartwig reactions for $ArNH_2$ reacting with aryl halides. Most weak inorganic bases with the exception of Cs_2CO_3 are generally being used as aqueous solutions. If solid K_2CO_3 is used, we employ a 325 mesh variant.

Buchwald-Hartwig - ArNH2 - Boxplot of z-Score by Catalyst

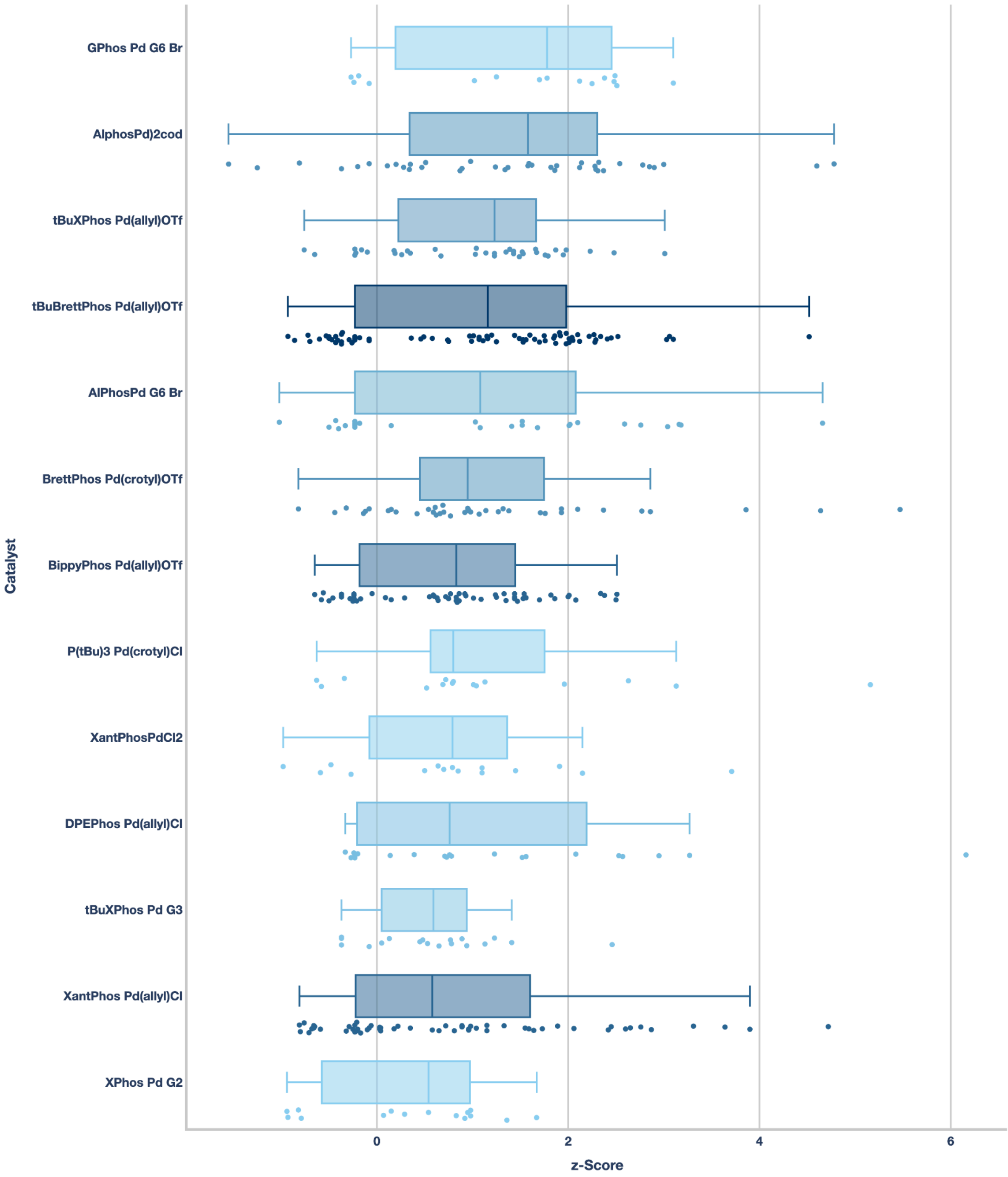

Figure S37: Catalyst boxplot for Buchwald-Hartwig reactions for $ArNH_2$ reacting with aryl halides

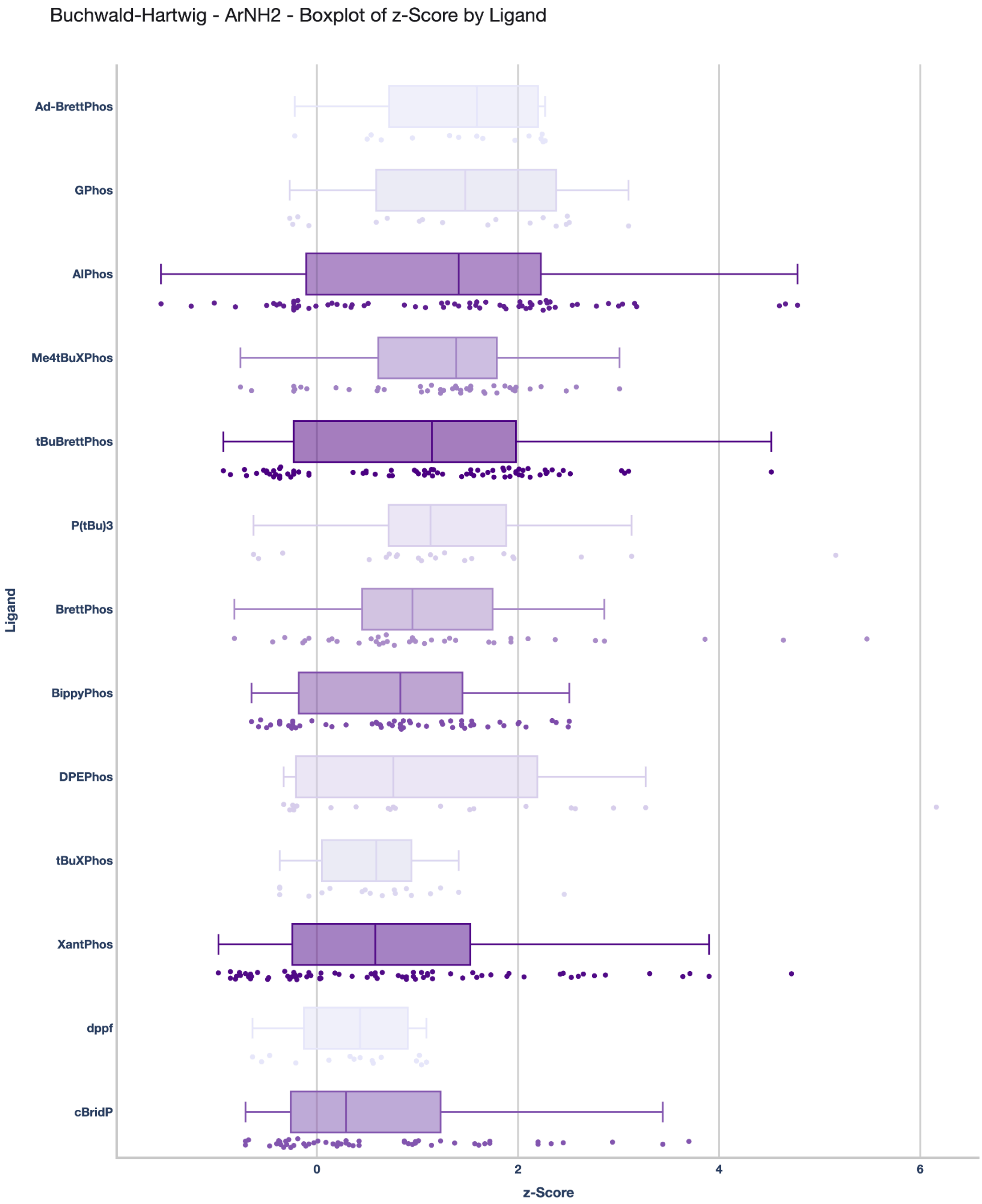

Figure S38: Ligand boxplot for Buchwald-Hartwig reactions for $ArNH_2$ reacting with aryl halides

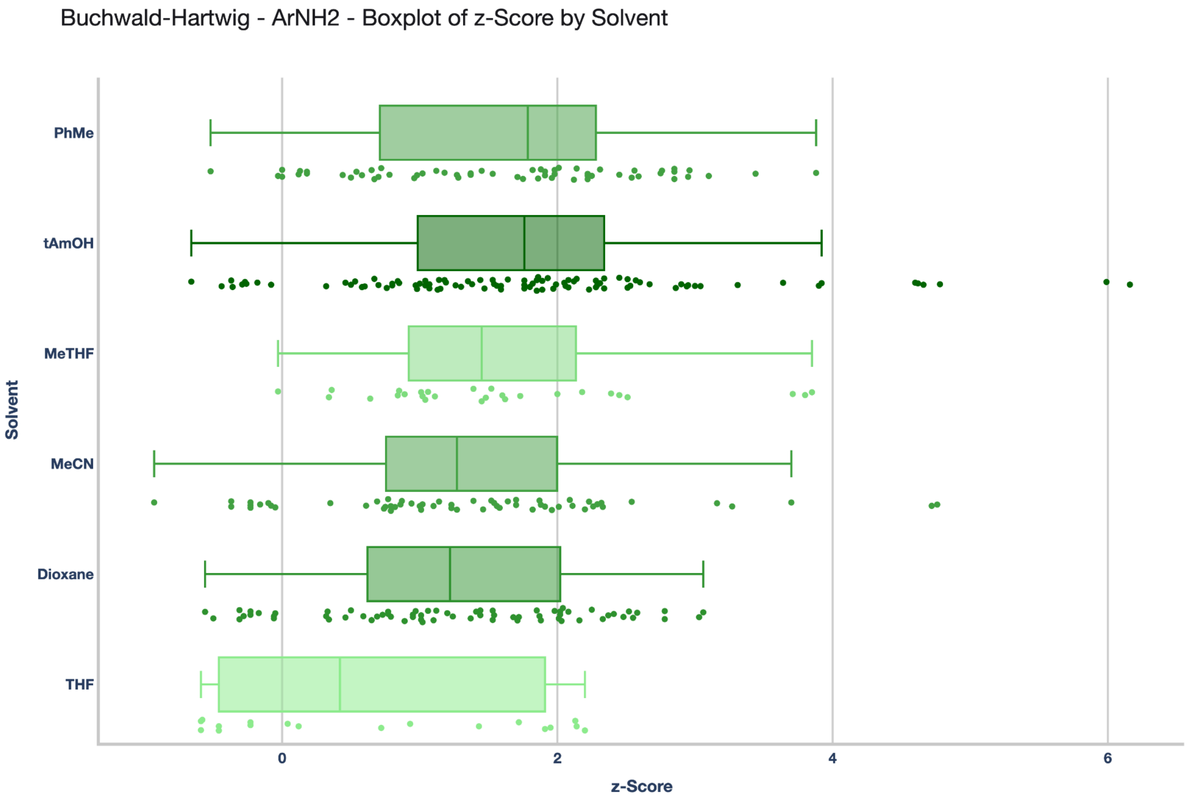

Figure S39: Solvent boxplot for Buchwald-Hartwig reactions for $ArNH_2$ reacting with aryl halides

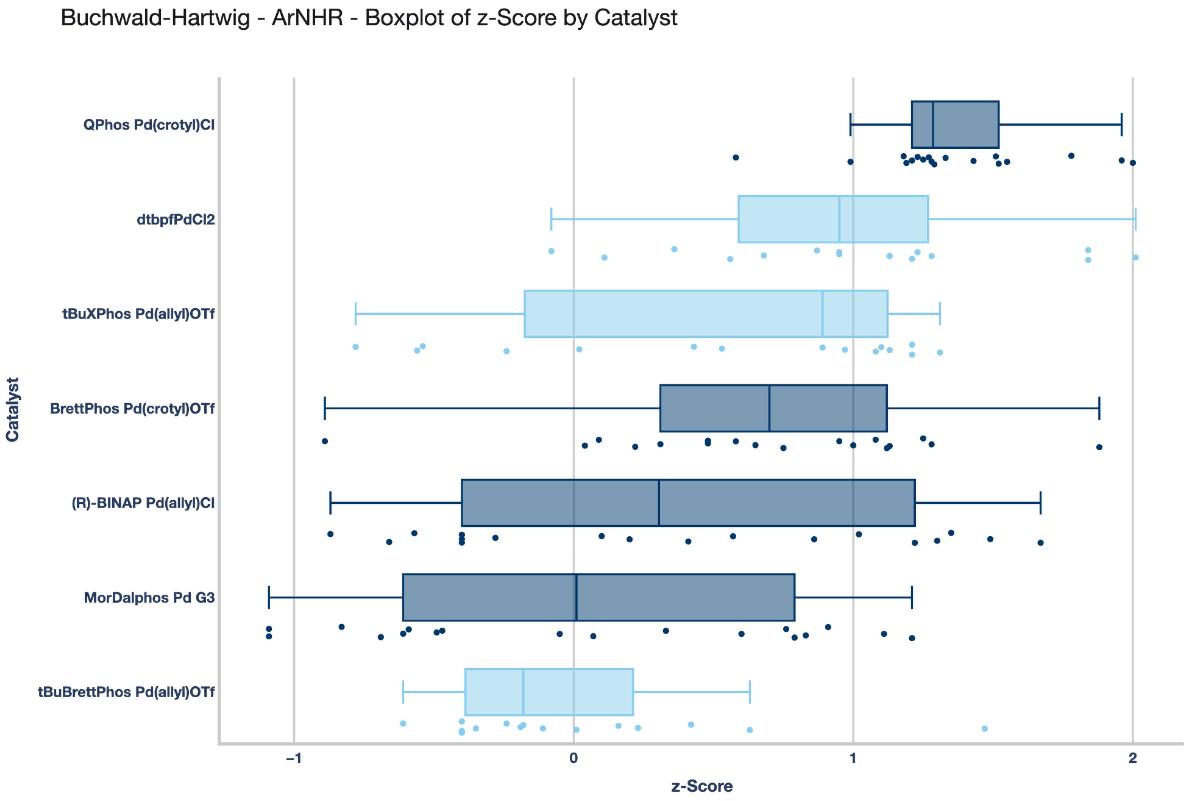

Figure S40: Catalyst boxplot for Buchwald-Hartwig reactions for ArNHR reacting with aryl halides

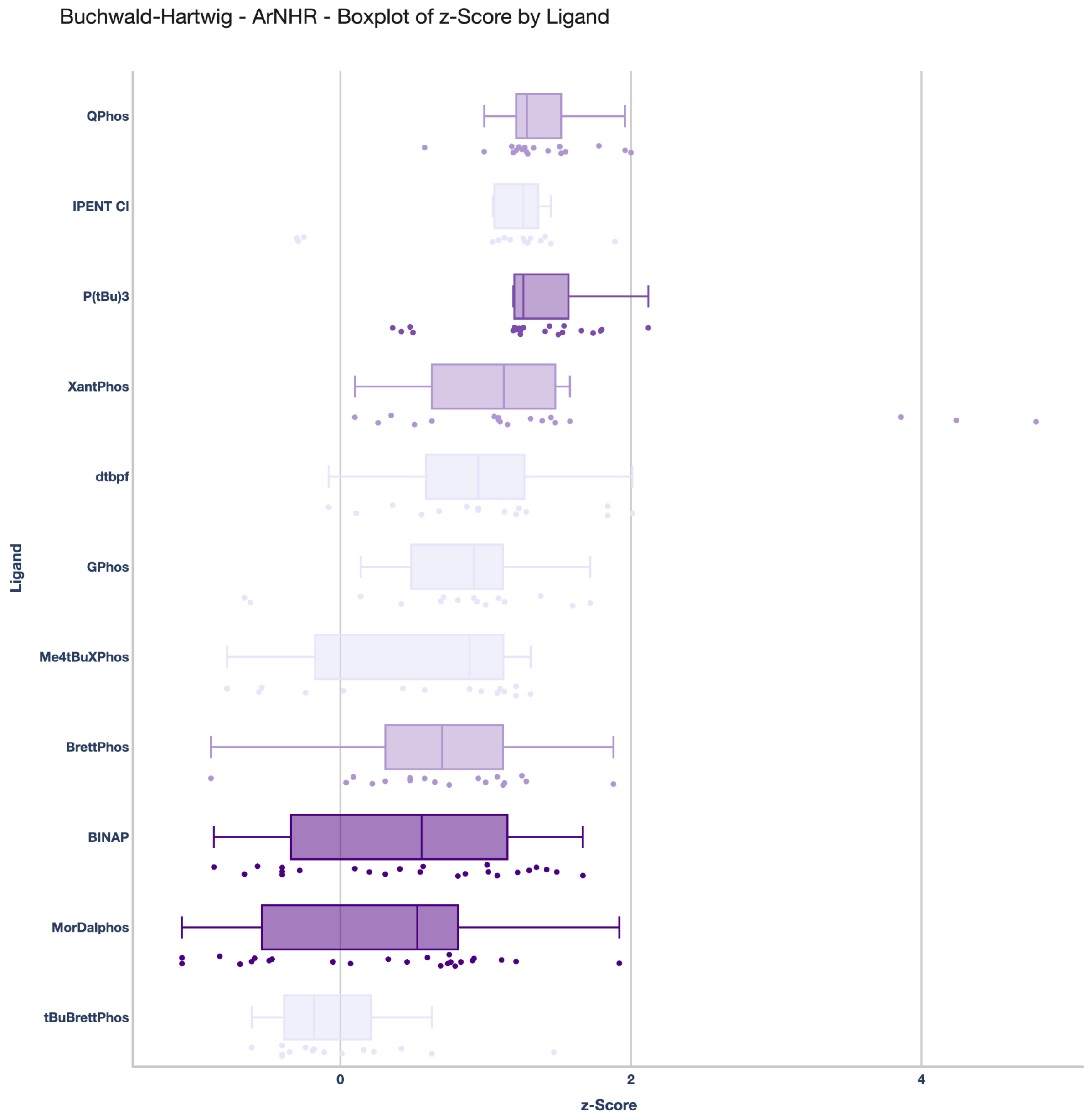

Figure S41: Ligand boxplot for Buchwald-Hartwig reactions for ArNHR reacting with aryl halides

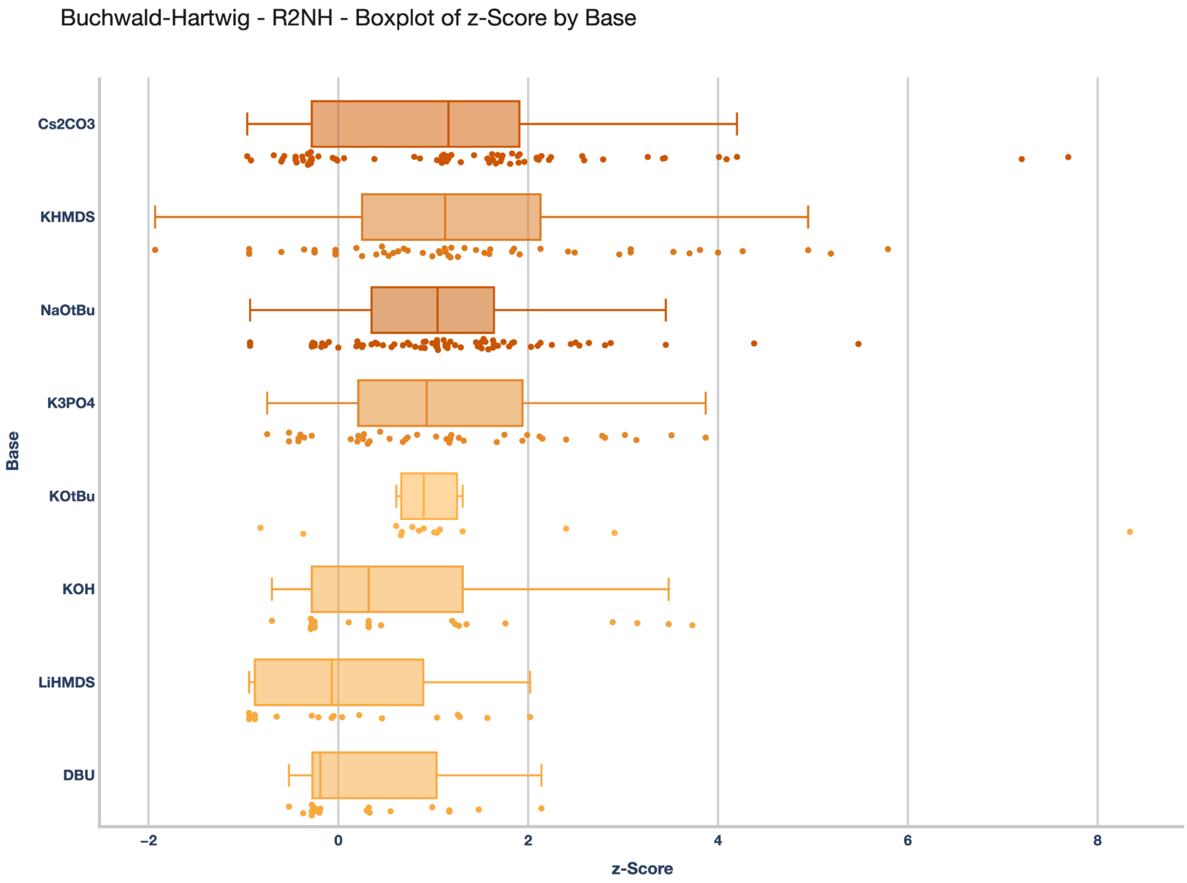

Figure S42: Base boxplot for Buchwald-Hartwig reactions for R_2NH reacting with aryl halides. Most weak inorganic bases with the exception of Cs_2CO_3 are generally being used as aqueous solutions. If solid K_2CO_3 is used, we employ a 325 mesh variant.

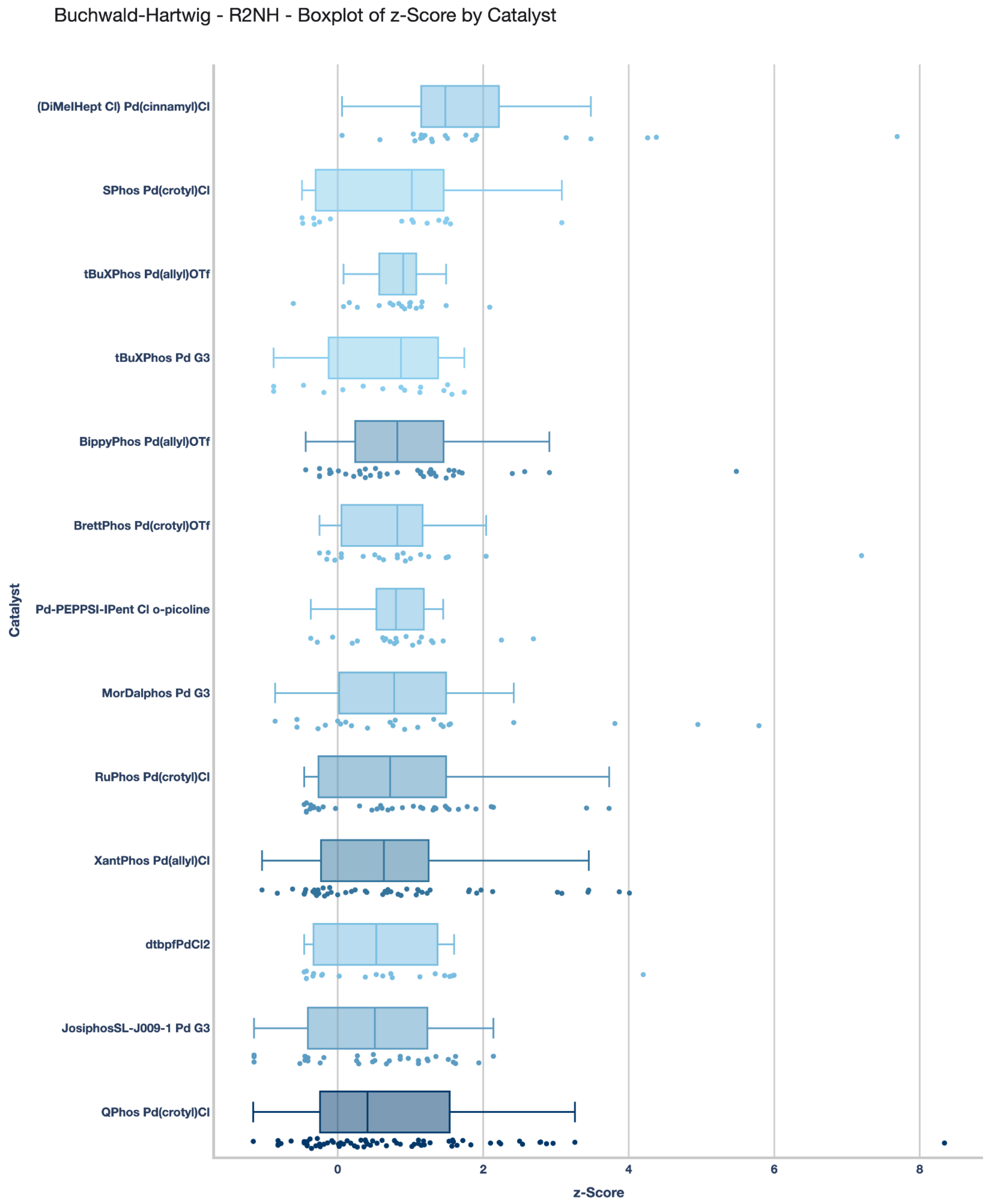

Figure S43: Catalyst boxplot for Buchwald-Hartwig reactions for R_2NH reacting with aryl halides

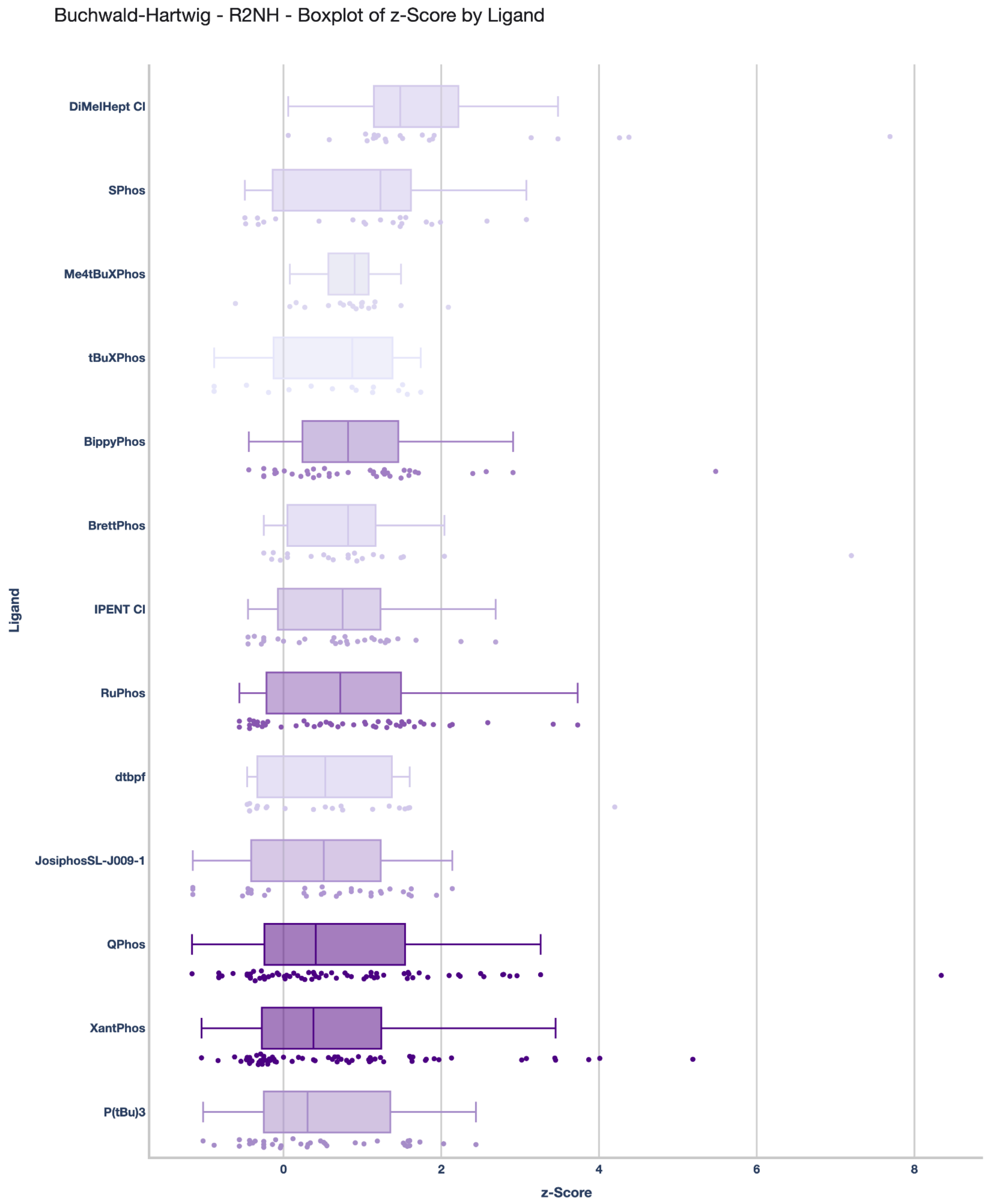

Figure S44: Ligand boxplot for Buchwald-Hartwig reactions for R_2NH reacting with aryl halides

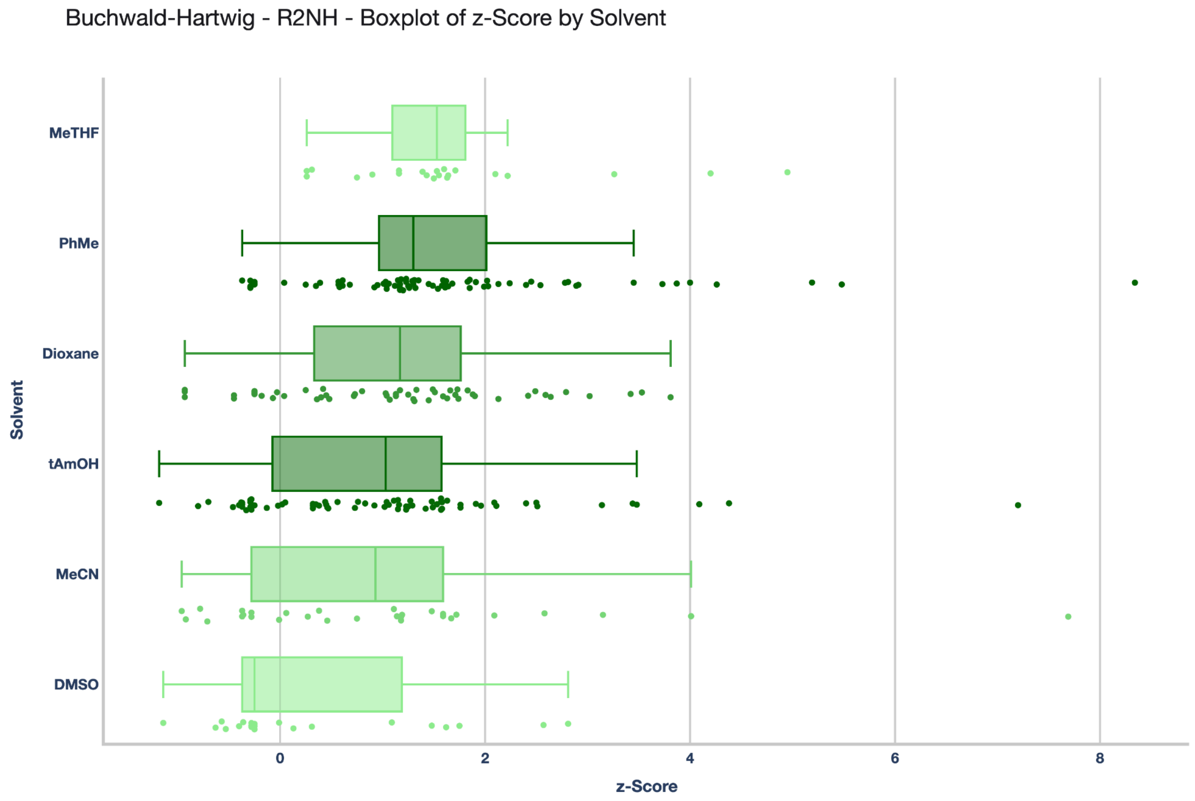

Figure S45: Solvent boxplot for Buchwald-Hartwig reactions for R_2NH reacting with aryl halides

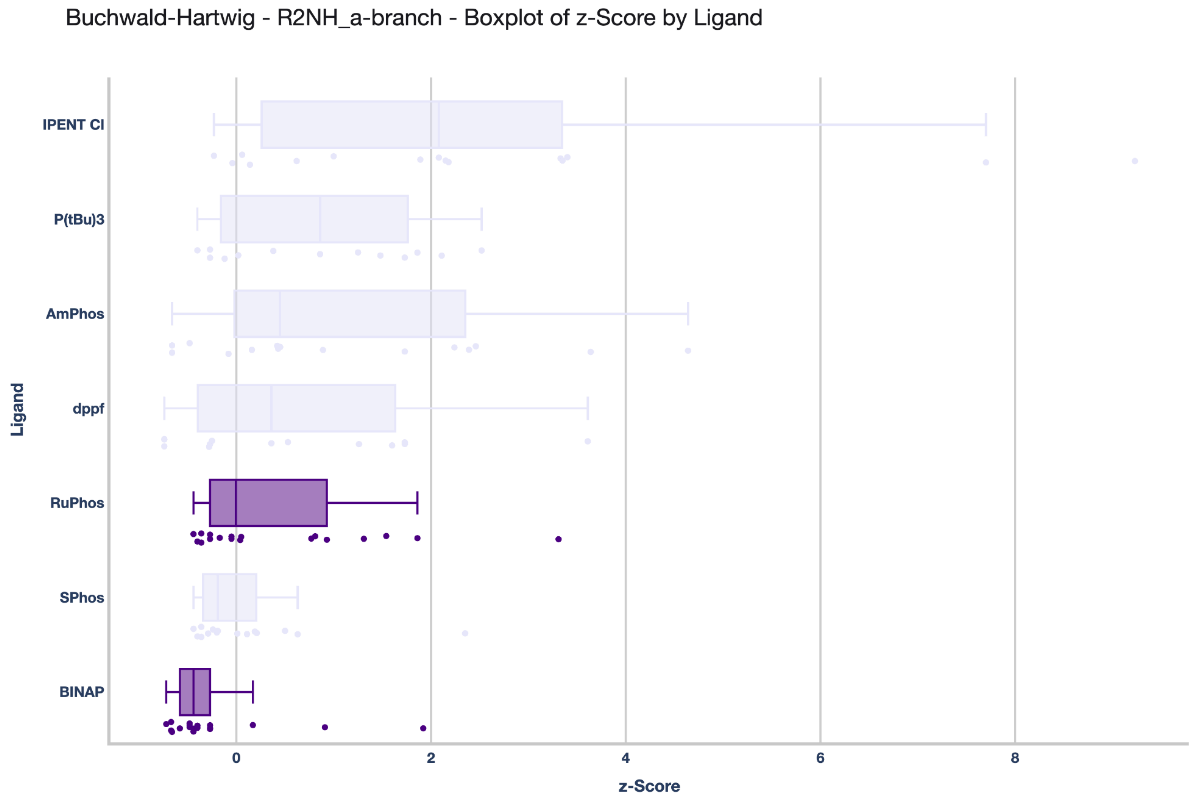

Figure S46: Ligand boxplot for Buchwald-Hartwig reactions for R_2NH a-branch reacting with aryl halides

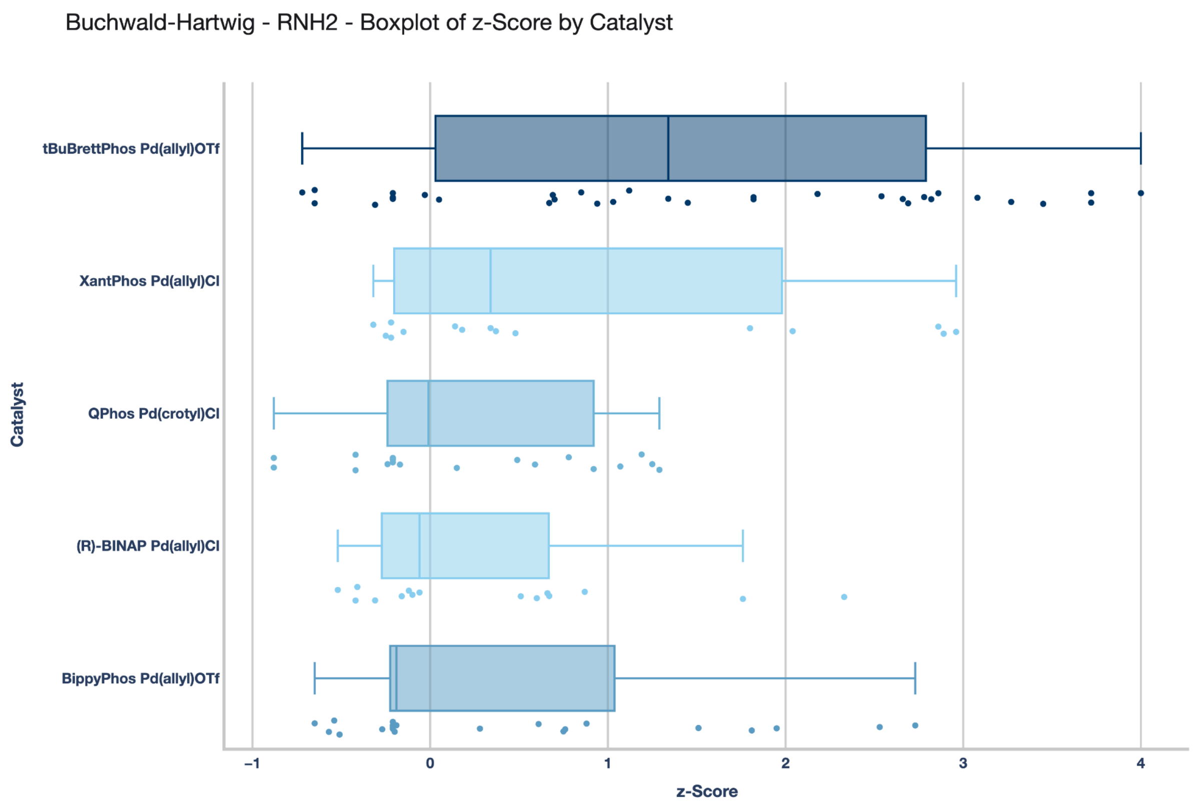

Figure S47: Catalyst boxplot for Buchwald-Hartwig reactions for RNH_2 reacting with aryl halides

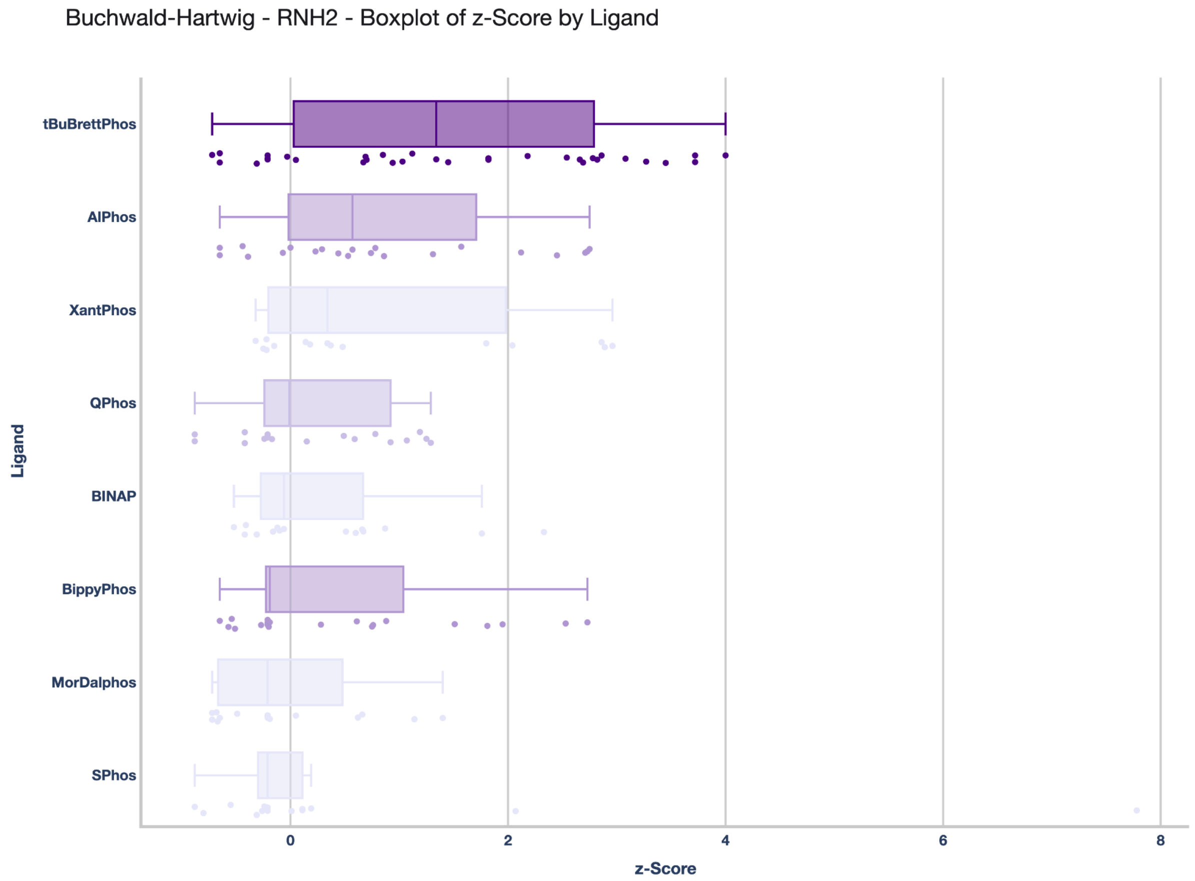

Figure S48: Ligand boxplot for Buchwald-Hartwig reactions for RNH_2 reacting with aryl halides

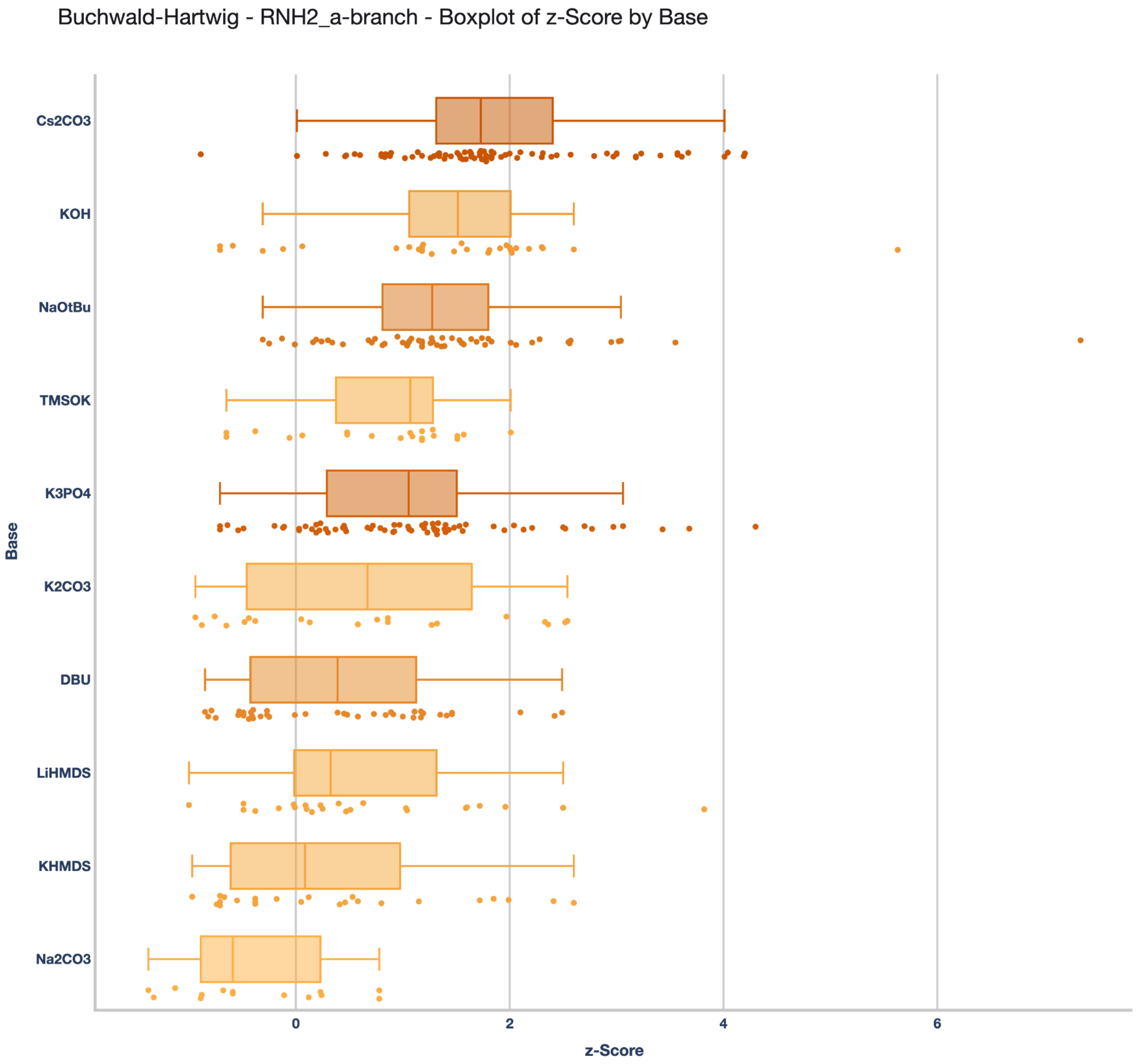

Figure S49: Base boxplot for Buchwald-Hartwig reactions for RNH_2 a-branch reacting with aryl halides. Most weak inorganic bases with the exception of Cs_2CO_3 are generally being used as aqueous solutions. If solid K_2CO_3 is used, we employ a 325 mesh variant.

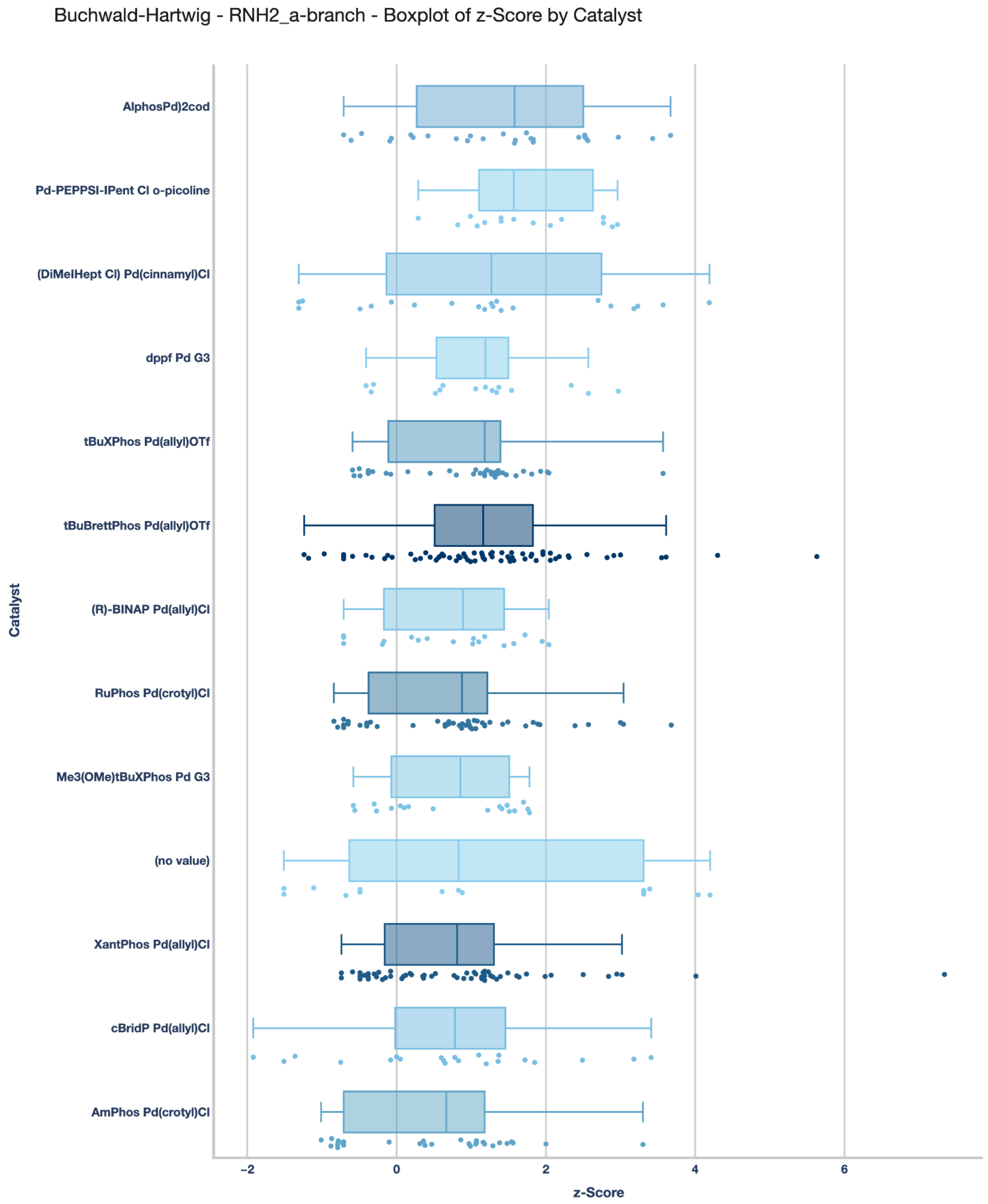

Figure S50: Catalyst boxplot for Buchwald-Hartwig reactions for RNH_2 a-branch reacting with aryl halides

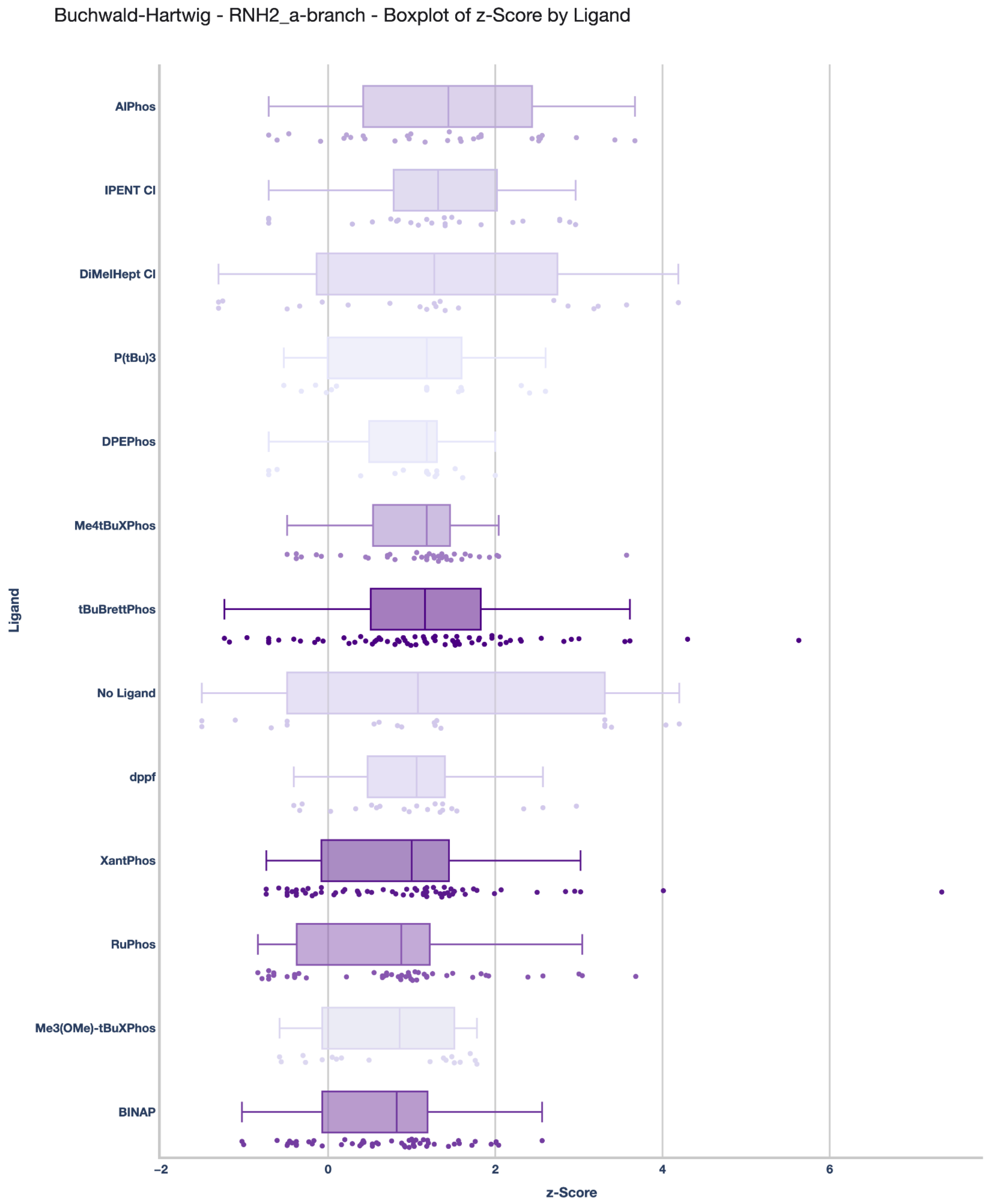

Figure S51: Ligand boxplot for Buchwald-Hartwig reactions for RNH_2 a-branch reacting with aryl halides

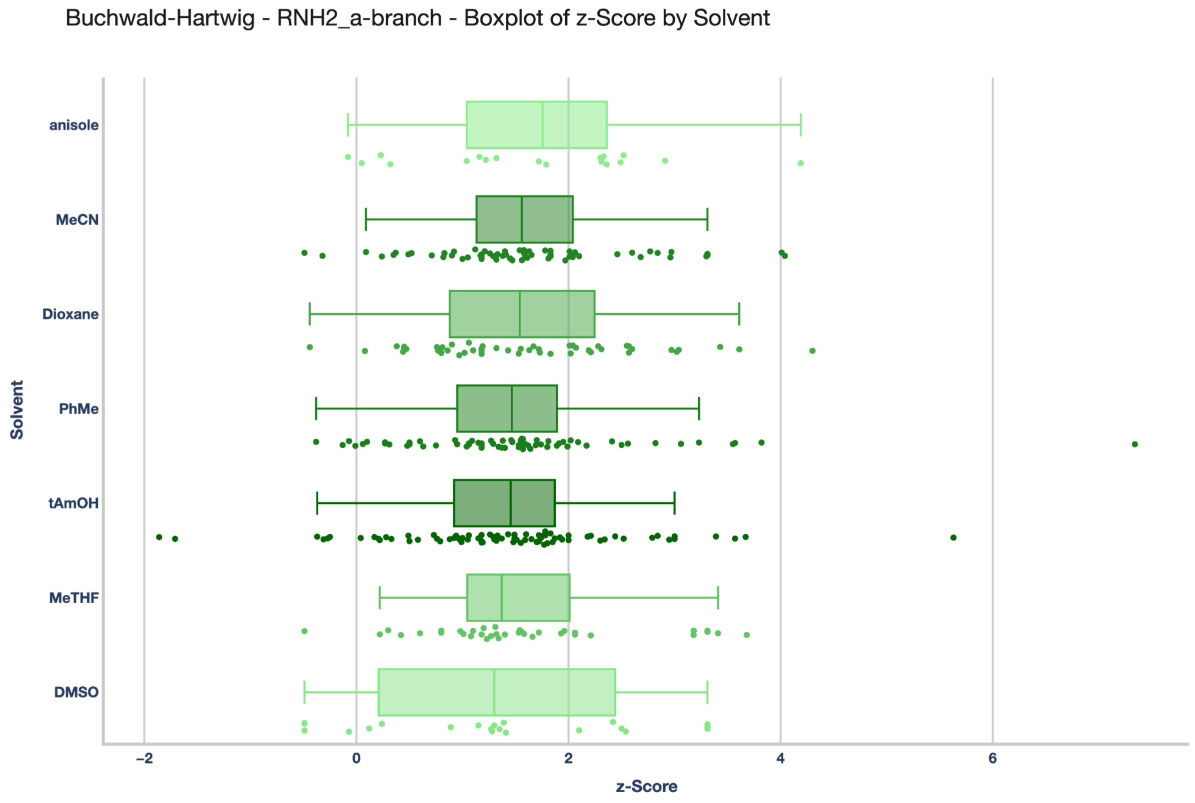

Figure S52: Solvent boxplot for Buchwald-Hartwig reactions for RNH_2 a-branch reacting with aryl halides

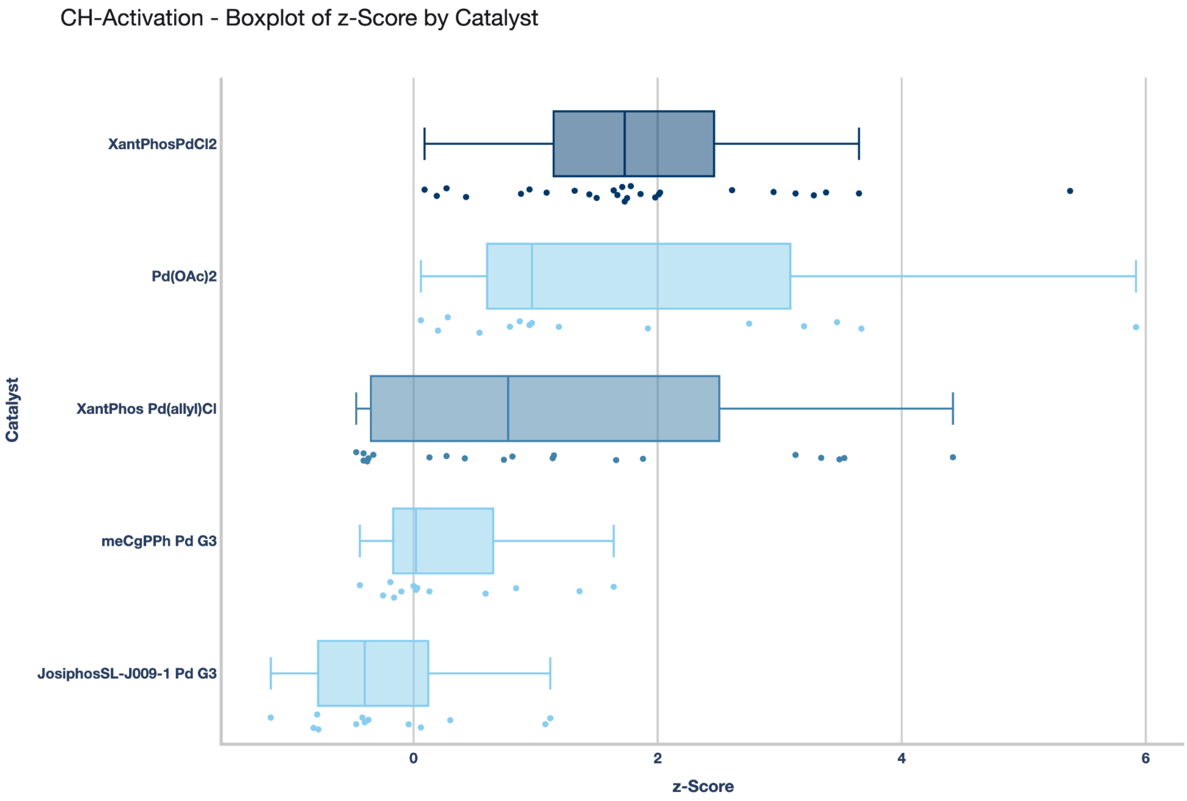

Figure S53: Catalyst boxplot for CH-Activation reactions

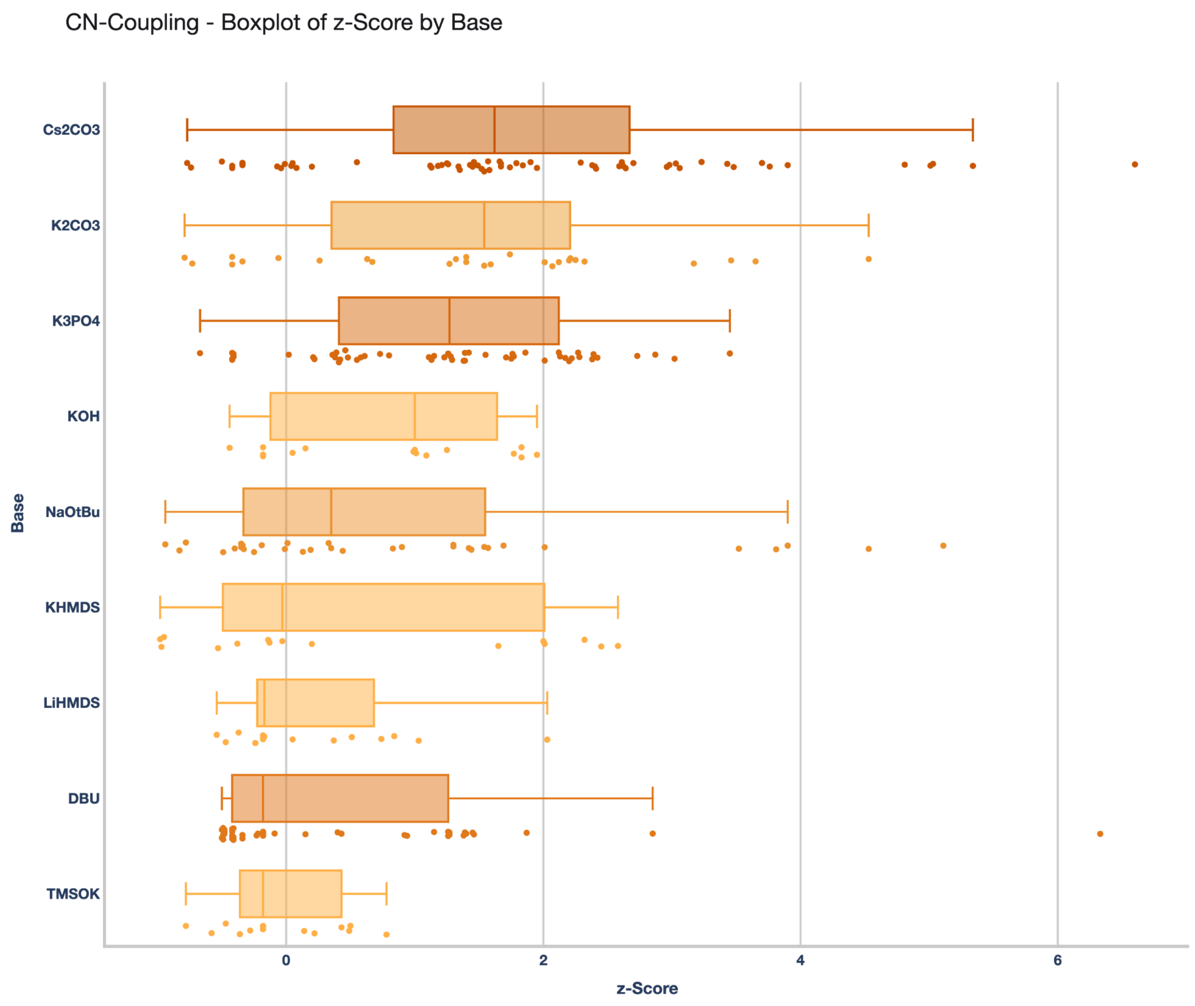

Figure S54: Base boxplot for CN-Coupling reactions

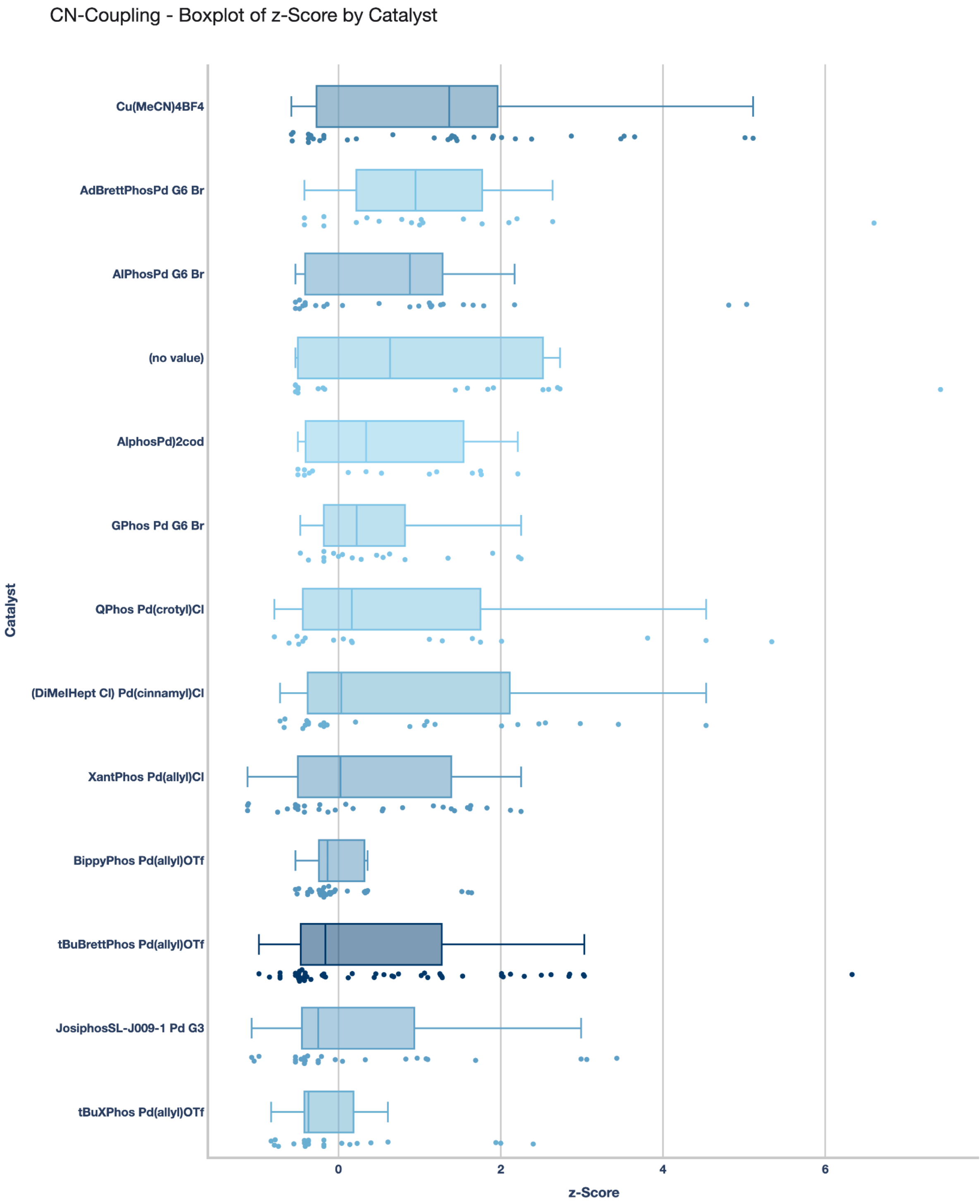

Figure S55: Catalyst boxplot for CN-Coupling reactions

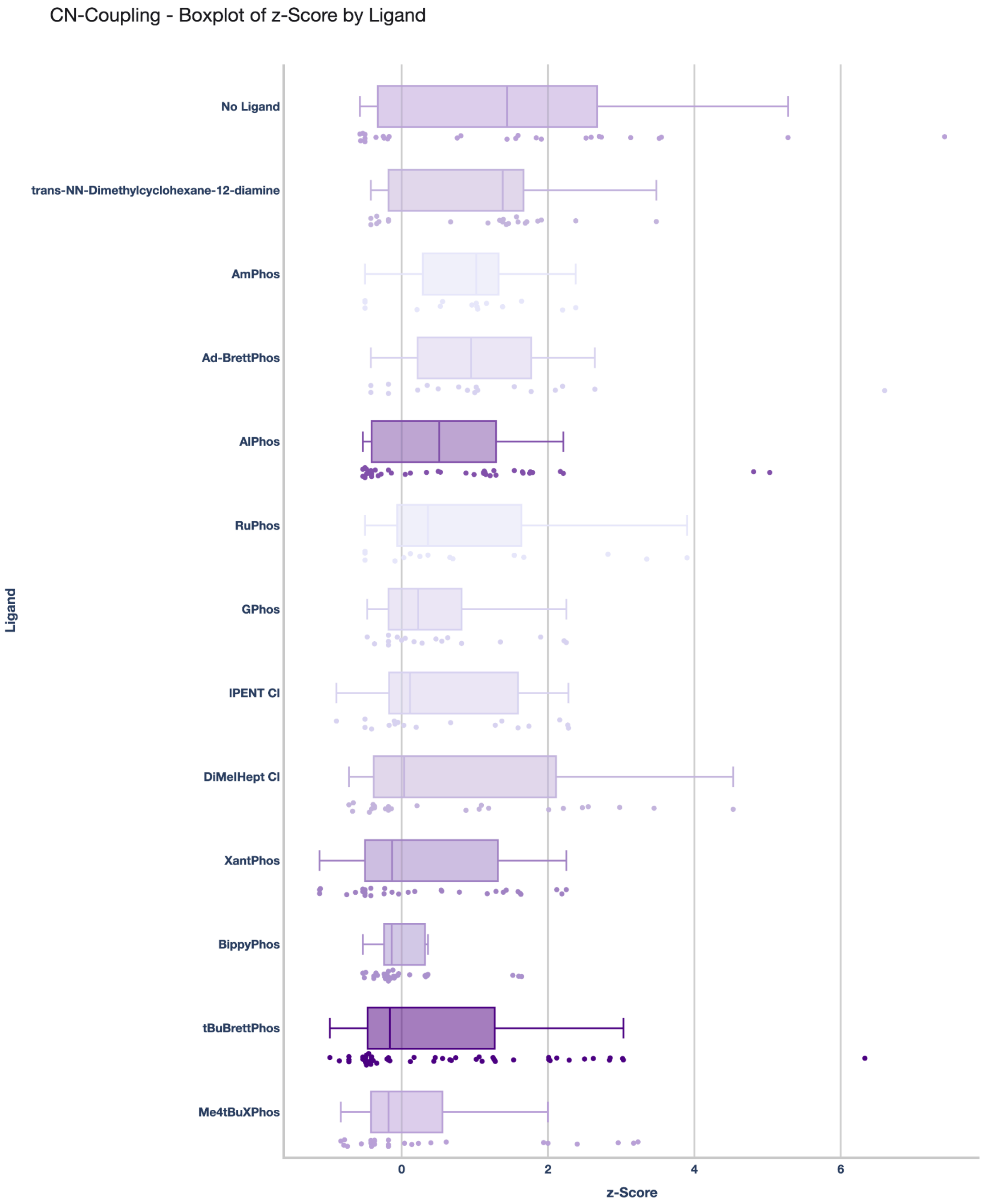

Figure S56: Ligand boxplot for CN-Coupling reactions

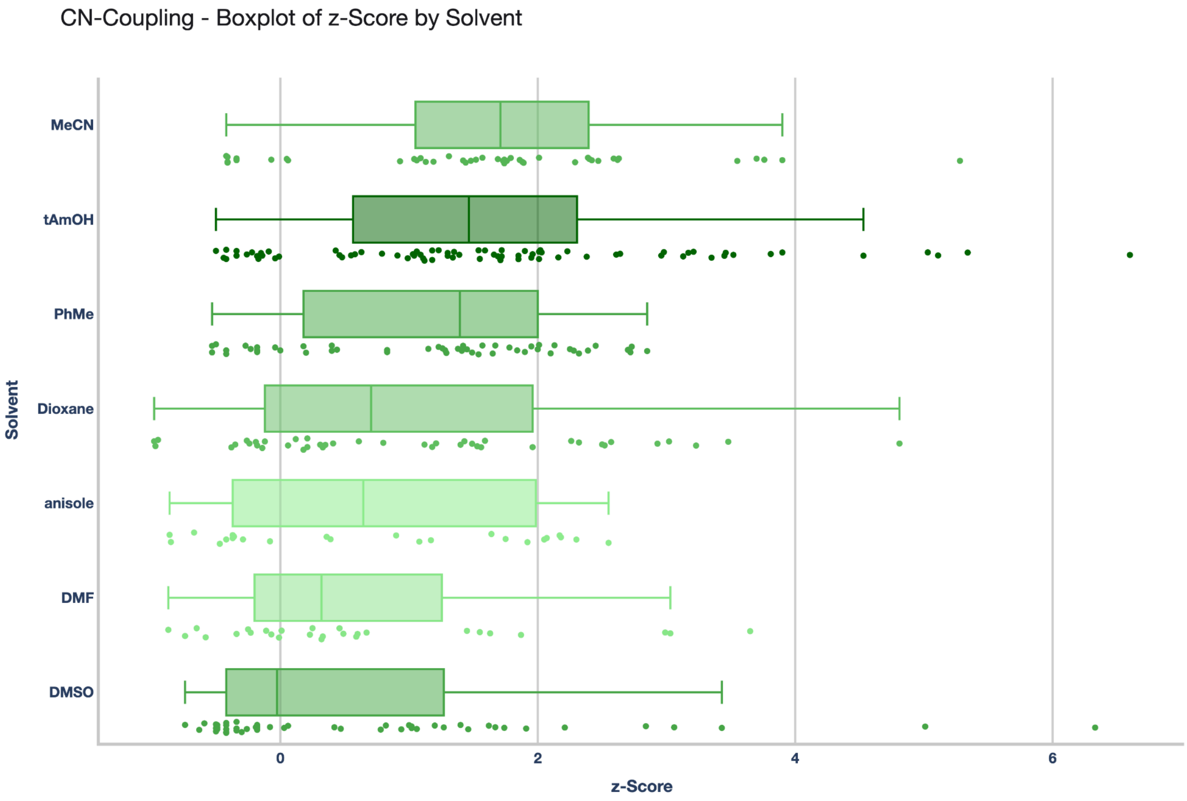

Figure S57: Solvent boxplot for CN-Coupling reactions

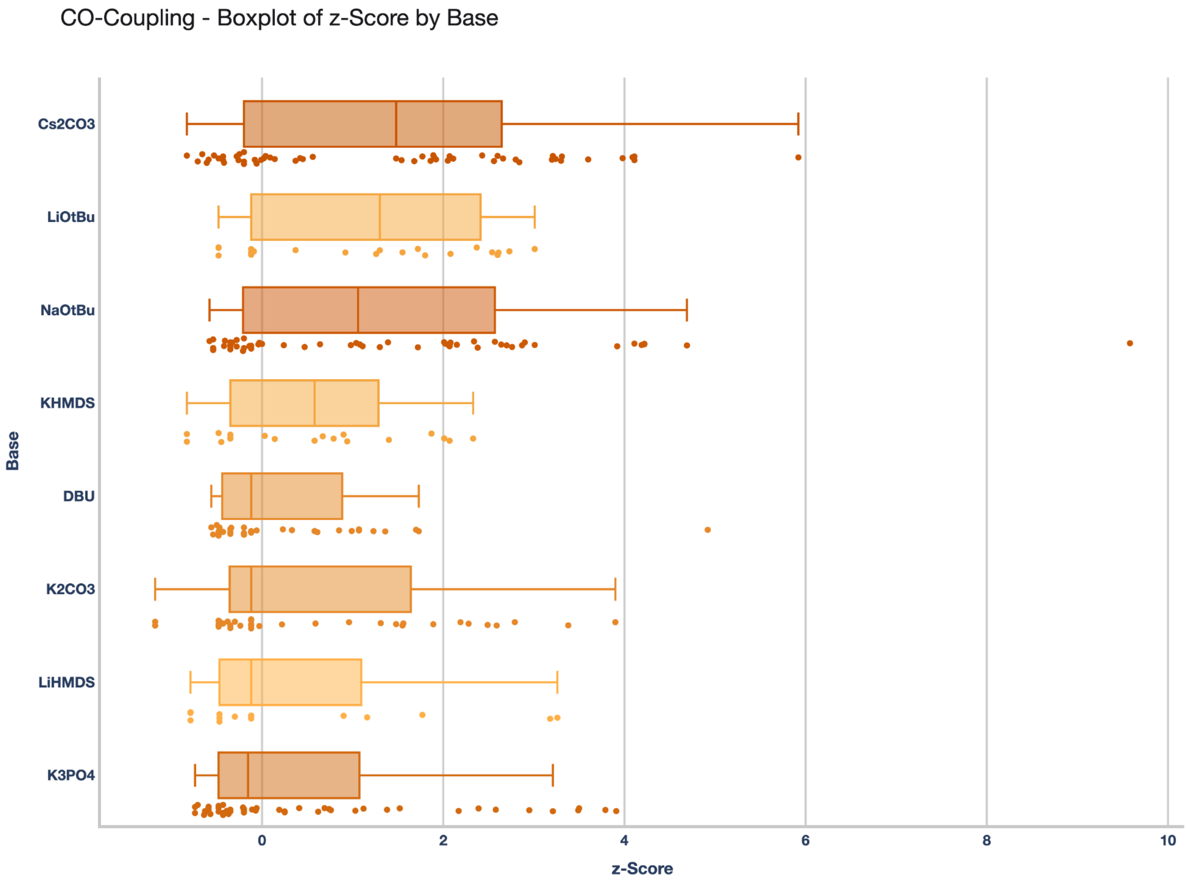

Figure S58: Base boxplot for CO-Coupling reactions

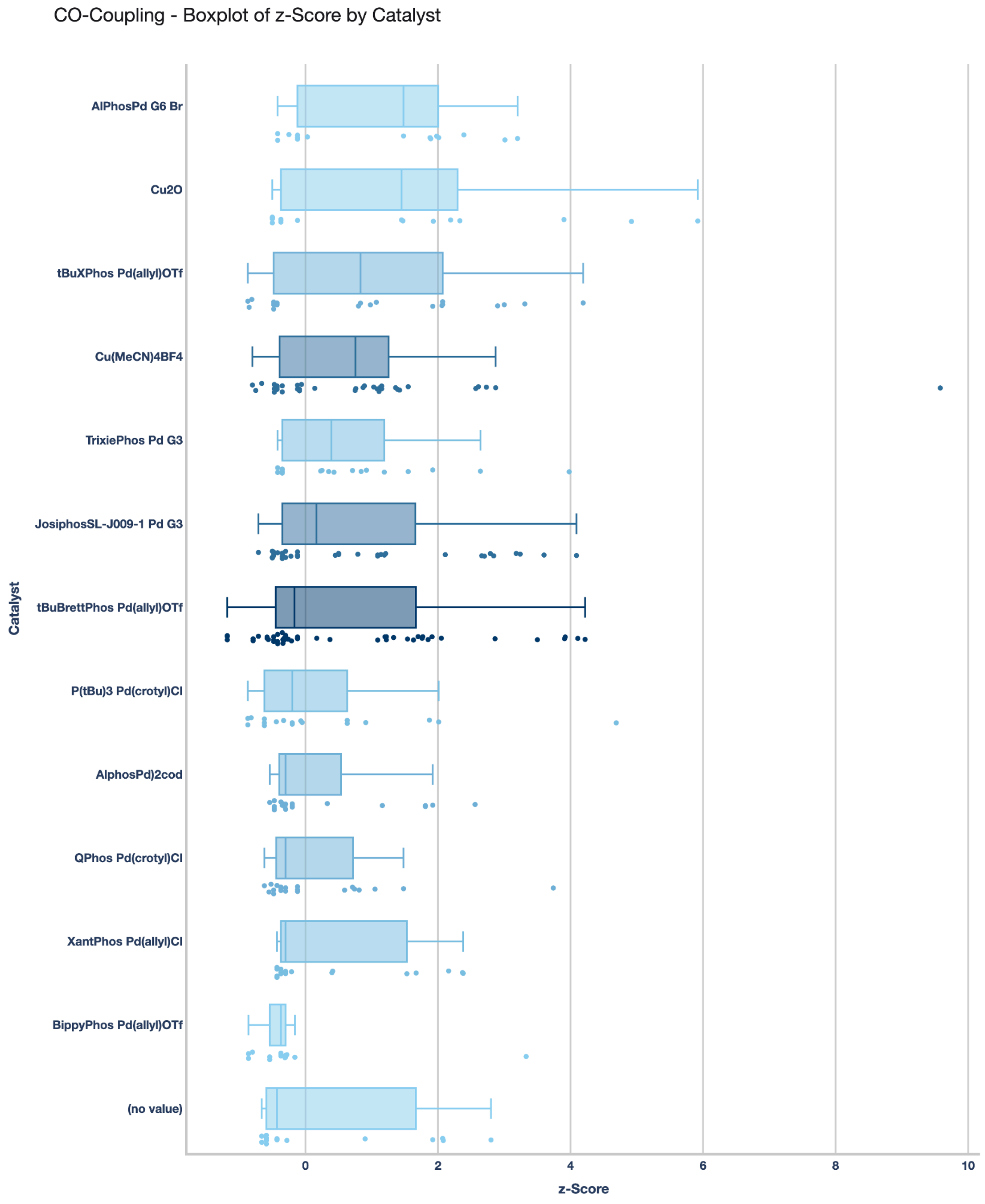

Figure S59: Catalyst boxplot for CO-Coupling reactions

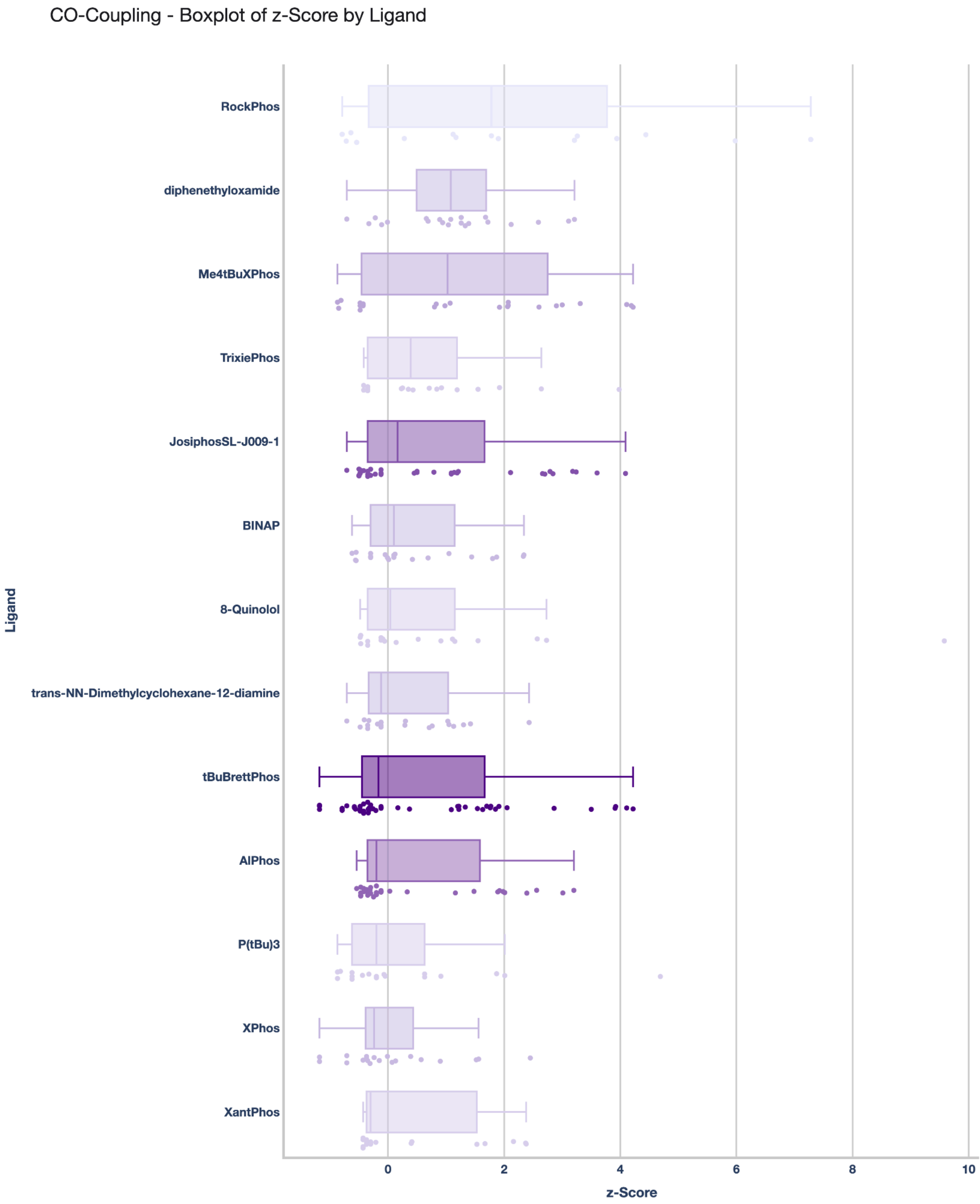

Figure S60: Ligand boxplot for CO-Coupling reactions

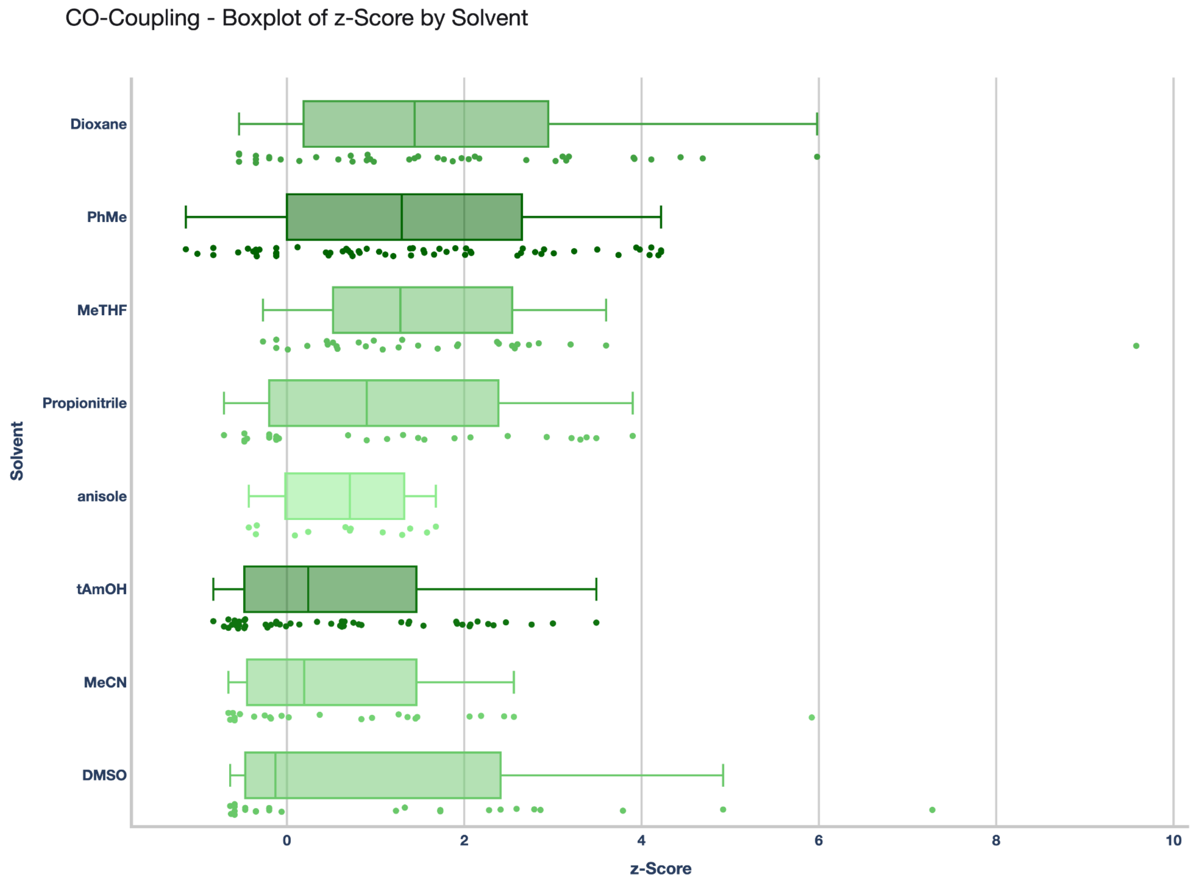

Figure S61: Solvent boxplot for CO-Coupling reactions

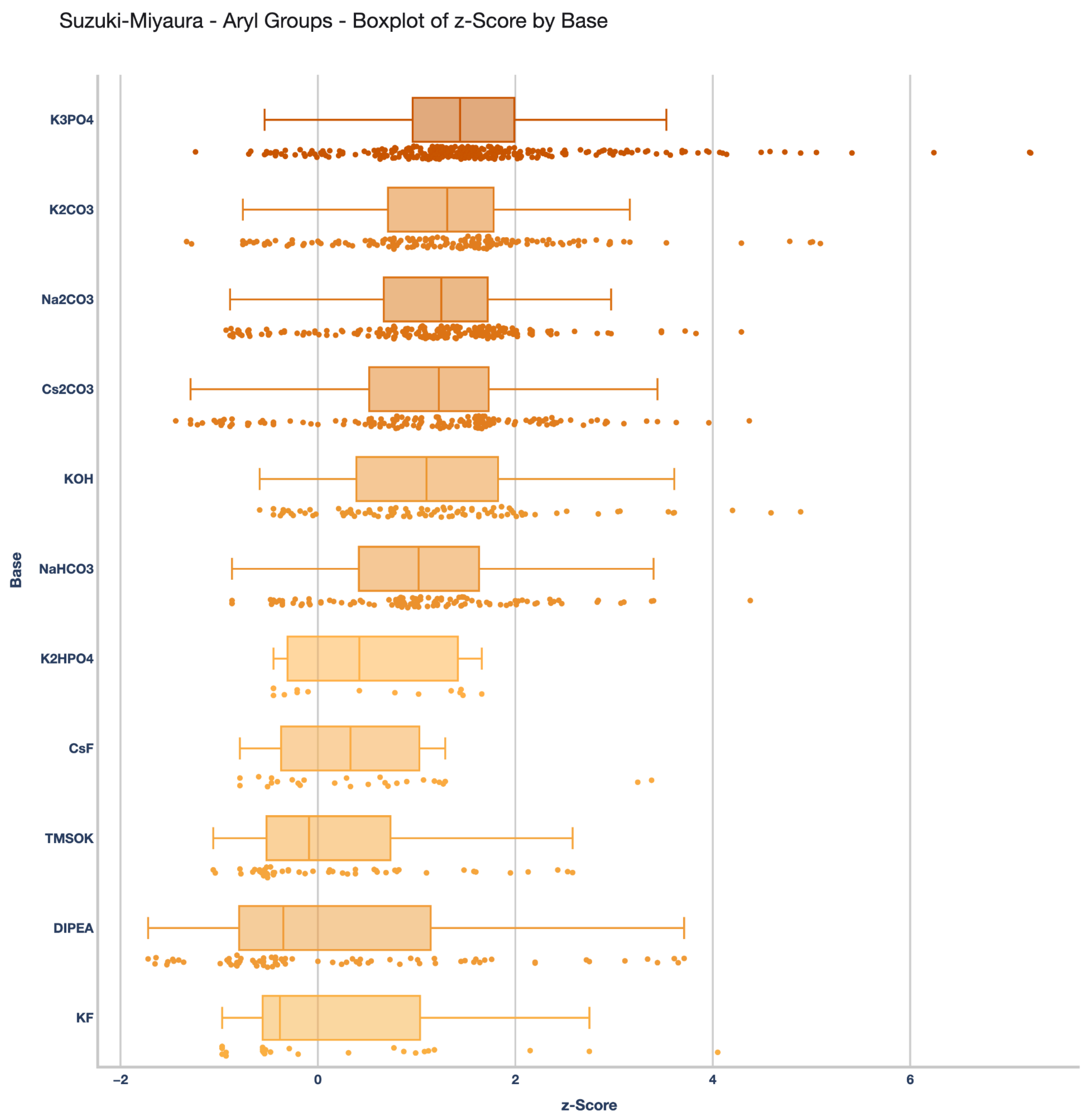

Figure S62: Base boxplot for Suzuki-Miyaura reactions for Aryl Groups

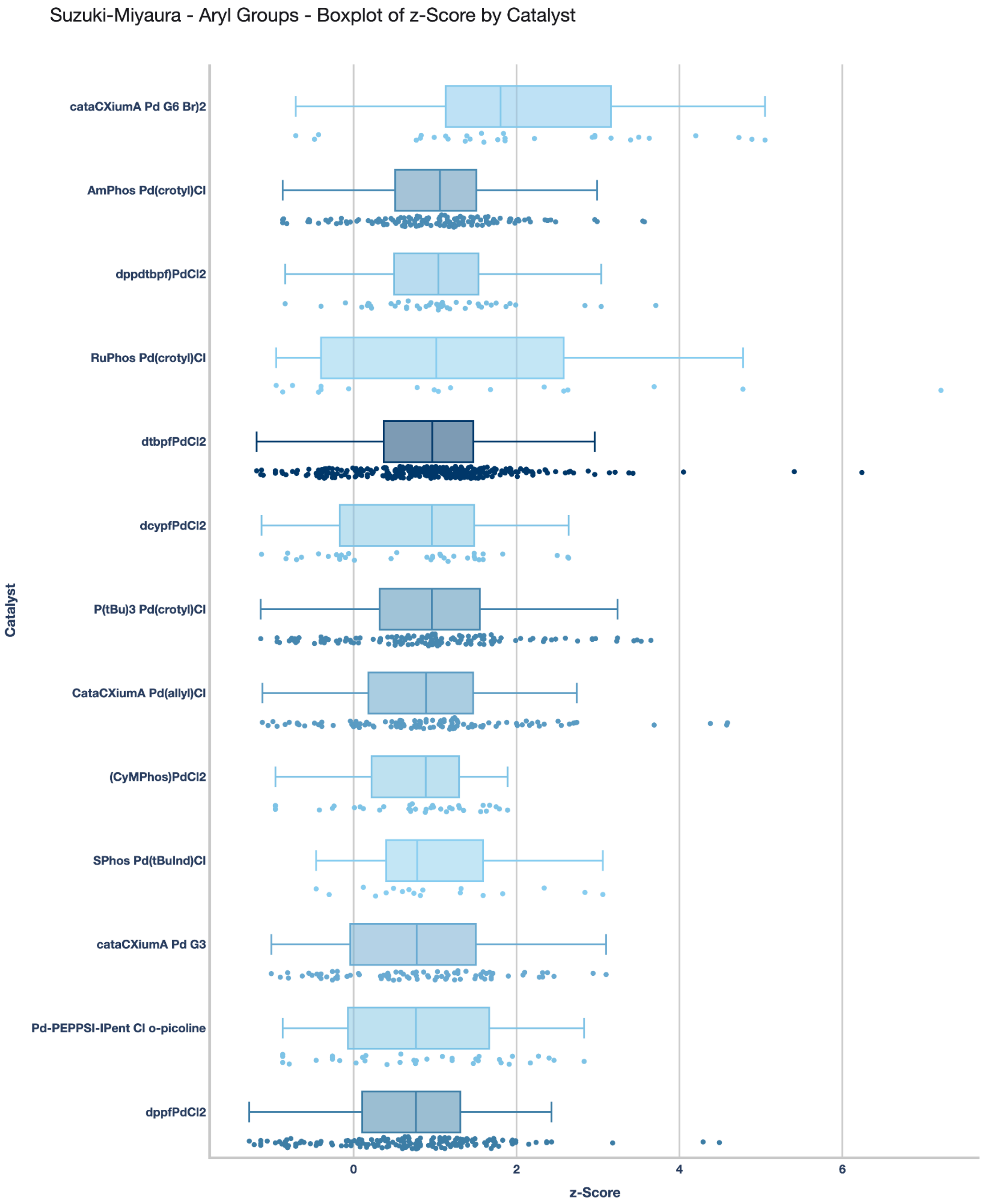

Figure S63: Catalyst boxplot for Suzuki-Miyaura reactions for Aryl Groups

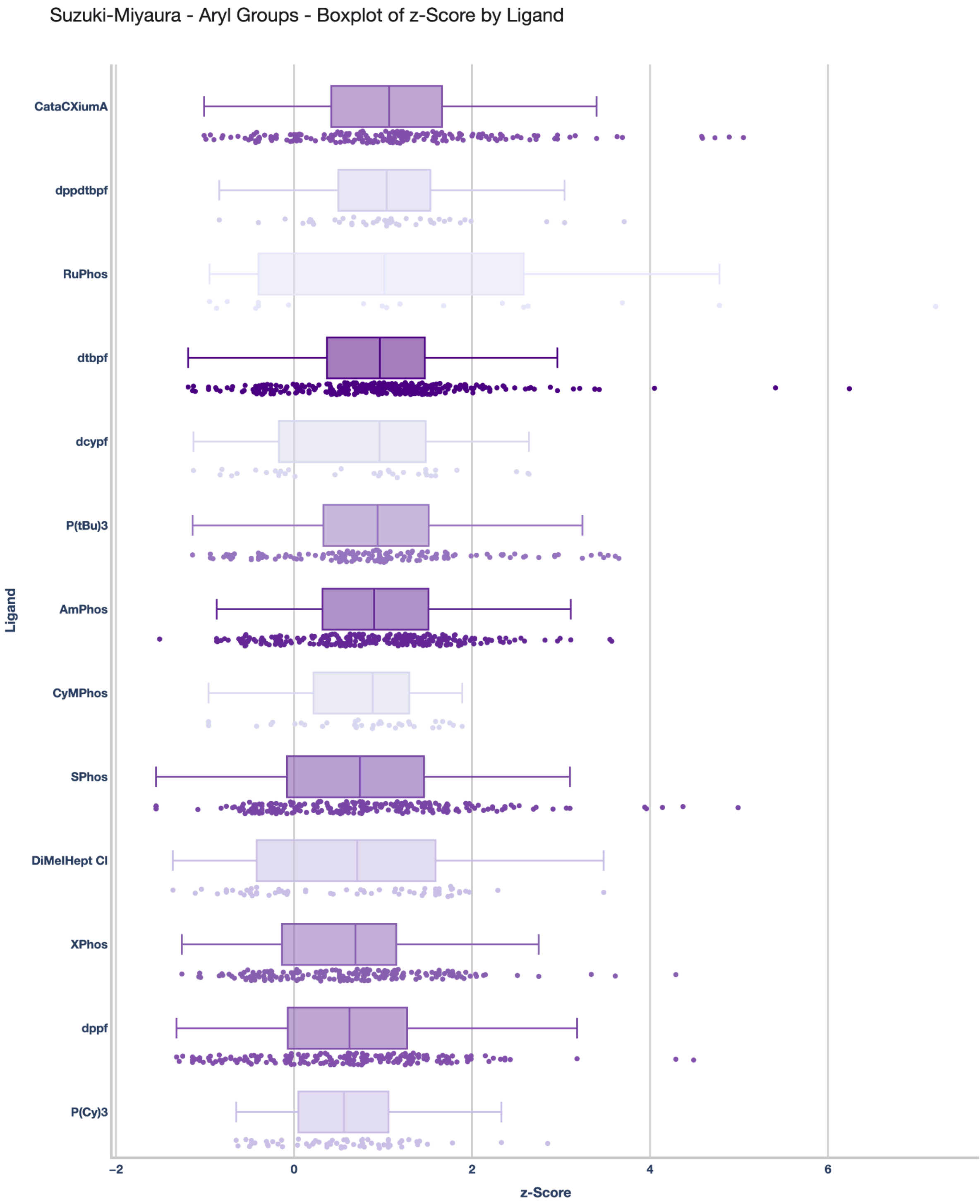

Figure S64: Ligand boxplot for Suzuki-Miyaura reactions for Aryl Groups

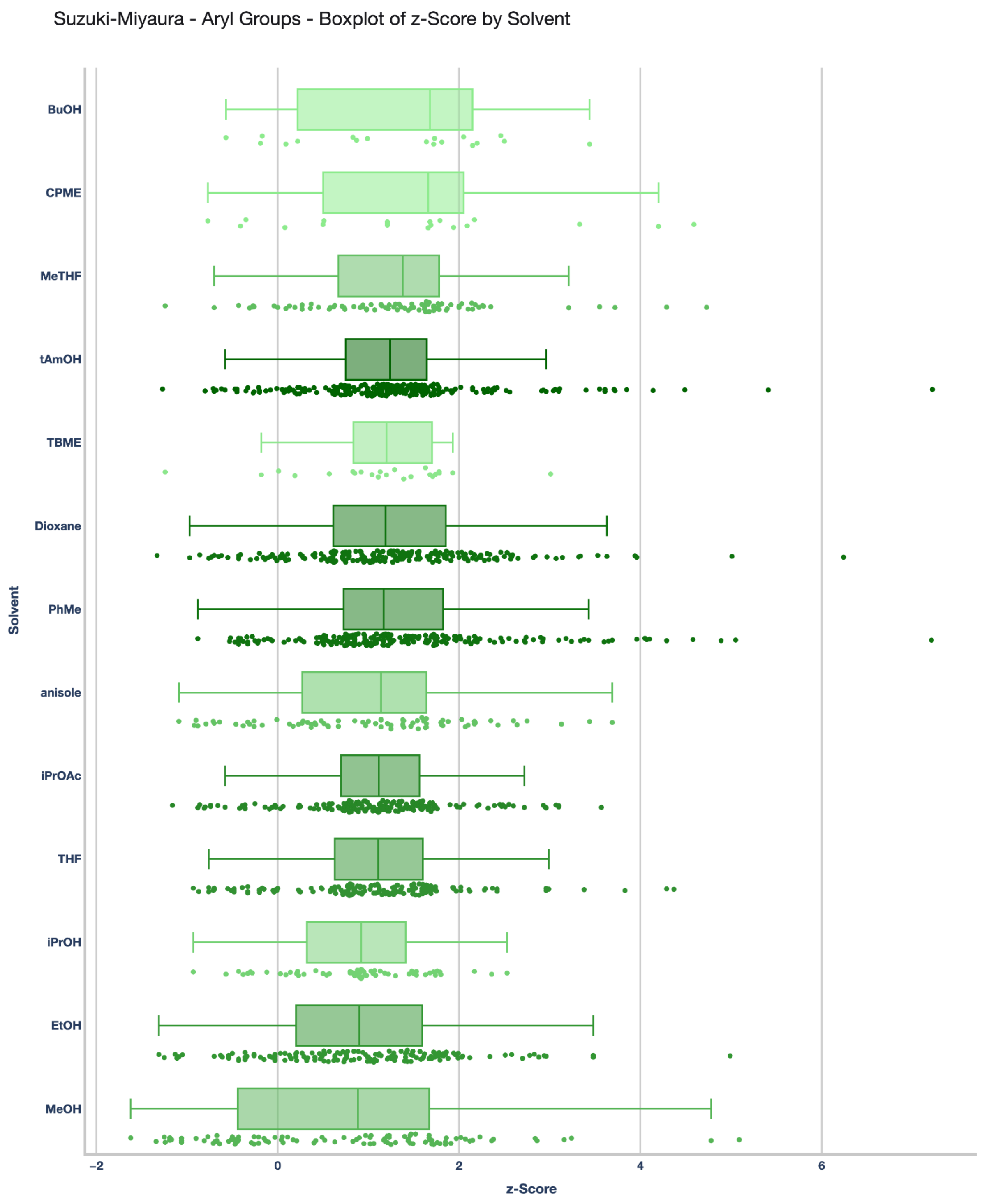

Figure S65: Solvent boxplot for Suzuki-Miyaura reactions for Aryl Groups

Limitations on Statistical Significance Testing

Standard statistical tests for comparing groups (e.g., Kruskal-Wallis, Mann-Whitney U) assume independent observations. Our data structure and all HTE data are in violation with this assumption by design. Multiple observations originate from the same experiment (ELN) and experiments are done iteratively, making them dependent. Therefore, we cannot apply standard statistical tests to assess the significance of differences between reagents, as this would yield misleading p-values. Instead, we present the full distribution in the boxplots, which convey uncertainty more honestly than a single p-value and allow visual assessment of overlap between reagent distributions. This reflects the reality that reagent performance depends heavily on the specific substrate pair.

Dataset Information per Reaction Type

The following definitions apply:

- **Reactions:** Represents the total number of chemical transformations for each specific reaction type.
- **Reagents:** Encompasses all components involved in the reaction, including additives, bases, catalysts, coupling reagents, ligands, and solvents.
- **Functional Groups:** Refers to the specific functional groups that react.
- **AREA_TOTAL_REDUCED:** This is the re-normalized peak area percentage, excluding peaks from the injection, reagents, or internal standards.

Total Reactions: 67,202
Reaction Types: 42

Buchwald-Hartwig
Reactions: 20,694
Reagents: 280 unique (median: 56.0 reactions/reagent)
Functional Groups: 18 unique
AREA_TOTAL_REDUCED: avg=19.65, std=27.06, skew=1.367, kurt=0.669
Shapiro-Wilk: W=0.7504, p=0.0000 → Not Normal (α=0.05)
Most frequent Reagents: Cs2CO3 (4227), NaOtBu (3743), K3PO4 (3437)
Most frequent FGs: ArBr (12229), ArCl (7265), RNH2 a-branch (4419)

Suzuki-Miyaura
Reactions: 11,594
Reagents: 172 unique (median: 52.0 reactions/reagent)
Functional Groups: 12 unique
AREA_TOTAL_REDUCED: avg=30.28, std=31.56, skew=0.770, kurt=-0.791
Shapiro-Wilk: W=0.8502, p=0.0000 → Not Normal (α=0.05)

Most frequent Reagents: K3PO4 (4093), Na2CO3 (2005), dtbpfPdCl2 (1589)
Most frequent FGs: ArBr (5866), ArB(OR)2 (5466), ArCl (4840)

Arylation, acidic C-H
Reactions: 4,152
Reagents: 134 unique (median: 40.0 reactions/reagent)
Functional Groups: 3 unique
AREA_TOTAL_REDUCED: avg=24.81, std=32.65, skew=1.107, kurt=-0.233
Shapiro-Wilk: W=0.7568, p=0.0000 → Not Normal (α=0.05)
Most frequent Reagents: Cs2CO3 (756), NEt3 (756), K3PO4 (540)
Most frequent FGs: Alkyl-H acidic (4152), ArBr (3960), Alkyl-Br (192)

Amide coupling
Reactions: 3,960
Reagents: 58 unique (median: 72.0 reactions/reagent)
Functional Groups: 7 unique
AREA_TOTAL_REDUCED: avg=35.98, std=32.30, skew=0.525, kurt=-1.094
Shapiro-Wilk: W=0.8896, p=0.0000 → Not Normal (α=0.05)
Most frequent Reagents: No Ligand (3960), DIC (1610), HOPO (872)
Most frequent FGs: RCO2H or M (3672), ArNH2 (1488), RNH2 a-branch (1056)

CN-Coupling
Reactions: 3,918
Reagents: 198 unique (median: 26.5 reactions/reagent)
Functional Groups: 16 unique
AREA_TOTAL_REDUCED: avg=20.94, std=26.44, skew=1.118, kurt=-0.019
Shapiro-Wilk: W=0.7879, p=0.0000 → Not Normal (α=0.05)
Most frequent Reagents: CuI (1451), Cs2CO3 (888),
trans-NN-Dimethylcyclohexane-12-diamine (819)
Most frequent FGs: ArBr (1926), ArI (1368), ROC(O)NR2 (816)

CO-Coupling
Reactions: 3,123
Reagents: 177 unique (median: 24.0 reactions/reagent)
Functional Groups: 6 unique
AREA_TOTAL_REDUCED: avg=13.31, std=20.54, skew=1.762, kurt=2.596
Shapiro-Wilk: W=0.7059, p=0.0000 → Not Normal (α=0.05)
Most frequent Reagents: CuI (641), Cs2CO3 (556), K3PO4 (454)
Most frequent FGs: ArBr (1899), ArOH (864), Alkyl-OH a-branch (768)

CH-Activation
Reactions: 2,240
Reagents: 126 unique (median: 20.0 reactions/reagent)
Functional Groups: 8 unique

AREA_TOTAL_REDUCED: avg=14.84, std=21.09, skew=1.766, kurt=2.750
Shapiro-Wilk: W=0.7398, p=0.0000 → Not Normal (α=0.05)
Most frequent Reagents: K2CO3 (780), XantPhos (566), KOPiv (556)
Most frequent FGs: ArH (1376), ArBr (744), ArI (488)

Condensation
Reactions: 2,220
Reagents: 73 unique (median: 24.0 reactions/reagent)
Functional Groups: 15 unique
AREA_TOTAL_REDUCED: avg=21.19, std=23.54, skew=1.023, kurt=0.025
Shapiro-Wilk: W=0.8432, p=0.0000 → Not Normal (α=0.05)
Most frequent Reagents: No Ligand (2210), water (300), NMM (202)
Most frequent FGs: RNH2 (942), ArNH2 (528), RCHO (462)

Negishi, in-situ
Reactions: 1,752
Reagents: 86 unique (median: 21.0 reactions/reagent)
Functional Groups: 4 unique
AREA_TOTAL_REDUCED: avg=14.54, std=18.95, skew=1.478, kurt=1.407
Shapiro-Wilk: W=0.7756, p=0.0000 → Not Normal (α=0.05)
Most frequent Reagents: TMEDA (944), Octanoic acid (420), AmPhos (338)
Most frequent FGs: ArBr (1656), Alkyl-Br (1104), Alkyl-I (408)

Cyclization
Reactions: 1,656
Reagents: 133 unique (median: 20.0 reactions/reagent)
Functional Groups: 10 unique
AREA_TOTAL_REDUCED: avg=19.55, std=26.47, skew=1.350, kurt=0.741
Shapiro-Wilk: W=0.7606, p=0.0000 → Not Normal (α=0.05)
Most frequent Reagents: No Ligand (1108), T3P (316), DIPEA (220)
Most frequent FGs: ArBr (792), RCO2H or M (312), alkyne (216)

Borylation, Miyaura
Reactions: 1,402
Reagents: 101 unique (median: 16.0 reactions/reagent)
Functional Groups: 1 unique
AREA_TOTAL_REDUCED: avg=15.49, std=22.75, skew=1.811, kurt=2.683
Shapiro-Wilk: W=0.7197, p=0.0000 → Not Normal (α=0.05)
Most frequent Reagents: Potassium 2-ethylhexanoate (644), KOAc (240), XPhos (240)
Most frequent FGs: ArBr (1402)

Suzuki-Miyaura, in situ
Reactions: 1,296

Reagents: 75 unique (median: 32.0 reactions/reagent)
Functional Groups: 3 unique
AREA_TOTAL_REDUCED: avg=15.82, std=22.52, skew=1.811, kurt=2.724
Shapiro-Wilk: W=0.7297, p=0.0000 → Not Normal (α=0.05)
Most frequent Reagents: Brij 35 (492), P(tBu)3 (422), P(tBu)3 Pd(crotyl)Cl (390)
Most frequent FGs: ArBr (1296), ArCl (912), alkene-I (96)

Alkylation
Reactions: 984
Reagents: 45 unique (median: 20.0 reactions/reagent)
Functional Groups: 11 unique
AREA_TOTAL_REDUCED: avg=36.93, std=36.79, skew=0.473, kurt=-1.371
Shapiro-Wilk: W=0.8377, p=0.0000 → Not Normal (α=0.05)
Most frequent Reagents: No Ligand (900), LiI (148), Cs2CO3 (132)
Most frequent FGs: Alkyl-OSO2R (480), arom. NH (480), Alkyl-Cl (240)

Deprotection
Reactions: 936
Reagents: 9 unique (median: 16.0 reactions/reagent)
Functional Groups: 2 unique
AREA_TOTAL_REDUCED: avg=49.07, std=35.81, skew=-0.169, kurt=-1.571
Shapiro-Wilk: W=0.8718, p=0.0000 → Not Normal (α=0.05)
Most frequent Reagents: No Ligand (936), TFE (104), HFIP (80)
Most frequent FGs: Protecting Group (840), R3N (96)

Negishi
Reactions: 840
Reagents: 98 unique (median: 16.0 reactions/reagent)
Functional Groups: 3 unique
AREA_TOTAL_REDUCED: avg=11.01, std=17.12, skew=2.279, kurt=5.336
Shapiro-Wilk: W=0.6808, p=0.0000 → Not Normal (α=0.05)
Most frequent Reagents: Potassium 2-ethylhexanoate (116), LiCl (106), No Ligand (90)
Most frequent FGs: Alkyl-M (456), ArBr (432), ArCl (408)

Heck
Reactions: 696
Reagents: 72 unique (median: 20.0 reactions/reagent)
Functional Groups: 3 unique
AREA_TOTAL_REDUCED: avg=10.00, std=12.37, skew=1.099, kurt=0.153
Shapiro-Wilk: W=0.8022, p=0.0000 → Not Normal (α=0.05)
Most frequent Reagents: TBAC (280), N-Methyldicyclohexylamin (256), TBD (200)
Most frequent FGs: alkene (696), ArCl (384), ArBr (312)

CC-Coupling
Reactions: 680
Reagents: 82 unique (median: 16.0 reactions/reagent)
Functional Groups: 4 unique
AREA_TOTAL_REDUCED: avg=16.39, std=20.75, skew=1.420, kurt=1.811
Shapiro-Wilk: W=0.7949, p=0.0000 → Not Normal (α=0.05)
Most frequent Reagents: K3PO4 (176), NiBr2-glyme (120), tBubpyCAMCN (120)
Most frequent FGs: ArCl (312), ArBr (272), RCO2R (272)

SNAr
Reactions: 648
Reagents: 46 unique (median: 12.0 reactions/reagent)
Functional Groups: 9 unique
AREA_TOTAL_REDUCED: avg=18.21, std=21.04, skew=1.201, kurt=1.101
Shapiro-Wilk: W=0.8319, p=0.0000 → Not Normal (α=0.05)
Most frequent Reagents: No Ligand (628), NaOtBu (144), DBU (100)
Most frequent FGs: ArF (384), Alkyl-OH a-branch (312), Lactam (216)

Hydrolysis
Reactions: 624
Reagents: 74 unique (median: 12.0 reactions/reagent)
Functional Groups: 3 unique
AREA_TOTAL_REDUCED: avg=13.23, std=24.39, skew=2.034, kurt=3.192
Shapiro-Wilk: W=0.6124, p=0.0000 → Not Normal (α=0.05)
Most frequent Reagents: Cs2CO3 (152), KOH (148), B(OH)3 (140)
Most frequent FGs: ArBr (408), RCN (120), ArCl (96)

Salt formation
Reactions: 396
Reagents: 1 unique (median: 396.0 reactions/reagent)
Functional Groups: 2 unique
AREA_TOTAL_REDUCED: avg=28.66, std=40.51, skew=0.816, kurt=-1.179
Shapiro-Wilk: W=0.6629, p=0.0000 → Not Normal (α=0.05)
Most frequent Reagents: No Ligand (396)
Most frequent FGs: RNH2 (216), R3N (180)

Sonogashira
Reactions: 369
Reagents: 50 unique (median: 18.0 reactions/reagent)
Functional Groups: 2 unique
AREA_TOTAL_REDUCED: avg=12.35, std=17.94, skew=1.536, kurt=1.522
Shapiro-Wilk: W=0.7314, p=0.0000 → Not Normal (α=0.05)
Most frequent Reagents: NEt3 (248), Cu(MeCN)4BF4 (157), CuI (112)
Most frequent FGs: alkyne (369), ArBr (369)

Stetter
Reactions: 288
Reagents: 23 unique (median: 24.0 reactions/reagent)
Functional Groups: 2 unique
AREA_TOTAL_REDUCED: avg=25.27, std=22.12, skew=0.786, kurt=0.094
Shapiro-Wilk: W=0.9156, p=0.0000 → Not Normal (α=0.05)
Most frequent Reagents: No Ligand (264), thiazolium-NHC*HCl (216), NEt3 (88)
Most frequent FGs: RCHO (288), alkene (288)

Oxidation
Reactions: 264
Reagents: 11 unique (median: 36.0 reactions/reagent)
Functional Groups: 2 unique
AREA_TOTAL_REDUCED: avg=31.90, std=30.06, skew=0.696, kurt=-0.695
Shapiro-Wilk: W=0.8847, p=0.0000 → Not Normal (α=0.05)
Most frequent Reagents: No Ligand (264), KOH (48), Brij 35 (48)
Most frequent FGs: Alkyl-H acidic (216), ArNH2 (48)

Cyanation
Reactions: 264
Reagents: 33 unique (median: 12.0 reactions/reagent)
Functional Groups: 1 unique
AREA_TOTAL_REDUCED: avg=20.19, std=29.42, skew=1.280, kurt=0.229
Shapiro-Wilk: W=0.7158, p=0.0000 → Not Normal (α=0.05)
Most frequent Reagents: CuI (108), Zn (84), XantPhos Pd(allyl)Cl (48)
Most frequent FGs: ArBr (264)

Activation
Reactions: 240
Reagents: 16 unique (median: 16.0 reactions/reagent)
Functional Groups: 1 unique
AREA_TOTAL_REDUCED: avg=11.93, std=18.90, skew=2.172, kurt=4.606
Shapiro-Wilk: W=0.6807, p=0.0000 → Not Normal (α=0.05)
Most frequent Reagents: No Ligand (240), PEP-K (120), TrichloroACN (64)
Most frequent FGs: Alkyl-OH a-branch (240)

Hydrodehalogenation
Reactions: 216
Reagents: 4 unique (median: 108.0 reactions/reagent)
Functional Groups: 1 unique
AREA_TOTAL_REDUCED: avg=15.48, std=20.16, skew=1.762, kurt=2.693
Shapiro-Wilk: W=0.7592, p=0.0000 → Not Normal (α=0.05)
Most frequent Reagents: Pd/C (120), Missing (120), No Ligand (96)

Most frequent FGs: ArCl (216)

Glycosidation
Reactions: 192
Reagents: 1 unique (median: 192.0 reactions/reagent)
Functional Groups: 2 unique
AREA_TOTAL_REDUCED: avg=37.06, std=21.93, skew=0.386, kurt=-0.888
Shapiro-Wilk: W=0.9555, p=0.0000 → Not Normal (α=0.05)
Most frequent Reagents: No Ligand (192)
Most frequent FGs: RCO2R (192), Alkyl-OH primary (192)

Stannylation
Reactions: 192
Reagents: 21 unique (median: 24.0 reactions/reagent)
Functional Groups: 2 unique
AREA_TOTAL_REDUCED: avg=9.20, std=17.07, skew=2.493, kurt=6.496
Shapiro-Wilk: W=0.5992, p=0.0000 → Not Normal (α=0.05)
Most frequent Reagents: LiCl (32), CsF (32), KOAc (32)
Most frequent FGs: ArBr (192), RSnR3 (192)

Dimerization, reductive
Reactions: 192
Reagents: 24 unique (median: 16.0 reactions/reagent)
Functional Groups: 1 unique
AREA_TOTAL_REDUCED: avg=10.91, std=11.89, skew=0.963, kurt=0.073
Shapiro-Wilk: W=0.8519, p=0.0000 → Not Normal (α=0.05)
Most frequent Reagents: Potassium 2-ethylhexanoate (80), meCgPPh Pd G3 (80), meCgPPh (80)
Most frequent FGs: ArOSO2R (192)

Dehydration
Reactions: 192
Reagents: 19 unique (median: 16.0 reactions/reagent)
Functional Groups: 1 unique
AREA_TOTAL_REDUCED: avg=4.80, std=9.08, skew=3.095, kurt=11.935
Shapiro-Wilk: W=0.5895, p=0.0000 → Not Normal (α=0.05)
Most frequent Reagents: No Ligand (192), DBU (40), PyBrOP (36)
Most frequent FGs: RCONHR (192)

Borylation, C-H
Reactions: 120
Reagents: 16 unique (median: 16.0 reactions/reagent)
Functional Groups: 1 unique
AREA_TOTAL_REDUCED: avg=6.24, std=10.86, skew=2.011, kurt=3.485

Shapiro-Wilk: W=0.6326, p=0.0000 → Not Normal (α=0.05)
Most frequent Reagents: [Ir(cod)OMe]2 (72), [Ir(cod)Cl]2 (48), dtbpy (20)
Most frequent FGs: ArH (120)

CS-Coupling
Reactions: 120
Reagents: 35 unique (median: 6.0 reactions/reagent)
Functional Groups: 3 unique
AREA_TOTAL_REDUCED: avg=28.21, std=25.69, skew=0.484, kurt=-0.808
Shapiro-Wilk: W=0.8947, p=0.0000 → Not Normal (α=0.05)
Most frequent Reagents: K2S2O5 (72), Sodium formate (66), TBAB (60)
Most frequent FGs: ArBr (96), ArI (24), RSH (24)

Mitsunobu
Reactions: 120
Reagents: 10 unique (median: 12.0 reactions/reagent)
Functional Groups: 1 unique
AREA_TOTAL_REDUCED: avg=4.97, std=14.67, skew=3.822, kurt=14.696
Shapiro-Wilk: W=0.3855, p=0.0000 → Not Normal (α=0.05)
Most frequent Reagents: No Ligand (96), DIPEA (48), POCl3 (16)
Most frequent FGs: ArOH (120)

Wittig
Reactions: 118
Reagents: 6 unique (median: 28.0 reactions/reagent)
Functional Groups: 2 unique
AREA_TOTAL_REDUCED: avg=11.35, std=16.58, skew=1.095, kurt=-0.457
Shapiro-Wilk: W=0.6952, p=0.0000 → Not Normal (α=0.05)
Most frequent Reagents: No Ligand (118), KOtPent (32), LiHMDS (30)
Most frequent FGs: RCH2PPh3X (118), R2CO (118)

Hydration
Reactions: 96
Reagents: 7 unique (median: 12.0 reactions/reagent)
Functional Groups: 1 unique
AREA_TOTAL_REDUCED: avg=7.18, std=13.93, skew=3.300, kurt=12.252
Shapiro-Wilk: W=0.5605, p=0.0000 → Not Normal (α=0.05)
Most frequent Reagents: No Ligand (96), Na2CO3 (48), TBAHS (32)
Most frequent FGs: RCN (96)

Addition
Reactions: 96
Reagents: 13 unique (median: 8.0 reactions/reagent)
Functional Groups: 2 unique

AREA_TOTAL_REDUCED: avg=4.21, std=10.42, skew=3.054, kurt=8.810
Shapiro-Wilk: W=0.4640, p=0.0000 → Not Normal (α=0.05)
Most frequent Reagents: No Ligand (96), DMAP (8), KOAc (8)
Most frequent FGs: Alkyl-H acidic (96), RCN (96)

Sandmeyer
Reactions: 96
Reagents: 7 unique (median: 24.0 reactions/reagent)
Functional Groups: 1 unique
AREA_TOTAL_REDUCED: avg=1.18, std=6.15, skew=5.452, kurt=29.217
Shapiro-Wilk: W=0.1913, p=0.0000 → Not Normal (α=0.05)
Most frequent Reagents: amyl nitrite (96), No Ligand (96), CuF2 (32)
Most frequent FGs: ArNH2 (96)

Reduction
Reactions: 48
Reagents: 13 unique (median: 6.0 reactions/reagent)
Functional Groups: 2 unique
AREA_TOTAL_REDUCED: avg=69.13, std=27.77, skew=-1.119, kurt=0.466
Shapiro-Wilk: W=0.8595, p=0.0000 → Not Normal (α=0.05)
Most frequent Reagents: Strykers reagent (24), tAmOH (24), AcOH (24)
Most frequent FGs: alkene (24), arene (24)

Deoxyfluorination
Reactions: 48
Reagents: 3 unique (median: 32.0 reactions/reagent)
Functional Groups: 1 unique
AREA_TOTAL_REDUCED: avg=45.07, std=27.81, skew=-0.301, kurt=-1.018
Shapiro-Wilk: W=0.9236, p=0.0040 → Not Normal (α=0.05)
Most frequent Reagents: No Ligand (48), Et3N.3HF (32), DBU (8)
Most frequent FGs: Alkyl-OH a-branch (48)

Fluorination, oxidative
Reactions: 48
Reagents: 5 unique (median: 6.0 reactions/reagent)
Functional Groups: 2 unique
AREA_TOTAL_REDUCED: avg=4.08, std=11.18, skew=5.980, kurt=36.596
Shapiro-Wilk: W=0.3204, p=0.0000 → Not Normal (α=0.05)
Most frequent Reagents: No Ligand (48), py.HF (16), CsF (6)
Most frequent FGs: ArH (24), ROCSSR (24)

Chlorination
Reactions: 48
Reagents: 5 unique (median: 8.0 reactions/reagent)

Functional Groups: 1 unique
AREA_TOTAL_REDUCED: avg=19.38, std=17.38, skew=0.492, kurt=-0.632
Shapiro-Wilk: W=0.9032, p=0.0008 → Not Normal (α=0.05)
Most frequent Reagents: No Ligand (48), POCl3 (42), NMM (8)
Most frequent FGs: RCO2R (48)

Protection
Reactions: 24
Reagents: 8 unique (median: 6.0 reactions/reagent)
Functional Groups: 1 unique
AREA_TOTAL_REDUCED: avg=4.84, std=12.31, skew=2.733, kurt=6.499
Shapiro-Wilk: W=0.4543, p=0.0000 → Not Normal (α=0.05)
Most frequent Reagents: PMBCl (24), No Ligand (24), DIPEA (6)
Most frequent FGs: ArOH (24)

Underlying Distribution Histograms

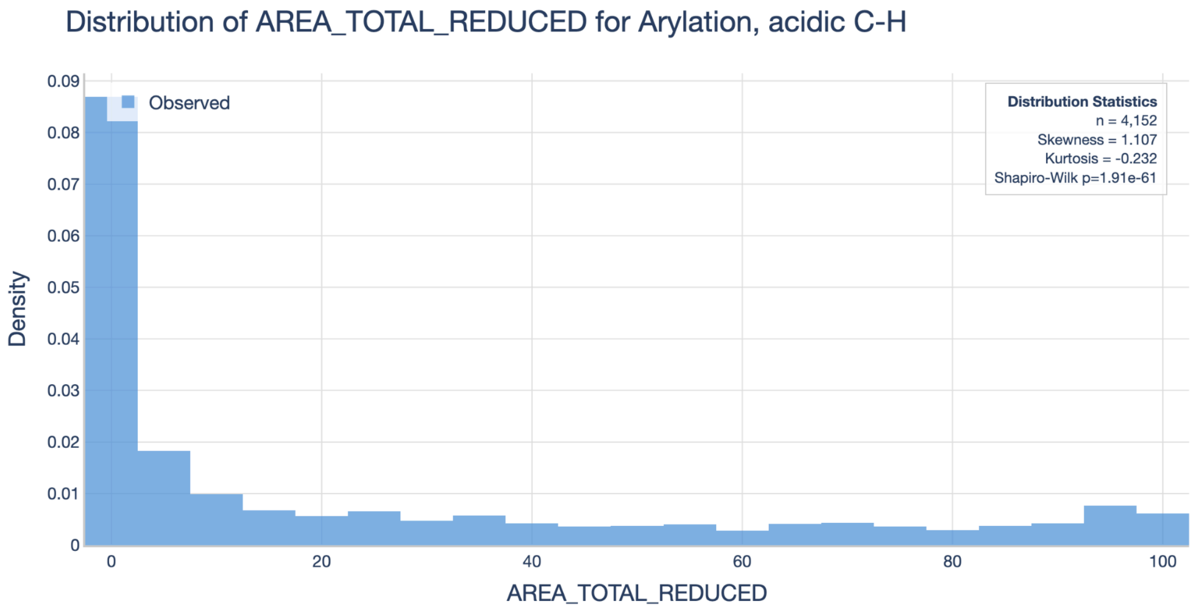

Figure S66: Histogram of the underlying distribution of AREA_TOTAL_REDUCED for Arylation, acidic C-H

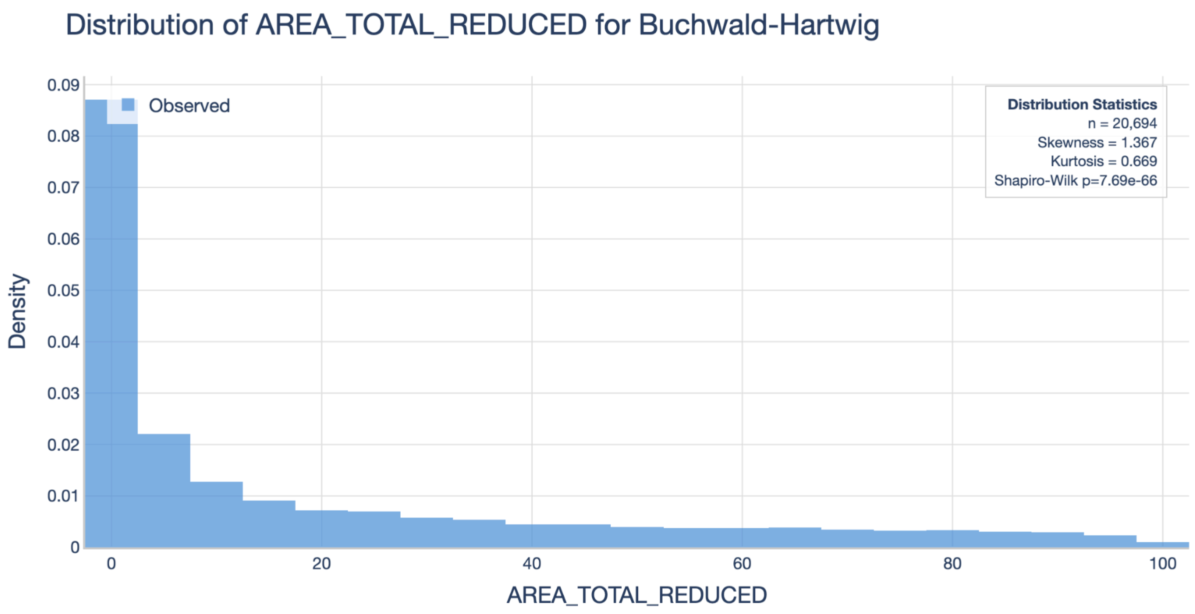

Figure S67: Histogram of the underlying distribution of AREA_TOTAL_REDUCED for Buchwald-Hartwig

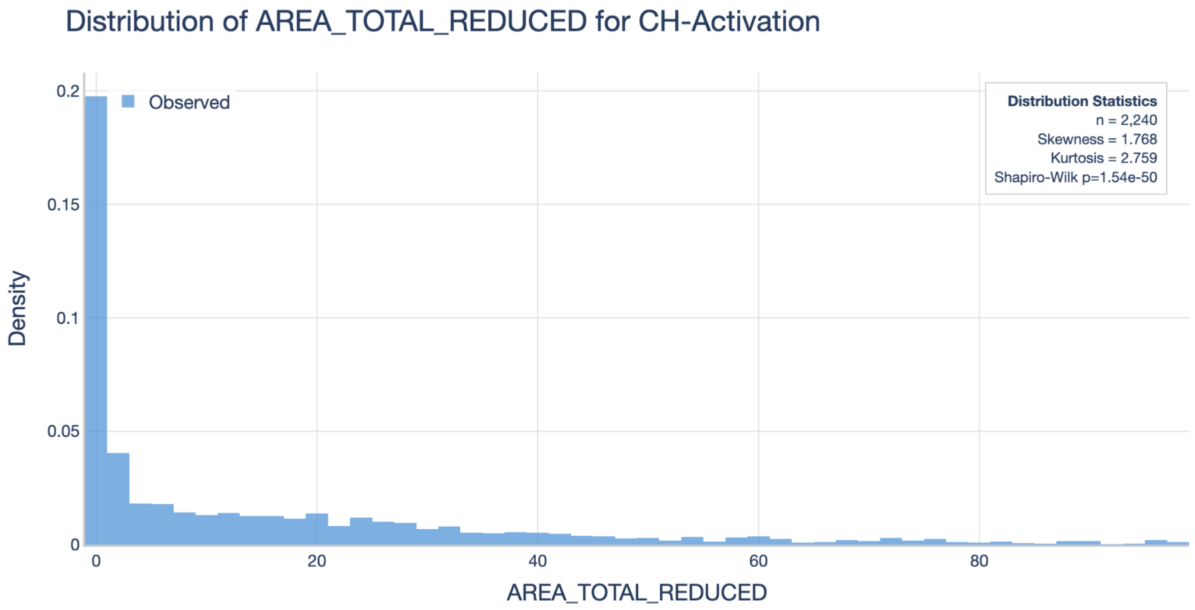

Figure S68: Histogram of the underlying distribution of AREA_TOTAL_REDUCED for CH-Activation

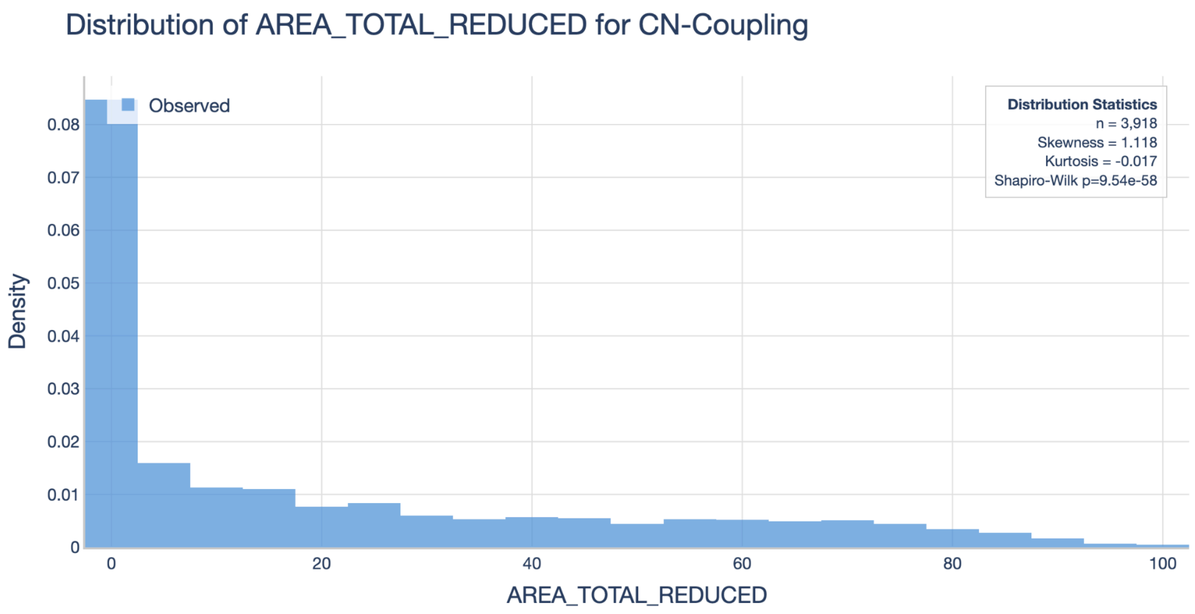

Figure S69: Histogram of the underlying distribution of AREA_TOTAL_REDUCED for CN-Coupling

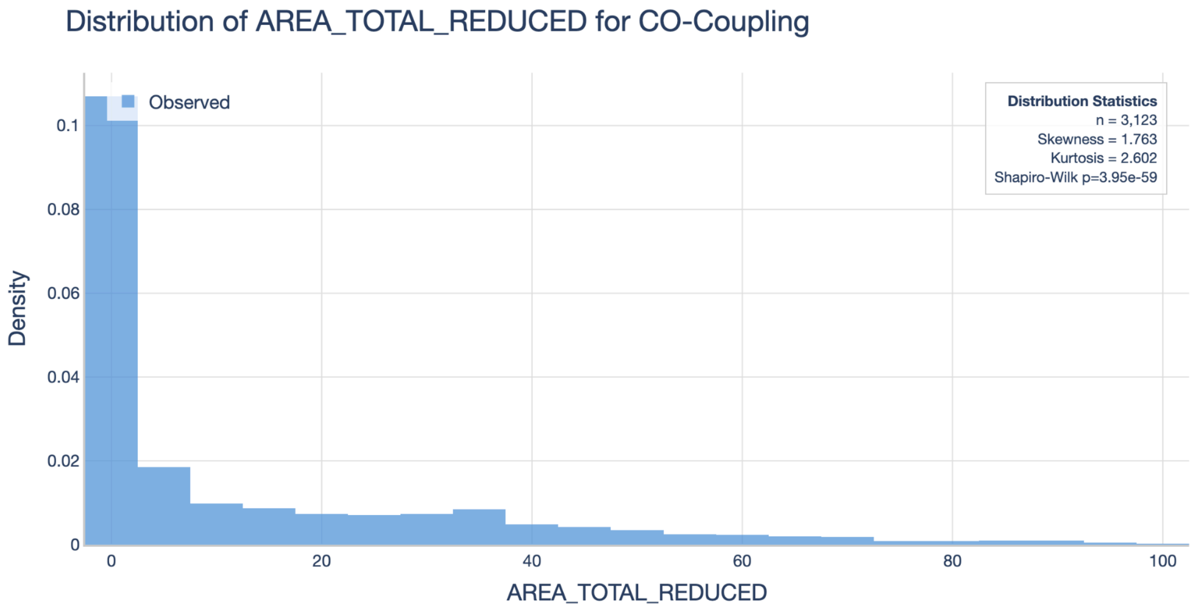

Figure S70: Histogram of the underlying distribution of AREA_TOTAL_REDUCED for CO-Coupling

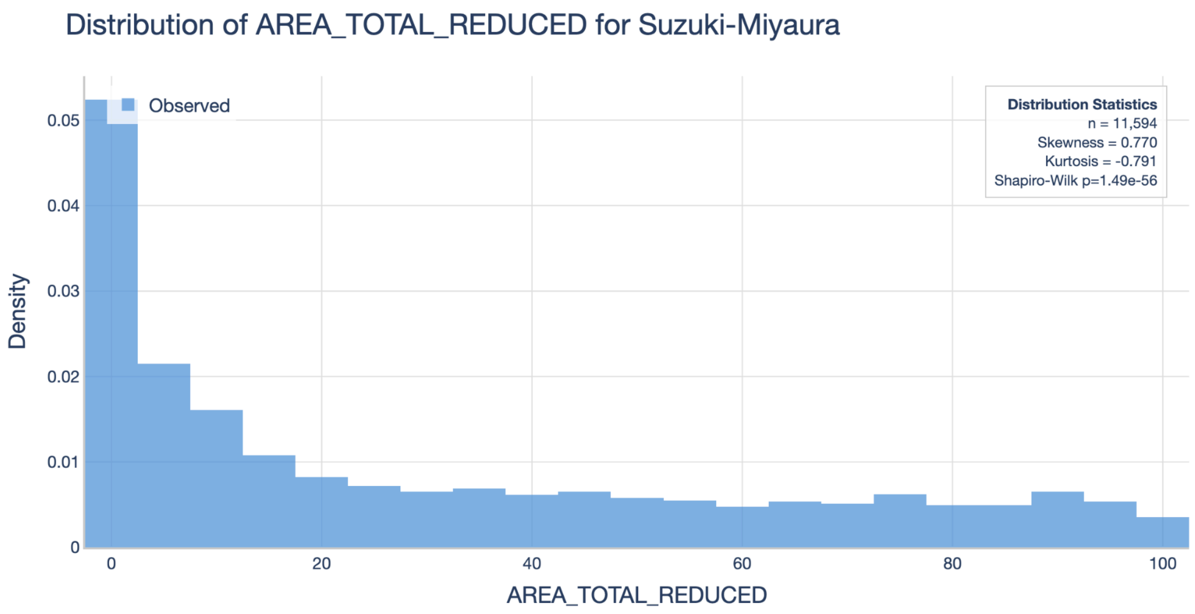

Figure S71: Histogram of the underlying distribution of AREA_TOTAL_REDUCED for Suzuki-Miyaura

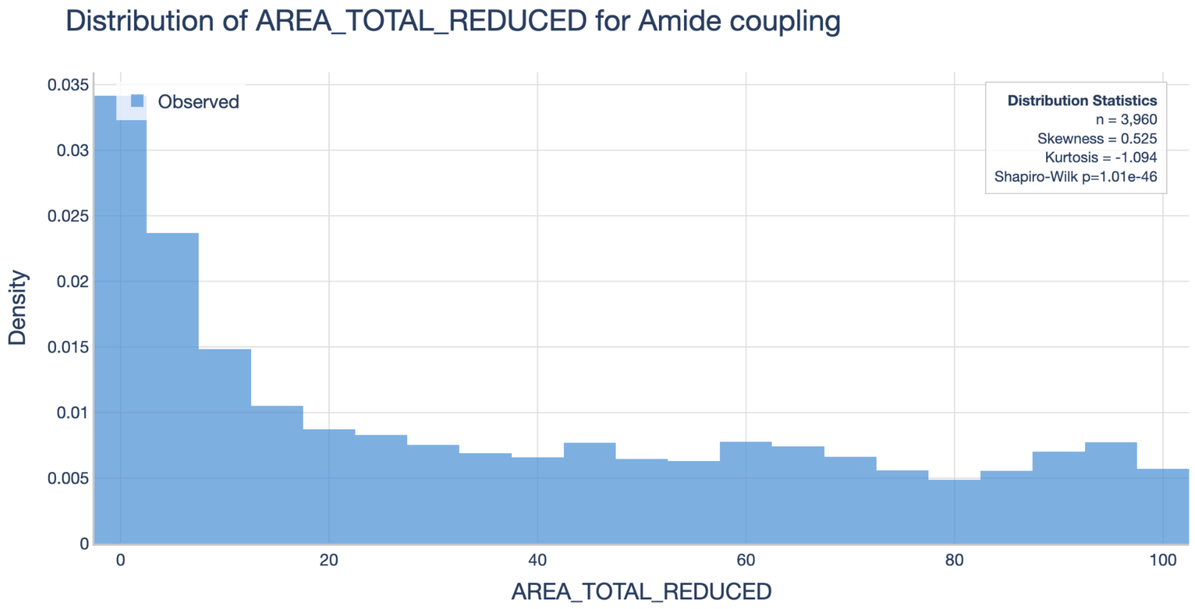

Figure S72: Histogram of the underlying distribution of AREA_TOTAL_REDUCED for Amide coupling

References

(1) Wuitschik, G.; Jost, V.; Schindler, T.; Jakubik, M. HTE OS: A High-Throughput Experimentation Workflow Built from the Ground Up. *Org. Proc. Res. Dev.* **2024**, *28*, 2875–2884. https://doi.org/10.1021/acs.oprd.4c00160.
(2) *Rdkit: 2024_03_3*; 2024.
(3) Rinehart, N. I.; Saunthwal, R. K.; Wellauer, J.; Zahrt, A. F.; Schlemper, L.; Shved, A. S.; Bigler, R.; Fantasia, S.; Denmark, S. E. A Machine-Learning Tool to Predict Substrate-Adaptive Conditions for Pd-Catalyzed C–N Couplings. *Science* **2023**, *381*, 965–972. https://doi.org/10.1126/science.adg2114.
