# Supplementary material for: Which Reaction Conditions Work on Drug-Like Molecules? Lessons from 66,000 High-Throughput Experiments
Source: ACS Cent Sci. 2026 Feb 5;12(2):222–32. doi: 10.1021/acscentsci.5c02031 (PMC12947288; doi:10.1021/acscentsci.5c02031)
Supplement: Supplementary file 4 [file oc5c02031_si_004.pdf]

oc-2025-02031g.R1

Name: Peer Review Information for "Which Reaction Conditions Work on Drug-Like Molecules? Lessons from 66,000 High-Throughput Experiments"

First Round of Reviewer Comments

Reviewer: 1

Comments to the Author

This study presents a z-score-based statistical analysis method derived from 66,000 high-throughput experiments to recommend optimal reagents for complex drug-like molecules under specific reaction conditions, demonstrating notable innovation and practical value. The public release of the dataset and interactive tool provides data-driven decision support for reaction optimization in chemical synthesis. However, the paper exhibits significant shortcomings in method validation, data quality assessment, bias control, and the generalizability of result interpretation, which undermine the reliability and broader applicability of its conclusions. Furthermore, the discussion insufficiently addresses the limitations of the z-score method, lacks comparative analysis with machine learning approaches, and does not adequately demonstrate the method's applicability beyond Roche's internal datasets. Therefore, it is recommended that the authors supplement with relevant experiments, deepen the methodological discussion, enhance data transparency, and conduct thorough external validation before consideration for acceptance.

1. The reliance on LC-MS peak area percentages for quantifying reaction products, rather than isolated yields, somewhat undermines the accuracy and comparability of the data. While the authors justify this approach due to the lack of standards and high-throughput needs, they do not sufficiently discuss the systematic errors introduced, such as variations in response factors between different compounds or the impact of unintegrated side products. It is recommended to supplement with validation experiments on representative reactions, for instance, by performing a correlation analysis between actual isolated yields and peak area percentages, to demonstrate the reliability of this proxy metric. Without this, readers may question the practical guidance value of the z-score rankings.

2. The dataset exhibits significant selection bias, primarily comprising "difficult reactions" and "complex molecules from drug discovery," which means the recommendations may not be suitable for routine or simpler systems. Although the authors mention this bias in the discussion, they do not assess its specific impact on the generalizability of their conclusions. It is suggested to add a dedicated section discussing how this bias affects the recommendations for different reaction types and to attempt validation on public datasets or standard reactions to illustrate the method's applicability and limitations across a broader chemical space.

3. While the z-score method is simple and user-friendly, its statistical efficacy depends on the normality and homogeneity of the data distribution. The paper does not adequately test these distributional characteristics of the reaction data. If the data are severely skewed or contain multiple clusters, the z-score may not accurately reflect the true performance of reagents. The authors should supplement with descriptions of the data distributions for different reaction types (e.g., using histograms or Q-Q plots) and discuss whether more robust statistical measures (like Median Absolute Deviation) should be employed for correction in cases of non-normal distributions.

4. The paper repeatedly emphasizes the superiority of this method over literature recommendations or machine learning methods but lacks direct, quantitative comparative evidence. For instance, there are no parallel experimental validations comparing the z-score recommended optimal conditions against classic literature conditions on the same substrates, nor is there a systematic comparison with existing predictive models (e.g., descriptor-based or reaction-based machine learning). It is recommended to design a set of control experiments comparing the actual reaction performance under conditions recommended by different methods to strengthen the persuasiveness of the paper's claims.

5. The authors mention that "users can filter reagents" through the tool but do not specify whether the tool supports user-uploaded data or the addition of new reaction types, which limits its general utility and long-term value. Furthermore, the algorithmic details behind the "recommendations" (e.g., the rationale for the default choice of 'n' in "top n z-scores") are not sufficiently explained, potentially affecting user trust in the results. It is advised to provide more detailed parameter tuning guidance in the Methods section or Supplementary Information and consider developing features that support user data import.

6. When discussing catalyst performance variations, the authors attribute some results to "batch quality differences" or "selection bias" but do not explain how these factors were identified or controlled. As the activity of catalysts from different batches can significantly

impact reaction outcomes, it is recommended to introduce quality control metrics during data collection (e.g., catalyst purity, batch numbers) and incorporate them as covariates in the analysis to improve the rigor of the conclusions.

7. The paper lacks discussion on the reaction scale effect. High-throughput experiments are typically conducted on microgram to milligram scales, whereas practical synthesis often occurs on gram scales. Differences in mixing efficiency, heat transfer, catalyst loading, etc., between these scales may affect the direct transferability of conditions. The authors should supplement the discussion with considerations of the scale effect and, if possible, provide scale-up validation results for some reactions.

8. Although the authors provide a public dataset and online tool, the lack of substrate structure information severely limits its value for machine learning or more detailed structure-activity relationship studies. While confidentiality concerns are understandable, it is recommended to provide descriptors of key structural features of the substrates (e.g., number of functional groups, polarity, steric hindrance) to help readers better understand the impact of "drug molecule complexity" on reaction condition selection and to facilitate transfer learning or model development by other researchers.

Reviewer: 2

Comments to the Author

The authors have conducted a retrospective statistical analysis of 66,000 reactions from High Throughput Screenings for reaction conditions of various transformations performed in their facility, with the goal of creating a tool that suggests the most promising reagents for a given transformation of certain educts. After analyzing the results of the reactions via LC-MS, they used a normalized area% of the transformation’s product to calculate a z-score for each experiment, stating how many standard deviations the value is away from the mean product area% of all experiments of the respective transformation. They then built an analysis tool that combines the top results for a certain reagent in each transformation for all transformations in a class of educts and reaction type to identify the best-performing reagent for this combination. They offer an interactive implementation of their tool, combined with the underlying dataset, as a GitHub repository and as a hosted web application. In the manuscript, they then discuss the results for the two most prevalent reaction types of their dataset: Buchwald-Hartwig and Suzuki-Miyaura cross-

coupling reactions. They compare the results with those of a similar study based on literature-reported Buchwald-Hartwig reaction conditions which they have published before. They found that the optimal reagents as determined retrospectively from their HTE differ from the optimal reagents according to the literature, which they explain by the bias of their selection of educts towards “difficult reactions” of highly functionalized building blocks, while literature contains more examples for basic transformations.

The manuscript is generally very well written, the data and analysis seems to be sound and the goal of the study is highly relevant. A tool that suggests optimal reagents based on experimental data from thousands of reactions performed under standardized conditions in the same lab is very valuable to the community, as the research for optimal conditions in the literature always suffers from limited comparability between labs and publications.

The following points need attention:

1. The figures and captions need to be improved.

a. In my opinion, Figure 1 does not meet the standards of professionalism that I expect from a journal such as ACS Central Science. The resolution is also too low. Additionally, it might be AI-generated. Unless it was drawn by one of the authors, I would expect crediting the source or AI tool it was created with.

b. The Boxplots

i. Standard box plots only show the outliers as data points, deviations from the standard format should be explained in the caption.

ii. The color gradient is not explained and is misleading, as it draws away attention from the best performing ligand. It is not immediately obvious that ligands are sorted by performance from top to bottom and the color gradient marks the number of data points. Both should be explained in the caption.

iii. The axis labels are much too small to be readable and serif fonts should be avoided in figures & graphs. A vector-based format would improve resolution.

iv. Figure 6 lacks an x-axis

v. Can the authors make any statements on the statistical significance of your results, e.g. the differences between reagents? It looks like the datapoints spread very far and the medians are very close to each other for almost all ligands shown.

c. The decision tree (Fig. 5)

i. The text of the figure is too small in comparison with the size of the figure, while there is a lot of blank space. Also, the text is very pixelated, a vector-based graphics format would be more suitable. Figure 3b) of the previous work (Fitzner et al., 26) is a good example of how it could be improved

ii. In the "Legend" box, the abbreviation ELN is used as a label and it is also very prevalent in the webtool, but it is never explained what ELN stands for. The whole notation scheme {1. name (#)z (#)ELN} looks quite crowded at this small font size and could be formatted clearer. The two labels "No Recommendation ..." and "Recommended ..." are in different font sizes.

iii. In the box for primary anilines, Ad-BrettPhos is written with a hyphen, while it is written together in line 24 on page 6. The last entry in the same box (Triisobutylphosphatrane) is misaligned after the line break

d. The caption of Supplementary Figure 1 lacks an explanation for the yellow and blue backgrounds of some of the ligands. Also, it seems to be adapted from a different source, which means there should be a statement in the caption where this figure was published first and whether the authors obtained permission to reproduce the figure in this work.

2. For publication in a Diamond Open Access Journal, I expect full transparency and publication of all underlying data that is necessary to comprehend and use the results of the work. I fully understand that the authors cannot release detailed structures of compounds from the company or clients. However, as the authors state, the conditions found to be optimal are most likely highly dependent on the substrate structure, as the literature contains different optima for simpler substrates, compared to the highly functionalized substrates of the "difficult reactions" in the data set. This means the tool is probably of very limited use if it can only be queried by broad subcategories like "ArNH2".

a. Would it be possible to include a structure search in the online tool, so that the user can enter their structure of interest and the tool adjusts the results to be actually fitting for the user's structure (for example by filtering or weighing the results included in the output by how similar the substrates were to the user's input), without revealing the actual structures of the molecules in the dataset?

b. On page 12, line 52 the authors mention sampling at three different time/temperature points. Why are neither temperature nor time reported with the conditions? As these factors can be crucial for a successful transformation, it would be nice to have a similar analysis as for the other factors, or at least a statement of the respective temperature and time in the box that shows when you hover over a data point in your webtool.

c. On page 5, line 20 the authors state that the internal tool has more advanced features. While this is totally understandable from an economic point of view for the company, I don't think it is a good statement to make in this paper. What does the user get from it, except the feeling of "not getting the full picture"?

3. The authors state that they limit the number of z-scores to be included per category and transformation, because they only want to look at optima. However, there are many negative z-scores in the plots. Do those all stem from transformations where one positive outlier raised the mean area% so much that the mean is higher than the area% of some of the samples within the top 5? Independent on the answer, this phenomenon should be discussed in the manuscript.

4. Some parts of the text would profit from minor improvements and clarifications:

a. p. 6, l. 14-15: This is confusing, the previous paragraph listed SPhos as the second best behind DiMeIHept Cl. If the previous work contrasts this, the authors should provide more context.

b. p. 6, l. 20-21: Do the authors have any explanation where the stark difference for QPhos originates?

c. p. 8, l. 6 & 13: It is not obvious here that "Fitzner et al." is the author´s own previous work, while they just refer to the same paper as "our own work" or "our previous analysis" on the page before. They also suddenly write of the authors in the third person. It should be made clear that they are referring to the same paper as before and that it is their own work.

d. p. 8, l. 26-27: The statement of this sentence is not clear to me. "Also" suggests a follow-up to a previous statement, but there is no relatable statement before. Which difference in substrate complexity do the authors mean? And compared to what are the solvent and base preferences for the coupling of unhindered secondary aliphatic amines differing?

e. p. 9, l. 49-50: Can the authors back up the claim "significant influence" with statistical analysis? What kind of matched pairs do they refer to?

f. p. 10, l. 34: Are those the absolute best base-solvent combinations, or are there different optimal base-solvent combinations for different catalysts?

g. p. 10, l. 44-45: Why does only Cs2CO3 lead to hydrolysis when used as aqueous solution? It seems like this depends on the substrate and not on Cs2CO3? Or were all other inorganic bases weaker?

h. p. 10, l. 48: “The prominent representation of all three combinations in Figure 7” is not comprehensible to me, as there is no information on the water content in the figure. Which three combinations are meant?

i. p. 12, l. 50: Re-normalized to what? This needs to be elaborated.

j. p. 13, l. 8-9: Do the authors have data to back up the claim that the normalized peak area percentages are more robust than native yields?

k. In the Dataset Information (p. 44 of SI), the meanings of the different categories/values should be explained once. What do you mean by Reagents, is a solvent classified as a reagent here? What do the authors mean by Functional Groups, those participating in the bond formation or those present in the substrates? What does AREA_TOTAL_REDUCED mean? And how can the authors have more “Most frequent FGs” than reactions, if there is only one FG in the reaction (e.g. as seen for Cyanation, 264 reactions, 1056 ArBr)?

Author's Response to Peer Review Comments:

Response to Decision Letter

Editorial

Author List: Please place an asterisk (*) after the corresponding author name(s) in the author list.
— Corresponding authors are marked.

Supporting Information: If the manuscript is accompanied by any Supporting Information for Publication, a brief description of the supplementary material is required in the manuscript, before the reference list. The appropriate format is: Supporting Information. Brief statement in non-sentence format listing the contents of the material supplied as Supporting Information.
Please list each supporting item individually.
**Examples of sufficient descriptions: “Supporting Information: 1H NMR spectra for all compounds” or “Additional experimental details, materials, and methods, including photographs of experimental setup.”*

**Examples of insufficient descriptions: "Supporting Information: Figures S1-S3" or "Additional figures as mentioned in the text."*
— Supporting Information statement has been added.

Supporting Information: Please label all graphics/tables in the following format: "Figure S1, S2…", "Scheme S1, S2…." or "Table S1, S2…", etc.
— Captions have been adjusted.

Supporting Information: Please number all pages in the following format: S1, S2, S3, etc. — Page numbering has been adjusted.

Synopsis: ACS Central Science requires a brief synopsis. The synopsis should be no more than 200 characters (including spaces) and should reasonably correlate with the Table of Contents (TOC) graphic. The synopsis is intended to explain the importance of the article to a broader readership across the sciences. Please place your synopsis in the manuscript file after the TOC graphic and label as "Synopsis."
— A synopsis has been added.

> Data analysis of 66,000 high-throughput chemistry experiments results in a practical tool for chemists making complex molecules, delivering insights on what works and thus increasing success rate.

TOC Graphic: Include a TOC graphic illustrating the significance of the paper. The TOC graphic should be something that is representative of your entire work. Color schemes or illustrations typically make good choices. The TOC graphic must be original and free from any copyright issues. Confirm that all text is legible. Present the TOC graphic on the last page of the manuscript by itself. Please label the TOC as "TOC Graphic". A caption describing the TOC is not needed. Please see more information/guidelines for TOC Graphics at the following link: http://pubsapp.acs.org/paragonplus/submission/toc_abstract_graphics_guidelines.pdf?
— A TOC graphic has been added.

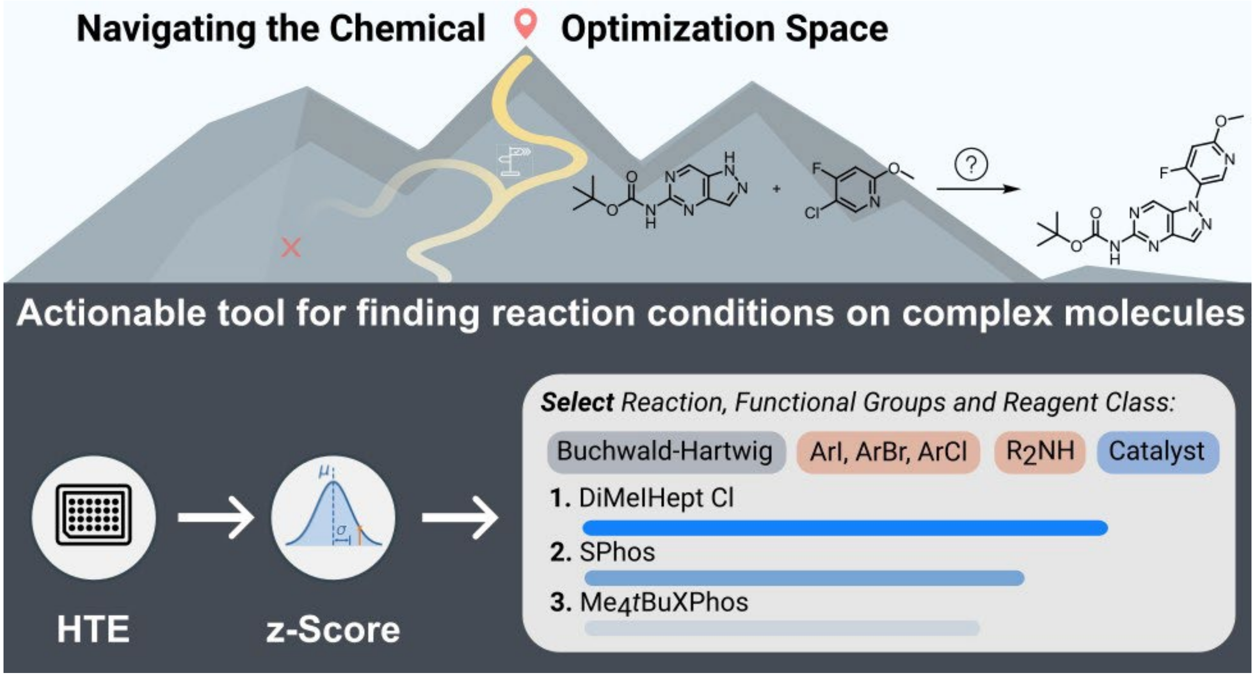

Reviewer: 1

Recommendation: Major revisions required.

Comments:

This study presents a z-score-based statistical analysis method derived from 66,000 high-throughput experiments to recommend optimal reagents for complex drug-like molecules under specific reaction conditions, demonstrating notable innovation and practical value. The public release of the dataset and interactive tool provides data-driven decision support for reaction optimization in chemical synthesis. However, the paper exhibits significant shortcomings in method validation, data quality assessment, bias control, and the generalizability of result interpretation, which undermine the reliability and broader applicability of its conclusions. Furthermore, the discussion insufficiently addresses the limitations of the z-score method, lacks comparative analysis with machine learning approaches, and does not adequately demonstrate the method's applicability beyond Roche's internal datasets. Therefore, it is recommended that the authors supplement with relevant experiments, deepen the methodological

discussion, enhance data transparency, and conduct thorough external validation before consideration for acceptance.

— We thank this reviewer for their constructive feedback. We made considerable efforts to address the following points to warrant acceptance of the revised manuscript.

1. The reliance on LC-MS peak area percentages for quantifying reaction products, rather than isolated yields, somewhat undermines the accuracy and comparability of the data. While the authors justify this approach due to the lack of standards and high-throughput needs, they do not sufficiently discuss the systematic errors introduced, such as variations in response factors between different compounds or the impact of unintegrated side products. It is recommended to supplement with validation experiments on representative reactions, for instance, by performing a correlation analysis between actual isolated yields and peak area percentages, to demonstrate the reliability of this proxy metric. Without this, readers may question the practical guidance value of the z-score rankings.

— We appreciate the reviewer's concern regarding the validity of the assumption to treat peak percentages as a proxy for isolated yields.

For a dedicated, quantitative investigation into this topic, we refer the reviewer to our related publication that we have deposited on ChemRxiv after receiving the feedback on this submission (10.26434/chemrxiv-2025-g7wp0). e have added the following paragraph to the manuscript ~~close to the end of the discussion section~~:

> We demonstrate the predictive power of reduced peak area percentages in a recent publication on the Pd-catalyzed coupling of barbituric and Meldrum's acid with aryl halides. In it, we showed for fourteen examples that high, medium and low peak area percentages in HTE translate well into peak area percentages and finally isolated yields upon scale-up in the lab. We observed that chemical instability during workup or high hydrophilicity preventing extraction can reduce isolated yields, but did not break the link between HTE results and outcomes in the laboratory. (10.26434/chemrxiv-2025-g7wp0)

2. The dataset exhibits significant selection bias, primarily comprising "difficult reactions" and "complex molecules from drug discovery," which means the recommendations may not be suitable for routine or simpler systems. Although the authors mention this bias in the discussion, they do not assess its specific impact on the generalizability of their conclusions. It is suggested to add a dedicated section discussing how this bias affects the recommendations for different reaction types and

to attempt validation on public datasets or standard reactions to illustrate the method's applicability and limitations across a broader chemical space.

— This reviewer correctly states that while extensive, our dataset is not a random sampling of the chemical space and is subject to several inherent biases that influence its composition. Firstly, a survivorship bias exists, as the reactions included are primarily "difficult reactions" where initial attempts by the submitting chemist failed. Transformations solvable through routine literature searches or standard recommended reaction conditions therefore tend to be underrepresented. Thus, we would expect successful hits from these reaction conditions to be less frequent in the dataset and these would thereby have lower z-scores. The user would then be steered towards potentially more expensive catalysts, more polar solvents and bases with lower pK_A's. We cannot know however whether these conditions produce reaction outcomes inferior to literature suggestions. We suspect that both sets of reaction conditions will lead to product formation for simpler substrates and thus disadvantage to the user is limited to being stirred towards more modern catalysts and milder reaction conditions.

Validating simple substrates is a trivial pursuit in our opinion, because these tend to react with any palladium catalyst and solvent combination, not offering much information. In addition, given the size and diversity of our dataset (66k reactions), a dedicated validation effort on a practical scale will not be in relation to the desired effect size.

In response to this reviewer's comment, we have extended the discussion about the biases of our dataset and the recommendations arising from it (adjusted text highlighted in blue):

> While extensive, our dataset is not a random sampling of the chemical space and is subject to several inherent biases that influence its composition. Firstly, a survivorship ~~selection~~ bias exists, as the reactions included are primarily "difficult reactions" where initial attempts by the submitting chemist failed ~~submitted by chemists for HTE~~. Transformations solvable through routine literature searches or standard recommended reaction conditions therefore tend to be underrepresented. Thus, we would expect successful hits from these reaction conditions to be less frequent in the dataset and these would thereby have lower z-scores. We therefore expect that the recommended conditions tend to include modern catalysts and milder reaction conditions (i.e. more expensive catalysts, more polar solvents and bases with lower pK_A) compared to literature-sourced ones (cf. Fitzner et al).[26]

3. While the z-score method is simple and user-friendly, its statistical efficacy depends on the normality and homogeneity of the data distribution. The paper does not adequately test these distributional characteristics of the reaction data. If the data are severely skewed or contain multiple clusters, the z-score may not accurately reflect the true performance of reagents. The authors should supplement with descriptions of the data distributions for different reaction types (e.g., using histograms or Q-Q plots) and discuss whether more robust statistical measures (like Median Absolute Deviation) should be employed for correction in cases of non-normal distributions.

We acknowledge the reviewer's concern regarding the normality and homogeneity assumptions underlying the z-score methodology. And we have to clearly say that the underlying distributions are non-normal. The data exhibits positive skewness (median within-reaction type skewness = 1.36), inherent to reaction data of challenging reactions. None of the reaction types pass the Shapiro-Wilk normality test (α = 0.05). We have added histograms of the underlying distributions for the most common reaction types, as well as skewness and kurtosis to the SI section Dataset Information per Reaction Type.

However, we argue that z-scores remain appropriate for our use case. When a distribution is non-normal, it remains a useful standardized location measure—expressing how many standard deviations a value lies from the mean. What does not hold for non-normal distributions is the use of z-scores for probabilistic inference (e.g., "$z > 1.96$ corresponds to $p < 0.05$").

Since we use z-scores purely for standardization, and not for probability calculations, the non-normality of our distributions does not compromise our conclusions. We present full distributions in the boxplots, which we hope convey the overlap between reagent distributions. This reflects the reality that reagent performance depends heavily on the specific substrate pair.

We should have communicated this more clearly in the paper, so we thank you for the feedback and in turn, have added this section to the end of the method section:

> One important caveat for interpreting the results is that the underlying distributions are non-normal. They exhibit substantial positive skew (median skewness = 1.36) and none of the reaction types pass the Shapiro–Wilk normality test (α = 0.05) (further information is available in the SI sections: Dataset Information per Reaction Type and Underlying Distribution Histograms). This skewness is inherent to challenging reaction datasets. Although z-scores can be computed for any distribution, they do not permit probabilistic inference

when normality is violated. In this work, z-scores are therefore used solely for normalization, not for interpreting probabilities.

4. The paper repeatedly emphasizes the superiority of this method over literature recommendations or machine learning methods but lacks direct, quantitative comparative evidence. For instance, there are no parallel experimental validations comparing the z-score recommended optimal conditions against classic literature conditions on the same substrates, nor is there a systematic comparison with existing predictive models (e.g., descriptor-based or reaction-based machine learning). It is recommended to design a set of control experiments comparing the actual reaction performance under conditions recommended by different methods to strengthen the persuasiveness of the paper's claims.

Following the recommendation of this reviewer, we designed two case study reactions to benchmark the z-score tool with established methods, namely a literature-driven and a machine learning one. To this end, we selected two pairs of sterically hindered amines and bromopyridines from our company inventory, expecting the resulting Buchwald-Hartwig couplings to be difficult in order to get a clear signal.

For both coupling reactions, we then designed one plate each using the z-score app, an
AI-model for Buchwald-Hartwig reactions published by the Denmark group (*Science* **2023**, *381*, 965) and literature precedent, making sure that the literature precedent plates were designed by a different co-worker than the other two, each party without knowledge of the suggested plate designs.

We observed for one of the two, the reaction of 3,3-dimethylmorpholine with 3-bromo-2-methylpyridine, partial conversion to product only on the z-score plate using chloro carbene-based catalysts in the presence of NaO^{t}Bu in either toluene or MeTHF. For the other, the reaction of 2,5-dimethylpyrrole with 3-bromoisonicotinaldehyde, we found no hits for any Pd- or Cu-catalyzed reaction condition. The detailed results, including plate design and pie charts of three samples each can be found in the SI.

Similarly, we have added the following paragraphs to the results section of the manuscript:

In order to evaluate the usefulness of this tool over existing approaches, we selected two pairs of available sterically hindered amines and bromopyridines, expecting the resulting Buchwald-Hartwig couplings to be difficult in order to obtain a large effect size. For both coupling reactions, we then designed one plate each using the z-score app, an AI-model for Buchwald-Hartwig reactions

published by the Denmark group[4] and literature precedent, making sure that the literature precedent plates were designed by a different co-worker than the others.

In the reaction of 3,3-dimethylmorpholine with 3-bromo-2-methylpyridine, partial conversion to product was only observed on the z-score plate using chloro carbene-based catalysts in the presence of NaO^{t}Bu in either toluene or MeTHF. For the reaction of 2,5-dimethylpyrrole with 3-bromoisonicotinaldehyde, we found no hits for any Pd or Cu--catalyzed reaction condition. The detailed results can be found in the Supporting Information.

In the same vein, we have added a new section Results of Comparison Experiments to the Supporting Information. Figures are omitted here to save space.

Results of Comparison Experiments

To benchmark the insights of our dataset compared to literature data and machine learning models, we conducted two reactions using plate designs derived from three inspiration sources. Two Buchwald-Hartwig reactions were selected based on their anticipated high difficulty and availability of the starting materials in the company stockroom and without prior knowledge on the reaction outcomes.

All results we obtained are presented and we did not run any experiments on other transformations. Design of the plates based on literature-precedent was performed by a different person and without seeing the designs created using the z-score app or the Buchwald-Hartwig AI model released by the Buchwald group.[2]. Following our standard procedure,[3] two plates were solid-dosed in a nitrogen-filled glovebox, sealed, transferred to another glovebox where liquids were added, heating/stirring was performed as well as sampling. Information related to the reaction execution can be found in the cheatsheets for these reactions generated by HTE OS that are part of the supporting information.

For the arylation of 3,3-dimethylmorpholine with 3-bromo-2-methylpyridinehe z-sScore app predicted the chlorocarbene catalysts that show at least some activity in this transformation. As these are not part of the scope of the AI prediction model and also not picked up during Scifinder search, neither of these plates showed product.

For the arylation of 2,5-dimethylpyrrole with 3-bromoisonicotinaldehyde, none of the Pd-catalyzed reaction conditions resulted in product. Product mass was observed in some of the Cu-catalyzed conditions on the Plate designed using Scifinder, but the associated peaks in UV were too minute for us to be comfortable to assign them to be product.

Arylation of 3,3-dimethylmorpholine with 3-bromo-2-methylpyridine

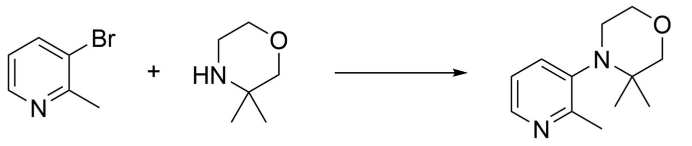

Equation S1: Arylation of 3,3-dimethylmorpholine with 3-bromo-2-methylpyridine

Design through the z-Score App

In order to obtain the design for this plate, catalysts, bases and solvents were retrieved separately for Buchwald-Hartwig reactions of α-branched secondary amines with aryl bromides and chlorides. Because there are only 9 transformations in the dataset for this combination of reaction type and reacting functional groups, the parameter minimum number of ELNs in the options section of the app was set to one. The top six catalysts were selected and amended by two catalysts further down the list that are differ structurally significantly from the others. For solvents (minimum number of ELNs = 5) and bases (minimum number of ELNs = 4), the top four entries were chosen and from all permutations 12 chosen based on chemical compatibility and diversity. The resulting plate design as well as the reaction outcomes are shown in Figures SX to SX.

Figure S9: Results of z-score plate, Sample 1 taken after stirring at 2 h, 80 °C.

Figure S10: Results of z-score plate, Sample 2 taken after stirring at 4 h, 80 °C.

Figure SX: Results of z-score plate, Sample 3 taken after stirring at 21 h, 80 °C. We highlighted the LCMS of vial A3 to give an impression of the size of the product peak and the cleanliness of the reaction. Also visible are the small amounts of what we believe to be product isomer formed in column 9 (KHMDS/PhMe).

We observe two distinct groups of peaks with product mass, one formed in the presence of chlorocarbene catalysts and NaOtBu and one group in the presence of KHMDS in toluene. The peaks of the latter are only barely visible in Figure S5 and are consistent with respect to retention time to the peaks observed on the AI plate under the same conditions. The former group of peaks was assigned to product, as it has a retention time distinct from the benzyne product, is associated with the expected mass and is formed under a set of related conditions. Also, we couldn't conceive of other product isomers that could be formed under these reaction conditions. Although we haven't isolated the product and characterized it, we believe the peak is real and the result a good starting point for further optimization.

Design through Scifinder research

The design of the literature-driven plate was based on Scifinder reaction query (Equation S2), searching for C-N disconnections of the indicated substructure, which afforded 11k hits. The results were manually screened and transition-metal free reactions (e.g.
nucleophilic aromatic substitution) were excluded. A 96 well plate was designed based on the prevalent catalysts, bases and solvents.

Equation S2: Scifinder query used to find reaction conditions.

Figure S11: Results of Scifinder plate, Sample 1 taken after stirring at 2 h, 80 °C.

Figure S12: Results of Scifinder plate, Sample 2 taken after stirring at 4 h, 80 °C.

Figure S13: Results of Scifinder plate, Sample 3 taken after stirring at 19 h, 80 °C.

Design through AI Prediction

The prediction of the reaction outcomes was conducted using the published version of the model published by Denmark et al.[2] The results are shown in Figure SX.

Figure S14: AI prediction for the arylation of 3,3-dimethylmorpholine with 3-bromo-2-methylpyridine.

Based on this prediction, we arrived at the following plate layout (Figure SX). As the number of reagent combinations predicted to result in product was smaller than 96, we filled the plate with similar solvents and bases as well as with catalysts predicted to have small or no activity. Et-CPhos was omitted, since we do not have a Pd-precatalyst of it available.

Figure S15: Plate designed for the arylation of 3,3-dimethylmorpholine with
3-bromo-2-methylpyridine based on AI prediction. Reagents predicted to lead to product conversion highlighted in green.

Figure S16: Results of the AI prediction plate, Sample 1 taken after stirring at 2 h, 80 °C.

Figure S17: Results of the AI prediction plate, Sample 2 taken after stirring at 5 h, 80 °C.

Figure S18: Results of the AI prediction plate, Sample 3 taken after stirring at 22 h, 80 °C.

The results shown in Figure SX to SX indicate that no product is formed under any of these conditions. We do observe what we believe to be the formation of the 4-pyridyl isomer in the presence of KHMDS/PhMe, a peak with a retention time distinct from what we believe to be the product that we observed in the z-score plate. As the formation of the 4-pyridyl isomer proceeds through a benzyne mechanism, its presence irrespective of catalyst would be explained.

Arylation of 2,5-dimethylpyrrole with 3-bromoisonicotinaldehyde

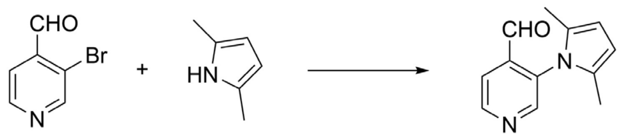

Equation S32: Arylation of 2,5-dimethylpyrrole with 3-bromoisonicotinaldehyde.

Design through the z-Score App

In order to obtain the design for this plate, catalysts, bases and solvents were retrieved separately for Buchwald-Hartwig reactions of aromatic NH-groups with aryl halides. We selected catalysts based on the top ligands as well as appropriate combinations of the top bases and solvents.

Figure S19: Results of z-score plate, Sample 1 taken after stirring at 3 h, 80 °C.

Figure S20: Results of z-score plate, Sample 2 taken after stirring at 5 h, 80 °C.

Figure S21: Results of z-score plate, Sample 3 taken after stirring at 19 h, 80 °C.

The results shown in Figure SX to SX indicate that no product is formed under any of these conditions.

Design through Scifinder research

The design of the literature-driven plate was based on Scifinder reaction query (Equation S4), searching for C-N disconnections of the indicated substructure, which afforded 28k hits. The results were manually screened and transition-metal free reactions (e.g. pyrrole ring formation) were excluded. A 96 well plate was designed based on the prevalent catalysts, bases and solvents.

Equation S4: Scifinder query used to find reaction conditions.

Figure S22: Results of Scifinder plate, Sample 1 taken after stirring at 3 h, 110 °C.

Figure S23: Results of Scifinder plate, Sample 2 taken after stirring at 7 h, 110 °C.

Figure S24: Results of Scifinder plate, Sample 3 taken after stirring at 23 h, 110 °C.

The results shown in Figure SX to SX indicate that no product is formed under any of these conditions. For the Cu-catalyzed conditions that were included on this plate because they were part of the search results, we observed what could be interpreted as traces of product, but the peaks were so small and inconsistent that we decided against tagging them as such.

Design through AI Prediction

The prediction of the reaction outcomes was conducted using the published version of the model published by Denmark et al.[2] The results are shown in Figure SX.

Figure S20: AI prediction for the arylation of 3,3-dimethylmorpholine with 3-bromo-2-methylpyridine.

Based on this prediction, we arrived at the following plate layout (Figure SX). As the model predicts little conversion for the whole condition space, we included conditions that were predicted to show at least some activity:

Figure S21: Plate designed for the arylation of 2,5-dimethylpyrrole with 3-bromoisonicotinaldehyde.

Figure S22: Results of the 2nd AI prediction plate, Sample 1 taken after stirring at 3 h, 80 °C.

Figure S23: Results of the 2nd AI prediction plate, Sample 2 taken after stirring at 5 h, 80 °C.

Figure S24: Results of the 2nd AI prediction plate, Sample 3 taken after stirring at 19 h, 80 °C.

The results shown in Figure SX to SX indicate that no product is formed under any of these conditions. This outcome is in good agreement with the predicted, low, yields.

5. The authors mention that "users can filter reagents" through the tool but do not specify whether the tool supports user-uploaded data or the addition of new reaction types, which limits its general utility and long-term value.

The option to upload a dataset has been added to the web app, to allow users to complement the built-in dataset with additional reaction data. The upload occurs on the user side and is not stored or sent back outside the browser session.

The list of reaction types and functional groups is dynamically generated from the dataset.

Furthermore, the algorithmic details behind the "recommendations" (e.g., the rationale for the default choice of 'n' in "top n z-scores") are not sufficiently explained, potentially affecting user trust in the results. It is advised to provide more detailed parameter tuning guidance in the Methods section or Supplementary Information and consider developing features that support user data import.

Regarding the choice of the parameters “Minimum Number of ELNs” and “Top-N z-Score per
(ELN_ID, selected reactant type(s))”, we aim to strike a balance between robust performance (large n) of a reagent and rewarding reagents for extraordinary performance under specific conditions (small n).

The methodology section has been supplemented with an explanation about the number of transformations parameter, its effect and our rationale for setting it at the default value (new text in blue).

> Our data processing workflow is as follows: First, we calculate the z-scores of all experiments in a given chemical transformation. Then we calculate the median of the top n z-scores in which a given reagent was present. When n is large, reagents are preferred that show good performance under a variety of conditions. When n is small, individual examples of large significant outperformance are weighted higher. By default, n is set to five which in our experience strikes a good balance between selecting for robust performance (large n) of a reagent and rewarding reagents for extraordinary performance under specific conditions (small n).
>
> [...]

Empirically, we set the minimum number of transformations as well as the number of z-scores to be included to five. Importantly we do not include all z-scores per category and transformation. This is critical as we search for the optima of the reaction space and therefore disregard the minima. Users can influence the displayed ranking by filtering out reagents that were not used in a minimum number of transformations. The minimum number of transformations in which a reagent has to be present in order to be displayed prevents outliers from being displayed that showed good results only in a small number of transformations. Specific conditions present in the substrates tested may favor a given reagent which would have failed to perform if it had been used more broadly. In some cases, users may be interested in these outliers and thus reduce that number. This can be because even rarely used reagents should be tried or because the combination in question of transformation, reacting functional groups and component roles contains not many data points ~~like following sparse literature precedent, the risk increases that~~.

6. When discussing catalyst performance variations, the authors attribute some results to "batch quality differences" or "selection bias" but do not explain how these factors were identified or controlled. As the activity of catalysts from different batches can significantly impact reaction outcomes, it is recommended to introduce quality control metrics during data collection (e.g., catalyst purity, batch numbers) and incorporate them as covariates in the analysis to improve the rigor of the conclusions.

— The reviewer addresses a valid point regarding the batch-to-batch variability in reagents, which is a general challenge in practical chemistry, extending beyond HTE and transition metal catalysis. In this work, and within our laboratory, we implement a comprehensive batch-tracking system that allows us to trace back the specific reagents (e.g., catalysts) employed in each experiment. Specifically, we exclusively purchase commercial catalysts with corresponding Certificates of Analysis and store them under an inert atmosphere. Furthermore, we transfer small aliquots into a nitrogen-filled glovebox for dispensing in HTE, while the remainder of the same batch is kept in the fridge for long-term storage.

In addition, given the nature of our HTE activities, we utilize very small amounts (i.e., milligram quantities) of catalysts for a given reaction campaign. As a result, we rarely need to switch to a new catalyst batch in the span of this dataset. In the few instances where a new batch is required, we reorder from the same supplier to minimize the potential for significant deviations in quality. Therefore, for a statistically large-enough HTE campaign, the performance variability attributed to batch differences is minimized and effectively constant. While we agree with the principle of including batch numbers

as covariates, the consistent sourcing and dispensing procedure ensures that the impact of this variability is substantially mitigated in our data set.

In response to this reviewer's comment, we clarified that we aim to reduce the mentioned batch-to-batch variability as explained above (added text highlighted in blue):

> We have to caution however, that these differences could also result from batch quality differences or biases present in catalyst selection and reaction substrates. In our laboratory, we minimize unexplained batch-to-batch variability by implementing a batch-tracking system and purchasing large amounts of commercial reagents from the same suppliers.

7. The paper lacks discussion on the reaction scale effect. High-throughput experiments are typically conducted on microgram to milligram scales, whereas practical synthesis often occurs on gram scales. Differences in mixing efficiency, heat transfer, catalyst loading, etc., between these scales may affect the direct transferability of conditions. The authors should supplement the discussion with considerations of the scale effect and, if possible, provide scale-up validation results for some reactions.

— We appreciate the reviewer's concern regarding the reaction scale effect and HTE translatability. The transfer of reaction conditions from HTE screenings in micro- to milligram scale to round-bottom flask chemistry on gram scale is a continuously studied challenge, as evidenced by accounts from other HTE groups. In our opinion, HTE serves to identify initial hits which informs subsequent optimization efforts for robust, scalable conditions performed at the traditional bench scale.

While this manuscript lacks an exemplified case study to bridge reaction performance across scales, we have successfully scaled numerous reactions, confirming that the best conditions are reliably reproduced on a larger scale. For a dedicated, quantitative investigation into this topic, we refer the reviewer to our related publication that we have deposited on ChemRxiv after initial submission of this manuscript (10.26434/chemrxiv-2025-g7wp0).

To comment more broadly on the transferability of HTE hits to traditional bench chemistry, we have taken several measures to reduce the amount of change observed between well-based and flask-based reactions. For instance, our experimental design utilizes choices like direct solid dispensing and mixing with magnetic stir plates. Further, to address potential differential scaling effects, we employ a 'split and pool'

scale-up strategy, running the identical reaction condition in 96-well format and then combining several to afford a large quantity of target compound.
Although miniature screenings require higher relative catalyst/base loadings for practical reasons, these parameters are kept constant during scaling to maintain an equivalent concentration profile.

In response to this reviewer's comment, we have added the following paragraph to the manuscript close to the end of the discussion section:

> A necessary caveat when interpreting datasets derived from HTE campaigns is the challenge of translatability to conventional bench-scale synthesis. This challenge arises from differences in heat and mass transfer, mixing efficiency, degree of solid grinding, and surface area-to-volume ratios between micro- and gram scales. However, it cannot be overstated that HTE primarily serves to identify good starting points for process optimization; robust and scalable conditions require dedicated follow-up optimization at the bench. In our hands the most successful HTE conditions qualitatively reproduce the optimal outcome (e.g., product/selectivity) on larger scale. Moreover, we could show that calibrated peak area percentages agree with isolated yields of scale-ups in a quantitative case study (10.26434/chemrxiv-2025-g7wp0).

8. Although the authors provide a public dataset and online tool, the lack of substrate structure information severely limits its value for machine learning or more detailed structure-activity relationship studies. While confidentiality concerns are understandable, it is recommended to provide descriptors of key structural features of the substrates (e.g., number of functional groups, polarity, steric hindrance) to help readers better understand the impact of "drug molecule complexity" on reaction condition selection and to facilitate transfer learning or model development by other researchers.

We appreciate this reviewer's suggestion to provide substrate structure information or descriptors to enhance the utility of the dataset for machine learning (ML) and structure-activity relationship (SAR) studies. We agree such information would be highly valuable.

Due to necessary confidentiality concerns and intellectual property protection, we must maintain the anonymity of the synthesized structures. Providing specific descriptors of compounds at the individual level carries a significant and increasing risk of reverse engineering, a challenge commonly recognized in industry publications, for example in Raghavan et al. JACS 2024, 15070. Therefore, we can't provide individual descriptor

data granular enough to be useful for machine-learning without risking disclosure of project-related compounds. We believe the public dataset and online tool still provide significant value to the community despite the constraints on compound-specific data sharing.

Notwithstanding these restrictions, we are able to share the distribution of 11 descriptors of the overall dataset similar to what was done in Raghavan et al. JACS 2024, 15070, SI page S7. These include measures of drug-likeness (clogP, fraction C(sp)^3, total polar surface area, number of hydrogen bonding acceptors/donors), as well as counts of rings, heteroatoms, heavy atoms and rotatable bonds and molecular weight.

In the Supporting Information we have added a figure describing the distribution of these descriptors. We are confident these will allow the readers to assess the kind of chemical space we base our analysis on.

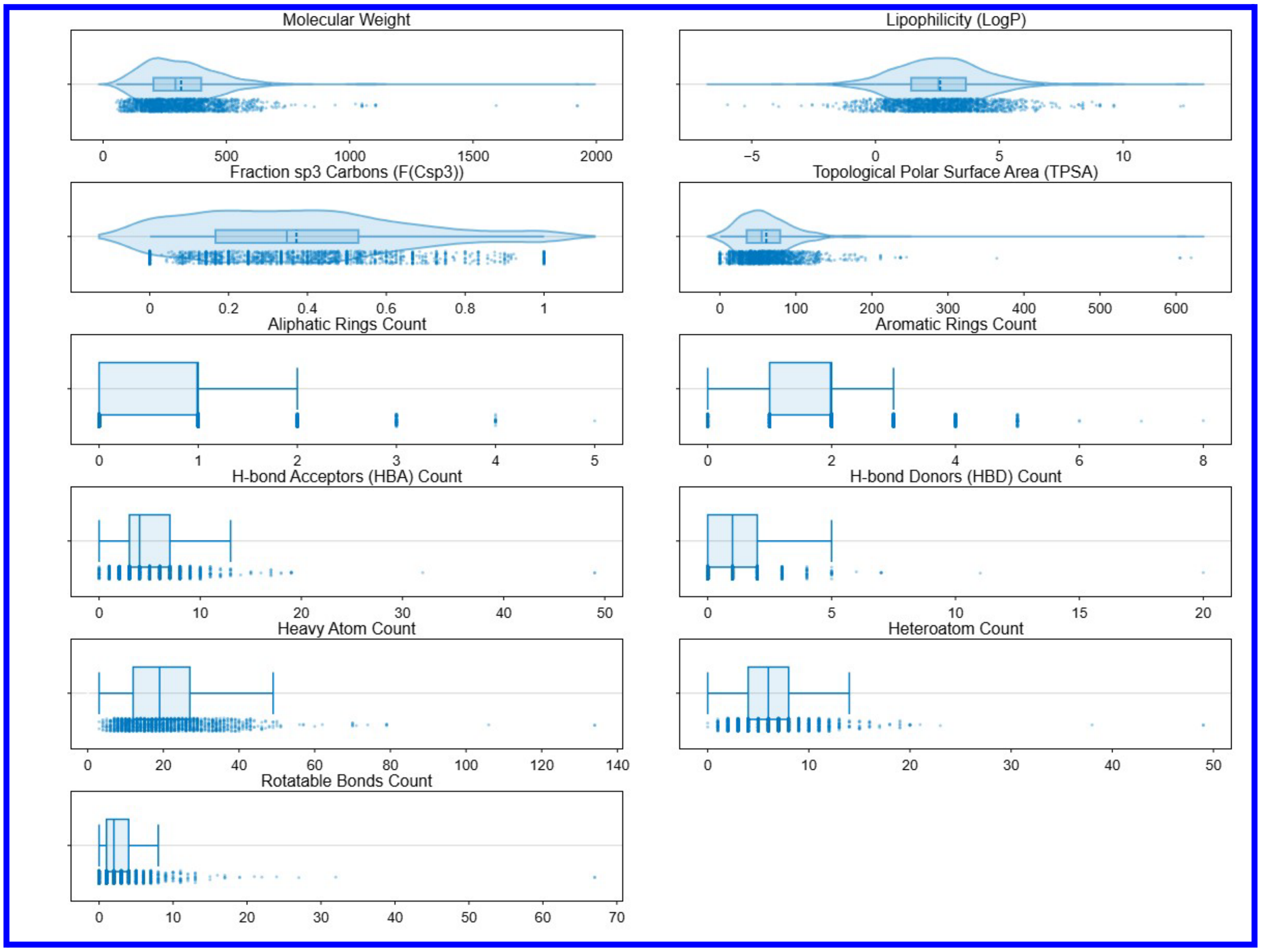

Figure S2: Boxplots of 11 descriptors of the synthesized products in this dataset. Descriptors were calculated with rdkit version 2024_03_3.[1]

A sentence to guide the reader to this data has been added to the introduction section (added text in blue):

> Most transformations we receive failed to yield meaningful amounts of product before being submitted for screening. Thus, our dataset is skewed towards difficult reaction types and substrate combinations, where small changes to the reaction conditions or substrate structure lead to big changes in outcome. Descriptors of the products of the reactions screened in this dataset are shown in Figure S2 in the Supporting Information (SI). We often receive advanced intermediates as starting materials and our customers expect us to use them resourcefully for a maximum number of experiments. Conversely, reducing reaction scale too much can compromise result quality, specifically for heterogeneous reactions or those conducted close to the reaction medium's boiling point. Therefore, we try to reduce the number of experiments by increasing the hit-rate and quality.

Reviewer: 2

Recommendation: Major revisions required.

Comments:

The authors have conducted a retrospective statistical analysis of 66,000 reactions from High Throughput Screenings for reaction conditions of various transformations performed in their facility, with the goal of creating a tool that suggests the most promising reagents for a given transformation of certain educts. After analyzing the results of the reactions via LC-MS, they used a normalized area% of the transformation's product to calculate a z-score for each experiment, stating how many standard deviations the value is away from the mean product area% of all experiments of the respective transformation. They then built an analysis tool that combines the top results for a certain reagent in each transformation for all transformations in a class of educts and reaction type to identify the best-performing reagent for this combination. They offer an interactive implementation of their tool, combined with the underlying dataset, as a GitHub repository and as a hosted web application. In the manuscript, they then discuss the results for the two most prevalent reaction types of their dataset: Buchwald-Hartwig and Suzuki-Miyaura cross-coupling reactions. They compare the results with those of a similar study based on literature-reported Buchwald-Hartwig reaction conditions which they have published before. They found that the optimal reagents as determined retrospectively from their HTE differ from the optimal reagents according to the literature, which they explain by the bias of their selection of educts towards "difficult reactions" of highly functionalized building blocks, while literature contains more examples for basic transformations.

The manuscript is generally very well written, the data and analysis seems to be sound and the goal of the study is highly relevant. A tool that suggests optimal reagents based on experimental data from thousands of reactions performed under standardized conditions in the same lab is very valuable to the community, as the research for optimal conditions in the literature always suffers from limited comparability between labs and publications.

— We thank this reviewer for their positive evaluation and share their optimism about the relevance of this tool for the community.

The following points need attention:

1. The figures and captions need to be improved.

a. *In my opinion, Figure 1 does not meet the standards of professionalism that I expect from a journal such as ACS Central Science. The resolution is also too low. Additionally, it might be AI-generated. Unless it was drawn by one of the authors, I would expect crediting the source or AI tool it was created with.*

We thank the reviewer for their insightful comment about the figures in this manuscript. We have replaced the illustrative Figure 1 with a descriptive alternative that introduces the context of this work more clearly. We hope this figure will satisfy the expectations put towards articles published in ACS Central Science.

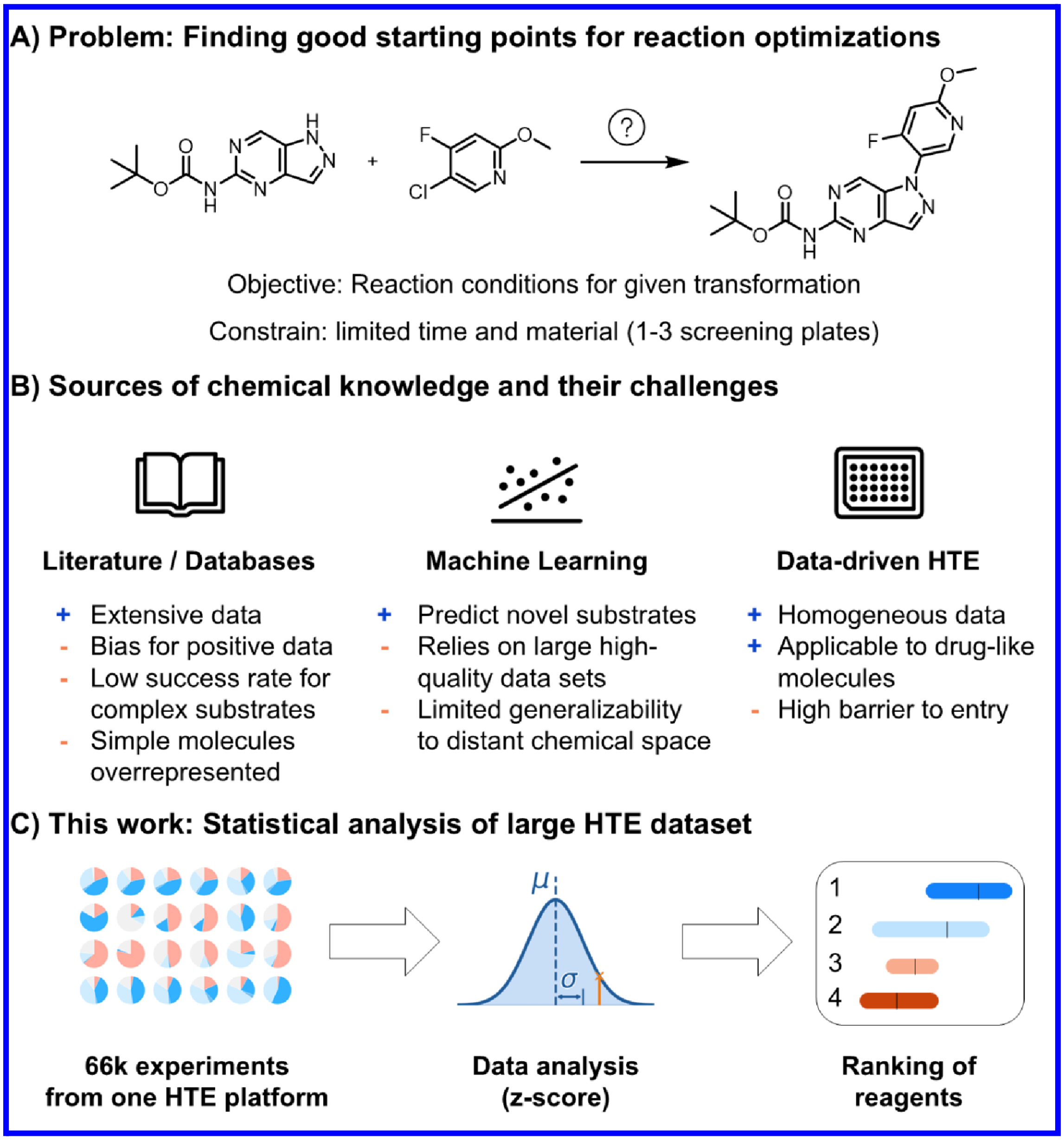

Figure 1: A) Reaction optimization for molecular synthesis is a complex problem and in the pharmaceutical industry material and time resources are limited. B) Starting points for an experimental screening campaign can arise from literature mining, machine learning algorithms or mining of experimental high-throughput experimentation data. C) In this work we showcase how statistical analysis of a large HTE-derived data set enables ranking of promiscuous reaction conditions.

b. The Boxplots

i. Standard box plots only show the outliers as data points, deviations from the standard format should be explained in the caption.

ii. The color gradient is not explained and is misleading, as it draws away attention from the best performing ligand. It is not immediately obvious that ligands are sorted by performance from top to bottom and the color gradient marks the number of data points. Both should be explained in the caption.

iii. The axis labels are much too small to be readable and serif fonts should be avoided in figures & graphs. A vector-based format would improve resolution.

We have improved the boxplot figures and the decision tree figure with higher resolution, improved the axis labeling and changed the font size and type. We will provide vectorized drawings at the proof stage. Also, we have explained the color gradient in the caption.

In response to this reviewer's comments, we adjusted the captions of Figures 3-4, 6-7 (added text highlighted in blue):

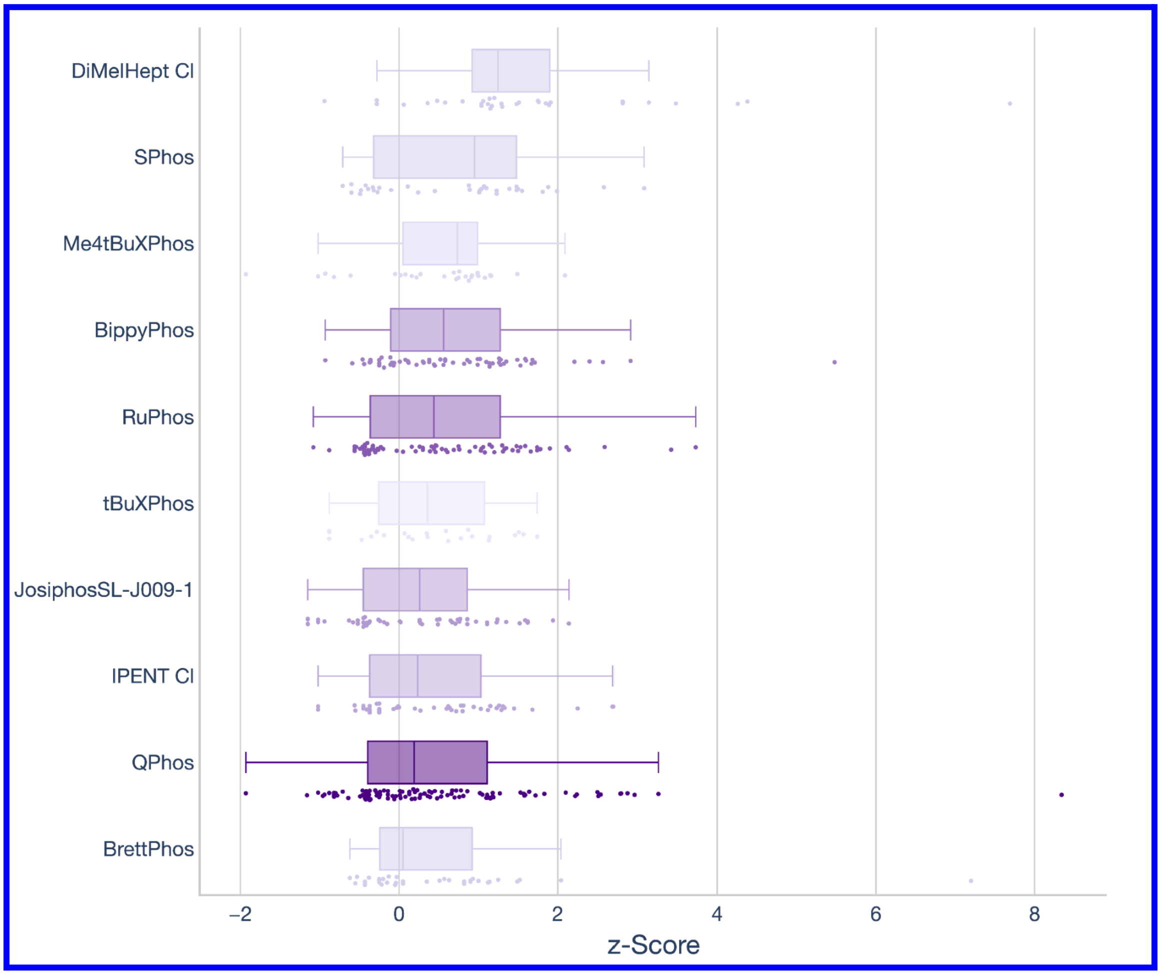

Fig. 3: Boxplot of z-score by ligand for aryl bromides/aryl chlorides reacting with secondary amines. The top 10 ligands that are shown were used in at least five different chemical transformations and the top five hits per transformation were considered for each ligand. Below each boxplot we show all underlying datapoints. We use a color gradient to indicate the number of reactions, thereby highlighting ligands that are supported by a greater number of datapoints.

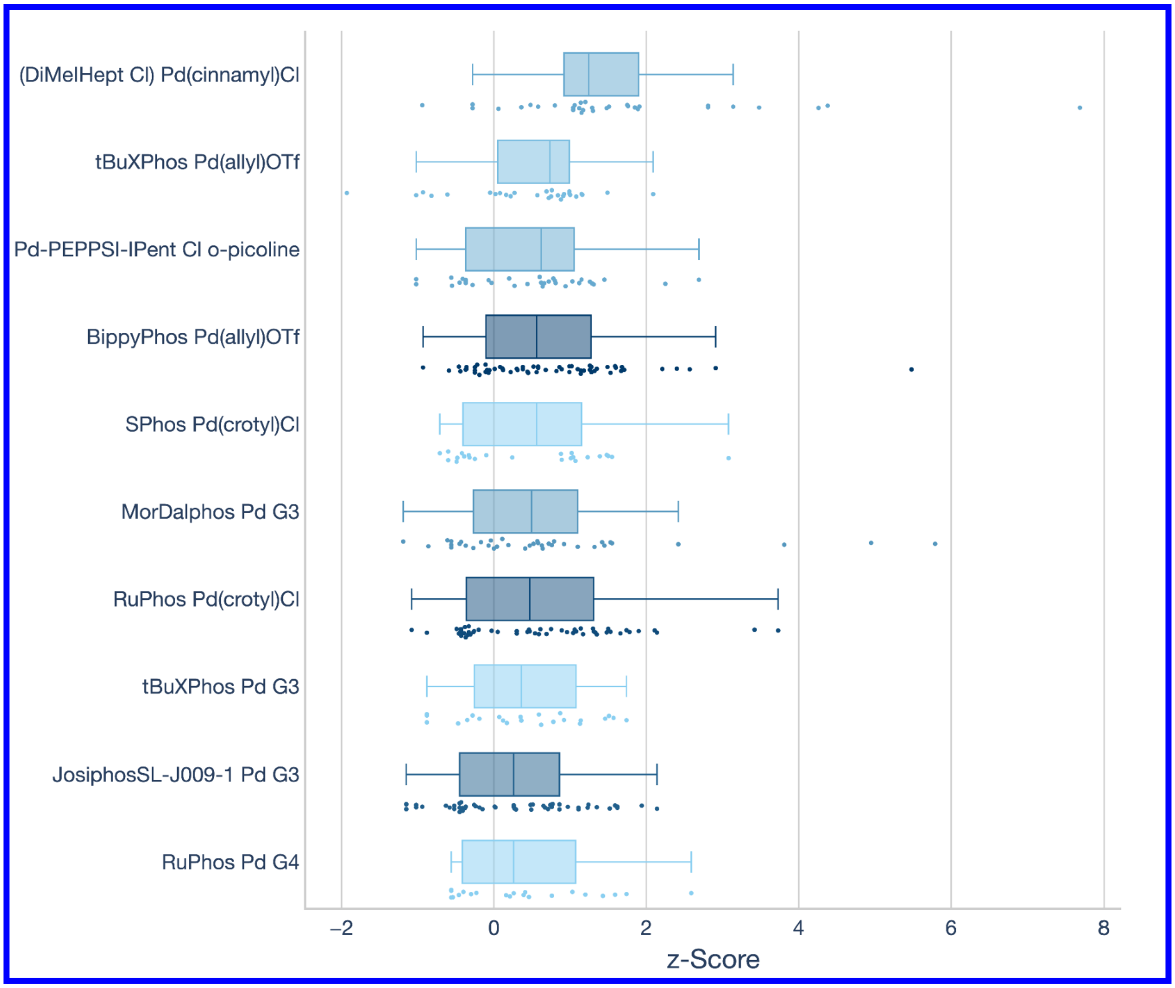

Fig. 4: Boxplot of z-score by catalysts for aryl halides reacting with secondary amines. The top 10 catalysts that are shown were used in at least five different chemical transformations and the top five hits per transformation were considered for each catalyst. Below each boxplot we show all underlying datapoints. We use a color gradient to indicate the number of reactions, thereby highlighting catalysts that are supported by a greater number of datapoints.

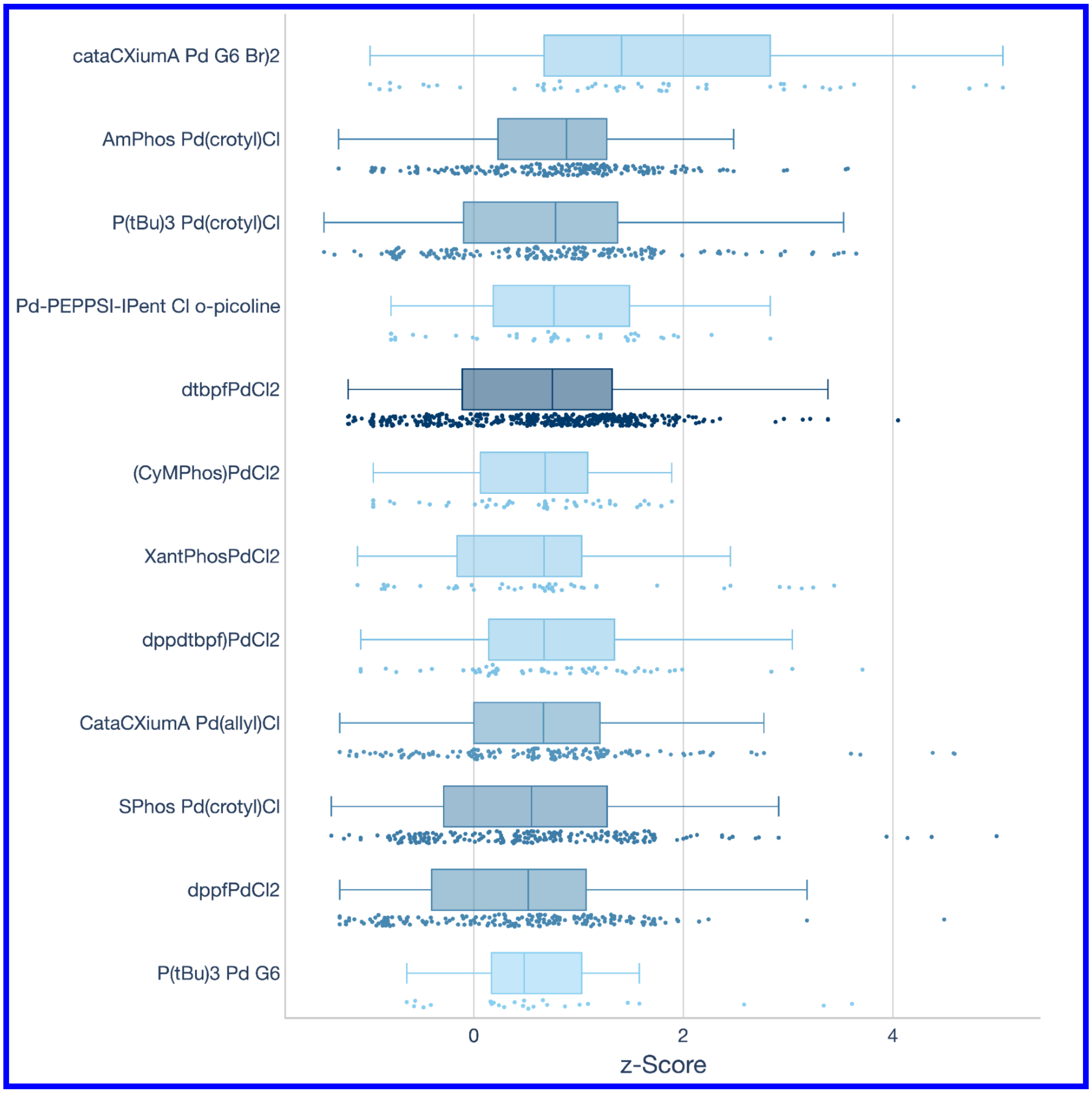

Fig. 6: Boxplot of z-Score by catalyst for aryl halides reacting with different types of aryl boronates.The top 12 catalysts that are shown were used in at least five different chemical transformations and the top five hits per transformation were considered for each catalyst. Below each boxplot we show all underlying datapoints. We use a color gradient to indicate the number of reactions, thereby highlighting catalysts that are supported by a greater number of datapoints.

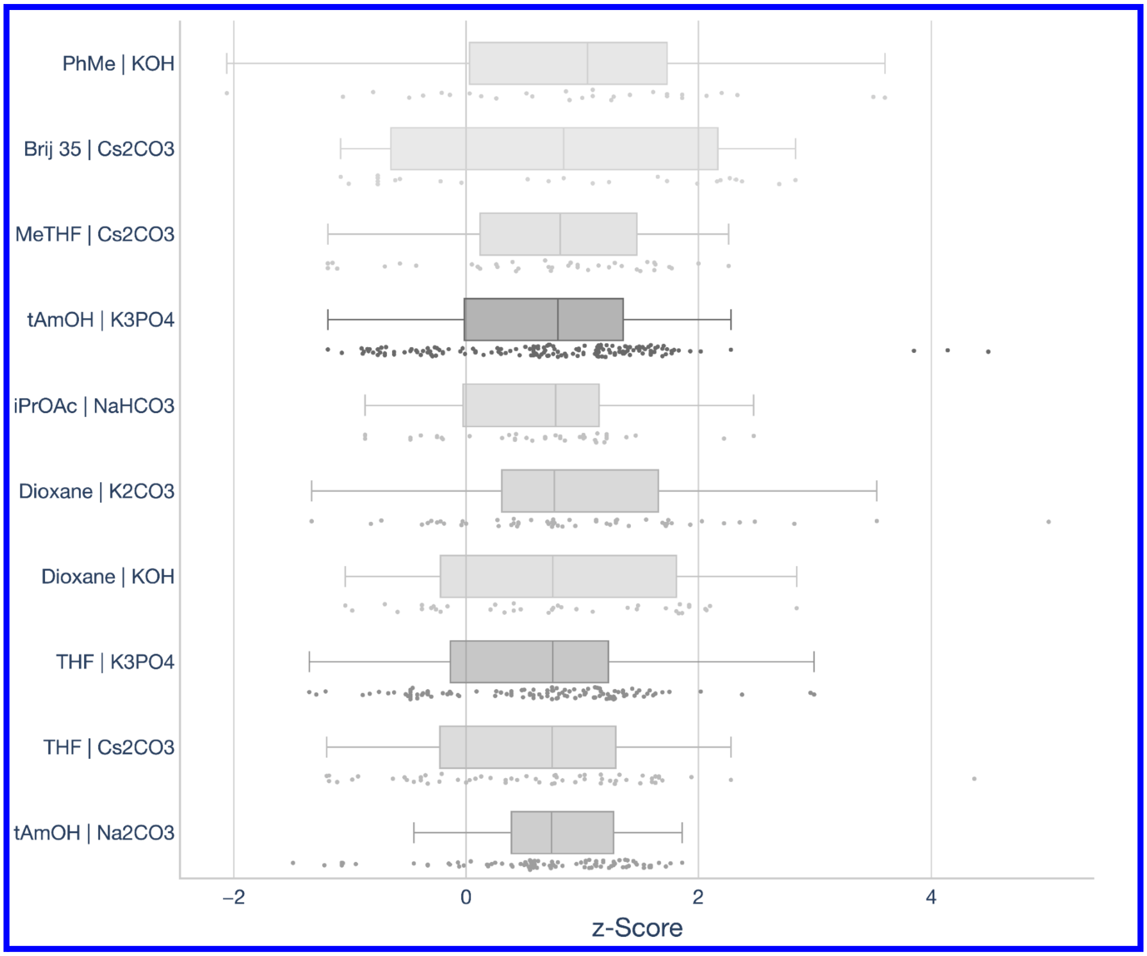

Fig. 7: z-Score Boxplot of solvent/base-combinations for aryl halides reacting with different types of aryl boronates in Suzuki-Miyaura couplings. The top 10 Base-solvent combinations that are shown were used in at least five different chemical transformations and the top five hits per transformation were considered for each catalyst. Below each boxplot we show all underlying datapoints. We use a color gradient to indicate the number of reactions, thereby highlighting bases that are supported by a greater number of datapoints.

It should be noted that Figure 7 in the revised version shows a modified ranking of solvent/base-combinations for aryl halides reacting with different types of aryl boronates in Suzuki-Miyaura couplings compared to the initial version. We admittedly could not reproduce the previous figure, which we attribute to the small differences in medians or a malfunction of the web app. Now the figure aligns with the output of the web app.

iv. Figure 6 lacks an x-axis

Figure 6 was updated to include an x-axis (see previous point).

v. Can the authors make any statements on the statistical significance of your results, e.g. the differences between reagents? It looks like the datapoints spread very far and the medians are very close to each other for almost all ligands shown.

Regarding the question of whether we can make statements on the statistical significance of differences between reagents: We cannot apply standard statistical tests to assess significance due to violations of independence assumptions. Standard statistical tests for comparing groups (Kruskal-Wallis, Mann-Whitney U) assume independent observations. Our data structure, and HTE experiments by design, violate this assumption:

- Multiple observations originate from the same experiment (ELN)
- Experiments are conducted iteratively, making later results depend on earlier results

Applying these tests would yield misleading p-values. Instead, we present the full distributions in our boxplots, which show the substantial spread reflecting variability across substrate pairs and allow visual assessment of overlap between reagent distributions. The boxplots demonstrate that while certain reagents consistently achieve higher median z-scores, substantial overlap exists between rankings. This reflects the reality that reagent performance depends heavily on the specific substrate pair.

In turn of the feedback of the reviewer we have added this section to the SI, to clarify this question to the reader:

> Limitations on Statistical Significance Testing
>
> Standard statistical tests for comparing groups (e.g., Kruskal-Wallis, Mann-Whitney U) assume independent observations. Our data structure and all HTE data are in violation with this assumption by design. Multiple observations originate from the same experiment (ELN) and experiments are done iteratively, making them dependent. Therefore, we cannot apply standard statistical tests to assess the significance of differences between reagents, as this would yield misleading p-values. Instead, we present the full distribution in the boxplots, which convey uncertainty more honestly than a single p-value and allow visual assessment of overlap between reagent distributions. This reflects the reality that reagent performance depends heavily on the specific substrate pair.

c. The decision tree (Fig. 5)

i. The text of the figure is too small in comparison with the size of the figure, while there is a lot of blank space. Also, the text is very pixelated, a vector-based graphics format would be more suitable. Figure 3b) of the previous work (Fitzner et al., 26) is a good example of how it could be improved

ii. In the "Legend" box, the abbreviation ELN is used as a label and it is also very prevalent in the webtool, but it is never explained what ELN stands for. The whole notation scheme {1. name (#)z (#)ELN} looks quite crowded at this small font size and could be formatted clearer. The two labels "No Recommendation …" and "Recommended …" are in different font sizes.

iii. In the box for primary anilines, Ad-BrettPhos is written with a hyphen, while it is written together in line 24 on page 6. The last entry in the same box (Triisobutylphosphatrane) is misaligned after the line break

We have redrawn the decision tree figure in vector format, increasing font size and resolution.
Also, we have simplified the information content, moving the legend into the caption. The ELN (electronic lab notebook) number represents the number of independent reactions. The color scheme was adjusted and the rank reported in Fitzner et al. was omitted for clarity. Lastly, we removed the typos.

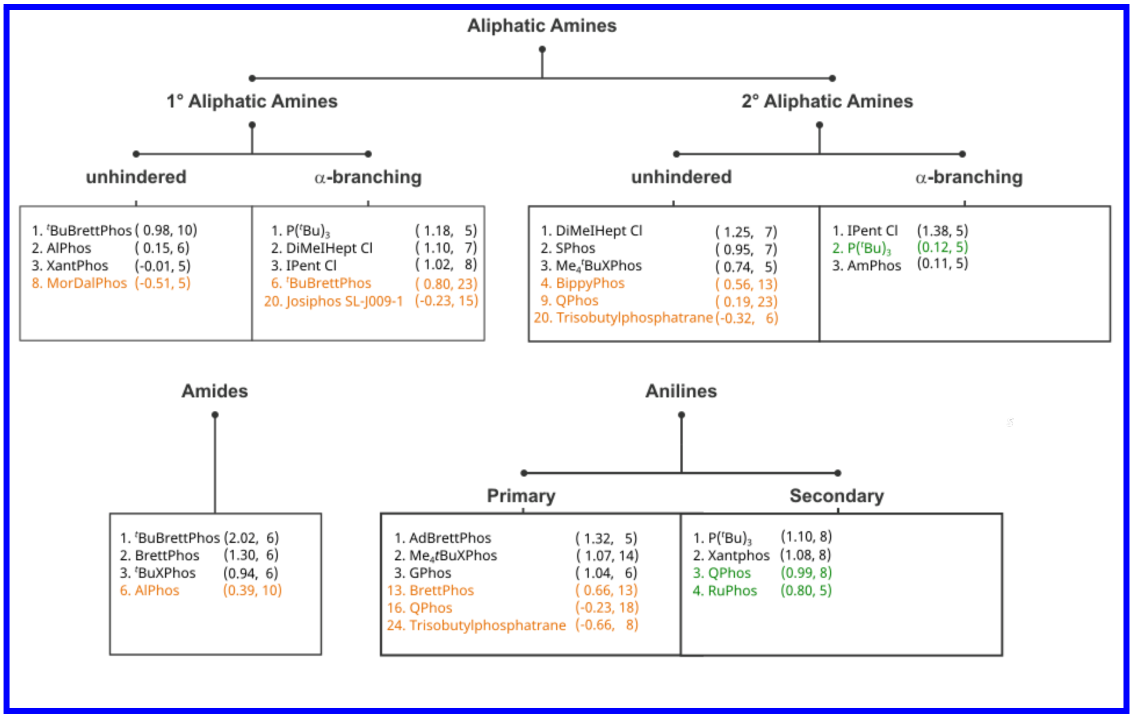

Fig. 5: Hierarchical tree diagram illustrating the recommended Pd ligands for the Buchwald-Hartwig cross-coupling of aryl halides with three primary nucleophile classes: aliphatic amines, amides, and anilines. Ligand recommendations are based on z-score across a minimum of five synthetic transformations for each substrate category (z-score and number of experiments in brackets). ~~Comparison of the best ligands for Buchwald-Hartwig cross-couplings for aliphatic amines, amides and anilines reacting with aryl halides. All ligand recommendations are based upon at least five transformations. Not all~~ Recommendations previously published by us when analyzing literature data are indicated in orange if they do not overlap, and green if they are shared in both sets~~ by Fitzner et al~~.[26] Top performing ligands in the literature dataset that are underrepresented in our dataset are omitted.~~ Not all ligands are included in our graph, as we lack sufficient data in our dataset.~~

d. The caption of Supplementary Figure 1 lacks an explanation for the yellow and blue backgrounds of some of the ligands. Also, it seems to be adapted from a different source, which means there should be a statement in the caption where this figure was published first and whether the authors obtained permission to reproduce the figure in this work.

Figure S1 was created by us as an internal resource and therefore we own the rights to it. This is the first time it is published.

Based on our experience we have classified the ligands that performed consistently well for Suzuki-Miyaura and Buchwald-Hartwig reactions in yellow and blue, respectively. It should be noted that this classification was not created by a quantitative method but our qualitative observations.

The caption of Figure S1 in the supporting information has been amended to explain the color coding of the ligands (new text in blue).

> Figure S1: Pd-Ligands employed by us in the form of precatalysts. The arrangement in the 2D-plane is based on structural similarity and observation of similar behavior in coupling reactions. Blue shading indicates ligands we found to perform consistently well in Buchwald-Hartwig reactions. Yellow shading indicates ligands we found to perform consistently well in Suzuki-Miyaura couplings.

2. For publication in a Diamond Open Access Journal, I expect full transparency and publication of all underlying data that is necessary to comprehend and use the results of the work. I fully understand that the authors cannot release detailed structures of compounds from the company or clients. However, as the authors state, the conditions found to be optimal are most likely highly dependent on the substrate structure, as the literature contains different optima for simpler substrates, compared to the highly functionalized substrates of the "difficult reactions" in the data set. This means the tool is probably of very limited use if it can only be queried by broad subcategories like "ArNH2".

We share the reviewer's stance towards open access data. Accordingly, we disclose both the underlying data set, as well as the code repository of the web app to query it. We appreciate this reviewer's suggestion to provide more detailed substrate structure information or descriptors to enhance the utility of the dataset for arriving at actionable conclusions. We agree such information would be highly valuable.

However, due to necessary confidentiality concerns and intellectual property protection, we must maintain the anonymity of the synthesized structures. Accordingly, we can not specify the structures further than the reacting functional group. For instance, providing compound-specific descriptors carries a significant and increasing risk of reverse engineering, a challenge commonly recognized in industry publications. Therefore, providingany compound-specific descriptor data is a hard limitation of this work, aligning with the common pharmaceutical practice. We believe the public dataset and online tool still provide significant value to the community despite the constraints on compound-specific data sharing.

Notwithstanding these restrictions, we are able to share the distribution of 11 descriptors of the overall dataset similar to what was done in Raghavan et al. JACS 2024, 15070, SI page S7. These include measures of drug-likeness (clogP, fraction C(sp)[3], total polar surface area, number of hydrogen bonding acceptors/donors), as well as counts of rings, heteroatoms, heavy atoms and rotatable bonds and molecular weight.

In the Supporting Information we have added a figure describing the distribution of these descriptors. We are confident these will allow the readers to assess the kind of chemical space we base our analysis on.

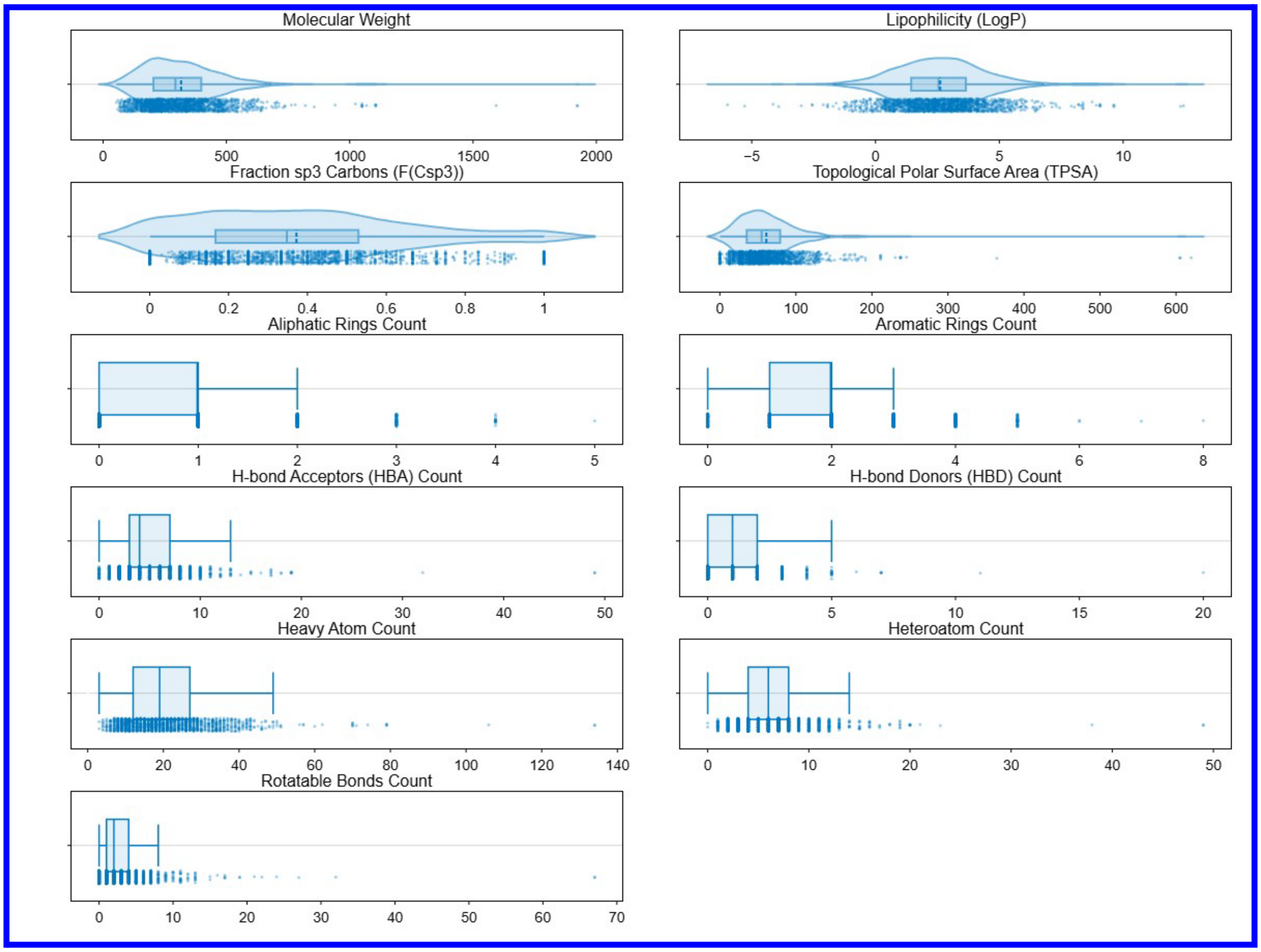

Figure S2: Boxplots of 11 descriptors of the synthesized products in this dataset. Descriptors were calculated with rdkit version 2024_03_3.[1]

A sentence to guide the reader to this data has been added to the introduction section (added text in blue):

Most transformations we receive failed to yield meaningful amounts of product before being submitted for screening. Thus, our dataset is skewed towards difficult reaction types and substrate combinations, where small changes to the reaction conditions or substrate structure lead to big changes in outcome. Descriptors of the products of the reactions screened in this dataset are shown in Figure S2 in the Supporting Information (SI). We often receive advanced intermediates as starting materials and our customers expect us to use them resourcefully for a maximum number of experiments. Conversely, reducing reaction scale too much can compromise result quality, specifically for heterogeneous reactions or those conducted close to the reaction medium's boiling point. Therefore, we try to reduce the number of experiments by increasing the hit-rate and quality.

a. Would it be possible to include a structure search in the online tool, so that the user can enter their structure of interest and the tool adjusts the results to be actually fitting for the user's structure (for example by filtering or weighing the results included in the output by how similar the substrates were to the user's input), without revealing the actual structures of the molecules in the dataset?

The reviewer raises an interesting feature request to the web app, enabling users to adjust results based on their input structure's similarity to dataset substrates. Implementing it requires extensive engineering effort, necessitating the creation of several new work packages including integrating structural drawing capabilities, developing on-the-fly molecular descriptor calculation, building a robust similarity search engine against the entire dataset, and designing the front-end logic for weighted result filtering. As such, the proposed structure search functionality is beyond the scope of the current work and the resources allocated for this publication. We encourage the research community to fork the repository and introduce the proposed structure-based filtering and weighing feature based on the reacting functional groups. We believe this contribution would be a valuable and logical extension of the tool.

b. On page 12, line 52 the authors mention sampling at three different time/temperature points. Why are neither temperature nor time reported with the conditions? As these factors can be crucial for a successful transformation, it would be nice to have a similar analysis as for the other factors, or at least a statement of the respective temperature and time in the box that shows when you hover over a data point in your webtool.

We thank this reviewer for their suggestion to improve our web app and agree that display of the time and temperature profile is insightful for the practitioner. Therefore, we have updated our dataset and webtool to surface this information. Now, the exact

time-temperature profile of each data point is displayed in the section Reaction Conditions when hovering.

In the section Yield Calculation and Evaluation in the Methods section we have added a comment that the specific temperature-time profiles are available in the web app.

> Typically, we sample each reaction at three different time/temperature points. The maximum observed value is then included in the final dataset. The exact time-temperature profile is displayed in the web app when hovering over a data point.
> We only include plates for which at least one set of conditions resulted in a peak with more than 10 area percent normalized product.

c. On page 5, line 20 the authors state that the internal tool has more advanced features. While this is totally understandable from an economic point of view for the company, I don't think it is a good statement to make in this paper. What does the user get from it, except the feeling of "not getting the full picture"?

We agree with this reviewer that our statement about an internal version with more features does not add value to the reader. We have therefore omitted the sentence in question.

> Our tool allows the user to visualize the data by selecting functional groups, reaction and reagent types. The z-score distribution for the best reagents is then presented as a series of box-plots. ~~Our internal version of this tool furthermore allows for the slicing of the data by substructure, project or manually selected transformation subsets.~~

3. *The authors state that they limit the number of z-scores to be included per category and transformation, because they only want to look at optima. However, there are many negative z-scores in the plots. Do those all stem from transformations where one positive outlier raised the mean area% so much that the mean is higher than the area% of some of the samples within the top 5? Independent on the answer, this phenomenon should be discussed in the manuscript.*

For a z-score to be negative, the only requirement is that a reagent underperforms its peers in a given transformation. Having one or a few positive outliers on a plate is one way to obtain negative z-scores, but outliers will not increase the average outcome a lot. A z-score becomes large negative when almost everything works on a plate except for the reagent in question. This results in a high mean product area% which is subtracted from a low observed area. Since the standard deviation is also small, the

resulting z-score will be large and negative. As a conclusion, positive outliers on a plate result in large and positive z-scores compensated by a large number of mildly negative z-scores for the rest of the plate. Conversely, negative outliers on a plate result in large negative z-scores compensated by a large number of mildly positive z-scores for the rest of the plate.

The methodology section has been supplemented with a comment on negative z-scores (new text in blue).

> We allow the user to control the number of top z-scores to be included, because the user's preference may vary. Using a large number of z-scores will favor reagents that work robustly under a variety of conditions whereas picking only the top one or two z-scores will favor reagents that work outstandingly well, but only under certain conditions. This equips the user with more granular control, especially in combination with setting a minimum number of transformations in which a reagent has to have been tested. While we only include the top z-scores of a reagent, we do not require them to be positive. This means that positive as well as negative performance of a reagent in different transformations is taken into account. We aim to limit the influence of outliers on the reagent ranking by using the median of the z-scores instead of the average.

4. Some parts of the text would profit from minor improvements and clarifications:
a. p. 6, l. 14-15: This is confusing, the previous paragraph listed SPhos as the second best behind DiMeIHept Cl. If the previous work contrasts this, the authors should provide more context.

We thank this reviewer for spotting the discrepancy regarding the ranking of SPhos across two figures. We added an explanation for the stark difference between the placement of SPhos as a ligand and the one of SPhos Pd(crotyl)Cl below Figure 4:

> It is striking that SPhos as a ligand ranks so much higher when the pre-catalyst type is not considered. The reason for that is that we use SPhos in the form of SPhos Pd(allyl)OTf in two Buchwald-Hartwig couplings of secondary amines. In these it performed so well that it would have topped the list with a median z-score of 1.36, if it had occurred in the dataset five times or more. This is the default setting for the minimum number of transformations a reagent has to occur in for it to be shown. SPhos Pd(crotyl)Cl only achieves a median z-Score of 0.55. Pooling the two results in the observed overall median z-Score of 0.95 for SPhos as a ligand. It is known that steric bulk on the allyl ligand prevents Pd(I)-dimer formation[32] as well as the switch from chloride as a counterion to triflate[33]. We believe, however, that data from two transformations is not enough

to make a general statement, especially since in none of the transformations the two SPhos precatalysts were compared head-to-head.

b. p. 6, l. 20-21: Do the authors have any explanation where the stark difference for QPhos originates?

We were surprised by this as well and we do not have an explanation. Before we had this comprehensive analysis and the amount of data required for it, we relied on our own analysis of literature Buchwald-Hartwig reactions (Chem. Sci., 2020,11, 13085) and literature searches for reaction substructure to design our plates. Thus, QPhos Pd(crotyl)Cl (purchased from Johnson Matthey) was present often. While we clearly observe hits with the latter and thus the catalyst is active *per se*, the hits are not as common and exceptional compared to other catalysts. Since we almost exclusively use this QPhos pre-catalyst, albeit different batches over time, we cannot exclude an issue with this specific form of the catalyst. We recently received QPhos Pd G6 Br and it remains to be seen if this pre-catalyst outperforms the former, as t seen for Catacxium A in Suzuki-Miyaura reactions.

c. p. 8, l. 6 & 13: It is not obvious here that "Fitzner et al." is the author´s own previous work, while they just refer to the same paper as "our own work" or "our previous analysis" on the page before. They also suddenly write of the authors in the third person. It should be made clear that they are referring to the same paper as before and that it is their own work.

We have clarified the common authorship of one of the co-authors in the section "Comparing our Buchwald-Hartwig Recommendations with the Literature" (adjusted text highlighted in blue):

> Fig. 5: Comparison of the best ligands for Buchwald-Hartwig cross-couplings for aliphatic amines, amides and anilines reacting with aryl halides. All ligand recommendations are based upon at least five transformations. Not all recommendations previously published by us when analyzing literature data ~~by Fitzner et al~~.[26] are included in our graph, as we lack sufficient data in our dataset. [...]
>
> When chemists set up and optimize new reactions, they typically consult scientific literature and reaction databases. To evaluate the difference between using literature review and our real-world HTE data-driven tool, we compared the best ligands by z-score to the evaluation of Buchwald-Hartwig literature published in our previous work ~~by Fitzner et al~~.[26] For this we used their decision

tree and compared their top three recommendations for various aliphatic amines to our best performing ligands.

d. p. 8, l. 26-27: The statement of this sentence is not clear to me. "Also" suggests a follow-up to a previous statement, but there is no relatable statement before. Which difference in substrate complexity do the authors mean? And compared to what are the solvent and base preferences for the coupling of unhindered secondary aliphatic amines differing?

We apologize for the ambiguous phrasing. The “also” phrase relates to similar observations in the Suzuki Miyaura case study that follows later in the manuscript. We refer to the higher complexity in our substrate set compared to the molecules most represented in literature.

The last paragraph of section Comparing our Buchwald-Hartwig Recommendations with the Literature was updated (adjusted text highlighted in blue):

> The perceived increase ~~difference~~ in substrate complexity compared to typical literature substrates may ~~also~~ be the reason why we observe differing solvent and base preferences for the coupling of unhindered secondary aliphatic amines.

e. p. 9, l. 49-50: Can the authors back up the claim "significant influence" with statistical analysis? What kind of matched pairs do they refer to?

We thank this reviewer for the comment. After educating ourselves about the strict definition of matched pairs, we weakened the claim and added a pointer to the different performance of
Catacxium A pre-catalysts. The corrected statement reads with changes highlighted in blue:

> We again observe~~find~~ an ~~significant~~ influence of the pre-catalyst activation system for example in the different performance of pre-catalysts containing Catacxium A ~~in matched pairs~~.

f. p. 10, l. 34: Are those the absolute best base-solvent combinations, or are there different optimal base-solvent combinations for different catalysts?

The depicted base-solvent combinations are irrespective of the used catalyst. However, when the catalyst is introduced as a variable, the optimal base-solvent

combination depends on the catalyst. The sample size of each triplet is small, therefore more actionable conclusions can be derived from the solvent-base doublet.

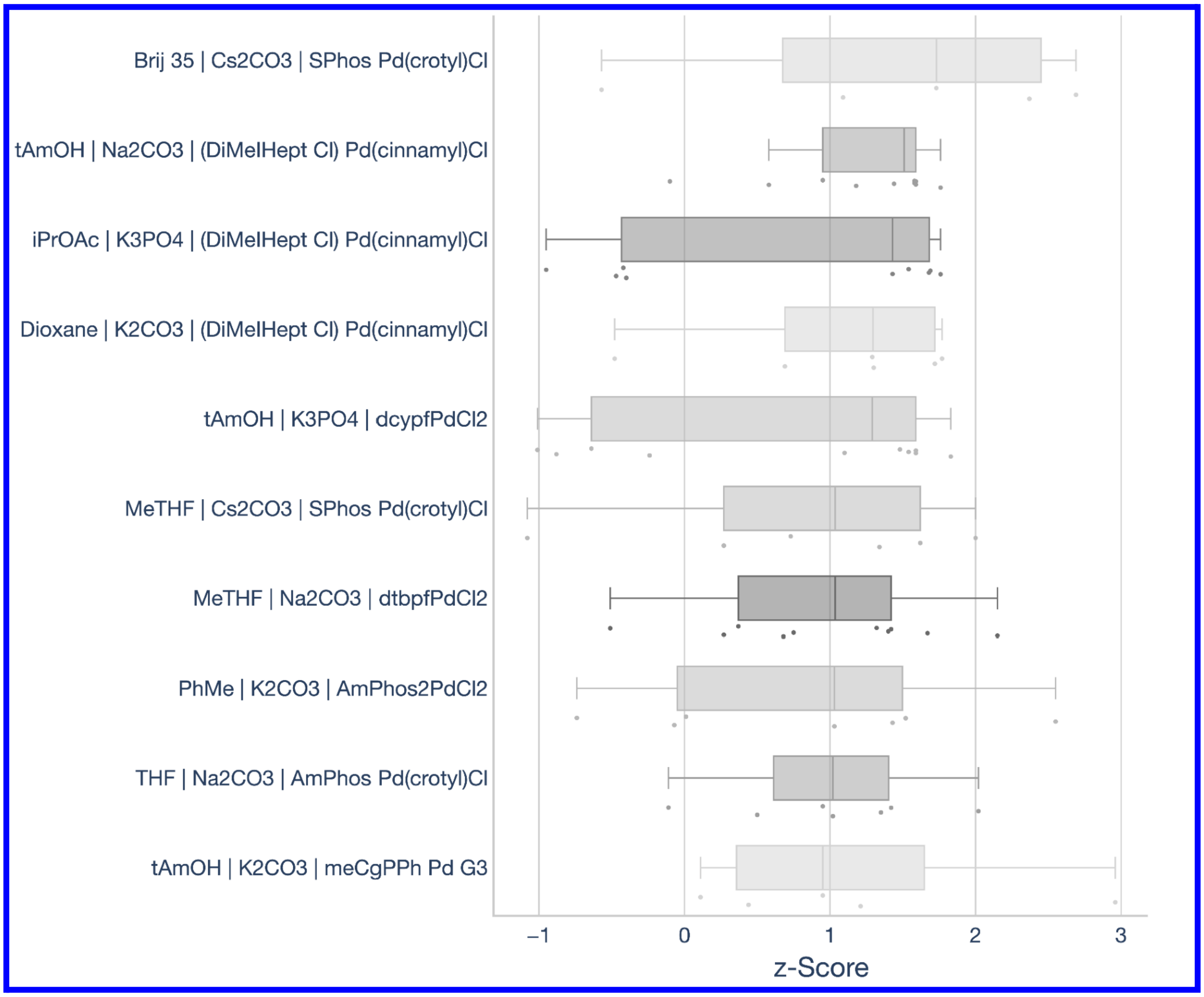

g. p. 10, l. 44-45: Why does only Cs2CO3 lead to hydrolysis when used as aqueous solution? It seems like this depends on the substrate and not on Cs2CO3? Or were all other inorganic bases weaker?

Indeed, the claim we made was misleading.Hydrolysis primarily depends on the substrate and occurs with bases other than Cs_2CO_3. We, and very likely others before us, observed however, that the hydrolysis extent can be reduced by either having little water present, using weaker bases or using an apolar solvent that is poorly water-miscible. In order to clarify this, we amended the text (changes in blue):

> In our dataset, inorganic bases are used as aqueous solution with the exception of Cs_2CO_3 where we commonly either use no water or add only a few equivalents. We use these conditions in Suzuki-Miyaura couplings to prevent

hydrolysis of sensitive functional groups like esters. Another way to achieve this is to use a weaker base like aqueous $NaHCO_3$ or a less polar organic solvent like toluene or isopropyl acetate. The prominent representation of Cs_2CO_3, $NaHCO_3$ and water-immiscible solvents ~~all three combinations~~ in Figure 7 is likely attributed to the prevalence of sensitive substrates we receive for screening.

h. p. 10, l. 48: "The prominent representation of all three combinations in Figure 7" is not comprehensible to me, as there is no information on the water content in the figure. Which three combinations are meant?

To avoid over-complicating Figure 7, we intentionally chose not to display the secondary solvent for Suzuki-Miyaura reactions. Instead, the focus was placed on our established procedure of using Cs_2CO_3 in its dry form versus an aqueous solution, as applied to the other inorganic bases. The tool does, however, allow for the visualization of secondary solvents, and the resulting chart is as follows:

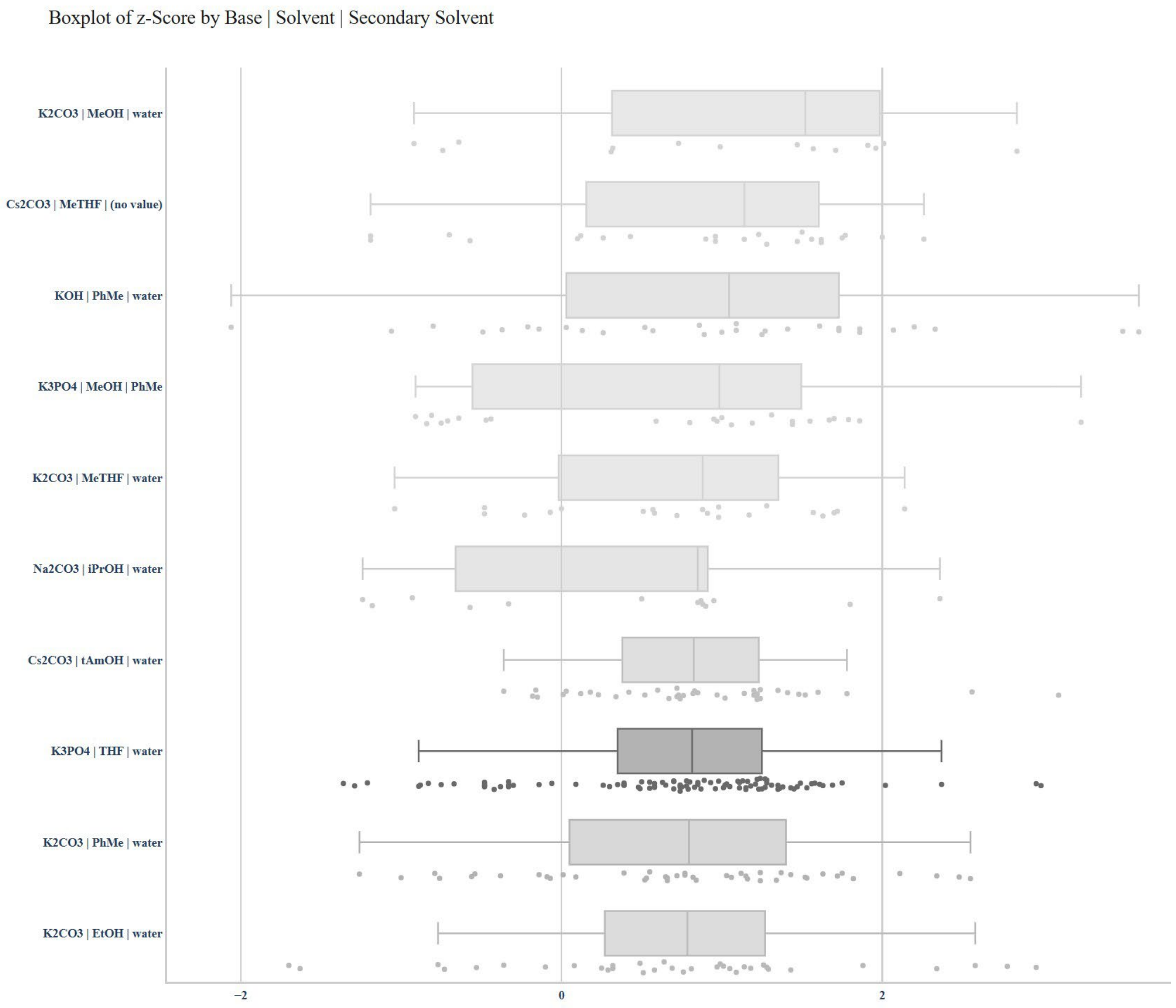

The chart is of limited utility because methanol is frequently used as a co-solvent with toluene. This practice further fragments the categories and fails to indicate whether water was also present, which is typically the case. Although the specific ranking is affected, the core assertion regarding the use of Cs_2CO_3, weak inorganic bases like $NaHCO_3$, and apolar solvents coupled with strong bases such as KOH remains valid.

We clarified the text to read:

> For Suzuki-Miyaura couplings the right combination of solvent and base can be as important as the right catalyst (Fig. 7). One reason may be the importance of bridging the polarity differences between the aryl boronate, aryl halide and the catalyst.[41] To facilitate transmetalation of the boron species, water is routinely added. In our dataset, inorganic bases are used as aqueous solution with the

> exception of Cs_2CO_3 where we either use no water or add only a few equivalents. We use these conditions in Suzuki-Miyaura couplings to prevent hydrolysis of sensitive functional groups like esters. Another way to achieve this is to use a weaker base like aqueous $NaHCO_3$ or a less polar organic solvent like toluene or isopropyl acetate. The prominent representation of Cs_2CO_3, $NaHCO_3$ and water-immiscible solvents ~~all three combinations~~ in Figure 7 is likely attributed to the prevalence of sensitive substrates we receive for screening.

i. p. 12, l. 50: Re-normalized to what? This needs to be elaborated.

To clarify, the raw peak areas are normalized to exclude injection peaks and all peaks resulting from reagents or internal standards. This is done by re-calculating peak area percentages from the peak areas while omitting all peaks that do not result from starting materials or products.

We have added a clarifying sentence to the methodology section:

> To arrive at a robust measure of reaction outcome, we instead opt to transform peak area percentages. We re-normalize the measured peak area percent to exclude injection peaks and all peaks resulting from reagents or internal standards. This is accomplished by re-calculating peak area percentages from the raw peak areas while omitting all peaks that do not result from starting materials or products.

j. p. 13, l. 8-9: Do the authors have data to back up the claim that the normalized peak area percentages are more robust than native yields?

We would like to clarify that we compare re-normalized peak area percentages with relative yields, not with isolated [native], resulting from the actual dosed weights of internal standard and limiting starting material as well as peak areas of product and internal standard. This relative yield can be converted into an actual yield by co-injecting known amounts of internal standard and product reference. Omission of peaks unrelated to the transformation in question, e.g. injection peaks, internal standards, solvents, leads to a more reliable representation of the reaction performance in our experience also when compared to relative yields. These are useful, but are prone to produce large outliers due to the way they are calculated.

Here the modified text (changes in blue):

Nevertheless, we found these normalized peak area percentages to be more robust than relative yields. We calculate the latter by multiplying the ratio of product peak area and internal standard with the ratio of actual weights dosed of internal standard and limiting starting material. This relative yield is useful for comparing hits within a transformation, but also prone to produce large outliers introduced through weighing or integration errors. T~~and t~~hus we prefer to use normalized peak area percentages ~~them~~ to evaluate and compare reaction outcomes within a chemical transformation.

k. *In the Dataset Information (p. 44 of SI), the meanings of the different categories/values should be explained once. What do you mean by Reagents, is a solvent classified as a reagent here? What do the authors mean by Functional Groups, those participating in the bond formation or those present in the substrates? What does AREA_TOTAL_REDUCED mean? And how can the authors have more "Most frequent FGs" than reactions, if there is only one FG in the reaction (e.g. as seen for Cyanation, 264 reactions, 1056 ArBr)?*

We thank the reviewers for spotting the inconsistency of functional group and reaction count. There was an error in the code that led to an overestimation of the functional group count. This has been fixed and the tables in the section Dataset Information per Reaction Type in the Supporting Information (SI) have been corrected.

Apart from that, we have clarified the meaning of the terms in the section Dataset Information per Reaction Type in the Supporting Information (SI):

The following definitions apply:

- **Reactions:** Represents the total number of chemical transformations for each specific reaction type.
- **Reagents:** Encompasses all components involved in the reaction, including additives, bases, catalysts, coupling reagents, ligands, and solvents.
- **Functional Groups:** Refers to the specific functional groups that react.
- **AREA_TOTAL_REDUCED:** This is the re-normalized peak area percentage, excluding peaks from the injection, reagents, or internal standards.

oc-2025-02031g.R2

Name: Peer Review Information for "Which Reaction Conditions Work on Drug-Like Molecules? Lessons from 66,000 High-Throughput Experiments"

Second Round of Reviewer Comments

Reviewer: 1

Comments to the Author

The authors have comprehensively and diligently addressed the reviewers' comments and suggestions. In the rebuttal letter, they provided detailed point-by-point explanations of the specific measures taken to respond to the more than ten major concerns raised by the two reviewers. From the updated manuscript content, it is evident that most of these revisions have been implemented: these include adding discussions in the manuscript regarding the selection bias of the dataset, the transferability of HTE conditions to conventional-scale synthesis, and the applicability of the z-score method to non-normally distributed data; incorporating a case study in the results section comparing recommendations with literature and an AI model; elaborating on the data processing workflow, rationale for parameter selection, and explanations of yield calculations in the methods section; enhancing the quality of all figures, including redrawing Figure 1, optimizing the presentation of boxplots and the decision tree diagram, and adding axis labels and detailed captions; and supplementing the Supporting Information with distributions of substrate descriptors, histograms of data distributions, and detailed results of the comparison experiments. Furthermore, the authors updated the online tool per the suggestions, adding features for user data upload and display of reaction time-temperature information. These revisions have significantly improved the paper's rigor, transparency, and readability, effectively addressing the core issues raised by the reviewers concerning method validation, data quality, interpretation of results, and tool utility.

While the authors have made substantial revisions, the paper could benefit from further refinement in the following areas:

First, regarding method validation, the authors cite a preprint article that has not yet undergone peer review as the primary basis for the correlation between LC-MS peak area percentages and isolated yields. To strengthen the reliability of the conclusions, it is recommended that the authors include, at a minimum, a small internal validation dataset within the Supporting Information of the current paper. For instance, for the reaction types covered in this work, such as Buchwald-Hartwig or Suzuki-Miyaura, several representative substrates could be selected to directly compare the normalized peak area percentages from HTE experiments with the isolated yields measured subsequently upon gram-scale synthesis, accompanied by a scatter plot and correlation coefficient. This would provide more direct evidence to readers of the feasibility of the chosen proxy metric, reduce reliance on external unpublished data, and make the argument more self-contained and complete.

Secondly, the discussion concerning data selection bias and its impact could be deepened. While the authors correctly note the dataset's bias towards "difficult reactions" and analyze the potential trend of recommendations favoring more modern and expensive reagents, a quantitative assessment of how this bias specifically affects the generalizability of the recommended conditions is still lacking. It is suggested that a supplementary analysis be conducted: select a set of classic substrate pairs with relatively simple structures and ample literature reports from public databases, then apply the recommendations from this tool and classic conditions from the literature for prediction or limited experimental testing, comparing their expected performance differences. This analysis could be placed in the Supporting Information, aiming to visually demonstrate the tool's applicability boundaries in "simple" versus "difficult" chemical spaces. This would help users more clearly understand the tool's strengths and limitations, enabling them to make more informed decisions.

Finally, the description of the online tool's functionality and accessibility could be further clarified. While the authors have added a user data upload feature, guidance on how to prepare data, format requirements, and how uploaded data interacts with the built-in dataset (e.g., analyzed side-by-side or integrated) remains unclear. It is recommended to add a brief data upload and usage guide in the Methods section of the paper or in the README file of the tool's GitHub repository, explaining the recommended data structure, required fields, and the interpretation of results after upload. Simultaneously, the limitation

that the tool cannot provide filtering based on substrate structural similarity should be explicitly mentioned in the Discussion section of the paper and listed as a potential future development direction to manage user expectations and encourage community contributions.

Reviewer: 2

Comments to the Author

I thank the authors for the additional work they put into their manuscript, and I think that it is considerably improved over their initial submission. The authors have demonstrated the effectiveness of their tool by adding key experimental data on a direct comparison experiment between sets of reactions designed independently using either the presented tool, literature data (SciFinder), or an AI model. Although the tool was only successful in one of the two case studies, the other methods were not successful at all, emphasizing the difficulty of the imposed tasks and thus strengthening the impact of the tool.

Additionally, several passages and figures that were previously ambiguous or unclear have been expanded or accompanied with a legend or description to explain more details, improving comprehensibility. Drawbacks and Limitations of the presented method have been clarified and are transparently described in the manuscript.

The web application has been upgraded to include more details on temperature and reaction time and now features the possibility to complement the built-in dataset with additional reaction data locally.

I am pleased to hear that the authors agree that more detailed access to the dataset would drastically improve the usability of the tool for the scientific community, however they made clear that this is not possible due to the company's concerns that information on proprietary structures could otherwise be extracted through reverse engineering. As this cannot be changed, I think the restricted tool is still valuable for the community.

I therefore recommend accepting this manuscript after two minor improvements:

1. In the discussion of the new comparison study, a 4-pyridyl isomer is described to be formed as a byproduct through a benzyne mechanism. Given the broad audience of ACS Central Science, I would suggest including a small reaction scheme to accompany the discussion.

2. There are several occurrences of the placeholder “SX” in the text of the SI where the number of a supporting figure should be referenced.

Author's Response to Peer Review Comments:

Response to Decision Letter

Editorial

Cover Art: Please prepare a short caption of 50 words or fewer to explain the significance of the research the front cover depicts. The caption will appear next to your cover on the journal website if accepted/approved. Please upload the caption as text only in Word .doc format as a “Cover Art Caption” file (no header required).

— A cover art has been submitted. The corresponding caption, also submitted separately as Cover Art Caption.doc file reads:

> This cover depicts a chemist navigating reaction space provided with a map of high-quality starting points based on z-score analysis of 66,000 HTE reactions using an interactive web app. This tool allows chemists dealing with complex substrates to overcome conventional biases and accelerate their ascent on the golden path to the reaction optimum. (Image: Vincenzo Pani)

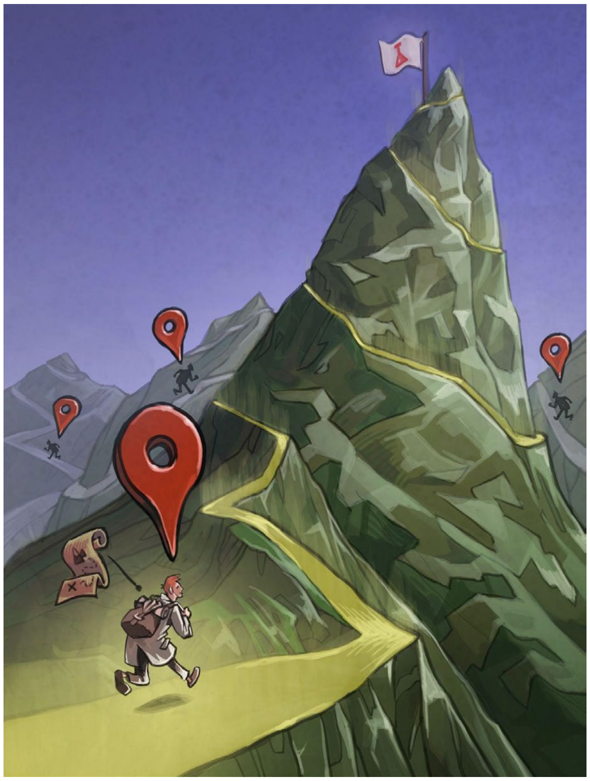

Reviewer: 1

Recommendation: Publish in ACS Central Science after minor revisions noted.

Comments:

The authors have comprehensively and diligently addressed the reviewers' comments and suggestions. In the rebuttal letter, they provided detailed point-by-point explanations of the specific measures taken to respond to the more than ten major concerns raised by the two reviewers. From the updated manuscript content, it is evident that most of these revisions have been implemented: these include adding discussions in the manuscript regarding the selection bias of the dataset, the transferability of HTE conditions to conventional-scale synthesis, and the applicability of the z-score method to non-normally distributed data; incorporating a case study in the results section comparing recommendations with literature and an AI model; elaborating on the data processing workflow, rationale for parameter selection, and

explanations of yield calculations in the methods section; enhancing the quality of all figures, including redrawing Figure 1, optimizing the presentation of boxplots and the decision tree diagram, and adding axis labels and detailed captions; and supplementing the Supporting Information with distributions of substrate descriptors, histograms of data distributions, and detailed results of the comparison experiments. Furthermore, the authors updated the online tool per the suggestions, adding features for user data upload and display of reaction time-temperature information. These revisions have significantly improved the paper's rigor, transparency, and readability, effectively addressing the core issues raised by the reviewers concerning method validation, data quality, interpretation of results, and tool utility.

— We thank the reviewer for their overwhelming feedback, acknowledging the improvements brought to this version and their recommendation to publish in *ACS Central Science*.

While the authors have made substantial revisions, the paper could benefit from further refinement in the following areas:

First, regarding method validation, the authors cite a preprint article that has not yet undergone peer review as the primary basis for the correlation between LC-MS peak area percentages and isolated yields. To strengthen the reliability of the conclusions, it is recommended that the authors include, at a minimum, a small internal validation dataset within the Supporting Information of the current paper. For instance, for the reaction types covered in this work, such as Buchwald-Hartwig or Suzuki-Miyaura, several representative substrates could be selected to directly compare the normalized peak area percentages from HTE experiments with the isolated yields measured subsequently upon gram-scale synthesis, accompanied by a scatter plot and correlation coefficient. This would provide more direct evidence to readers of the feasibility of the chosen proxy metric, reduce reliance on external unpublished data, and make the argument more self-contained and complete.

— We acknowledge the reviewer's careful scrutiny regarding the correlation between the surrogate yields observed in High-Throughput Experimentation (HTE) and isolated synthetic yields. To preface, while we did not find peer-reviewed studies that delineate quantitatively the relationship between peak area percentages and isolated yields, we find it is common practice also outside HTE to guide reaction optimization using peak area percentages and thus widely accepted that these have predictive power for reaction outcomes.

Next, we would like to comment on the necessary for the requested validation dataset.

First, to clarify, the cited preprint (Ref 43) is a direct product of our laboratory and describes the validation experiments the reviewer is requesting—comparing normalized LC-MS area percentages with isolated yields for two sets of palladium-catalyzed cross couplings. Because those experiments were conducted using the same platforms, protocols, and substrate classes as the current work, we believe that this should be sufficient. Further evidence from generating an additional "small dataset" for this revision would necessarily have been anecdotal in nature and the required effort gone beyond the time we had available between the two revisions (also in light of Christmas break). .

Second, to provide an independent source for validation, we refer to a methodic scale-up validation performed by the HTE group of Merck/UPenn in 2014 (*Aldrichimica Acta* **2014**, *46*, 3, 71-80). They benchmarked a Suzuki–Miyaura reaction across three scales. They found:

> "[...] at three different reaction scales (5 µmol in 250 µL vials, 20 µmol in 1 mL vials, and 0.16 mmol in 8 mL vials), we were able to validate that the platform indeed gives the same result on scale up. The 6x2x2 screen was carried out in triplicate utilizing six diverse ligands, two solvents, and with and without water, and resulted in good reproducibility (eq 1).[8a] The excellent reaction fidelity among the different reaction scales allows the chemist to conduct 24 discrete reactions at the 25 µmol scale [...] and to be confident in the ability to scale the screening hits with similar results."

This consistency between yields obtained in 1 mL HTE vials used in our study and larger scale-ups ensures that the catalytic trends we report are directly applicable to preparative laboratory synthesis.

Lastly, the use of LC-MS area percentages as a robust proxy for yield in HTE is a well-established and broadly adopted paradigm in both industrial and academic high-throughput laboratories for a decade (see References 10, 11; *ACS Med. Chem. Lett.* **2017**, *8*, 6, 601–607; *Org. Process Res. Dev.* **2019**, *23*, 6, 1213–1242). This metric is often preferred during screening as it provides a direct measure of catalytic efficiency, independent of the variable material loss inherent in small-scale isolation and purification. Isolation on a small scale (<10 mg) introduces significant variability due to material loss during work-up and purification. It goes without saying that any organic chemistry methodology paper uses calibrated spectroscopic yields as indication before isolating reaction products in a substrate scope.

Secondly, the discussion concerning data selection bias and its impact could be deepened. While the authors correctly note the dataset's bias towards "difficult reactions" and analyze the potential trend of recommendations favoring more modern and expensive reagents, a quantitative assessment of how this bias specifically affects the generalizability of the recommended conditions is still lacking. It is suggested that a supplementary analysis be conducted: select a set of classic substrate pairs with relatively simple structures and ample literature reports from public databases, then apply the recommendations from this tool and classic conditions from the literature for prediction or limited experimental testing, comparing their expected performance differences. This analysis could be placed in the Supporting Information, aiming to visually demonstrate the tool's applicability boundaries in "simple" versus "difficult" chemical spaces. This would help users more clearly understand the tool's strengths and limitations, enabling them to make more informed decisions.

— We appreciate the reviewer's request for a deeper analysis of the tool's applicability boundaries. While we acknowledge that our dataset is biased toward "difficult" drug-like substrates as we point to multiple times in the manuscript, we contend that this bias is a deliberate and necessary feature for generating actionable conclusions.

We argue that traditional datasets are often dominated by "simple" substrates that occupy a "solved" chemical space; such substrates frequently reach a "performance ceiling" where multiple condition sets yield nearly identical results, thus providing few grounds for discriminating literature-research from our recommendations. There may be rare cases where more modern reaction conditions perform worse than "classical" ones for simple cases, but the more likely outcome in our view is that many reaction conditions perform similarly well. While more modern catalysts may cost more, the convenience and time saving of using a simple tool like z-score analysis still justifies its use in many settings.

The z-score method relies on the variance of the dataset to highlight conditions that outperform the mean. In a "simple" dataset (e.g. lack of sterical hindrance, few functional groups) where the mean conversion is high and the variance is low (because almost all conditions work), the z-scores for even the best catalysts would shrink toward zero, thus eroding the signal. In other words, the mean yield (μ) is high and the variance (σ) is extremely low. By focusing on "difficult" substrates, where the mean yield is lower and the variance between catalysts is higher, our tool maximizes the signal-to-noise ratio, allowing the z-score to clearly identify conditions that outperform the mean.

While this may lead to recommendations of more "sophisticated" or expensive catalyst systems, these are precisely the conditions required to overcome the increased

activation barriers of complex molecules. For "simple" substrates, while our tool may "over-engineer" a solution, the recommended conditions are highly likely to remain effective, albeit potentially less economical than classic literature methods.

Finally, the description of the online tool's functionality and accessibility could be further clarified. While the authors have added a user data upload feature, guidance on how to prepare data, format requirements, and how uploaded data interacts with the built-in dataset (e.g., analyzed side-by-side or integrated) remains unclear. It is recommended to add a brief data upload and usage guide in the Methods section of the paper or in the README file of the tool's GitHub repository, explaining the recommended data structure, required fields, and the interpretation of results after upload. Simultaneously, the limitation that the tool cannot provide filtering based on substrate structural similarity should be explicitly mentioned in the Discussion section of the paper and listed as a potential future development direction to manage user expectations and encourage community contributions.

— We thank the reviewer for the recommendation to extend the description of the data upload. We have elaborated the README file of the GitHub repository. We are now listing the file format, each column's data type and how the data is handled i.e. the uploaded dataset replaces the existing dataset. We have also added a limitations and future development section to the README to make the current limitations that structures are not provided clear and show our openness to community contributions.

Regarding the suggestion to discuss the absence of structural similarity filtering, we believe with the updated README of the repository this should avoid any potential confusion. Furthermore we believe the current text addresses this by clearly defining the functional boundaries of the tool. By stating in the Discussion that the dataset is provided 'without substrate structures' and that recommendations are categorized by 'reaction type, reacting functional group and reagent category,' we have implicitly but clearly excluded structural similarity as a filtering criterion in the discussion. We feel that adding further explicit negatives to the Discussion may become redundant, as the tool’s architecture is already defined by these parameters.

Reviewer: 2

Recommendation: Publish in ACS Central Science without change.

Comments:

I thank the authors for the additional work they put into their manuscript, and I think that it is considerably improved over their initial submission. The authors have demonstrated the effectiveness of their tool by adding key experimental data on a direct comparison experiment between sets of reactions designed independently using either the presented tool, literature data (SciFinder), or an AI model. Although the tool was only successful in one of the two case studies, the other methods were not successful at all, emphasizing the difficulty of the imposed tasks and thus strengthening the impact of the tool.
Additionally, several passages and figures that were previously ambiguous or unclear have been expanded or accompanied with a legend or description to explain more details, improving comprehensibility. Drawbacks and Limitations of the presented method have been clarified and are transparently described in the manuscript.
The web application has been upgraded to include more details on temperature and reaction time and now features the possibility to complement the built-in dataset with additional reaction data locally.
I am pleased to hear that the authors agree that more detailed access to the dataset would drastically improve the usability of the tool for the scientific community, however they made clear that this is not possible due to the company's concerns that information on proprietary structures could otherwise be extracted through reverse engineering. As this cannot be changed, I think the restricted tool is still valuable for the community.
I therefore recommend accepting this manuscript after two minor improvements:

— We thank this reviewer for their positive evaluation and share their optimism about the relevance of this tool for the community.

1. In the discussion of the new comparison study, a 4-pyridyl isomer is described to be formed as a byproduct through a benzyne mechanism. Given the broad audience of ACS Central Science, I would suggest including a small reaction scheme to accompany the discussion.

— We agree that a scheme detailing our mechanistic rationale will be beneficial to the audience. In the Supporting Information we have added a scheme to explain the formation of the 4-arylated isomer via a benzyne mechanism.

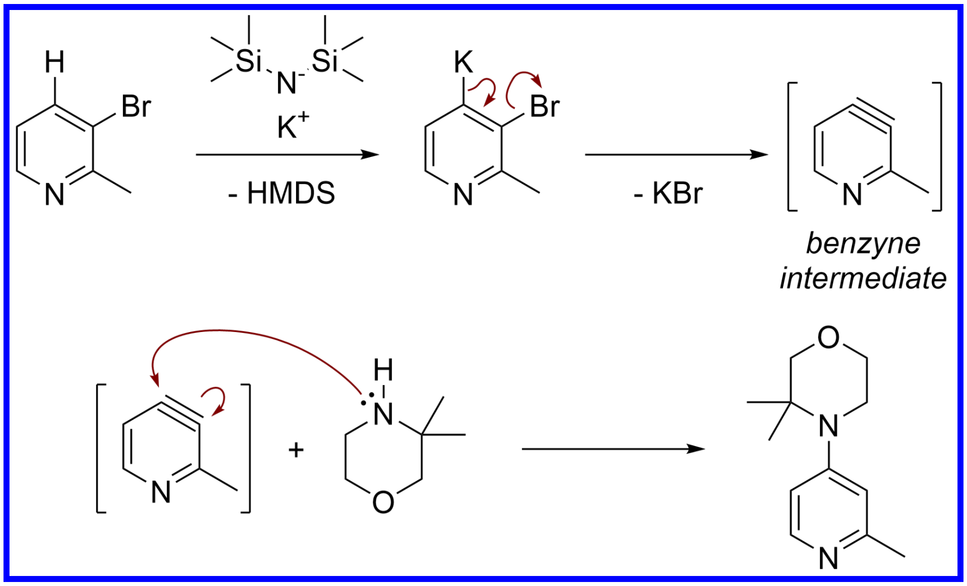

Equation S4: Proposed mechanism for the base-promoted formation of the 4-arylated isomer via a benzyne intermediate.

2. *There are several occurrences of the placeholder "SX" in the text of the SI where the number of a supporting figure should be referenced.*

— We thank the reviewer for their careful review. The placeholders have been replaced.
